# Spiders (Araneae) of Churchill, Manitoba: DNA barcodes and morphology reveal high species diversity and new Canadian records

**DOI:** 10.1186/1472-6785-13-44

**Published:** 2013-11-26

**Authors:** Gergin A Blagoev, Nadya I Nikolova, Crystal N Sobel, Paul DN Hebert, Sarah J Adamowicz

**Affiliations:** 1 Biodiversity Institute of Ontario, University of Guelph, Guelph, Ontario N1G 2W1, Canada; 2 Department of Integrative Biology, University of Guelph, Guelph, Ontario N1G 2W1, Canada

**Keywords:** Araneae, Biodiversity, COI, Cytochrome *c* oxidase subunit I, DNA barcoding, iBOL, Spiders, Subarctic, Arctic, Barcoding biotas

## Abstract

**Background:**

Arctic ecosystems, especially those near transition zones, are expected to be strongly impacted by climate change. Because it is positioned on the ecotone between tundra and boreal forest, the Churchill area is a strategic locality for the analysis of shifts in faunal composition. This fact has motivated the effort to develop a comprehensive biodiversity inventory for the Churchill region by coupling DNA barcoding with morphological studies. The present study represents one element of this effort; it focuses on analysis of the spider fauna at Churchill.

**Results:**

198 species were detected among 2704 spiders analyzed, tripling the count for the Churchill region. Estimates of overall diversity suggest that another 10–20 species await detection. Most species displayed little intraspecific sequence variation (maximum <1%) in the barcode region of the cytochrome *c* oxidase subunit I (COI) gene, but four species showed considerably higher values (maximum = 4.1-6.2%), suggesting cryptic species. All recognized species possessed a distinct haplotype array at COI with nearest-neighbour interspecific distances averaging 8.57%. Three species new to Canada were detected: *Robertus lyrifer* (Theridiidae), *Baryphyma trifrons* (Linyphiidae)*,* and *Satilatlas monticola* (Linyphiidae). The first two species may represent human-mediated introductions linked to the port in Churchill, but the other species represents a range extension from the USA. The first description of the female of *S. monticola* was also presented. As well, one probable new species of *Alopecosa* (Lycosidae) was recognized.

**Conclusions:**

This study provides the first comprehensive DNA barcode reference library for the spider fauna of any region. Few cryptic species of spiders were detected, a result contrasting with the prevalence of undescribed species in several other terrestrial arthropod groups at Churchill. Because most (97.5%) sequence clusters at COI corresponded with a named taxon, DNA barcoding reliably identifies spiders in the Churchill fauna. The capacity of DNA barcoding to enable the identification of otherwise taxonomically ambiguous specimens (juveniles, females) also represents a major advance for future monitoring efforts on this group.

## Background

Arctic ecosystems, especially those positioned on transition zones, are recognized as areas where the impacts of climate change will be observed first [1]. Despite this fact, the baseline knowledge of species composition needed to monitor biodiversity change is limited for most animal groups. Because it sits at the juncture of three ecoregions, and possesses a strong research infrastructure, Churchill provides a strategic setting for a long-term monitoring program in the Canadian arctic. As a result, it was selected as a site to demonstrate how a comprehensive DNA barcode reference library [2] can both extend understanding of current biodiversity and facilitate future biomonitoring programs. Recent studies of several arthropod groups at Churchill, coupling morphological and DNA barcode analysis, have revealed unexpectedly high diversity and many undescribed species [2-10]. These results have reinforced the need for additional molecular work on the fauna of this region. The present study responds to this need for a key group of invertebrate predators—spiders.

Spiders (Araneae) are a diverse order of arthropods with more than 44,000 described species [11]. Because of their importance as predators in many terrestrial settings, they have the potential to reveal subtle changes in environmental variables [12-14]. Early work in the Churchill region indicated that spiders were one of the most abundant terrestrial arthropod groups [15], but little information has been available on their diversity. The first study of its fauna indicated the presence of 31 taxa, but just 19 were identified to a species level [15]. Two linyphiids, *Pytyohyphantes subarcticus*[16] and *Wabasso quaestio*[17], were subsequently described from Churchill. Information on the local spider fauna was also extended through taxonomic studies on particular genera [18-20] and a faunal study for Manitoba [1]. Although 483 spiders are known from this province, just 64 of these species have been reported from the Churchill area.

This study provides a DNA barcode reference library for the spiders of Churchill, based upon six years of collection activity. It additionally investigates how well the morphological species concept in spiders corresponds with sequence clusters in the DNA barcode region of the cytochrome *c* oxidase subunit I (COI) mitochondrial gene [21,22]. The results indicate the presence of 198 species of spiders at Churchill, and establish the close correspondence between sequence clusters at COI and described species. This latter result indicates that DNA barcoding is a very effective identification tool for the spider assemblage at this locality. This study also extends progress toward a comprehensive DNA barcode reference library for the biota of the Churchill region [2-10,23], an effort which is creating new opportunities for ecological research and monitoring programs.

## Methods

### Collection of spiders

**Figure 1:**
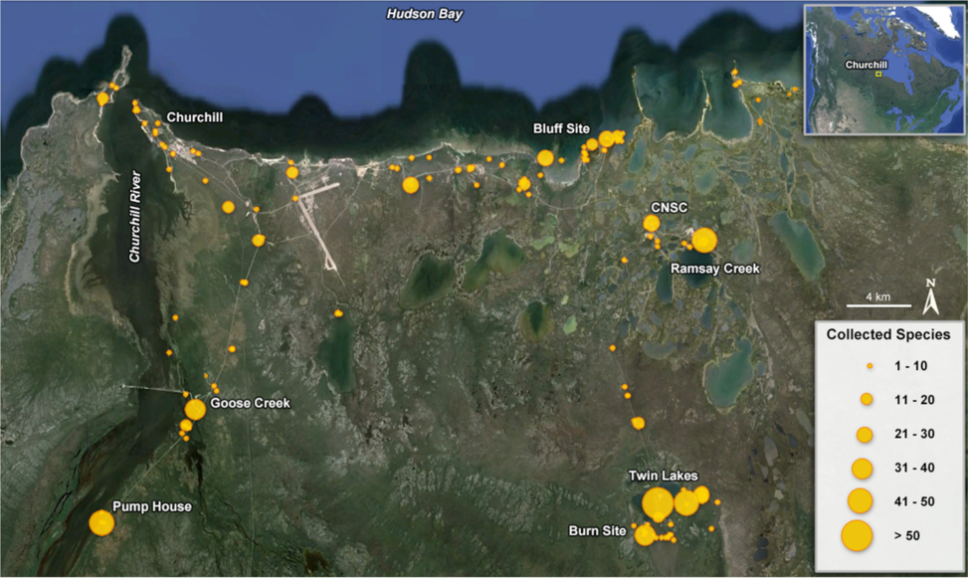
Map showing sites in the Churchill region that were sampled for spiders.

Spiders were collected during the snow-free months over a six-year interval from a wide range of habitats near Churchill using varied methods (Figure 1). These efforts resulted in the collection of 410 specimens from July 1-August 5, 2005; 517 from August 5-Sept 6, 2006; 548 from June 8-August 21, 2007; 32 from May 30-November 3, 2008; 1411 from July 17-August 15, 2009; and 547 from June 30-August 25, 2010. Most specimens were obtained through general collecting efforts by field course students and summer researcher assistants, but GAB carried out targeted sampling of spiders from July 17-August 2, 2009.

Collections were primarily made along Goose Creek Road, Cape Merry, Launch Road, Churchill Northern Studies Centre, and Twin Lakes (Figure 1). These collections (3465 specimens) were augmented with a small sample (41 specimens) from Wapusk National Park, Manitoba, producing a total of 3506 specimens. Collection localities and GPS co-ordinates for all specimens are available in the project “[CHSPI] All spiders of Churchill, Manitoba” through the Barcode of Life Data Systems (BOLD) (http://www.boldsystems.org) [24]. A list of specimens and key metadata are also provided in Additional file 1.

Diverse collecting methods were employed to maximize species recovery. Hand collecting was performed by sweep netting vegetation, by turning over stones and woody debris, and by searching lichen and moss substrates. Pan traps, Malaise traps, and Sticky traps (deployed in trees) yielded small numbers of specimens. Many specimens were collected in pitfall traps [25], made from white plastic containers (~10 cm diameter × 12 cm deep) that were placed along the marine shoreline and in fen, bog, tundra, and forested sites. 95% ethanol was added as a killing agent, and spiders were removed every two to four days. All specimens were then preserved in fresh 95% ethanol, and are now deposited at the Biodiversity Institute of Ontario, University of Guelph.

### Specimen selection and identification

The selection of specimens for molecular analysis employed two strategies. From 2005–2008, every specimen (1507) was barcoded and the adult spiders were subsequently identified morphologically by GAB. This phase of the work led to the recovery of sequences from 1013 specimens. Overall, 87 species were collected during this period, but this approach led to ‘oversampling’ of common species (e.g. 161 barcode records for *Pardosa lapponica*). After 2009, an effort was made to sequence no more than 10 specimens per species; so spiders were identified morphologically to the species level, when possible, before barcoding. As a rule, adult spiders in our dataset were identified to the species level based on morphology, but all representatives of certain small-bodied spider families (mainly Linyphiidae and Theridiidae) were barcoded because of the difficulty in species discrimination through morphology. Most juveniles and some females were assigned to a species based on their sequence similarity (<2%) to specimens of the taxon that were identified through morphological study [26-28]. Barcode clusters that were distinct from all others (>2% divergence), but that contained only juveniles, could not be identified morphologically and were thus assigned interim species codes and treated as separate species for analysis. Information on the life stage (A-adult; I-Immature) of each specimen is available through its record on BOLD. Standard taxonomic references were used for identification including: [17,19,20,29-66].

### Barcoding protocol

Whole specimens were arrayed in batches of 95 for databasing, photography, and tissue sampling, according to standard methods for high-throughput processing of specimens for DNA barcoding [67]. One leg was then removed from each specimen and placed into one of the wells in a 96-well plate. When a specimen was too small for leg removal, it was placed into the well, and the voucher was recovered after DNA extraction [68].

DNA barcoding was performed using standard, high-throughput methods at the Canadian Centre for DNA Barcoding [69-71]. DNA extraction employed a glass-fibre protocol [72], while polymerase chain reactions (PCR) were performed using standard PCR cocktails [70]. Primers were used to amplify the 658 bp barcode region of the cytochrome *c* oxidase subunit I (COI) gene, specifically the LepF1/LepR1 primers [73] or the LCO1490_t1/HCO2198_t1 Folmer primer pair [74], tailed with M13 [75]. The PCR thermal regime included the following steps: 94°C for a minute; 5 replicates of 94°C for a minute, 45°C for 40 seconds, and 72°C for one minute; 35 cycles of a minute at 94°C, 40 seconds at 51°C, and 72°C for a minute; and concluding with five minutes at 72°C. Primers used for PCR amplification as well as cycle sequencing for each specimen are available through BOLD. Sequences were assembled using CodonCode Aligner v. 3.0.2 (CodonCode Corporation), and sequences were examined for indels and stop codons as a check against pseudogenes.

### Analysis of genetic divergence

Analytical tools on BOLD were used to examine patterns of genetic divergence among the 2704 specimens with a sequence ≥500 bp. Nearest neighbour analysis (referred to as “barcode gap analysis” in BOLD3) plots the maximum pairwise divergence within a species against its minimum divergence to a different species. This plot indicates those cases where specimens can be reliably assigned to the correct species based on barcode analysis [76]. Although the use of Pairwise Distance (p-distance) has been advocated by some authors [77], Kimura-2-Parameter (K2P) [78] distances are similar unless nearest-neighbour distances are large (>12%) (Hebert, unpubl.). We employ K2P distances in our analysis partially for this reason, but also because this metric has been standard in prior barcoding studies. K2P and p-distances are reported as supplementary information (Additional file 2) to enable comparison of the values. A neighbour-joining (NJ) phenogram [79] employing the K2P distance model was constructed in MEGA5 [80], employing pairwise deletion of missing sites and with bootstraps based upon 500 replicates, which was subsequently ultrametricized in MEGA. This tree is presented to visualize genetic divergences, not as a phylogenetic hypothesis for these species.

### Biodiversity estimation

The completeness of sampling was visually assessed using the accumulation curve function on BOLD [24] for the 2704 specimens with a sequence ≥500 bp, considering both species and barcode clusters (Barcode Index Numbers – BINs [81]). This analysis resamples individuals with replacement, and we employed 100 iterations. The individual-based species richness estimator Chao1 [82] was also calculated using EstimateS Version 8.2 [83], with the default setting of 50 randomizations of input order. The composition of the fauna in terms of feeding guild was summarized by categorizing each species as an active predator, ambush predator, or web builder.

## Results and discussion

### Overview of the spiders of Churchill: diversity and distributions

COI sequences >500 bp were recovered from 77% of the specimens analyzed (2704/3506) (Additional file 1, Additional file 3). Among these records, 89% were fully compliant with the “barcode standard” as they possessed a sequence >500 bp with fewer than 1% Ns, and involved a record that was based on bidirectional sequence analysis. Sequencing success improved during the study, due largely to better preservation of specimens (e.g. more frequent ethanol exchange).

**Figure 2:**
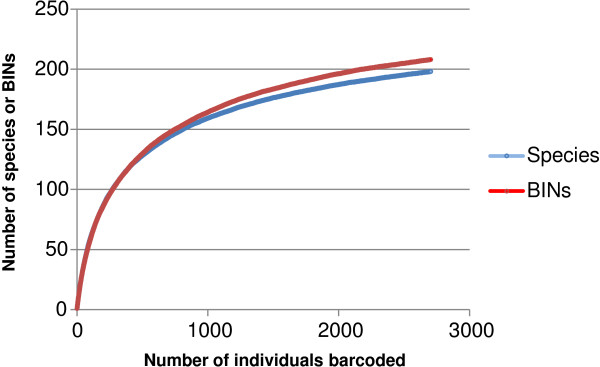
**Accumulation curves indicating the relationship between sample size and the number of species of spiders detected at Churchill.** The red line includes all sequenced specimens grouped by BIN (Barcode Index Numbers), and the blue line shows the number of spider species identified to a Linnean species name.

The joint morphological and DNA barcode analyses revealed 198 species representing 14 families and 98 genera (Table 1). This total includes 41% of the species of spiders known from Manitoba [1,19] and 14% of those recorded from Canada and Alaska [84]. Individual-based rarefaction curves based on both named species and BINs indicate that the fauna is well sampled (Figure 2). This conclusion is reinforced by the observation that just 34 species were represented by a single specimen, and 24 species by two individuals. Prior reports indicated the presence of 22 species in the Churchill region [1,15] that we did not collect, but some of these identifications are questionable. Interestingly, the Chao1 diversity estimator suggested that 220.4 spider species (95% confidence interval of 207.6-250.2) occur in the Churchill region. We conclude that most spider species in this region are now known, but that 10–20 taxa await detection.

**Table 1:** List of 198 species of spiders found in the Churchill region

	**Taxa**	**Distribution**	**N**
	**Amaurobiidae**		
*	*Cybaeopsis euopla* (Bishop & Crosby 1935)	NB	2
	**Araneidae**		
*	*Aculepeira carbonarioides* (Keyserling 1892)	HB	24
	*Araneus corticarius* (Emerton 1884)	NB	5
	*Araneus groenlandicola* (Strand 1906)	NS	18
*	*Araneus nordmanni* (Thorell 1870)	HB	2
*	*Araneus saevus* (L. Koch 1872)	HB	4
	*Hypsosinga pygmaea* (Sundevall 1831)	HB	13
	*Larinioides cornutus* (Clerck 1757)	HB	36
	*Larinioides patagiatus* (Clerck 1757)	HB	89
*	*Zygiella nearctica* Gertsch 1964	NB	34
	**Clubionidae**		
*	*Clubiona bryantae* Gertsch 1941	NB	2
*	*Clubiona furcata* Emerton 1919	HB	12
	*Clubiona norvegica* Strand 1900	HB	30
	*Clubiona praematura* Emerton 1909	HB	4
*	*Clubiona trivialis* C. L. Koch 1843	HB	39
	**Dictynidae**		
*	*Arctella lapponica* Holm 1945	HA	2
*	*Dictyna brevitarsa* Emerton 1915	NB	30
	*Dictyna major* Menge 1869	HB	33
	*Emblyna annulipes* (Blackwall 1846)	HB	18
*	*Emblyna manitoba* (Ivie 1947)	NB	5
*	*Emblyna peragrata* (Bishop & Ruderman 1946)	NB	5
*	*Hackmania prominula* (Tullgren 1948)	HB	1
	**Gnaphosidae**		
*	*Drassodes mirus* Platnick & Shadab 1976	HS	7
*	*Drassodes neglectus* (Keyserling 1887)	HS	6
*	*Gnaphosa borea* Kulczyn’ski 1908	HB	12
*	*Gnaphosa brumalis* Thorell 1875	NB	1
*	*Gnaphosa microps* Holm 1939	HB	11
*	*Gnaphosa muscorum* (L. Koch 1866)	HB	5
*	*Gnaphosa orites* Chamberlin 1922	HS	3
*	*Gnaphosa parvula* Banks 1896	NB	4
*	*Haplodrassus hiemalis* (Emerton 1909)	HB	6
*	*Haplodrassus signifer* (C. L. Koch 1839)	HS	6
*	*Micaria aenea* Thorell 1871	HB	3
*	*Micaria alpina* L. Koch 1872	HS	3
*	*Micaria constricta* Emerton 1894	HS	39
*	*Micaria pulicaria* (Sundevall 1831)	HB	8
	*Zelotes fratris* Chamberlin 1920	HB	1
*	*Zelotes sula* Lowrie & Gertsch 1955	HS	36
	**Hahniidae**		
*	*Hahnia cinerea* Emerton 1890	NB	19
	**Linyphiidae**		
	*Agyneta allosubtilis* Loksa 1965	HB	12
*	*Agyneta amersaxatilis* Saaristo & Koponen 1998	NB	1
*	*Agyneta fabra* (Keyserling 1886)	NB	6
*	*Agyneta jacksoni* Braendegaard 1937	NB	6
*	*Agyneta olivacea* (Emerton 1882)	HB	3
*	*Agyneta simplex* (Emerton 1926)	NB	1
*	*Allomengea dentisetis* (Grube 1861)	HB	9
	*Allomengea scopigera* (Grube 1859)	HA	10
**	*Baryphyma trifrons* (O. P.-Cambridge 1863)	HB	12
*	*Baryphyma trifrons affine* (Schenkel 1930)	HB	2
*	*Bathyphantes brevipes* (Emerton 1917)	NB	42
	*Bathyphantes brevis* (Emerton 1911)	NB	11
*	*Bathyphantes canadensis* (Emerton 1882)	HB	2
*	*Bathyphantes eumenis* (L. Koch 1879)	HS	1
*	*Bathyphantes gracilis* (Blackwall 1841)	HB	1
*	*Bathyphantes pallidus* (Banks 1892)	NB	3
*	*Bathyphantes reprobus* (Kulczyn’ski 1916)	HB	8
*	*Ceraticelus atriceps* (O. P.-Cambridge 1874)	NB	4
	*Ceraticelus crassiceps* Chamberlin & Ivie 1939	NB	8
*	*Ceratinella brunnea* Emerton 1882	NB	1
*	*Ceratinella ornatula* (Crosby & Bishop 1925)	NB	2
	*Cnephalocotes obscurus* (Blackwall 1834)	HB	8
*	*Diplocentria bidentata* (Emerton 1882)	HB	22
*	*Diplocentria rectangulata* (Emerton 1915)	HB	3
	*Dismodicus decemoculatus* (Emerton 1882)	NB	39
	*Entelecara* sp. 1GAB	-	2
*	*Erigone aletris* Crosby & Bishop 1928	HB	9
*	*Erigone arctica* (White 1852)	HB	10
*	*Erigone arctophylacis* Crosby & Bishop 1928	NB	4
	*Erigone cristatopalpus* Simon, 1884	NB	32
*	*Erigone dentigera* O. P.-Cambridge 1874	HB	3
*	*Erigone tirolensis* L. Koch 1872	HS	5
	*Estrandia grandaeva* (Keyserling 1886)	HB	85
*	*Floricomus rostratus* (Emerton 1882)	NB	1
*	*Gonatium crassipalpum* Bryant 1933	NB	4
*	*Grammonota angusta* Dondale 1959	NB	2
*	*Grammonota gentilis* Banks 1898	NB	48
*	*Grammonota maritima* Emerton 1925	NB	28
*	*Hilaira canaliculata* (Emerton 1915)	NB	2
*	*Horcotes quadricristatus* (Emerton 1882)	NS	4
*	*Hybauchenidium gibbosum* (Sørensen 1898)	NS	21
	*Hypomma marxi* (Keyserling 1886)	NB	16
*	*Hypselistes semiflavus* (L. Koch 1879)	HA	5
*	*Improphantes complicatus* (Emerton 1882)	HB	26
*	*Incestophantes washingtoni* (Zorsch 1937)	NS	13
*	*Islandiana falsifica* (Keyserling 1886)	HA	11
*	*Islandiana holmi* Ivie 1965	NB	42
	*Kaestneria pullata* (O. P.-Cambridge 1863)	HB	21
	*Kaestneria rufula* (Hackman 1954)	NB	6
	*Lepthyphantes alpinus* (Emerton 1882)	HB	90
	*Mecynargus paetulus* (O. P.-Cambridge 1875)	HA	23
*	*Metopobactrus prominulus* (O. P.-Cambridge 1872)	HB	1
	*Microlinyphia pusilla* (Sundevall 1830)	HS	2
	*Mughiphantes* sp. 1GAB	-	3
*	*Oedothorax trilobatus* (Banks 1896)	NB	5
*	*Oreoneta leviceps* (L. Koch 1879)	NA	1
*	*Oreonetides vaginatus* (Thorell 1872)	HB	9
*	*Pelecopsis mengei* (Simon 1884)	HB	3
*	*Phlattothrata parva* (Kulczyn’ski 1926)	HB	4
*	*Pityohyphantes cristatus* Chamberlin & Ivie 1942	NB	22
	*Pityohyphantes limitaneus* (Emerton 1915)	NB	46
	*Pityohyphantes subarcticus* Chamberlin & Ivie 1943	NS	60
*	*Pocadicnemis americana* Millidge 1976	NB	8
*	*Poeciloneta calcaratus* (Emerton 1909)	NB	1
*	*Poeciloneta variegata* (Blackwall 1841)	HB	1
*	*Praestigia kulczynskii* Eskov 1979	HB	2
*	*Satilatlas marxi* Keyserling 1886	NA	9
**	*Satilatlas monticola* Millidge 1981	NB	36
*	*Sciastes dubius* (Hackman 1954)	NB	2
	*Sciastes hastatus* Millidge 1984	NB	1
*	*Sciastes truncatus* (Emerton 1882)	NB	6
	*Scotinotylus alpinus* (Banks 1896)	HS	5
*	*Scotinotylus sacer* (Crosby 1929)	HB	4
	*Scotinotylus* sp. 1GAB	-	1
*	*Scylaceus pallidus* (Emerton 1882)	NB	6
	*Scyletria inflata* Bishop & Crosby 1938	NB	2
*	*Semljicola lapponicus* (Holm 1939)	HS	1
*	*Semljicola obtusus* (Emerton 1915)	HS	1
*	*Sisicottus montanus* (Emerton 1882)	NB	36
	*Sisis rotundus* (Emerton 1925)	NB	1
*	*Souessa spinifera* (O. P.-Cambridge 1874)	NB	12
*	*Styloctetor purpurescens* (Keyserling 1886)	NB	2
	*Tapinocyba bicarinata* (Emerton 1913)	NB	4
	*Tapinocyba minuta* (Emerton 1909)	NB	1
	*Tapinocyba* sp. 1GAB	-	1
*	*Tiso aestivus* (L. Koch 1872)	HS	8
*	*Tmeticus ornatus* (Emerton 1914)	NB	6
*	*Tunagyna debilis* (Banks 1892)	NB	6
	*Typhochrestus pygmaeus* (Sørensen 1898)	HA	1
*	*Wabasso cacuminatus* Millidge 1984	NB	7
*	*Wabasso quaestio* (Chamberlin 1949)	HS	4
*	*Walckenaeria castanea* (Emerton 1882)	NB	3
	*Walckenaeria communis* (Emerton 1882)	NB	11
*	*Walckenaeria exigua* (Millidge 1983)	NB	2
*	*Walckenaeria karpinskii* (O. P.-Cambridge 1873)	HB	4
*	*Walckenaeria kochi* (O. P.-Cambridge 1872)	HB	10
*	*Walckenaeria lepida* (Kulczyn’ski 1885)	HB	7
*	*Walckenaeria palustris* Millidge 1983	NB	6
*	*Walckenaeria spiralis* (Emerton 1882)	HB	2
*	*Zornella armata* (Banks 1906)	NB	2
	**Liocranidae**		
*	*Agroeca ornata* Banks 1892	NB	3
	**Lycosidae**		
*	*Alopecosa aculeata* (Clerck 1757)	HB	20
	*Alopecosa hirtipes* (Kulczyn’ski 1907)	NA	18
**	*Alopecosa* sp. 1GAB	NS	10
*	*Arctosa alpigena* (Doleschall 1852)	HB	1
	*Arctosa insignita* (Thorell 1872)	NA	52
*	*Arctosa raptor* (Kulczyn’ski 1885)	NS	9
*	*Pardosa dromaea* (Thorell 1878)	NB	19
	*Pardosa furcifera* (Thorell 1875)	NB	91
	*Pardosa fuscula* (Thorell 1875)	NB	26
*	*Pardosa glacialis* (Thorell 1872)	HA	4
	*Pardosa groenlandica* (Thorell 1872)	NS	9
	*Pardosa hyperborea* (Thorell 1872)	HB	46
	*Pardosa lapponica* (Thorell 1872)	HA	179
*	*Pardosa moesta* Banks 1892	NB	8
	*Pardosa podhorskii* (Kulczyn’ski 1907)	NA	2
	*Pardosa uintana* Gertsch 1933	NB	13
*	*Pirata bryantae* Kurata 1944	NB	1
*	*Pirata piraticus* (Clerck 1757)	HB	18
	*Piratula canadensis* (Dondale & Redner 1981)	HB	1
	**Philodromidae**		
	*Philodromus alascensis* Keyserling 1884	HB	17
*	*Philodromus histrio* (Latreille 1819)	HB	2
*	*Philodromus peninsulanus* Gertsch 1934	NB	7
*	*Thanatus formicinus* (Clerck 1757)	HB	5
	*Thanatus rubicellus* Mello-Leitão 1929	NB	22
	*Tibellus maritimus* (Menge 1875)	HB	12
	**Salticidae**		
*	*Chalcoscirtus glacialis* Caporiacco 1935	HA	1
*	*Pelegrina montana* (Emerton 1891)	NB	1
*	*Pellenes lapponicus* (Sundevall 1833)	NB	1
*	*Sitticus ammophilus* (Thorell 1875)	HB	1
*	*Sitticus finschi* (L. Koch 1879)	HB	1
	*Sitticus floricola palustris* (Peckham & Peckham 1883)	NB	9
*	*Sitticus ranieri* (Peckham & Peckham 1909)	HB	20
*	*Sitticus striatus* Emerton 1911	NB	2
	**Tetragnathidae**		
*	*Pachygnatha clercki* Sundevall 1823	HB	13
	*Tetragnatha extensa* (Linnaeus 1758)	HB	110
*	*Tetragnatha versicolor* Walckenaer 1841	NB	14
	**Theridiidae**		
*	*Enoplognatha intrepida* (Sørensen 1898)	NS	9
*	*Ohlertidion ohlerti* (Thorell 1870)	HB	15
*	*Phylloneta impressa* (L. Koch 1881)	HB	2
	*Robertus borealis* (Kaston 1946)	NB	1
*	*Robertus fuscus* (Emerton 1894)	NB	13
**	*Robertus lyrifer* Holm 1939	HS	2
*	*Theridion pictum* (Walckenaer 1802)	HB	10
*	*Thymoites oleatus* (L. Koch 1879)	HB	2
	**Thomisidae**		
*	*Coriarachne brunneipes* Banks 1893	NB	1
*	*Ozyptila arctica* Kulczyn’ski 1908	HS	20
*	*Ozyptila gertschi* Kurata 1944	HS	1
	*Xysticus britcheri* Gertsch 1934	HB	51
	*Xysticus canadensis* Gertsch 1934	HB	1
*	*Xysticus deichmanni* Sørensen 1898	NA	15
*	*Xysticus durus* (Sørensen 1898)	NS	53
*	*Xysticus ellipticus* Turnbull, Dondale & Redner 1965	NB	3
	*Xysticus labradorensis* Keyserling 1887	NS	31
*	*Xysticus luctuosus* (Blackwall 1836)	HB	5
*	*Xysticus nigromaculatus* Keyserling 1884	NB	4
*	*Xysticus obscurus* Collett 1877	HB	5
	*Xysticus triangulosus* Emerton 1894	NB	5
	*Xysticus triguttatus* Keyserling 1880	NB	1

Species newly reported for Canada are marked by two asterisks, while those new for Manitoba are marked with one asterisk. Taxonomy follows Platnick [11], while species distributions follow Benell-Aitchison and Dondale [20], Dondale et al. [39], and Platnick [11]. N indicates the number of barcode records for each species. Abbreviations in the Distribution column: NA - Nearctic, Arctic; NS - Nearctic, Subarctic; NB - Nearctic, Boreal; HA - Holarctic, Arctic; HS - Holarctic, Subarctic; HB - Holarctic, Boreal.

**Figure 3:**
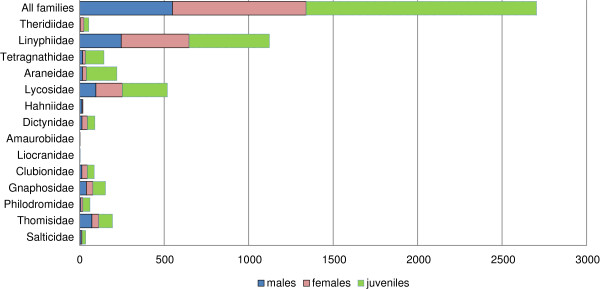
Number of adult and juvenile spiders for each family collected from the Churchill area.

Juveniles represented 50.4% of the specimens collected, but they varied in abundance from 0% in the Hahniidae to 82% in the Tetragnathidae (Figure 3). However, 98% of the barcode clusters could be identified to a species because they included some adult specimens. This analysis indicated that the Linyphiidae dominated the fauna with 100 species, 50.5% of the total (Table 1). Lycosidae were in second place with 19 species (9.6%), followed by Gnaphosidae (16 species; 8.1%) and Thomisidae (14 species; 7.1%). Another ten families were represented by fewer than 10 species each, jointly comprising 24.7% of the fauna: Araneidae (9 species), Theridiidae and Salticidae (8), Dictynidae (7), Philodromidae (6), Clubionidae (5), and Tetragnathidae (3). The remaining families (Amaurobiidae, Hahniidae, and Liocranidae) were each represented by a single species.

**Figure 4:**
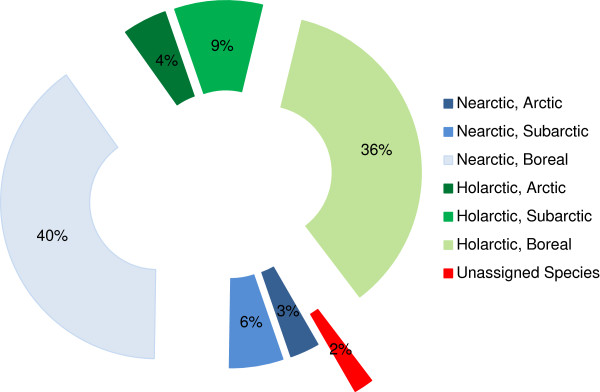
**Distribution patterns for spiders barcoded from Churchill.** Unassigned species refer to those collected as juveniles only; these lack a species-level identification and range information.

Half of the spider species (50.5%) at Churchill have a Holarctic distribution, while the remaining species are Nearctic (Figure 4). Table 1 lists the species detected and details their habitat preferences (arctic, sub-arctic, boreal) using assignments made by earlier authors [1,39]. Arctic species inhabit stony tundra, pebbly beaches, gravel bars, patches of lichens, and the litter beneath plant species typical of the arctic. Subarctic species are most common in stony habitats and in habitats with scattered trees such as *Populus* and *Salix*. Finally, boreal species are associated with conifers, aspens, and other plants typical of the boreal forest. Fifteen species (7.7%) are typical of the Arctic zone (Table 1, Figure 3), with linyphiids (8 species) and lycosids (5 species) dominating. Another 29 species (14.8%) are sub-Arctic with a dominance of linyphiids (41.4%) and gnaphosids (24.1%). The remaining species (150; 76.5%) at Churchill are typical of the boreal zone with Linyphiidae (51.7%) and Lycosidae (6.9%) dominating. Four other species (2%) lack ecological data because they could not be morphologically identified, as only juveniles were collected, and they did not closely match any other sequences on BOLD or GenBank (June, 2013).

The spider fauna at Churchill included species with varied feeding strategies; 128 (64.6%) are web builders, 37 (18.7%) are ambush predators, and 33 (16.7%) are active predators.

### Correspondence between morphological species and barcode clusters

**Figure 5:**
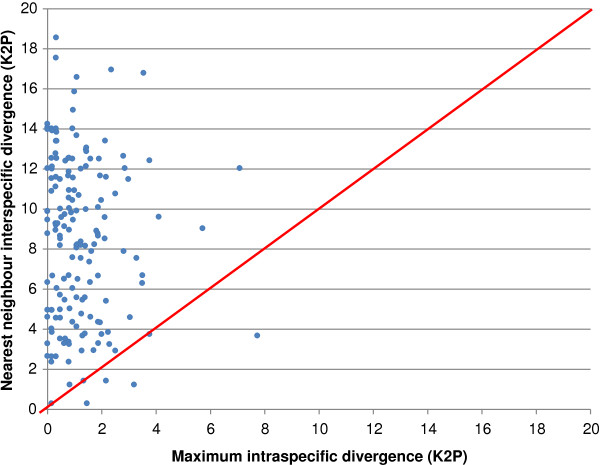
**Summary of sequence distances (K2P) at COI for spiders of Churchill.** Each species is represented by a point, with its maximum intraspecific distance plotted against its nearest neighbour (minimum interspecific) distance.

There was strong correspondence between the boundaries of barcode clusters and species designations based on morphology. Nearly all species (97%, 159/164) represented by two or more individuals displayed a barcode gap (Figure 5), reflecting the fact that the maximum intraspecific divergence was less than the distance to the nearest neighbour. As well, most of these species (94%, 158/168) showed more than 2% divergence from their nearest neighbour. The other 34 species (those represented by a single specimen) all showed more than 2% divergence from their nearest neighbour, and most (31/34) had >4% divergence. Even prior to taxonomic reassessments motivated by the barcode results, it is clear that DNA barcoding is a very effective tool for identification of spiders. Moreover, the close correspondence between BINs and species (Figure 2) indicates the value of DNA barcoding as a quick tool for the determination of species richness in unstudied araneofaunas.

### Taxonomic insights

The 198 species included representatives of 98 genera and 14 families (Table 1), including one species new to science and three new for Canada. One wolf spider (Lycosidae), belonging to the *Alopecosa pictilis* group [20,33], is probably undescribed and will be treated in a future publication (Blagoev and Dondale, unpubl.). *Robertus lyrifer* has a known Palaearctic distribution, and thus the “true” distribution could be Holarctic, which was previously overlooked, or this species may have been inadvertently introduced through ships visiting the port in Churchill. By contrast, *Satilatlas monticola* represents a range extension for a species previously only known from one locality in the USA [11,19]. Here we present the first description of the female of that species.

### New species for Canada

#### Family Theridiidae (cobweb weavers)

**Figure 6:**
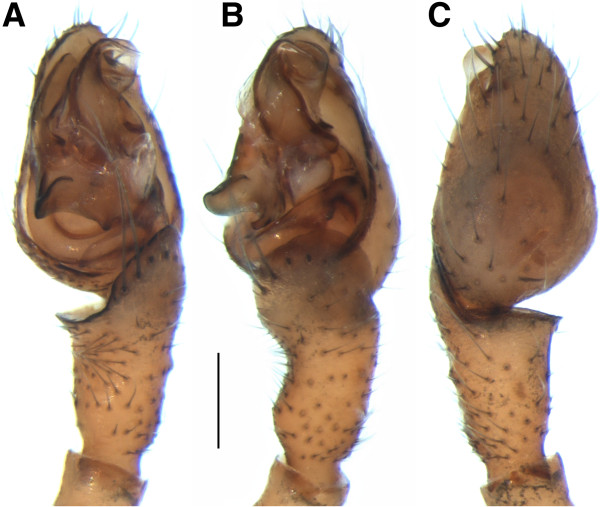
**Male of *Robertus lyrifer *(Theridiidae).** Left palp, **(A)** ventral, **(B)** prolateral, and **(C)** dorsal views. Scale bar: 0.2 mm.

**Figure 7:**
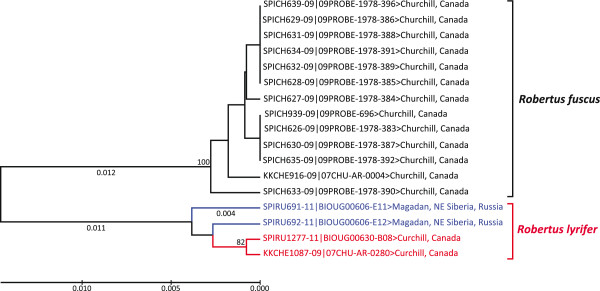
**Ultrametric neighbour-joining tree (based upon K2P distances) showing the relationships among 658 bp COI barcode sequences for *Robertus lyrifer*.** The red branches show sequences from Churchill, while sequences from other regions are in blue. Bootstrap values are based on 500 replications.

One of the new Canadian species (Figure 6), *Robertus lyrifer* Holm, has only previously been recorded from northern and central Europe [11,85]. However, the diagnostic feature for males of this species—the shape of the left palp—was identical in the specimen from Churchill and its counterparts from Europe [47,56]. As well, both specimens from Churchill showed close barcode similarity (0.5% divergence) to *R. lyrifer* from Russia (Figure 7).

#### Family Linyphiidae (dwarf and sheetweb weavers)

**Figure 8:**
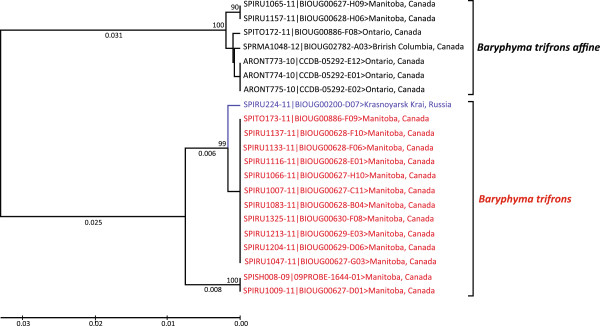
**Ultrametric neighbour-joining tree (K2P) showing the relationships among 658 bp COI barcode sequences for members of the *Baryphyma trifrons *complex.** The red branches show sequences from presumptive *Baryphyma trifrons* from Churchill, while a sequence from Russia is in blue. Bootstrap values are based on 500 replications.

**Figure 9:**
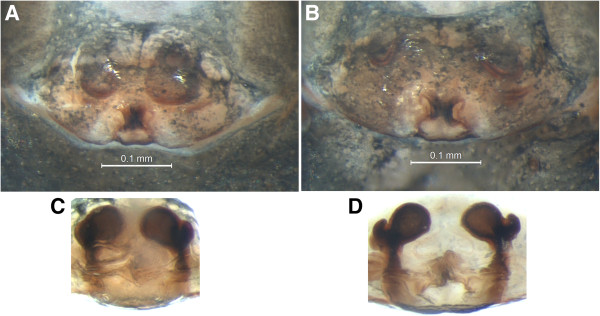
**Females of *Baryphyma *species. (A, C) ***B. trifrons affine* and **(B, D) ***B. trifrons*. **(A, B)** Epigyne ventral and **(C, D)** vulva dorsal views. Scale bar: 0.1 mm.

*Baryphyma trifrons* (O. P.-Cambridge), a Palearctic species which is very morphologically variable, currently includes ten synonyms [11]. Two monophyletic clusters of this species with a minimum divergence of 6.4% occur at Churchill (Figure 8). One resembles *B. trifrons affine* (Schenkel), which is no longer recognized as a valid subspecies [11,65], while the other resembles *B. trifrons* (O. P.-Cambridge). The sequences of *B. trifrons affine* from Churchill clustered with specimens from Ontario and British Columbia, while the second group clustered with a specimen from Russia. We conclude that the latter cluster represents *B. trifrons*. Although it is currently considered a synonym of *B. trifrons affine*[84], the barcode results challenge this conclusion, indicating the need for further taxonomic work (Figure 9).

*Satilatlas monticola* Millidge has until now been viewed as endemic to Elk Mountain, Colorado [19]. Originally described from a single male, our collections included 36 individuals (12 ♂♂, 22 ♀♀, and 2 juveniles) of this taxon, enabling the first description of its female morphology.

#### Family Linyphiidae Blackwall, 1859

##### Genus *Satilatlas* Keyserling, 1886

*Satilatlas monticola* Millidge, 1981.

*S. m.* Millidge, 1981: 252, f. 16, 30–31.

#### Material examined

Canada, Manitoba, Churchill - 4 ♂, 26 km SE Churchill, Twin Lakes, pt; lat. 58.6300, long. -93.7979; 29 m a.s.l.; 19-Jul-09; leg. D. Porco. - 1 ♂, 8 ♀, 16 km E Churchill, Bird Cove, pt; lat. 58.7639, long. -93.8970, 0 m; 20-Jul-09; leg. D. Porco. - 5 ♂, 4 ♀, 16 km E Churchill, Bird Cove, pt; lat. 58.7639, long. -93.8970, 0 m; 25-Jul-09; leg. G. Blagoev. - 1 ♂, 26 km SE Churchill, Twin Lakes, pt; lat. 58.6300, long. -93.8190, 34 m; 26-Jul-09; leg. D. Porco. - 1 ♂, 1 ♀, 26 km SE Churchill, Twin Lakes, pt; lat. 58.6300, long. -93.7979, 29 m; 27-Jul-09; leg. D. Porco. - 2 ♀, 16 km E Churchill, Bird Cove, pt; lat. 58.7639, long. -93.8970, 0 m; 27-Jul-09; leg. D. Porco. - 1 ♀, 26 km SE Churchill, Twin Lakes burn site, pt; lat. 58.6180, long. -93.8290, 53 m; 30-Jul-09; leg. G. Blagoev. - 1 ♂, Canada, 16 km E Churchill, Bird Cove, Rock Bluff A, grasses between ponds, close to pond 34; lat. 58.7718, long. -93.8439, 0 m; 08-Jul-10; leg. B. Laforest. - 3 ♀, 1 juvenile, 16 km E Churchill, Bird Cove, Rock Bluff A, ocean beach; lat. 58.7709, long. -93.8509, 7 m; 23-Jul-10; leg. V. Junea. - 2 ♀, 16 km E Churchill, Bird Cove, Rock Bluff A, ocean beach; lat. 58.7709, long. -93.8509, 7 m; 26-Jul-10; leg. V. Junea. - 1 ♀, 23 km E Churchill, Ramsay Creek, boreal forest; lat. 58.7304, long. -93.7805, 3 m; 30-Jul-10; leg. V. Junea. - 1 juvenile, 26 km SE Churchill, Twin Lakes fen, lat. 58.7666, long. -93.8529, 5 m; 02-Aug-10; leg. V. Junea.

The male of *Satilatlas monticola* was described by Millidge [19] from a specimen taken under stones at an elevation of more than 3600 m a.s.l. at East Maroon Pass, Elk Mountains, Pitkin and Gunnison Counties, Colorado, USA.

#### Diagnosis

**Figure 10:**
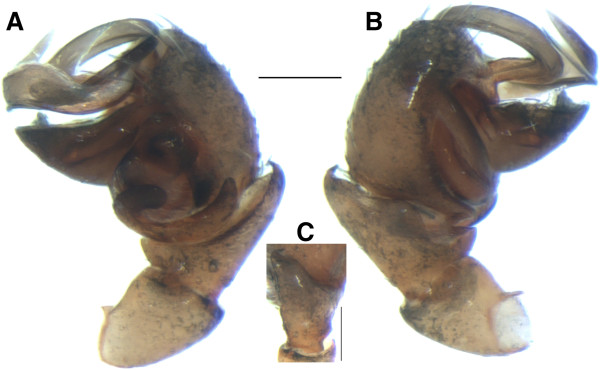
**Male of *Satilatlas monticola *.** Left palp, **(A)** retrolateral and **(B)** prolateral views, and **(C)** palpal tibia, dorsal view. Scale bar: 0.1 mm.

**Figure 11:**
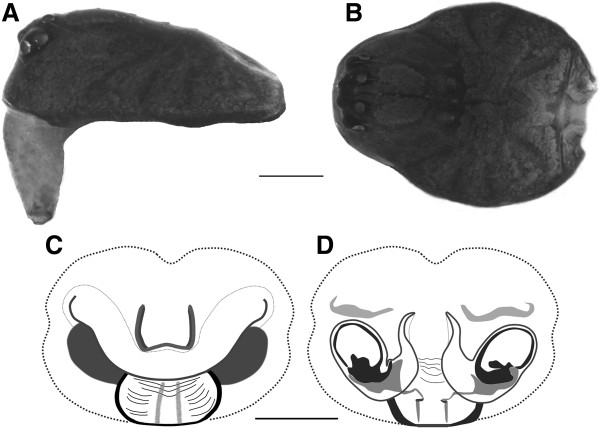
**Female of *Satilatlas monticola*.** Carapace, **(A)** lateral and **(B)** dorsal views. Epigyne, **(D)** ventral view and vulva **(E)** dorsal view. Scale bars: **(A, B)** 0.2 mm, and **(C, D)** 0.1 mm.

The structure of the palp in *Satilatlas monticola* suggests its close relationship with *Satilatlas gentilis*[19]. The male palp in specimens from Churchill clearly matched the illustrations in Millidge (Figure 10). The same resemblance was apparent in females of these two species as the shape of the epigyne in *S. monticola* is close to that in *S. gentilis*. However, the spermatheca in *S. monticola* is always inclined at an acute angle (Figure 11C, D), while that in *S. gentilis* is perpendicular to the central vertical axis of the epigyne. The epigyne of *S. monticola* also has a very broad, trapezium-shaped process which entirely covers the cavity with the openings to the sperm ducts.

#### Female

Total length: 1.8-2.3 mm. Carapace (Figure 11A, B) dark brownish: 0.80 × 0.62, nearly circular in dorsal view. Chaetotaxy: F, Pt I-IV, 0-0-0-0; Ti I-III, 3-0-0-0; Ti IV, 5-0-0-0; Mt I-III, 1-0-0-0; Mt IV, 0-0-0-0. Cephalic region is differentiated from the rest of the prosoma by darker bands. Sternum smooth monotonous with the same color. Legs yellowish-brown with darker transverse stripes in the bases of the limbs. Leg IV > leg I > leg II > leg III (Table 2).

**Table 2:** **Mean length in mm of leg segments of** ***Satilatlas monticola*** **female, based on a sample of five adult females**

**Legs**	**Trochanter**	**Femur**	**Patella**	**Tibia**	**Metatarsus**	**Tarsus**	**Total**
I	0.07	0.52	0.24	0.50	0.34	0.26	1.93
II	0.07	0.49	0.23	0.41	0.34	0.26	1.80
III	0.07	0.44	0.21	0.36	0.34	0.24	1.66
IV	0.07	0.61	0.23	0.59	0.44	0.28	2.22

#### Ecology

Specimens were found in wet areas near both Hudson Bay and inland ponds where it occurred among small stones and grass from mid-July to early August. Most specimens (27 adults) were collected in pitfall traps, but 7 adults and 2 juveniles were collected by hand.

##### Distribution

Previously only known from its type locality in Colorado, the present records extend its range to Churchill, suggesting this species can be expected to occur in alpine and low arctic habitats in western North America.

### Cryptic species

High “intraspecific” divergences (>2%) were found in 27 species and all these cases merit critical study as candidates for cryptic species (Additional file 3). However, some of these cases likely represent intraspecific variation as divergences greater than 2% have been reported in some arthropod species [6,7,86]. However, four Churchill species possessed >4% divergence and these taxa are discussed in more detail because they are the strongest candidates for cryptic species.

#### Family Araneidae (orbweavers)

**Figure 12:**
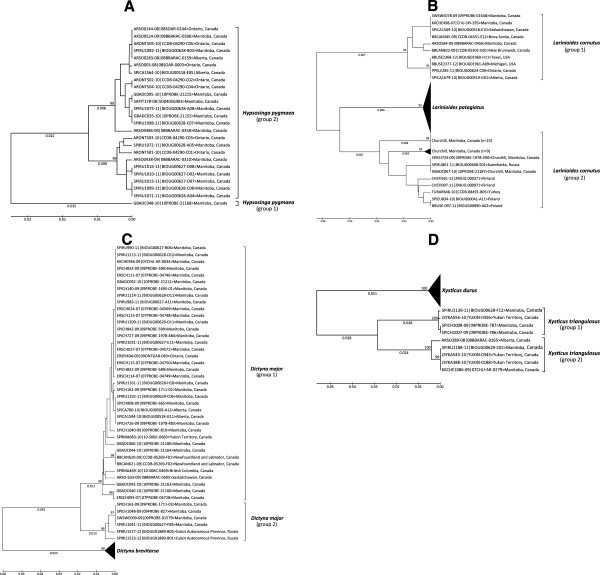
**Ultrametric neighbour-joining trees (K2P) showing deep sequence divergence at COI in four species: A)*Hypsosinga pygmaea*has two haplotype groups with 6.2% minimum divergence; B)*Larinioides cornutus*has two haplotype groups with 5.9% divergence and that are paraphyletic with respect to*L. patagiatus;*C)*Dictyna major*has two haplotype groups with 4.1% divergence; D)*Xysticus triangulosus*has two haplotype groups with 5.7% divergence.** Bootstrap values are based on 500 replications.

The Holarctic species, *Hypsosinga pygmaea* (Sundevall), includes two deeply divergent (6.2%) sequence clusters at Churchill (Figure 12A). One cluster was also collected in central Canada (Alberta, Manitoba, Ontario), but the sole representative of the other cluster matched specimens from Russia and Finland (not included in the paper). Although the two groups could not be separated morphologically, their sympatric occurrence in Churchill suggests their status as sibling species.

A second Holarctic species, *Larinioides cornutus* (Clerck), also includes two haplotype clusters at Churchill with a minimum divergence of 5.9%. Interestingly, the two clusters are paraphyletic with another member of this genus, *L. patagiatus* (Figure 12B). The first cluster includes specimens from across Canada and the northern USA, while the second cluster is closely similar to sequences from various European countries. In fact, an identical haplotype was detected in eastern Asia (Kamchatka, Russia).

#### Family Dictynidae (meshweavers)

*Dictyna major* Menge, a morphologically distinctive taxon, includes two sequence clusters with a minimum divergence of 3.5% (Figure 12C). One group has representatives from five provinces (Alberta, British Columbia, Manitoba, Newfoundland and Labrador, Saskatchewan) and from the Yukon Territory. By contrast, specimens in the second cluster group with members of this species from eastern Russia as well as several European countries (data not included in this paper). The four Churchill sequences in group 2 are identical, and this low genetic variation is suggestive of a recent introduction.

#### Family Thomisidae (crab spiders)

*Xysticus triangulosus* Emerton, a Nearctic species, includes two clusters at Churchill with a minimum distance of 5.5%. Both clusters were also collected in the Yukon (Figure 12D). As members of these clusters appear morphologically indistinguishable, future work should test for evidence of divergence at nuclear loci which would signal their status as distinct species.

### Species with low barcode divergence

**Figure 13:**
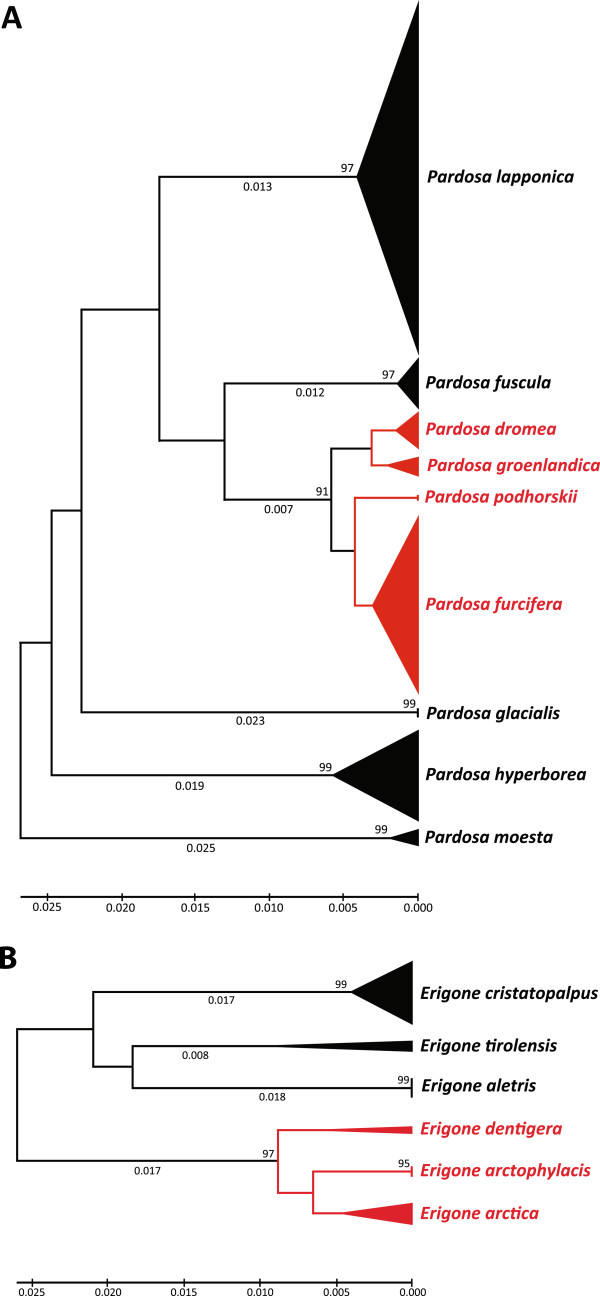
**Ultrametric neighbour-joining trees (K2P) showing shallow sequence divergence among COI sequences for two sibling species groups: A) four *Pardosa *species (*P. dromaea*, *P. groenlandica*, *P. podhorskii*, *P. furcifera*) of the *P. modica *group, and B) three *Erigone *species (*E. arctica*, *E. arctophylacis*, *E. dentigera*).** Bootstrap values are based on 500 replications.

Although different species usually show more than 2% interspecific divergence, lower levels of sequence divergence should occur in young species, and they have been detected in many groups [87,88]. In some extreme cases, valid species pairs differ by only a single bp in the barcode region [89] or not at all [73]. Two cases of young species assemblages were detected in this study involving species of *Pardosa* and *Erigone* (Figure 13).

Nine species of *Pardosa* were present at Churchill, most well separated by barcodes. However, four of these species (*Pardosa groenlandica*, *P. dromaea*, *P. furcifera*, *P. podhorskii*), belonging to the *Pardosa modica* group [20], showed limited divergence (Figure 13A). In particular, the minimum distance between *P. groenlandica* and *P. dromaea* was 0.8%, while that between *P. furcifera* and *P. podhorskii* was 1.2%. Although these are considered as “good species”, they can only be distinguished by genitalic characters. Their shallow genetic divergences suggest a recent origin, a fact which explains their limited morphological divergence. Our results, as well as the conclusions of other researchers ( [27], Dondale pers. comm.), suggest the *P. modica* group needs revision. A similar case was observed in *Erigone* (Figure 13B), where three species (*Erigone arctica*, *E. arctophylacis*, *E. dentigera*) possess a minimum inter-specific distance of 1.4%.

The fact that some species assemblages show low sequence divergences does not compromise the use of DNA barcoding for their identification [22,90]. Actually, all of the closely related species at Churchill formed distinct barcode groups. However, the presence of species such as this demonstrates the importance of the involvement of taxonomic specialists in the construction of DNA barcode reference libraries.

## Conclusions

This study has developed the first comprehensive DNA barcode reference library for the spider fauna of any region. The results indicate that DNA barcodes permit the discrimination of all species present at Churchill. Given the prevalence of juvenile spiders in most collections, DNA barcoding is a powerful tool for the identification of specimens, an important advance for future biomonitoring programs. Because the vast majority of barcode clusters correspond with a named species, the incidence of cryptic species appears to be low in northern spiders. The strong morphological/molecular correspondence indicates that prior morphological studies have been effective in species recognition in spiders, a situation which contrasts with that in several other groups at Churchill, especially parasitoid members of the order Hymenoptera [5,10]. This suggests that speciation in parasitoids, which tend to be host specific, is often associated only with biochemical evolution (e.g. in olfaction and immunity) rather than external morphological differentiation. This apparently contrasts with speciation in spiders, which is typically accompanied by genital and other morphological divergence. The present study did, however, detect four cases in which the prospect for cryptic species is high, and further studies on the other species showing high intraspecific divergence will likely extend the number of such cases.

Statistical analysis of the relationship between species discovery and sample size suggested that only about 20 species of spiders await detection at Churchill. However, because the present collections were made during the snow-free season, vernal species associated with snow edges were unlikely to be sampled. Because our work failed to detect 22 species reported in earlier work at Churchill [1,15], the Churchill fauna may include nearly 250 species. As with other arthropod groups, the spider fauna at Churchill includes a mix of Nearctic and Holarctic species. The small-bodied, web-building Linyphiidae was dominant (50.5% of species), followed by the active predators Lycosidae (9.6%), and two families of ambush predators, Gnaphosidae (8.1%) and Thomisidae (7.1%).

Our study has revealed a remarkable diversity of spider species in the Churchill region, increasing the fauna from 64 to 198 species. It also provides an important foundation for future biomonitoring, ecological studies, and taxonomic investigations.

## Abbreviations

CCDB: Canadian centre for DNA barcoding; BOLD: Barcode of Life Data Systems; CNSC: Churchill Northern Studies Centre; COI: Cytochrome *c* Oxidase subunit I; PCR: Polymerase chain reaction; E: East; SE: Southeast; pt: Pitfall traps; lat.: Latitude; long.: Longitude; a.s.l.: Above sea level; leg.: Collector.

## Competing interests

The authors declare that they have no competing interests.

## Authors’ contributions

GAB conducted field collecting, performed the morphological identifications, managed the BOLD projects, performed the analysis of the molecular data, and wrote the taxonomic insights. NIN participated in sequence analysis, including sequence editing, sequence alignment, and data validation. CNS performed statistical analysis and prepared some of the figures. SJA contributed to the conception and analyzed the sequence data. PDNH provided institutional support and led the grant applications funding the study. GAB, PDNH, and SJA designed and conducted the study. GAB, SJA, and PDNH wrote the manuscript. All authors read and approved the final manuscript.

## Supplementary Material

Additional file 1 **List of all barcoded spiders involved in Churchill study.** Legend: I – immature, A – adult. Click here for file

Additional file 2 Check-list of spiders with genetic divergence values included in the Churchill study. Click here for file

Additional file 3 **Ultrametricized neighbour-joining tree (K2P) for all 2704 COI sequences >500 bp from spiders collected at Churchill.** Red branches indicate the cryptic species, and blue colouring is used to highlight the new species records for Canada. Click here for file
