# Supplementary material for: Spiders (Araneae) of Churchill, Manitoba: DNA barcodes and morphology reveal high species diversity and new Canadian records
Source: BMC Ecol. 2013 Nov 26;13:44. doi: 10.1186/1472-6785-13-44 (PMC4222278; doi:10.1186/1472-6785-13-44)
Supplement: Additional file 1 — List of all barcoded spiders involved in Churchill study. Legend: I – immature, A – adult. [file 1472-6785-13-44-S1.docx]

| **Taxa** | **Life Stage** | **Sample ID** | **Process ID** | **GenBank ID** | **Latitude** | **Longitude** |
| --- | --- | --- | --- | --- | --- | --- |
| *Aculepeira carbonarioides* | I | 09PROBE-1978-493 | SPICH736-09 | GU684354 | 58.6300 | -94.2300 |
| *Aculepeira carbonarioides* | I | 10PROBE-21142 | GBADC022-10 | HQ956685 | 58.7340 | -94.1120 |
| *Aculepeira carbonarioides* | I | 10PROBE-21167 | GBADC047-10 | HQ956708 | 58.7340 | -94.1120 |
| *Aculepeira carbonarioides* | I | 10PROBE-21170 | GBADC050-10 | HQ956711 | 58.6340 | -93.7860 |
| *Aculepeira carbonarioides* | I | 10PROBE-21172 | GBADC052-10 | HQ956713 | 58.6340 | -93.7860 |
| *Aculepeira carbonarioides* | I | 10PROBE-21181 | GBADC061-10 | HQ956722 | 58.6340 | -93.7860 |
| *Aculepeira carbonarioides* | I | 10PROBE-21184 | GBADC064-10 | HQ956724 | 58.6610 | -93.8320 |
| *Aculepeira carbonarioides* | I | 10PROBE-21188 | GBADC068-10 | HQ956728 | 58.6610 | -93.8320 |
| *Aculepeira carbonarioides* | I | 10PROBE-21207 | GBADC087-10 | HQ956746 | 58.7180 | -94.1240 |
| *Aculepeira carbonarioides* | I | 10PROBE-21208 | GBADC088-10 | HQ956747 | 58.7180 | -94.1240 |
| *Aculepeira carbonarioides* | I | 10PROBE-21210 | GBADC090-10 | HQ956748 | 58.7180 | -94.1240 |
| *Aculepeira carbonarioides* | A | BIOUG00627-A09 | SPIRU981-11 | KF367807 | 58.6340 | -93.7860 |
| *Aculepeira carbonarioides* | A | BIOUG00627-A10 | SPIRU982-11 | KF367806 | 58.6340 | -93.7860 |
| *Aculepeira carbonarioides* | A | BIOUG00627-E06 | SPIRU1026-11 | KF367802 | 58.6329 | -93.7869 |
| *Aculepeira carbonarioides* | I | BIOUG00627-F11 | SPIRU1043-11 | KF367803 | 58.6329 | -93.7869 |
| *Aculepeira carbonarioides* | A | BIOUG00628-B10 | SPIRU1089-11 | KF367808 | 58.7340 | -94.1120 |
| *Aculepeira carbonarioides* | A | BIOUG00629-A01 | SPIRU1163-11 | KF367798 | 58.6217 | -93.8371 |
| *Aculepeira carbonarioides* | A | BIOUG00629-A02 | SPIRU1164-11 | KF367804 | 58.6217 | -93.8371 |
| *Aculepeira carbonarioides* | I | CHU-SPI-462 | KKCHE665-07 | KF367800 | 58.6200 | -93.8300 |
| *Aculepeira carbonarioides* | A | CHU-SPI-588 | KKCHE791-07 | KF367810 | 58.7470 | -94.1340 |
| *Aculepeira carbonarioides* | I | CHU-SPI-604 | KKCHE807-07 | KF367801 | 58.7470 | -94.1340 |
| *Aculepeira carbonarioides* | I | INV0218 | KKCHE058-06 | KF367809 | 58.6320 | -93.7860 |
| *Aculepeira carbonarioides* | I | INV0308 | KKCHE064-06 | KF367805 | 58.6320 | -93.7860 |
| *Aculepeira carbonarioides* | I | JBWM0005950 | KKCHE221-07 | KF367799 | 58.6170 | -93.8210 |
| *Agroeca ornata* | A | 09PROBE-1686-01 | SPICH122-09 | GU683786 | 58.7600 | -93.9540 |
| *Agroeca ornata* | A | 09PROBE-1773-01 | SPICH195-09 | GU683787 | 58.6260 | -94.2300 |
| *Agroeca ornata* | A | 09PROBE-734 | SPICH977-09 | GU684500 | 58.6300 | -93.8190 |
| *Agyneta allosubtilis* | I | 09PROBE-1752-01 | SPICH186-09 | GU684012 | 58.7310 | -93.7800 |
| *Agyneta allosubtilis* | A | 09PROBE-1835-01 | SPICH004-09 | GU683853 | 58.6300 | -93.8190 |
| *Agyneta allosubtilis* | A | 09PROBE-1842-01 | SPICH011-09 | GU683827 | 58.6300 | -93.8190 |
| *Agyneta allosubtilis* | A | 09PROBE-1849-01 | SPICH018-09 | GU683854 | 58.6300 | -93.8190 |
| *Agyneta allosubtilis* | I | 09PROBE-1854-01 | SPICH023-09 | GU683855 | 58.6300 | -93.8190 |
| *Agyneta allosubtilis* | I | 09PROBE-1978-151 | SPICH394-09 | GU684232 | 58.7310 | -93.7800 |
| *Agyneta allosubtilis* | I | 09PROBE-1978-152 | SPICH395-09 | GU684229 | 58.7310 | -93.7800 |
| *Agyneta allosubtilis* | I | 09PROBE-1978-153 | SPICH396-09 | GU684230 | 58.7310 | -93.7800 |
| *Agyneta allosubtilis* | A | 09PROBE-1978-154 | SPICH397-09 | GU684227 | 58.7310 | -93.7800 |
| *Agyneta allosubtilis* | A | 09PROBE-1978-155 | SPICH398-09 | GU684228 | 58.7310 | -93.7800 |
| *Agyneta allosubtilis* | A | 09PROBE-1978-157 | SPICH400-09 | GU684225 | 58.7310 | -93.7800 |
| *Agyneta allosubtilis* | A | 09PROBE-1978-227 | SPICH470-09 | GU684045 | 58.6300 | -93.7980 |
| *Agyneta fabra* | A | 09PROBE-1863-01 | SPICH032-09 | GU683848 | 58.6300 | -93.8190 |
| *Agyneta fabra* | A | 09PROBE-1978-124 | SPICH367-09 | GU684233 | 58.6690 | -94.1600 |
| *Agyneta fabra* | A | 09PROBE-1978-189 | SPICH432-09 | GU684234 | 58.6260 | -94.2300 |
| *Agyneta fabra* | A | 09PROBE-1978-19 | SPICH262-09 | GU684296 | 58.6690 | -94.1600 |
| *Agyneta fabra* | A | 09PROBE-1978-244 | SPICH487-09 | GU684089 | 58.6630 | -94.1670 |
| *Agyneta fabra* | A | BIOUG00632-B12 | SPIRU1376-11 | KF368264 | 58.7710 | -93.8510 |
| *Agyneta jacksoni* | A | 09PROBE-1978-403 | SPICH646-09 | GU684531 | 58.7640 | -93.8970 |
| *Agyneta jacksoni* | A | 09PROBE-1978-502 | SPICH745-09 | GU684372 | 58.7890 | -93.7090 |
| *Agyneta jacksoni* | A | 09PROBE-613 | SPICH856-09 | GU684444 | 58.7900 | -94.2270 |
| *Agyneta jacksoni* | I | 09PROBE-639 | SPICH882-09 | GU684445 | 58.7900 | -94.2270 |
| *Agyneta jacksoni* | A | BIOUG00630-A08 | SPIRU1265-11 | KF368265 | 58.6610 | -93.8320 |
| *Agyneta jacksoni* | A | BIOUG00632-C04 | SPIRU1380-11 | KF368266 | 58.7710 | -93.8510 |
| *Agyneta olivacea* | A | 09PROBE-1833-01 | SPICH002-09 | GU683856 | 58.6300 | -93.8190 |
| *Agyneta olivacea* | A | 09PROBE-1858-01 | SPICH027-09 | GU683857 | 58.6300 | -93.8190 |
| *Agyneta olivacea* | A | 09PROBE-1978-161 | SPICH404-09 | GU684231 | 58.6180 | -93.8290 |
| *Agyneta simplex* | I | 09PROBE-1824-01 | SPICH236-09 | GU683974 | 58.6300 | -93.8190 |
| *Allomengea dentisetis* | I | 07CHU-AR-0059 | KKCHE971-09 | GU683668 | 58.7620 | -94.1780 |
| *Allomengea dentisetis* | I | 07CHU-AR-0061 | KKCHE973-09 | GU683672 | 58.7620 | -94.1780 |
| *Allomengea dentisetis* | I | 07CHU-AR-0062 | KKCHE974-09 | GU683671 | 58.7690 | -94.1600 |
| *Allomengea dentisetis* | A | 07CHU-AR-0063 | KKCHE975-09 | GU683676 | 58.7690 | -94.1600 |
| *Allomengea dentisetis* | A | 07CHU-AR-0064 | KKCHE976-09 | GU683675 | 58.7690 | -94.1600 |
| *Allomengea dentisetis* | A | 07CHU-AR-0066 | KKCHE978-09 | GU683677 | 58.7620 | -94.1780 |
| *Allomengea dentisetis* | I | 07CHU-AR-0067 | KKCHE979-09 | HM862437 | 58.7680 | -94.1560 |
| *Allomengea dentisetis* | A | 09PROBE-1978-344 | SPICH587-09 | GU684160 | 58.6260 | -94.2300 |
| *Allomengea dentisetis* | A | 09PROBE-754 | SPICH997-09 | GU684469 | 58.7880 | -94.2280 |
| *Allomengea scopigera* | A | 09PROBE-1978-113 | SPICH356-09 | GU684211 | 58.6690 | -94.1600 |
| *Allomengea scopigera* | A | 09PROBE-1978-118 | SPICH361-09 | GU684210 | 58.6690 | -94.1600 |
| *Allomengea scopigera* | I | 09PROBE-1978-128 | SPICH371-09 | GU684207 | 58.6690 | -94.1600 |
| *Allomengea scopigera* | I | 09PROBE-1978-130 | SPICH373-09 | GU684208 | 58.6690 | -94.1600 |
| *Allomengea scopigera* | A | 09PROBE-1978-272 | SPICH515-09 | GU684095 | 58.6630 | -94.1670 |
| *Allomengea scopigera* | A | 09PROBE-1978-83 | SPICH326-09 | GU684288 | 58.6690 | -94.1600 |
| *Allomengea scopigera* | I | 09PROBE-1978-96 | SPICH339-09 | GU684212 | 58.6690 | -94.1600 |
| *Allomengea scopigera* | I | 09PROBE-1978-97 | SPICH340-09 | GU684209 | 58.6690 | -94.1600 |
| *Allomengea scopigera* | I | HLC-26865 | KKCHE896-09 | KF367812 | 58.7760 | -94.1880 |
| *Allomengea scopigera* | I | HLC-26866 | KKCHE897-09 | KF367811 | 58.7760 | -94.1880 |
| *Alopecosa aculeata* | I | 07CHU-AR-0242 | KKCHE1049-09 | GU683726 | 58.7600 | -93.9620 |
| *Alopecosa aculeata* | I | 07CHU-AR-0292 | KKCHE1099-09 | GU683603 | 58.7600 | -93.9620 |
| *Alopecosa aculeata* | I | 07PROBE-02808 | TWSC194-08 | KF367822 | 58.6300 | -93.8190 |
| *Alopecosa aculeata* | I | 07PROBE-02821 | TWSC207-08 | KF367821 | 58.7310 | -93.7800 |
| *Alopecosa aculeata* | I | 07PROBE-02856 | TWSC242-08 | KF367818 | 58.7300 | -93.7840 |
| *Alopecosa aculeata* | I | 07PROBE-06539 | TWSC053-07 | KF367823 | 58.6300 | -93.7750 |
| *Alopecosa aculeata* | I | 07PROBE-06540 | TWSC054-07 | KF367814 | 58.6300 | -93.7750 |
| *Alopecosa aculeata* | I | 07PROBE-06546 | TWSC060-07 | KF367816 | 58.6170 | -93.8140 |
| *Alopecosa aculeata* | I | 07PROBE-06550 | TWSC064-07 | KF367825 | 58.7310 | -93.7800 |
| *Alopecosa aculeata* | I | 07PROBE-06554 | TWSC068-07 | KF367828 | 58.6300 | -93.7750 |
| *Alopecosa aculeata* | I | 07PROBE-06555 | TWSC069-07 | KF367819 | 58.6300 | -93.7750 |
| *Alopecosa aculeata* | I | 09-PROBE-08104 | JDTGS008-09 | GU679906 | 58.6250 | -93.8190 |
| *Alopecosa aculeata* | I | 09-PROBE-08122 | JDTGS026-09 | GU679894 | 58.6180 | -93.8290 |
| *Alopecosa aculeata* | A | BIOUG00628-B01 | SPIRU1080-11 | KF367827 | 58.7544 | -93.9980 |
| *Alopecosa aculeata* | A | BIOUG00633-B04 | SPIRU1404-11 | KF367813 | 58.7720 | -93.8431 |
| *Alopecosa aculeata* | I | CHU-SPI-370 | KKCHE557-07 | KF367820 | 58.7540 | -93.9490 |
| *Alopecosa aculeata* | I | CHU-SPI-388 | KKCHE591-07 | KF367826 | 58.6320 | -93.7860 |
| *Alopecosa aculeata* | I | HLC13410 | KKCHE053-06 | KF367815 | 58.7680 | -93.8680 |
| *Alopecosa aculeata* | I | HLC-26848 | KKCHE879-09 | KF367824 | 58.6200 | -93.7800 |
| *Alopecosa aculeata* | I | HLC-26854 | KKCHE885-09 | KF367817 | 58.6200 | -93.7800 |
| *Alopecosa hirtipes* | A | 07CHU-AR-0208 | KKCHE1015-09 | GU683702 | 58.6530 | -93.1910 |
| *Alopecosa hirtipes* | I | 07CHU-AR-0209 | KKCHE1016-09 | GU683707 | 58.6530 | -93.1910 |
| *Alopecosa hirtipes* | I | 07CHU-AR-0210 | KKCHE1017-09 | GU683706 | 58.6630 | -93.1910 |
| *Alopecosa hirtipes* | I | 07CHU-AR-0213 | KKCHE1020-09 | GU683705 | 58.6620 | -93.1890 |
| *Alopecosa hirtipes* | I | 07CHU-AR-0214 | KKCHE1021-09 | GU683704 | 58.6620 | -93.1890 |
| *Alopecosa hirtipes* | I | 07CHU-AR-0215 | KKCHE1022-09 | GU683709 | 58.6620 | -93.1890 |
| *Alopecosa hirtipes* | I | 07CHU-AR-0216 | KKCHE1023-09 | GU683708 | 58.6510 | -93.1950 |
| *Alopecosa hirtipes* | I | 07CHU-AR-0225 | KKCHE1032-09 | GU683716 | 58.1280 | -92.8560 |
| *Alopecosa hirtipes* | A | 07CHU-AR-0259 | KKCHE1066-09 | GU683742 | 58.6620 | -93.1890 |
| *Alopecosa hirtipes* | A | 09PROBE-1621-01 | SPISH004-09 | GU683934 | 58.6300 | -93.7980 |
| *Alopecosa hirtipes* | A | 09PROBE-1621-02 | SPISH005-09 | GU683930 | 58.6300 | -93.7980 |
| *Alopecosa hirtipes* | I | 09PROBE-1694-01 | SPICH137-09 | GU683931 | 58.7310 | -93.7800 |
| *Alopecosa hirtipes* | I | 09PROBE-1694-02 | SPICH138-09 | GU683932 | 58.7310 | -93.7800 |
| *Alopecosa hirtipes* | I | 09PROBE-1694-03 | SPICH139-09 | GU683933 | 58.7310 | -93.7800 |
| *Alopecosa hirtipes* | A | 09PROBE-1890-01 | SPICH055-09 | GU683898 | 58.7700 | -93.8430 |
| *Alopecosa hirtipes* | A | 09PROBE-1891-01 | SPICH056-09 | GU683897 | 58.7700 | -93.8430 |
| *Alopecosa hirtipes* | A | 09PROBE-1892-01 | SPICH057-09 | GU683899 | 58.7700 | -93.8430 |
| *Alopecosa hirtipes* | I | BIOUG00633-A10 | SPIRU1398-11 | KF367829 | 58.7285 | -93.7909 |
| *Alopecosa* sp. 1GAB | I | 07CHU-AR-0201 | KKCHE1008-09 | GU683697 | 58.7600 | -94.0860 |
| *Alopecosa* sp. 1GAB | A | 07CHU-AR-0202 | KKCHE1009-09 | GU683696 | 58.7600 | -94.0860 |
| *Alopecosa* sp. 1GAB | A | 07CHU-AR-0203 | KKCHE1010-09 | GU683695 | 58.7600 | -94.0860 |
| *Alopecosa* sp. 1GAB | A | 07CHU-AR-0204 | KKCHE1011-09 | GU683701 | 58.7600 | -94.0860 |
| *Alopecosa* sp. 1GAB | A | 07CHU-AR-0205 | KKCHE1012-09 | GU683700 | 58.7600 | -94.0860 |
| *Alopecosa* sp. 1GAB | I | 07CHU-AR-0206 | KKCHE1013-09 | GU683699 | 58.7600 | -94.0860 |
| *Alopecosa* sp. 1GAB | I | 07PROBE-02739 | TWSC126-08 | KF367832 | 58.7600 | -94.0860 |
| *Alopecosa* sp. 1GAB | I | 07PROBE-02770 | TWSC157-08 | KF367833 | 58.7600 | -94.0860 |
| *Alopecosa* sp. 1GAB | I | 07PROBE-02883 | TWSC269-08 | KF367831 | 58.7600 | -94.0860 |
| *Alopecosa* sp. 1GAB | I | 07PROBE-06502 | TWSC016-07 | KF367830 | 58.7640 | -94.0870 |
| *Araneus corticarius* | A | BIOUG00627-B05 | SPIRU989-11 | KF367838 | 58.7306 | -93.7804 |
| *Araneus corticarius* | I | BIOUG00627-E10 | SPIRU1030-11 | KF367834 | 58.6192 | -93.8291 |
| *Araneus corticarius* | A | BIOUG00628-B09 | SPIRU1088-11 | KF367836 | 58.7340 | -94.1120 |
| *Araneus corticarius* | A | BIOUG00629-A04 | SPIRU1166-11 | KF367837 | 58.7306 | -93.7804 |
| *Araneus corticarius* | A | BIOUG00630-E01 | SPIRU1306-11 | KF367835 | 58.7305 | -93.7805 |
| *Araneus groenlandicola* | I | 09PROBE-01647 | SWSWE077-09 | GU681023 | 58.6600 | -93.8300 |
| *Araneus groenlandicola* | I | 09PROBE-1880-01 | SPICH046-09 | GU683834 | 58.7500 | -94.0840 |
| *Araneus groenlandicola* | A | 09PROBE-1978-489 | SPICH732-09 | GU684353 | 58.6300 | -94.2300 |
| *Araneus groenlandicola* | I | 10PROBE-21165 | GBADC045-10 | HQ956706 | 58.7340 | -94.1120 |
| *Araneus groenlandicola* | I | 10PROBE-21169 | GBADC049-10 | HQ956710 | 58.7340 | -94.1120 |
| *Araneus groenlandicola* | I | 10PROBE-21200 | GBADC080-10 | HQ956740 | 58.6610 | -93.8320 |
| *Araneus groenlandicola* | I | 10PROBE-21204 | GBADC084-10 | HQ956743 | 58.7180 | -94.1240 |
| *Araneus groenlandicola* | I | 10PROBE-21206 | GBADC086-10 | HQ956745 | 58.7180 | -94.1240 |
| *Araneus groenlandicola* | I | 10PROBE-21214 | GBADC094-10 | KF367848 | 58.7180 | -94.1240 |
| *Araneus groenlandicola* | I | BIOUG00627-G04 | SPIRU1048-11 | KF367844 | 58.7614 | -94.0115 |
| *Araneus groenlandicola* | I | BIOUG00627-G05 | SPIRU1049-11 | KF367847 | 58.7614 | -94.0115 |
| *Araneus groenlandicola* | I | CHU-SPI-109 | KKCHE312-07 | KF367842 | 58.6170 | -93.8210 |
| *Araneus groenlandicola* | I | CHU-SPI-476 | KKCHE679-07 | KF367846 | 58.7470 | -94.1340 |
| *Araneus groenlandicola* | I | CHU-SPI-496 | KKCHE699-07 | KF367843 | 58.7470 | -94.1340 |
| *Araneus groenlandicola* | I | INV0068 | KKCHE057-06 | KF367840 | 58.6320 | -93.7860 |
| *Araneus groenlandicola* | I | INV0127 | KKCHE063-06 | KF367845 | 58.6320 | -93.7860 |
| *Araneus groenlandicola* | I | INV0300 | KKCHE095-06 | KF367841 | 58.6320 | -93.7860 |
| *Araneus groenlandicola* | A | JBWM0005937 | KKCHE216-07 | KF367839 | 58.7550 | -93.9980 |
| *Araneus nordmanni* | A | 09PROBE-942 | SPICH1163-09 | GU684587 | 58.6300 | -93.7980 |
| *Araneus nordmanni* | I | CHU-SPI-072 | KKCHE181-06 | KF367849 | 58.6220 | -93.8100 |
| *Araneus saevus* | I | 09PROBE-01621 | SWSWE051-09 | GU681017 | 58.6190 | -93.8290 |
| *Araneus saevus* | I | 09-PROBE-08112 | JDTGS016-09 | GU679828 | 58.6180 | -93.8290 |
| *Araneus saevus* | I | CHU-SPI-004 | KKCHE113-06 | KF367851 | 58.6220 | -93.8100 |
| *Araneus saevus* | I | CHU-SPI-015 | KKCHE124-06 | KF367850 | 58.6180 | -93.8230 |
| *Arctella lapponica* | I | 07CHU-AR-0025 | KKCHE937-09 | GU683641 | 58.6260 | -94.2300 |
| *Arctella lapponica* | I | 07CHU-AR-0237 | KKCHE1044-09 | GU683728 | 58.7500 | -93.9100 |
| *Arctosa alpigena* | I | CHU-SPI-482 | KKCHE685-07 | KF367852 | 58.7380 | -93.8190 |
| *Arctosa insignita* | I | 07CHU-AR-0282 | KKCHE1089-09 | GU683599 | 58.7570 | -93.9840 |
| *Arctosa insignita* | I | 07PROBE-02749 | TWSC136-08 | KF367862 | 58.7050 | -94.0520 |
| *Arctosa insignita* | I | 07PROBE-02797 | TWSC183-08 | KF367868 | 58.7050 | -94.0520 |
| *Arctosa insignita* | I | 07PROBE-02798 | TWSC184-08 | KF367897 | 58.7380 | -93.8190 |
| *Arctosa insignita* | I | 07PROBE-02799 | TWSC185-08 | KF367898 | 58.7380 | -93.8190 |
| *Arctosa insignita* | I | 07PROBE-02800 | TWSC186-08 | KF367864 | 58.7050 | -94.0520 |
| *Arctosa insignita* | I | 07PROBE-02803 | TWSC189-08 | KF367900 | 58.7050 | -94.0520 |
| *Arctosa insignita* | I | 07PROBE-02806.b | TWSC282-09 | KF367853 | 58.7570 | -93.9840 |
| *Arctosa insignita* | I | 07PROBE-02809.b | TWSC281-09 | KF367895 | 58.7050 | -94.0520 |
| *Arctosa insignita* | I | 07PROBE-02813.b | TWSC279-09 | KF367878 | 58.7050 | -94.0520 |
| *Arctosa insignita* | I | 07PROBE-02819 | TWSC205-08 | KF367859 | 58.7300 | -93.7800 |
| *Arctosa insignita* | I | 07PROBE-02823.b | TWSC280-09 | KF367874 | 58.7310 | -93.7810 |
| *Arctosa insignita* | I | 07PROBE-02848 | TWSC234-08 | KF367867 | 58.7300 | -93.7840 |
| *Arctosa insignita* | I | 07PROBE-02857 | TWSC243-08 | KF367886 | 58.7300 | -93.7840 |
| *Arctosa insignita* | I | 07PROBE-02859 | TWSC245-08 | KF367882 | 58.7300 | -93.7840 |
| *Arctosa insignita* | I | 07PROBE-02865 | TWSC251-08 | KF367860 | 58.7770 | -93.7360 |
| *Arctosa insignita* | I | 07PROBE-02882 | TWSC268-08 | KF367889 | 58.7300 | -93.7840 |
| *Arctosa insignita* | I | 07PROBE-06501 | TWSC015-07 | KF367885 | 58.7050 | -94.0520 |
| *Arctosa insignita* | I | 07PROBE-06548 | TWSC062-07 | KF367901 | 58.7570 | -93.9840 |
| *Arctosa insignita* | I | 07PROBE-06549 | TWSC063-07 | KF367871 | 58.7570 | -93.9840 |
| *Arctosa insignita* | I | 07PROBE-06551 | TWSC065-07 | KF367891 | 58.7050 | -94.0520 |
| *Arctosa insignita* | I | 07PROBE-06552 | TWSC066-07 | KF367858 | 58.7050 | -94.0520 |
| *Arctosa insignita* | I | 07PROBE-06553 | TWSC067-07 | KF367894 | 58.7050 | -94.0520 |
| *Arctosa insignita* | I | 07PROBE-06556 | TWSC070-07 | KF367887 | 58.7050 | -94.0520 |
| *Arctosa insignita* | I | 07PROBE-06557 | TWSC071-07 | KF367870 | 58.7370 | -93.8230 |
| *Arctosa insignita* | A | 09PROBE-736 | SPICH979-09 | GU684502 | 58.7310 | -93.7800 |
| *Arctosa insignita* | A | BIOUG00629-G06 | SPIRU1240-11 | KF367876 | 58.7720 | -93.8431 |
| *Arctosa insignita* | A | BIOUG00630-B12 | SPIRU1281-11 | KF367865 | 58.7710 | -93.8510 |
| *Arctosa insignita* | A | BIOUG00630-C12 | SPIRU1293-11 | KF367863 | 58.7770 | -94.1880 |
| *Arctosa insignita* | A | BIOUG00630-D09 | SPIRU1302-11 | KF367879 | 58.7707 | -94.1808 |
| *Arctosa insignita* | A | BIOUG00630-D10 | SPIRU1303-11 | KF367855 | 58.7305 | -93.7805 |
| *Arctosa insignita* | A | BIOUG00630-D11 | SPIRU1304-11 | KF367893 | 58.7305 | -93.7805 |
| *Arctosa insignita* | A | BIOUG00630-D12 | SPIRU1305-11 | KF367888 | 58.7305 | -93.7805 |
| *Arctosa insignita* | I | BIOUG00633-A09 | SPIRU1397-11 | KF367877 | 58.7544 | -93.9980 |
| *Arctosa insignita* | I | CHU-SPI-181 | KKCHE384-07 | KF367866 | 58.6170 | -93.8210 |
| *Arctosa insignita* | I | CHU-SPI-422 | KKCHE625-07 | KF367883 | 58.7380 | -93.8190 |
| *Arctosa insignita* | I | CHU-SPI-436 | KKCHE639-07 | KF367861 | 58.7380 | -93.8190 |
| *Arctosa insignita* | I | CHU-SPI-446 | KKCHE649-07 | KF367875 | 58.7380 | -93.8190 |
| *Arctosa insignita* | I | CHU-SPI-492 | KKCHE695-07 | KF367899 | 58.7470 | -94.1340 |
| *Arctosa insignita* | I | CHU-SPI-494 | KKCHE697-07 | KF367869 | 58.7470 | -94.1340 |
| *Arctosa insignita* | I | CHU-SPI-511 | KKCHE714-07 | KF367872 | 58.7380 | -93.8190 |
| *Arctosa insignita* | I | CHU-SPI-522 | KKCHE725-07 | KF367880 | 58.7380 | -93.8190 |
| *Arctosa insignita* | I | CHU-SPI-523 | KKCHE726-07 | KF367884 | 58.7380 | -93.8190 |
| *Arctosa insignita* | A | CHU-SPI-550 | KKCHE753-07 | KF367854 | 58.7470 | -94.1340 |
| *Arctosa insignita* | A | CHU-SPI-558 | KKCHE761-07 | KF367896 | 58.7380 | -93.8190 |
| *Arctosa insignita* | I | CHU-SPI-562 | KKCHE765-07 | KF367902 | 58.7470 | -94.1340 |
| *Arctosa insignita* | A | CHU-SPI-576 | KKCHE779-07 | KF367890 | 58.7470 | -94.1340 |
| *Arctosa insignita* | I | CHU-SPI-605 | KKCHE808-07 | KF367873 | 58.7400 | -93.8200 |
| *Arctosa insignita* | I | CHU-SPI-611 | KKCHE814-07 | KF367856 | 58.7400 | -93.8200 |
| *Arctosa insignita* | I | JBWM0006097 | KKCHE267-07 | KF367881 | 58.7380 | -93.8190 |
| *Arctosa insignita* | I | JBWM0234316f | KKCHE298-07 | KF367892 | 58.1170 | -92.8500 |
| *Arctosa insignita* | I | JBWM0234316m | KKCHE297-07 | KF367857 | 58.1170 | -92.8500 |
| *Arctosa raptor* | I | 07CHU-AR-0255 | KKCHE1062-09 | GU683741 | 58.7600 | -93.9620 |
| *Arctosa raptor* | A | 07PROBE-06498 | TWSC012-07 | KF367905 | 58.7370 | -93.8230 |
| *Arctosa raptor* | A | 07PROBE-06499 | TWSC013-07 | KF367903 | 58.7310 | -93.8150 |
| *Arctosa raptor* | I | 09-PROBE-08103 | JDTGS007-09 | GU679905 | 58.6250 | -93.8190 |
| *Arctosa raptor* | A | 09PROBE-1678-02 | SPICH105-09 | GU683912 | 58.7610 | -93.9540 |
| *Arctosa raptor* | A | 09PROBE-1678-03 | SPICH106-09 | GU683913 | 58.7610 | -93.9540 |
| *Arctosa raptor* | A | 09PROBE-1678-04 | SPICH107-09 | GU683929 | 58.7610 | -93.9540 |
| *Arctosa raptor* | I | CHU-SPI-489 | KKCHE692-07 | KF367906 | 58.7470 | -94.1340 |
| *Arctosa raptor* | I | INV0414 | KKCHE051-06 | KF367904 | 58.6580 | -94.1670 |
| *Baryphyma trifrons* | A | 09PROBE-1644-01 | SPISH008-09 | HM432634 | 58.6190 | -93.8290 |
| *Baryphyma trifrons* | A | BIOUG00200-D07 | SPIRU224-11 | JF886509 | 71.4020 | 102.9960 |
| *Baryphyma trifrons* | A | BIOUG00627-C11 | SPIRU1007-11 | KF367910 | 58.7544 | -93.9980 |
| *Baryphyma trifrons* | I | BIOUG00627-D01 | SPIRU1009-11 | KF367914 | 58.7544 | -93.9980 |
| *Baryphyma trifrons* | I | BIOUG00627-G03 | SPIRU1047-11 | KF367913 | 58.7055 | -94.0536 |
| *Baryphyma trifrons* | A | BIOUG00627-H10 | SPIRU1066-11 | KF367912 | 58.7544 | -93.9980 |
| *Baryphyma trifrons* | I | BIOUG00628-B04 | SPIRU1083-11 | KF367907 | 58.7544 | -93.9980 |
| *Baryphyma trifrons* | I | BIOUG00628-E01 | SPIRU1116-11 | KF367916 | 58.7544 | -93.9980 |
| *Baryphyma trifrons* | A | BIOUG00628-F06 | SPIRU1133-11 | KF367909 | 58.7544 | -93.9980 |
| *Baryphyma trifrons* | A | BIOUG00628-F10 | SPIRU1137-11 | KF367911 | 58.6329 | -93.7869 |
| *Baryphyma trifrons* | I | BIOUG00629-D06 | SPIRU1204-11 | KF367915 | 58.7180 | -94.1240 |
| *Baryphyma trifrons* | A | BIOUG00629-E03 | SPIRU1213-11 | KF367917 | 58.6186 | -93.8291 |
| *Baryphyma trifrons* | A | BIOUG00630-F08 | SPIRU1325-11 | KF367908 | 58.7305 | -93.7805 |
| *Baryphyma trifrons affine* | A | BIOUG00627-H09 | SPIRU1065-11 | KF367919 | 58.7544 | -93.9980 |
| *Baryphyma trifrons affine* | I | BIOUG00628-H06 | SPIRU1157-11 | KF367918 | 58.7045 | -94.1742 |
| *Baryphyma trifrons affine* | A | BIOUG02782-A03 | SPRMA1048-12 | KF408094 | 50.1737 | -115.3000 |
| *Bathyphantes brevipes* | A | 09PROBE-1968-01 | SPICH100-09 | GU683771 | 58.6690 | -94.1600 |
| *Bathyphantes brevipes* | A | 09PROBE-1978-117 | SPICH360-09 | GU684261 | 58.6690 | -94.1600 |
| *Bathyphantes brevipes* | A | 09PROBE-1978-119 | SPICH362-09 | GU684262 | 58.6690 | -94.1600 |
| *Bathyphantes brevipes* | A | 09PROBE-1978-120 | SPICH363-09 | GU684259 | 58.6690 | -94.1600 |
| *Bathyphantes brevipes* | A | 09PROBE-1978-121 | SPICH364-09 | GU684260 | 58.6690 | -94.1600 |
| *Bathyphantes brevipes* | A | 09PROBE-1978-13 | SPICH256-09 | GU684329 | 58.6690 | -94.1600 |
| *Bathyphantes brevipes* | I | 09PROBE-1978-135 | SPICH378-09 | GU684257 | 58.6690 | -94.1600 |
| *Bathyphantes brevipes* | A | 09PROBE-1978-15 | SPICH258-09 | GU684333 | 58.6690 | -94.1600 |
| *Bathyphantes brevipes* | A | 09PROBE-1978-183 | SPICH426-09 | GU684258 | 58.6260 | -94.2300 |
| *Bathyphantes brevipes* | A | 09PROBE-1978-25 | SPICH268-09 | GU684348 | 58.6690 | -94.1600 |
| *Bathyphantes brevipes* | I | 09PROBE-1978-256 | SPICH499-09 | GU684034 | 58.6630 | -94.1670 |
| *Bathyphantes brevipes* | I | 09PROBE-1978-257 | SPICH500-09 | GU684117 | 58.6630 | -94.1670 |
| *Bathyphantes brevipes* | A | 09PROBE-1978-271 | SPICH514-09 | GU684031 | 58.6630 | -94.1670 |
| *Bathyphantes brevipes* | A | 09PROBE-1978-273 | SPICH516-09 | GU684032 | 58.6630 | -94.1670 |
| *Bathyphantes brevipes* | A | 09PROBE-1978-279 | SPICH522-09 | GU684029 | 58.6690 | -94.1600 |
| *Bathyphantes brevipes* | I | 09PROBE-1978-280 | SPICH523-09 | GU684030 | 58.6690 | -94.1600 |
| *Bathyphantes brevipes* | A | 09PROBE-1978-282 | SPICH525-09 | GU684035 | 58.6690 | -94.1600 |
| *Bathyphantes brevipes* | A | 09PROBE-1978-283 | SPICH526-09 | GU684033 | 58.6690 | -94.1600 |
| *Bathyphantes brevipes* | A | 09PROBE-1978-284 | SPICH527-09 | GU684036 | 58.6690 | -94.1600 |
| *Bathyphantes brevipes* | A | 09PROBE-1978-285 | SPICH528-09 | GU684038 | 58.6690 | -94.1600 |
| *Bathyphantes brevipes* | A | 09PROBE-1978-288 | SPICH531-09 | GU684121 | 58.6690 | -94.1600 |
| *Bathyphantes brevipes* | I | 09PROBE-1978-304 | SPICH547-09 | GU684119 | 58.6690 | -94.1600 |
| *Bathyphantes brevipes* | I | 09PROBE-1978-306 | SPICH549-09 | GU683996 | 58.6690 | -94.1600 |
| *Bathyphantes brevipes* | A | 09PROBE-1978-310 | SPICH553-09 | GU683999 | 58.6690 | -94.1600 |
| *Bathyphantes brevipes* | I | 09PROBE-1978-322 | SPICH565-09 | GU684000 | 58.6690 | -94.1600 |
| *Bathyphantes brevipes* | I | 09PROBE-1978-323 | SPICH566-09 | GU683997 | 58.6690 | -94.1600 |
| *Bathyphantes brevipes* | I | 09PROBE-1978-324 | SPICH567-09 | GU684118 | 58.6690 | -94.1600 |
| *Bathyphantes brevipes* | I | 09PROBE-1978-325 | SPICH568-09 | GU683998 | 58.6690 | -94.1600 |
| *Bathyphantes brevipes* | I | 09PROBE-1978-326 | SPICH569-09 | GU683995 | 58.6690 | -94.1600 |
| *Bathyphantes brevipes* | A | 09PROBE-1978-33 | SPICH276-09 | GU684331 | 58.6690 | -94.1600 |
| *Bathyphantes brevipes* | I | 09PROBE-1978-332 | SPICH575-09 | GU684177 | 58.6260 | -94.2300 |
| *Bathyphantes brevipes* | I | 09PROBE-1978-40 | SPICH283-09 | GU684328 | 58.6690 | -94.1600 |
| *Bathyphantes brevipes* | I | 09PROBE-1978-41 | SPICH284-09 | GU684332 | 58.6690 | -94.1600 |
| *Bathyphantes brevipes* | A | 09PROBE-1978-55 | SPICH298-09 | GU684330 | 58.6690 | -94.1600 |
| *Bathyphantes brevipes* | I | 09PROBE-1978-553 | SPICH796-09 | GU684389 | 58.6690 | -94.1600 |
| *Bathyphantes brevipes* | A | 09PROBE-1978-554 | SPICH797-09 | GU684390 | 58.6690 | -94.1600 |
| *Bathyphantes brevipes* | A | 09PROBE-1978-560 | SPICH803-09 | GU684352 | 58.6690 | -94.1600 |
| *Bathyphantes brevipes* | I | 09PROBE-1978-569 | SPICH812-09 | GU684391 | 58.6690 | -94.1600 |
| *Bathyphantes brevipes* | A | 09PROBE-1978-81 | SPICH324-09 | GU684326 | 58.6690 | -94.1600 |
| *Bathyphantes brevipes* | A | 09PROBE-876 | SPICH1097-09 | GU684639 | 58.6630 | -94.1670 |
| *Bathyphantes brevipes* | A | 09PROBE-880 | SPICH1101-09 | GU684640 | 58.6630 | -94.1670 |
| *Bathyphantes brevipes* | A | 09PROBE-937 | SPICH1158-09 | GU684641 | 58.6260 | -94.2300 |
| *Bathyphantes brevis* | A | 09PROBE-1978-12 | SPICH255-09 | GU684337 | 58.6690 | -94.1600 |
| *Bathyphantes brevis* | A | 09PROBE-1978-123 | SPICH366-09 | GU684255 | 58.6690 | -94.1600 |
| *Bathyphantes brevis* | I | 09PROBE-1978-133 | SPICH376-09 | GU684256 | 58.6690 | -94.1600 |
| *Bathyphantes brevis* | A | 09PROBE-1978-14 | SPICH257-09 | GU684336 | 58.6690 | -94.1600 |
| *Bathyphantes brevis* | A | 09PROBE-1978-20 | SPICH263-09 | GU684335 | 58.6690 | -94.1600 |
| *Bathyphantes brevis* | I | 09PROBE-1978-258 | SPICH501-09 | GU684037 | 58.6630 | -94.1670 |
| *Bathyphantes brevis* | A | 09PROBE-1978-290 | SPICH533-09 | GU684123 | 58.6690 | -94.1600 |
| *Bathyphantes brevis* | I | 09PROBE-1978-303 | SPICH546-09 | GU684120 | 58.6690 | -94.1600 |
| *Bathyphantes brevis* | A | 09PROBE-1978-35 | SPICH278-09 | GU684349 | 58.6690 | -94.1600 |
| *Bathyphantes brevis* | A | 09PROBE-1978-48 | SPICH291-09 | GU684334 | 58.6690 | -94.1600 |
| *Bathyphantes brevis* | A | 09PROBE-873 | SPICH1094-09 | GU684638 | 58.6630 | -94.1670 |
| *Bathyphantes canadensis* | A | 09PROBE-1978-49 | SPICH292-09 | GU684338 | 58.6690 | -94.1600 |
| *Bathyphantes canadensis* | I | 09PROBE-1978-53 | SPICH296-09 | GU684339 | 58.6690 | -94.1600 |
| *Bathyphantes eumenis* | A | 09PROBE-629 | SPICH872-09 | HM416915 | 58.7900 | -94.2270 |
| *Bathyphantes gracilis* | I | 09PROBE-1978-313 | SPICH556-09 | HM432632 | 58.6690 | -94.1600 |
| *Bathyphantes pallidus* | A | 09PROBE-1973-01 | SPISH038-09 | GU683961 | 58.6260 | -94.2300 |
| *Bathyphantes pallidus* | A | 09PROBE-1978-242 | SPICH485-09 | GU684040 | 58.6630 | -94.1670 |
| *Bathyphantes pallidus* | A | 09PROBE-1978-556 | SPICH799-09 | GU684388 | 58.6690 | -94.1600 |
| *Bathyphantes reprobus* | A | 09PROBE-1978-110 | SPICH353-09 | GU684265 | 58.6690 | -94.1600 |
| *Bathyphantes reprobus* | I | 09PROBE-1978-98 | SPICH341-09 | GU684267 | 58.6690 | -94.1600 |
| *Bathyphantes reprobus* | I | 09PROBE-812 | SPICH1033-09 | GU684721 | 58.6260 | -94.2300 |
| *Bathyphantes reprobus* | A | 09PROBE-814 | SPICH1035-09 | GU684720 | 58.6260 | -94.2300 |
| *Bathyphantes reprobus* | I | 09PROBE-815 | SPICH1036-09 | GU684722 | 58.6260 | -94.2300 |
| *Bathyphantes reprobus* | A | 09PROBE-816 | SPICH1037-09 | GU684717 | 58.6260 | -94.2300 |
| *Bathyphantes reprobus* | A | 09PROBE-817 | SPICH1038-09 | GU684719 | 58.6260 | -94.2300 |
| *Bathyphantes reprobus* | I | CHU-SPI-435 | KKCHE638-07 | KF367920 | 58.7380 | -93.8190 |
| *Ceraticelus atriceps* | I | 09-PROBE-08132 | JDTGS036-09 | GU679887 | 58.6250 | -93.8190 |
| *Ceraticelus atriceps* | I | 09-PROBE-08138 | JDTGS042-09 | GU679830 | 58.6250 | -93.8190 |
| *Ceraticelus atriceps* | A | BIOUG00629-D04 | SPIRU1202-11 | KF367921 | 58.6290 | -93.7980 |
| *Ceraticelus atriceps* | A | BIOUG00629-D09 | SPIRU1207-11 | KF367922 | 58.6189 | -93.8291 |
| *Ceraticelus crassiceps* | I | 07PROBE-04755 | ERSCH120-07 | KF367925 | 58.7350 | -94.1090 |
| *Ceraticelus crassiceps* | I | 07PROBE-04757 | ERSCH122-07 | KF367924 | 58.7350 | -94.1090 |
| *Ceraticelus crassiceps* | A | 09PROBE-1815-01 | SPICH227-09 | GU683972 | 58.6300 | -93.8190 |
| *Ceraticelus crassiceps* | A | 09PROBE-1817-01 | SPICH229-09 | GU683973 | 58.6300 | -93.8190 |
| *Ceraticelus crassiceps* | I | 09PROBE-1978-166 | SPICH409-09 | GU684219 | 58.6180 | -93.8290 |
| *Ceraticelus crassiceps* | A | BIOUG00627-C04 | SPIRU1000-11 | KF367926 | 58.7544 | -93.9980 |
| *Ceraticelus crassiceps* | A | BIOUG00627-C10 | SPIRU1006-11 | KF367927 | 58.7544 | -93.9980 |
| *Ceraticelus crassiceps* | A | BIOUG00630-A06 | SPIRU1263-11 | KF367923 | 58.7290 | -93.8150 |
| *Ceratinella brunnea* | I | CHU-SPI-391 | KKCHE594-07 | KF367928 | 58.6320 | -93.7860 |
| *Ceratinella ornatula* | A | 09PROBE-1832-01 | SPICH001-09 | GU683858 | 58.6300 | -93.8190 |
| *Ceratinella ornatula* | A | 09PROBE-1843-01 | SPICH012-09 | GU683859 | 58.6300 | -93.8190 |
| *Chalcoscirtus glacialis* | A | BIOUG00630-C01 | SPIRU1282-11 | KF367929 | 58.7710 | -93.8510 |
| *Clubiona bryantae* | A | BIOUG00627-H11 | SPIRU1067-11 | KF367930 | 58.6750 | -93.8416 |
| *Clubiona bryantae* | A | SD2406SG402 | SAPIT134-08 | KF367931 | 58.7300 | -93.8000 |
| *Clubiona furcata* | I | 07PROBE-04766 | ERSCH131-07 | KF367933 | 58.6300 | -93.7980 |
| *Clubiona furcata* | I | 09PROBE-1657-01 | SPISH012-09 | GU683939 | 58.6180 | -93.8290 |
| *Clubiona furcata* | I | 09PROBE-1666-01 | SPISH026-09 | GU683940 | 58.7610 | -93.9540 |
| *Clubiona furcata* | I | 09PROBE-1675-01 | SPISH031-09 | GU683938 | 58.6300 | -93.7980 |
| *Clubiona furcata* | A | 09PROBE-1978-190 | SPICH433-09 | GU684270 | 58.6260 | -94.2300 |
| *Clubiona furcata* | I | 09PROBE-1978-549 | SPICH792-09 | GU684398 | 58.7870 | -93.7140 |
| *Clubiona furcata* | I | 09PROBE-741 | SPICH984-09 | GU684499 | 58.6260 | -94.2300 |
| *Clubiona furcata* | A | 09PROBE-836 | SPICH1057-09 | GU684733 | 58.6630 | -94.1670 |
| *Clubiona furcata* | I | 10PROBE-21145 | GBADC025-10 | HQ956688 | 58.7340 | -94.1120 |
| *Clubiona furcata* | A | BIOUG00627-H08 | SPIRU1064-11 | KF367934 | 58.6329 | -93.7869 |
| *Clubiona furcata* | A | BIOUG00629-C05 | SPIRU1191-11 | KF367932 | 58.7179 | -94.1221 |
| *Clubiona furcata* | I | CHU-SPI-615 | KKCHE818-07 | KF367935 | 58.7400 | -93.8200 |
| *Clubiona norvegica* | I | 07CHU-AR-0050 | KKCHE962-09 | GU683661 | 58.7850 | -94.2030 |
| *Clubiona norvegica* | I | 07CHU-AR-0060 | KKCHE972-09 | GU683673 | 58.7620 | -94.1780 |
| *Clubiona norvegica* | I | 07PROBE-04629 | ERSCH084-07 | KF367940 | 58.6300 | -93.7980 |
| *Clubiona norvegica* | A | 09PROBE-1691-01 | SPICH130-09 | GU683809 | 58.7310 | -93.7800 |
| *Clubiona norvegica* | A | 09PROBE-1691-02 | SPICH131-09 | GU683813 | 58.7310 | -93.7800 |
| *Clubiona norvegica* | A | 09PROBE-1691-03 | SPICH132-09 | GU683806 | 58.7310 | -93.7800 |
| *Clubiona norvegica* | I | 09PROBE-1691-04 | SPICH133-09 | GU683810 | 58.7310 | -93.7800 |
| *Clubiona norvegica* | I | 09PROBE-1714-01 | SPICH169-09 | GU683811 | 58.6690 | -94.1600 |
| *Clubiona norvegica* | I | 09PROBE-1714-02 | SPICH170-09 | GU683808 | 58.6690 | -94.1600 |
| *Clubiona norvegica* | A | 09PROBE-1879-01 | SPICH045-09 | GU683903 | 58.7500 | -94.0840 |
| *Clubiona norvegica* | A | 09PROBE-1978-473 | SPICH716-09 | GU684511 | 58.7640 | -93.8970 |
| *Clubiona norvegica* | A | 09PROBE-1978-474 | SPICH717-09 | GU684516 | 58.7640 | -93.8970 |
| *Clubiona norvegica* | A | 09PROBE-1978-475 | SPICH718-09 | GU684517 | 58.7640 | -93.8970 |
| *Clubiona norvegica* | I | 09PROBE-1978-494 | SPICH737-09 | GU684399 | 58.7890 | -93.7090 |
| *Clubiona norvegica* | I | 09PROBE-1978-495 | SPICH738-09 | GU684400 | 58.7890 | -93.7090 |
| *Clubiona norvegica* | I | 09PROBE-632 | SPICH875-09 | GU684421 | 58.7900 | -94.2270 |
| *Clubiona norvegica* | I | 09PROBE-633 | SPICH876-09 | GU684419 | 58.7900 | -94.2270 |
| *Clubiona norvegica* | I | 09PROBE-634 | SPICH877-09 | GU684418 | 58.7900 | -94.2270 |
| *Clubiona norvegica* | I | 09PROBE-635 | SPICH878-09 | GU684420 | 58.7900 | -94.2270 |
| *Clubiona norvegica* | A | 09PROBE-699 | SPICH942-09 | GU684498 | 58.7900 | -94.2270 |
| *Clubiona norvegica* | I | 09PROBE-803 | SPICH1024-09 | GU684736 | 58.6260 | -94.2300 |
| *Clubiona norvegica* | A | 09PROBE-940 | SPICH1161-09 | GU684590 | 58.6260 | -94.2300 |
| *Clubiona norvegica* | A | BIOUG00627-E09 | SPIRU1029-11 | KF367943 | 58.7055 | -94.0536 |
| *Clubiona norvegica* | I | BIOUG00628-A01 | SPIRU1068-11 | KF367938 | 58.6750 | -93.8416 |
| *Clubiona norvegica* | A | BIOUG00628-D05 | SPIRU1108-11 | KF367942 | 58.6717 | -93.8395 |
| *Clubiona norvegica* | A | BIOUG00628-D07 | SPIRU1110-11 | KF367939 | 58.7055 | -94.0536 |
| *Clubiona norvegica* | I | BIOUG00629-B12 | SPIRU1186-11 | KF367941 | 58.6629 | -94.1679 |
| *Clubiona norvegica* | A | BIOUG00629-C04 | SPIRU1190-11 | KF367936 | 58.7178 | -94.1221 |
| *Clubiona norvegica* | I | CHU-SPI-452 | KKCHE655-07 | KF367944 | 58.7400 | -93.8200 |
| *Clubiona norvegica* | I | JBWM0005957 | KKCHE222-07 | KF367937 | 58.7640 | -93.9390 |
| *Clubiona praematura* | I | 07CHU-AR-0031 | KKCHE943-09 | GU683645 | 58.7050 | -94.0540 |
| *Clubiona praematura* | I | 07CHU-AR-0065 | KKCHE977-09 | GU683674 | 58.7690 | -94.1600 |
| *Clubiona praematura* | I | BIOUG00630-B07 | SPIRU1276-11 | KF367945 | 58.7690 | -93.8620 |
| *Clubiona praematura* | A | BIOUG00630-G04 | SPIRU1333-11 | KF367946 | 58.7690 | -93.8620 |
| *Clubiona trivialis* | I | 07PROBE-04546 | ERSCH001-07 | KF367973 | 58.6170 | -93.8140 |
| *Clubiona trivialis* | I | 07PROBE-04549 | ERSCH004-07 | KF367949 | 58.6170 | -93.8140 |
| *Clubiona trivialis* | I | 07PROBE-04598 | ERSCH053-07 | KF367956 | 58.7330 | -93.8200 |
| *Clubiona trivialis* | I | 07PROBE-04600 | ERSCH055-07 | KF367963 | 58.7330 | -93.8200 |
| *Clubiona trivialis* | I | 07PROBE-04603 | ERSCH058-07 | KF367951 | 58.7330 | -93.8200 |
| *Clubiona trivialis* | I | 07PROBE-04606 | ERSCH061-07 | KF367962 | 58.7330 | -93.8200 |
| *Clubiona trivialis* | I | 07PROBE-04608 | ERSCH063-07 | KF367972 | 58.7330 | -93.8200 |
| *Clubiona trivialis* | I | 07PROBE-04609 | ERSCH064-07 | KF367970 | 58.7330 | -93.8200 |
| *Clubiona trivialis* | I | 07PROBE-04612 | ERSCH067-07 | KF367969 | 58.7330 | -93.8200 |
| *Clubiona trivialis* | I | 07PROBE-04614 | ERSCH069-07 | KF367961 | 58.7330 | -93.8200 |
| *Clubiona trivialis* | I | 07PROBE-04627 | ERSCH082-07 | KF367952 | 58.6300 | -93.7980 |
| *Clubiona trivialis* | I | 07PROBE-04737 | ERSCH102-07 | KF367971 | 58.7350 | -94.1090 |
| *Clubiona trivialis* | I | 09-PROBE-08142 | JDTGS046-09 | GU679876 | 58.6250 | -93.8190 |
| *Clubiona trivialis* | I | 09-PROBE-08143 | JDTGS047-09 | GU679877 | 58.6250 | -93.8190 |
| *Clubiona trivialis* | I | 09-PROBE-08149 | JDTGS053-09 | GU679873 | 58.6180 | -93.8290 |
| *Clubiona trivialis* | A | 09PROBE-1636-01 | SPISH007-09 | GU683937 | 58.7960 | -93.7540 |
| *Clubiona trivialis* | A | 09PROBE-1685-01 | SPICH120-09 | GU683814 | 58.7310 | -93.7800 |
| *Clubiona trivialis* | A | 09PROBE-1685-02 | SPICH121-09 | GU683812 | 58.7310 | -93.7800 |
| *Clubiona trivialis* | I | 09PROBE-1712-01 | SPICH166-09 | GU683815 | 58.6180 | -93.8110 |
| *Clubiona trivialis* | A | 09PROBE-1888-01 | SPICH054-09 | GU683904 | 58.7500 | -94.0840 |
| *Clubiona trivialis* | A | 09PROBE-583 | SPICH826-09 | GU684417 | 58.6180 | -93.8290 |
| *Clubiona trivialis* | A | 09PROBE-789 | SPICH1010-09 | GU684737 | 58.6300 | -93.7980 |
| *Clubiona trivialis* | A | 09PROBE-790 | SPICH1011-09 | GU684735 | 58.6300 | -93.7980 |
| *Clubiona trivialis* | I | 10PROBE-21137 | GBADC017-10 | HQ956680 | 58.7340 | -94.1120 |
| *Clubiona trivialis* | A | BIOUG00627-B02 | SPIRU986-11 | KF367965 | 58.7550 | -93.9150 |
| *Clubiona trivialis* | A | BIOUG00627-G12 | SPIRU1056-11 | KF367968 | 58.6761 | -94.1442 |
| *Clubiona trivialis* | I | BIOUG00627-H02 | SPIRU1058-11 | KF367947 | 58.6761 | -94.1442 |
| *Clubiona trivialis* | I | BIOUG00627-H03 | SPIRU1059-11 | KF367955 | 58.6761 | -94.1442 |
| *Clubiona trivialis* | A | BIOUG00627-H04 | SPIRU1060-11 | KF367954 | 58.6761 | -94.1442 |
| *Clubiona trivialis* | A | BIOUG00627-H05 | SPIRU1061-11 | KF367953 | 58.6761 | -94.1442 |
| *Clubiona trivialis* | A | BIOUG00627-H07 | SPIRU1063-11 | KF367948 | 58.6329 | -93.7869 |
| *Clubiona trivialis* | I | BIOUG00628-C02 | SPIRU1093-11 | KF367964 | 58.6761 | -94.1442 |
| *Clubiona trivialis* | A | BIOUG00628-C03 | SPIRU1094-11 | KF367967 | 58.6761 | -94.1442 |
| *Clubiona trivialis* | A | BIOUG00629-A10 | SPIRU1172-11 | KF367958 | 58.6250 | -93.8190 |
| *Clubiona trivialis* | A | BIOUG00630-F10 | SPIRU1327-11 | KF367960 | 58.7305 | -93.7805 |
| *Clubiona trivialis* | I | CHU-SPI-069 | KKCHE178-06 | KF367966 | 58.6220 | -93.8100 |
| *Clubiona trivialis* | I | CHU-SPI-416 | KKCHE619-07 | KF367950 | 58.7540 | -93.9130 |
| *Clubiona trivialis* | I | CHU-SPI-463 | KKCHE666-07 | KF367957 | 58.7540 | -93.9130 |
| *Clubiona trivialis* | I | JBWM0005960 | KKCHE223-07 | KF367959 | 58.7470 | -94.1340 |
| *Cnephalocotes obscurus* | I | 09PROBE-1737-01 | SPICH176-09 | GU684019 | 58.7640 | -93.8970 |
| *Cnephalocotes obscurus* | I | 09PROBE-1895-01 | SPICH060-09 | GU683847 | 58.7700 | -93.8430 |
| *Cnephalocotes obscurus* | A | 09PROBE-1949-01 | SPICH088-09 | GU683832 | 58.7640 | -93.8970 |
| *Cnephalocotes obscurus* | A | 09PROBE-1978-162 | SPICH405-09 | GU684235 | 58.6180 | -93.8290 |
| *Cnephalocotes obscurus* | A | 09PROBE-1978-167 | SPICH410-09 | GU684238 | 58.6180 | -93.8290 |
| *Cnephalocotes obscurus* | A | 09PROBE-1978-168 | SPICH411-09 | GU684264 | 58.6180 | -93.8290 |
| *Cnephalocotes obscurus* | A | 09PROBE-1978-169 | SPICH412-09 | GU684263 | 58.6180 | -93.8290 |
| *Cnephalocotes obscurus* | A | BIOUG00630-G01 | SPIRU1330-11 | KF367974 | 58.7690 | -93.8620 |
| *Coriarachne brunneipes* | A | BIOUG00628-C04 | SPIRU1095-11 | KF367975 | 58.6761 | -94.1442 |
| *Cybaeopsis euopla* | A | 09PROBE-755 | SPICH998-09 | GU684503 | 58.7310 | -93.7800 |
| *Cybaeopsis euopla* | A | 09PROBE-756 | SPICH999-09 | GU684504 | 58.7310 | -93.7800 |
| *Dictyna brevitarsa* | I | 07PROBE-04547 | ERSCH002-07 | KF367987 | 58.6170 | -93.8140 |
| *Dictyna brevitarsa* | A | 07PROBE-04558 | ERSCH013-07 | KF367979 | 58.7310 | -93.7800 |
| *Dictyna brevitarsa* | I | 07PROBE-04621 | ERSCH076-07 | KF367980 | 58.7330 | -93.8200 |
| *Dictyna brevitarsa* | I | 09PROBE-01587 | SWSWE017-09 | GU681079 | 58.6300 | -93.8190 |
| *Dictyna brevitarsa* | I | 09-PROBE-08128 | JDTGS032-09 | GU679891 | 58.6250 | -93.8190 |
| *Dictyna brevitarsa* | I | 09-PROBE-08130 | JDTGS034-09 | GU679889 | 58.6250 | -93.8190 |
| *Dictyna brevitarsa* | I | 09-PROBE-08139 | JDTGS043-09 | GU679881 | 58.6250 | -93.8190 |
| *Dictyna brevitarsa* | I | 09PROBE-1711-01 | SPICH161-09 | GU683821 | 58.6180 | -93.8110 |
| *Dictyna brevitarsa* | I | 09PROBE-1711-04 | SPICH164-09 | GU683820 | 58.6180 | -93.8110 |
| *Dictyna brevitarsa* | I | 09PROBE-1711-05 | SPICH165-09 | GU683818 | 58.6180 | -93.8110 |
| *Dictyna brevitarsa* | A | 09PROBE-735 | SPICH978-09 | GU684497 | 58.7640 | -93.8970 |
| *Dictyna brevitarsa* | A | 09PROBE-818 | SPICH1039-09 | GU684709 | 58.6300 | -93.7980 |
| *Dictyna brevitarsa* | A | 09PROBE-820 | SPICH1041-09 | GU684703 | 58.6300 | -93.7980 |
| *Dictyna brevitarsa* | A | 09PROBE-821 | SPICH1042-09 | GU684710 | 58.6300 | -93.7980 |
| *Dictyna brevitarsa* | A | 09PROBE-823 | SPICH1044-09 | GU684707 | 58.6300 | -93.7980 |
| *Dictyna brevitarsa* | A | 09PROBE-824 | SPICH1045-09 | GU684712 | 58.6300 | -93.7980 |
| *Dictyna brevitarsa* | A | 09PROBE-828 | SPICH1049-09 | GU684708 | 58.6300 | -93.7980 |
| *Dictyna brevitarsa* | A | 09PROBE-831 | SPICH1052-09 | GU684705 | 58.6300 | -93.7980 |
| *Dictyna brevitarsa* | I | 09PROBE-832 | SPICH1053-09 | GU684706 | 58.6300 | -93.7980 |
| *Dictyna brevitarsa* | I | 10PROBE-21129 | GBADC009-10 | HQ956673 | 58.7340 | -94.1120 |
| *Dictyna brevitarsa* | A | BIOUG00627-A02 | SPIRU974-11 | KF367985 | 58.6340 | -93.7860 |
| *Dictyna brevitarsa* | A | BIOUG00628-A12 | SPIRU1079-11 | KF367983 | 58.7544 | -93.9980 |
| *Dictyna brevitarsa* | A | BIOUG00629-B09 | SPIRU1183-11 | KF367984 | 58.6921 | -94.1320 |
| *Dictyna brevitarsa* | A | BIOUG00629-E02 | SPIRU1212-11 | KF367986 | 58.6185 | -93.8290 |
| *Dictyna brevitarsa* | I | CHU-SPI-007 | KKCHE116-06 | KF367988 | 58.6180 | -93.8230 |
| *Dictyna brevitarsa* | I | CHU-SPI-010 | KKCHE119-06 | KF367976 | 58.6220 | -93.8100 |
| *Dictyna brevitarsa* | I | CHU-SPI-103 | KKCHE212-06 | KF367982 | 58.6820 | -94.1520 |
| *Dictyna brevitarsa* | I | CHU-SPI-131 | KKCHE334-07 | KF367978 | 58.6170 | -93.8210 |
| *Dictyna brevitarsa* | I | CHU-SPI-179 | KKCHE382-07 | KF367977 | 58.6170 | -93.8210 |
| *Dictyna brevitarsa* | A | CHU-SPI-283 | KKCHE486-07 | KF367981 | 58.6180 | -93.8230 |
| *Dictyna major* | I | 07CHU-AR-0034 | KKCHE946-09 | GU683650 | 58.7610 | -93.9520 |
| *Dictyna major* | I | 07PROBE-04569 | ERSCH024-07 | KF368004 | 58.7310 | -93.7800 |
| *Dictyna major* | I | 07PROBE-04572 | ERSCH027-07 | KF367994 | 58.7310 | -93.7800 |
| *Dictyna major* | I | 07PROBE-04728 | ERSCH093-07 | KF368000 | 58.7350 | -94.1090 |
| *Dictyna major* | I | 07PROBE-04746 | ERSCH111-07 | KF367993 | 58.7350 | -94.1090 |
| *Dictyna major* | I | 07PROBE-04748 | ERSCH113-07 | KF368001 | 58.7350 | -94.1090 |
| *Dictyna major* | I | 07PROBE-04749 | ERSCH114-07 | KF367989 | 58.7350 | -94.1090 |
| *Dictyna major* | I | 07PROBE-04750 | ERSCH115-07 | KF367998 | 58.7350 | -94.1090 |
| *Dictyna major* | A | 08BBARAC-0505 | ARSO633-09 | KF408101 | 49.1530 | -107.5170 |
| *Dictyna major* | A | 09ONTGAB-083 | ERSPI404-09 | GU682448 | 43.5283 | -80.2287 |
| *Dictyna major* | I | 09PROBE-01579 | SWSWE009-09 | GU681078 | 58.6900 | -93.8500 |
| *Dictyna major* | I | 09PROBE-1695-01 | SPICH140-09 | GU683816 | 58.7310 | -93.7800 |
| *Dictyna major* | I | 09PROBE-1711-02 | SPICH162-09 | GU683817 | 58.6180 | -93.8110 |
| *Dictyna major* | I | 09PROBE-1711-03 | SPICH163-09 | GU683819 | 58.6180 | -93.8110 |
| *Dictyna major* | A | 09PROBE-1978-483 | SPICH726-09 | GU684395 | 58.6300 | -94.2300 |
| *Dictyna major* | A | 09PROBE-1978-484 | SPICH727-09 | GU684396 | 58.6300 | -94.2300 |
| *Dictyna major* | A | 09PROBE-589 | SPICH832-09 | GU684430 | 58.7900 | -94.2270 |
| *Dictyna major* | A | 09PROBE-590 | SPICH833-09 | GU684431 | 58.7900 | -94.2270 |
| *Dictyna major* | A | 09PROBE-599 | SPICH842-09 | GU684429 | 58.7310 | -93.7800 |
| *Dictyna major* | I | 09PROBE-665 | SPICH908-09 | GU684432 | 58.6190 | -93.8290 |
| *Dictyna major* | A | 09PROBE-819 | SPICH1040-09 | GU684715 | 58.6300 | -93.7980 |
| *Dictyna major* | A | 09PROBE-827 | SPICH1048-09 | GU684716 | 58.6300 | -93.7980 |
| *Dictyna major* | I | 10PROBE-21160 | GBADC040-10 | HQ956702 | 58.7340 | -94.1120 |
| *Dictyna major* | I | 10PROBE-21164 | GBADC044-10 | HQ956705 | 58.7340 | -94.1120 |
| *Dictyna major* | I | 10PROBE-21180 | GBADC060-10 | HQ956721 | 58.6340 | -93.7860 |
| *Dictyna major* | I | 10PROBE-21212 | GBADC092-10 | HQ956750 | 58.7180 | -94.1240 |
| *Dictyna major* | A | 10-SKBC-0469 | SPRMA469-10 | JF887016 | 49.2513 | -119.6180 |
| *Dictyna major* | A | 10-SKBC-0683 | SPRMA683-10 | JF887121 | 60.9350 | -134.8270 |
| *Dictyna major* | I | BIOUG00509-A12 | SPICA700-10 | JF885880 | 52.2211 | -117.2220 |
| *Dictyna major* | I | BIOUG00518-G11 | SPICA1594-10 | JF885176 | 52.8081 | -118.0230 |
| *Dictyna major* | A | BIOUG00627-A11 | SPIRU983-11 | KF367991 | 58.7304 | -93.7805 |
| *Dictyna major* | A | BIOUG00627-B06 | SPIRU990-11 | KF367992 | 58.7177 | -94.1224 |
| *Dictyna major* | A | BIOUG00627-E11 | SPIRU1031-11 | KF368003 | 58.6192 | -93.8291 |
| *Dictyna major* | A | BIOUG00627-F09 | SPIRU1041-11 | KF367995 | 58.6329 | -93.7869 |
| *Dictyna major* | I | BIOUG00628-D11 | SPIRU1114-11 | KF367997 | 58.7304 | -93.7805 |
| *Dictyna major* | I | BIOUG00628-D12 | SPIRU1115-11 | KF368002 | 58.7304 | -93.7805 |
| *Dictyna major* | A | BIOUG00628-H10 | SPIRU1161-11 | KF367999 | 58.6329 | -93.7869 |
| *Dictyna major* | A | BIOUG00629-C06 | SPIRU1192-11 | KF367990 | 58.7179 | -94.1221 |
| *Dictyna major* | A | BIOUG00629-D11 | SPIRU1209-11 | KF367996 | 58.6191 | -93.8289 |
| *Dictyna major* | I | BIOUG01889-B01 | SPIRU1523-12 | KF408097 | 69.6560 | 170.2060 |
| *Dictyna major* | A | BIOUG01889-B05 | SPIRU1527-12 | KF408102 | 69.6560 | 170.2060 |
| *Dictyna major* | A | CCDB-05269-F01 | BBCAN620-09 | GU683557 | 49.4780 | -57.9730 |
| *Dictyna major* | A | CCDB-05269-F02 | BBCAN621-09 | GU683558 | 49.4780 | -57.9730 |
| *Diplocentria bidentata* | I | 09PROBE-1739-01 | SPICH178-09 | GU684003 | 58.7640 | -93.8970 |
| *Diplocentria bidentata* | A | 09PROBE-1821-01 | SPICH233-09 | GU683985 | 58.6300 | -93.8190 |
| *Diplocentria bidentata* | I | 09PROBE-1823-01 | SPICH235-09 | GU683986 | 58.6300 | -93.8190 |
| *Diplocentria bidentata* | A | 09PROBE-1845-01 | SPICH014-09 | GU683889 | 58.6300 | -93.8190 |
| *Diplocentria bidentata* | I | 09PROBE-1855-01 | SPICH024-09 | GU683887 | 58.6300 | -93.8190 |
| *Diplocentria bidentata* | A | 09PROBE-1857-01 | SPICH026-09 | GU683828 | 58.6300 | -93.8190 |
| *Diplocentria bidentata* | A | 09PROBE-1870-01 | SPICH039-09 | GU683888 | 58.6300 | -93.8190 |
| *Diplocentria bidentata* | I | 09PROBE-1978-147 | SPICH390-09 | GU684190 | 58.6300 | -93.7980 |
| *Diplocentria bidentata* | A | 09PROBE-1978-160 | SPICH403-09 | GU684191 | 58.6180 | -93.8290 |
| *Diplocentria bidentata* | A | 09PROBE-1978-165 | SPICH408-09 | GU684187 | 58.6180 | -93.8290 |
| *Diplocentria bidentata* | A | 09PROBE-1978-171 | SPICH414-09 | GU684189 | 58.6180 | -93.8290 |
| *Diplocentria bidentata* | A | 09PROBE-1978-172 | SPICH415-09 | GU684192 | 58.6180 | -93.8290 |
| *Diplocentria bidentata* | I | 09PROBE-1978-188 | SPICH431-09 | GU684194 | 58.6260 | -94.2300 |
| *Diplocentria bidentata* | I | 09PROBE-1978-195 | SPICH438-09 | GU684052 | 58.6300 | -93.7980 |
| *Diplocentria bidentata* | A | 09PROBE-1978-197 | SPICH440-09 | GU684054 | 58.6300 | -93.7980 |
| *Diplocentria bidentata* | A | 09PROBE-1978-205 | SPICH448-09 | GU684055 | 58.6300 | -93.7980 |
| *Diplocentria bidentata* | A | 09PROBE-1978-209 | SPICH452-09 | GU684050 | 58.6300 | -93.7980 |
| *Diplocentria bidentata* | A | 09PROBE-1978-214 | SPICH457-09 | GU684053 | 58.6300 | -93.7980 |
| *Diplocentria bidentata* | A | 09PROBE-1978-335 | SPICH578-09 | GU684178 | 58.6260 | -94.2300 |
| *Diplocentria bidentata* | A | BIOUG00630-E03 | SPIRU1308-11 | KF368006 | 58.7305 | -93.7805 |
| *Diplocentria bidentata* | A | BIOUG00630-G11 | SPIRU1340-11 | KF368005 | 58.6173 | -93.8123 |
| *Diplocentria bidentata* | A | BIOUG00630-H03 | SPIRU1344-11 | KF368007 | 58.6173 | -93.8123 |
| *Diplocentria rectangulata* | A | 09PROBE-1816-01 | SPICH228-09 | GU683987 | 58.6300 | -93.8190 |
| *Diplocentria rectangulata* | A | 09PROBE-1847-01 | SPICH016-09 | GU683886 | 58.6300 | -93.8190 |
| *Diplocentria rectangulata* | A | 09PROBE-1978-164 | SPICH407-09 | GU684188 | 58.6180 | -93.8290 |
| *Dismodicus decemoculatus* | I | 09PROBE-1751-01 | SPICH185-09 | GU684015 | 58.7310 | -93.7800 |
| *Dismodicus decemoculatus* | A | 09PROBE-1885-01 | SPICH051-09 | GU683835 | 58.7500 | -94.0840 |
| *Dismodicus decemoculatus* | A | 09PROBE-1978-04 | SPICH247-09 | GU684292 | 58.6690 | -94.1600 |
| *Dismodicus decemoculatus* | A | 09PROBE-1978-06 | SPICH249-09 | GU684295 | 58.6690 | -94.1600 |
| *Dismodicus decemoculatus* | A | 09PROBE-1978-394 | SPICH637-09 | GU684529 | 58.6250 | -93.8160 |
| *Dismodicus decemoculatus* | A | 09PROBE-1978-410 | SPICH653-09 | GU684530 | 58.7640 | -93.8970 |
| *Dismodicus decemoculatus* | A | 09PROBE-1978-58 | SPICH301-09 | GU684293 | 58.6690 | -94.1600 |
| *Dismodicus decemoculatus* | I | 09PROBE-738 | SPICH981-09 | GU684467 | 58.6260 | -94.2300 |
| *Dismodicus decemoculatus* | I | 09PROBE-797 | SPICH1018-09 | GU684683 | 58.7380 | -93.8190 |
| *Dismodicus decemoculatus* | A | 09PROBE-802 | SPICH1023-09 | GU684687 | 58.6260 | -94.2300 |
| *Dismodicus decemoculatus* | A | 09PROBE-810 | SPICH1031-09 | GU684697 | 58.6260 | -94.2300 |
| *Dismodicus decemoculatus* | A | 09PROBE-837 | SPICH1058-09 | GU684680 | 58.6630 | -94.1670 |
| *Dismodicus decemoculatus* | A | 09PROBE-838 | SPICH1059-09 | GU684691 | 58.6630 | -94.1670 |
| *Dismodicus decemoculatus* | A | 09PROBE-839 | SPICH1060-09 | GU684688 | 58.6630 | -94.1670 |
| *Dismodicus decemoculatus* | A | 09PROBE-840 | SPICH1061-09 | GU684681 | 58.6630 | -94.1670 |
| *Dismodicus decemoculatus* | A | 09PROBE-841 | SPICH1062-09 | GU684682 | 58.6630 | -94.1670 |
| *Dismodicus decemoculatus* | A | 09PROBE-842 | SPICH1063-09 | GU684694 | 58.6630 | -94.1670 |
| *Dismodicus decemoculatus* | I | 09PROBE-843 | SPICH1064-09 | GU684678 | 58.6630 | -94.1670 |
| *Dismodicus decemoculatus* | A | 09PROBE-844 | SPICH1065-09 | GU684690 | 58.6630 | -94.1670 |
| *Dismodicus decemoculatus* | A | 09PROBE-845 | SPICH1066-09 | GU684689 | 58.6630 | -94.1670 |
| *Dismodicus decemoculatus* | A | 09PROBE-846 | SPICH1067-09 | GU684686 | 58.6630 | -94.1670 |
| *Dismodicus decemoculatus* | A | 09PROBE-847 | SPICH1068-09 | GU684684 | 58.6630 | -94.1670 |
| *Dismodicus decemoculatus* | A | 09PROBE-848 | SPICH1069-09 | GU684679 | 58.6630 | -94.1670 |
| *Dismodicus decemoculatus* | A | 09PROBE-850 | SPICH1071-09 | GU684695 | 58.6630 | -94.1670 |
| *Dismodicus decemoculatus* | A | 09PROBE-851 | SPICH1072-09 | GU684676 | 58.6630 | -94.1670 |
| *Dismodicus decemoculatus* | A | 09PROBE-852 | SPICH1073-09 | GU684685 | 58.6630 | -94.1670 |
| *Dismodicus decemoculatus* | A | 09PROBE-853 | SPICH1074-09 | GU684677 | 58.6630 | -94.1670 |
| *Dismodicus decemoculatus* | I | 09PROBE-854 | SPICH1075-09 | GU684693 | 58.6630 | -94.1670 |
| *Dismodicus decemoculatus* | A | 09PROBE-855 | SPICH1076-09 | GU684692 | 58.6630 | -94.1670 |
| *Dismodicus decemoculatus* | I | 09PROBE-886 | SPICH1107-09 | GU684625 | 58.6260 | -94.2300 |
| *Dismodicus decemoculatus* | A | 09PROBE-888 | SPICH1109-09 | GU684628 | 58.6260 | -94.2300 |
| *Dismodicus decemoculatus* | I | 09PROBE-889 | SPICH1110-09 | GU684626 | 58.6260 | -94.2300 |
| *Dismodicus decemoculatus* | A | 09PROBE-933 | SPICH1154-09 | GU684627 | 58.6260 | -94.2300 |
| *Dismodicus decemoculatus* | A | 09PROBE-935 | SPICH1156-09 | GU684624 | 58.6260 | -94.2300 |
| *Dismodicus decemoculatus* | I | 10PROBE-21151 | GBADC031-10 | HQ956694 | 58.7340 | -94.1120 |
| *Dismodicus decemoculatus* | A | BIOUG00627-B04 | SPIRU988-11 | KF368010 | 58.7306 | -93.7804 |
| *Dismodicus decemoculatus* | A | BIOUG00628-B05 | SPIRU1084-11 | KF368011 | 58.7544 | -93.9980 |
| *Dismodicus decemoculatus* | A | BIOUG00628-B06 | SPIRU1085-11 | KF368008 | 58.7544 | -93.9980 |
| *Dismodicus decemoculatus* | I | BIOUG00629-D07 | SPIRU1205-11 | KF368009 | 58.7180 | -94.1240 |
| *Drassodes mirus* | A | 07CHU-AR-0251 | KKCHE1058-09 | GU683734 | 58.6780 | -94.1460 |
| *Drassodes mirus* | A | 07CHU-AR-0252 | KKCHE1059-09 | GU683733 | 58.6780 | -94.1460 |
| *Drassodes mirus* | I | 07CHU-AR-0257 | KKCHE1064-09 | GU683739 | 58.7050 | -94.0540 |
| *Drassodes mirus* | A | 09PROBE-1524-01 | SPISH001-09 | GU683947 | 58.7630 | -93.8660 |
| *Drassodes mirus* | A | 09PROBE-1673-01 | SPISH030-09 | GU683948 | 58.6300 | -93.7980 |
| *Drassodes mirus* | I | 09PROBE-1757-01 | SPICH188-09 | GU683804 | 58.7310 | -93.7800 |
| *Drassodes mirus* | A | 09PROBE-644 | SPICH887-09 | GU684422 | 58.6300 | -93.7980 |
| *Drassodes neglectus* | A | 09PROBE-1660-01 | SPISH016-09 | GU683952 | 58.6180 | -93.8290 |
| *Drassodes neglectus* | I | 09PROBE-1702-01 | SPICH148-09 | GU683807 | 58.6180 | -93.8290 |
| *Drassodes neglectus* | I | 09PROBE-1715-01 | SPICH171-09 | GU683951 | 58.6180 | -93.8290 |
| *Drassodes neglectus* | I | 09PROBE-1715-02 | SPICH172-09 | GU683949 | 58.6180 | -93.8290 |
| *Drassodes neglectus* | I | 09PROBE-1715-03 | SPICH173-09 | GU683950 | 58.6180 | -93.8290 |
| *Drassodes neglectus* | A | 09PROBE-941 | SPICH1162-09 | GU684591 | 58.7310 | -93.7800 |
| *Emblyna annulipes* | I | 07PROBE-04726 | ERSCH091-07 | KF368014 | 58.6300 | -93.7980 |
| *Emblyna annulipes* | I | 09-PROBE-08126 | JDTGS030-09 | GU679893 | 58.6250 | -93.8190 |
| *Emblyna annulipes* | I | 09-PROBE-08127 | JDTGS031-09 | GU679890 | 58.6250 | -93.8190 |
| *Emblyna annulipes* | I | 09-PROBE-08133 | JDTGS037-09 | GU679884 | 58.6250 | -93.8190 |
| *Emblyna annulipes* | I | 09-PROBE-08134 | JDTGS038-09 | GU679885 | 58.6250 | -93.8190 |
| *Emblyna annulipes* | I | 09-PROBE-08135 | JDTGS039-09 | GU679882 | 58.6250 | -93.8190 |
| *Emblyna annulipes* | I | 09-PROBE-08137 | JDTGS041-09 | GU679880 | 58.6250 | -93.8190 |
| *Emblyna annulipes* | I | 09-PROBE-08141 | JDTGS045-09 | GU679879 | 58.6250 | -93.8190 |
| *Emblyna annulipes* | I | 09-PROBE-08154 | JDTGS058-09 | GU679869 | 58.6250 | -93.8190 |
| *Emblyna annulipes* | I | 09-PROBE-08165 | JDTGS069-09 | GU679858 | 58.6920 | -94.1320 |
| *Emblyna annulipes* | I | 09-PROBE-08182 | JDTGS086-09 | GU679851 | 58.6300 | -93.8190 |
| *Emblyna annulipes* | I | 09-PROBE-08184 | JDTGS088-09 | GU679849 | 58.6250 | -93.8190 |
| *Emblyna annulipes* | A | 09PROBE-825 | SPICH1046-09 | GU684704 | 58.6300 | -93.7980 |
| *Emblyna annulipes* | A | 09PROBE-826 | SPICH1047-09 | GU684702 | 58.6300 | -93.7980 |
| *Emblyna annulipes* | A | 09PROBE-829 | SPICH1050-09 | GU684699 | 58.6300 | -93.7980 |
| *Emblyna annulipes* | A | 09PROBE-830 | SPICH1051-09 | GU684701 | 58.6300 | -93.7980 |
| *Emblyna annulipes* | I | BIOUG00629-A08 | SPIRU1170-11 | KF368013 | 58.6250 | -93.8190 |
| *Emblyna annulipes* | I | BIOUG00629-A09 | SPIRU1171-11 | KF368012 | 58.6250 | -93.8190 |
| *Emblyna manitoba* | I | 09PROBE-01624 | SWSWE054-09 | GU681043 | 58.6600 | -93.8300 |
| *Emblyna manitoba* | I | 10PROBE-21171 | GBADC051-10 | HQ956712 | 58.6340 | -93.7860 |
| *Emblyna manitoba* | A | BIOUG00627-B07 | SPIRU991-11 | KF368015 | 58.7177 | -94.1224 |
| *Emblyna manitoba* | I | BIOUG00627-B08 | SPIRU992-11 | KF368017 | 58.7177 | -94.1224 |
| *Emblyna manitoba* | I | BIOUG00627-F10 | SPIRU1042-11 | KF368016 | 58.6329 | -93.7869 |
| *Emblyna peragrata* | A | 09PROBE-600 | SPICH843-09 | GU684408 | 58.7310 | -93.7800 |
| *Emblyna peragrata* | A | 09PROBE-601 | SPICH844-09 | GU684428 | 58.7310 | -93.7800 |
| *Emblyna peragrata* | A | 09PROBE-822 | SPICH1043-09 | GU684713 | 58.6300 | -93.7980 |
| *Emblyna peragrata* | A | 09PROBE-833 | SPICH1054-09 | GU684714 | 58.6300 | -93.7980 |
| *Emblyna peragrata* | A | 09PROBE-834 | SPICH1055-09 | GU684711 | 58.6300 | -93.7980 |
| *Enoplognatha intrepida* | A | 09PROBE-594 | SPICH837-09 | GU684461 | 58.7900 | -94.2270 |
| *Enoplognatha intrepida* | A | 09PROBE-595 | SPICH838-09 | GU684462 | 58.7900 | -94.2270 |
| *Enoplognatha intrepida* | A | 09PROBE-624 | SPICH867-09 | GU684411 | 58.7900 | -94.2270 |
| *Enoplognatha intrepida* | A | 09PROBE-689 | SPICH932-09 | GU684491 | 58.7900 | -94.2270 |
| *Enoplognatha intrepida* | A | 09PROBE-691 | SPICH934-09 | GU684495 | 58.7900 | -94.2270 |
| *Enoplognatha intrepida* | A | 09PROBE-692 | SPICH935-09 | GU684493 | 58.7900 | -94.2270 |
| *Enoplognatha intrepida* | A | 09PROBE-693 | SPICH936-09 | GU684492 | 58.7900 | -94.2270 |
| *Enoplognatha intrepida* | A | 09PROBE-694 | SPICH937-09 | GU684494 | 58.7900 | -94.2270 |
| *Enoplognatha intrepida* | A | 09PROBE-903 | SPICH1124-09 | GU684642 | 58.6260 | -94.2300 |
| *Entelecara* sp. 1GAB | A | 09PROBE-614 | SPICH857-09 | GU684439 | 58.7900 | -94.2270 |
| *Entelecara* sp. 1GAB | A | 09PROBE-626 | SPICH869-09 | GU684440 | 58.7900 | -94.2270 |
| *Erigone aletris* | A | 09PROBE-1978-373 | SPICH616-09 | GU684152 | 58.7640 | -93.8970 |
| *Erigone aletris* | A | 09PROBE-1978-423 | SPICH666-09 | GU684533 | 58.7640 | -93.8970 |
| *Erigone aletris* | A | 09PROBE-1978-427 | SPICH670-09 | GU684534 | 58.7640 | -93.8970 |
| *Erigone aletris* | A | 09PROBE-770 | SPISH052-09 | GU684672 | 58.7640 | -93.8970 |
| *Erigone aletris* | A | BIOUG00629-H03 | SPIRU1249-11 | KF368021 | 58.7710 | -93.8510 |
| *Erigone aletris* | A | BIOUG00630-C10 | SPIRU1291-11 | KF368022 | 58.7666 | -93.8530 |
| *Erigone aletris* | I | BIOUG00632-B09 | SPIRU1373-11 | KF368019 | 58.7710 | -93.8510 |
| *Erigone aletris* | A | BIOUG00632-C02 | SPIRU1378-11 | KF368018 | 58.7710 | -93.8510 |
| *Erigone aletris* | A | BIOUG00632-C09 | SPIRU1385-11 | KF368020 | 58.7710 | -93.8510 |
| *Erigone arctica* | A | 09-PROBE-08144 | JDTGS048-09 | GU679874 | 58.7380 | -93.8180 |
| *Erigone arctica* | A | 09PROBE-1969-01 | SPICH101-09 | GU684006 | 58.6690 | -94.1600 |
| *Erigone arctica* | A | 09PROBE-1972-01 | SPISH037-09 | GU683975 | 58.6260 | -94.2300 |
| *Erigone arctica* | A | 09PROBE-1978-512 | SPICH755-09 | GU684373 | 58.7960 | -93.7540 |
| *Erigone arctica* | A | 09PROBE-1978-533 | SPICH776-09 | GU684376 | 58.7960 | -93.7540 |
| *Erigone arctica* | A | 09PROBE-1978-534 | SPICH777-09 | GU684375 | 58.7960 | -93.7540 |
| *Erigone arctica* | A | 09PROBE-1978-543 | SPICH786-09 | GU684377 | 58.7960 | -93.7540 |
| *Erigone arctica* | I | 09PROBE-1978-544 | SPICH787-09 | GU684374 | 58.7960 | -93.7540 |
| *Erigone arctica* | A | BIOUG00629-H04 | SPIRU1250-11 | KF368023 | 58.7710 | -93.8510 |
| *Erigone arctica* | A | BIOUG00632-B10 | SPIRU1374-11 | KF368024 | 58.7710 | -93.8510 |
| *Erigone arctophylacis* | A | 09PROBE-605 | SPICH848-09 | GU684448 | 58.7900 | -94.2270 |
| *Erigone arctophylacis* | A | 09PROBE-615 | SPICH858-09 | GU684410 | 58.7900 | -94.2270 |
| *Erigone arctophylacis* | A | 09PROBE-712 | SPICH955-09 | GU684471 | 58.7900 | -94.2270 |
| *Erigone arctophylacis* | A | 09PROBE-771 | SPISH053-09 | GU684675 | 58.7960 | -93.7540 |
| *Erigone cristatopalpus* | A | 09PROBE-1676-01 | SPICH103-09 | GU684004 | 58.6630 | -94.1680 |
| *Erigone cristatopalpus* | I | 09PROBE-1701-01 | SPICH147-09 | GU684005 | 58.6630 | -94.1680 |
| *Erigone cristatopalpus* | I | 09PROBE-1978-132 | SPICH375-09 | GU684206 | 58.6690 | -94.1600 |
| *Erigone cristatopalpus* | A | 09PROBE-1978-235 | SPICH478-09 | GU684083 | 58.6630 | -94.1670 |
| *Erigone cristatopalpus* | A | 09PROBE-1978-241 | SPICH484-09 | GU684084 | 58.6630 | -94.1670 |
| *Erigone cristatopalpus* | A | 09PROBE-1978-275 | SPICH518-09 | GU684086 | 58.6690 | -94.1600 |
| *Erigone cristatopalpus* | A | 09PROBE-1978-276 | SPICH519-09 | GU684081 | 58.6690 | -94.1600 |
| *Erigone cristatopalpus* | A | 09PROBE-1978-291 | SPICH534-09 | GU684151 | 58.6690 | -94.1600 |
| *Erigone cristatopalpus* | I | 09PROBE-1978-305 | SPICH548-09 | GU684150 | 58.6690 | -94.1600 |
| *Erigone cristatopalpus* | I | 09PROBE-1978-317 | SPICH560-09 | GU684153 | 58.6690 | -94.1600 |
| *Erigone cristatopalpus* | I | 09PROBE-1978-333 | SPICH576-09 | GU684149 | 58.6260 | -94.2300 |
| *Erigone cristatopalpus* | A | 09PROBE-1978-62 | SPICH305-09 | GU684277 | 58.6690 | -94.1600 |
| *Erigone cristatopalpus* | A | 09PROBE-1978-64 | SPICH307-09 | GU684274 | 58.6690 | -94.1600 |
| *Erigone cristatopalpus* | A | 09PROBE-1978-73 | SPICH316-09 | GU684279 | 58.6690 | -94.1600 |
| *Erigone cristatopalpus* | A | 09PROBE-1978-76 | SPICH319-09 | GU684276 | 58.6690 | -94.1600 |
| *Erigone cristatopalpus* | A | 09PROBE-1978-82 | SPICH325-09 | GU684275 | 58.6690 | -94.1600 |
| *Erigone cristatopalpus* | A | 09PROBE-648 | SPICH891-09 | GU684447 | 58.6300 | -93.7980 |
| *Erigone cristatopalpus* | A | 09PROBE-792 | SPICH1013-09 | GU684673 | 58.7380 | -93.8190 |
| *Erigone cristatopalpus* | A | 09PROBE-793 | SPICH1014-09 | GU684671 | 58.7380 | -93.8190 |
| *Erigone cristatopalpus* | A | 09PROBE-794 | SPICH1015-09 | GU684670 | 58.7380 | -93.8190 |
| *Erigone cristatopalpus* | A | 09PROBE-795 | SPICH1016-09 | GU684669 | 58.7380 | -93.8190 |
| *Erigone cristatopalpus* | A | 09PROBE-864 | SPICH1085-09 | GU684630 | 58.6630 | -94.1670 |
| *Erigone cristatopalpus* | I | 09PROBE-865 | SPICH1086-09 | GU684635 | 58.6630 | -94.1670 |
| *Erigone cristatopalpus* | A | 09PROBE-867 | SPICH1088-09 | GU684631 | 58.6630 | -94.1670 |
| *Erigone cristatopalpus* | A | 09PROBE-930 | SPICH1151-09 | GU684632 | 58.6260 | -94.2300 |
| *Erigone cristatopalpus* | A | 09PROBE-931 | SPICH1152-09 | GU684633 | 58.6260 | -94.2300 |
| *Erigone cristatopalpus* | A | 09PROBE-938 | SPICH1159-09 | GU684634 | 58.6260 | -94.2300 |
| *Erigone cristatopalpus* | A | BIOUG00628-F04 | SPIRU1131-11 | KF368033 | 58.7055 | -94.0536 |
| *Erigone cristatopalpus* | A | BIOUG00628-F08 | SPIRU1135-11 | KF368035 | 58.7544 | -93.9980 |
| *Erigone cristatopalpus* | A | BIOUG00628-H11 | SPIRU1162-11 | KF368032 | 58.6716 | -93.8396 |
| *Erigone cristatopalpus* | A | BIOUG00629-B10 | SPIRU1184-11 | KF368031 | 58.6629 | -94.1679 |
| *Erigone cristatopalpus* | A | BIOUG00629-C01 | SPIRU1187-11 | KF368034 | 58.6629 | -94.1676 |
| *Erigone dentigera* | A | BIOUG00627-F05 | SPIRU1037-11 | KF368025 | 58.6329 | -93.7869 |
| *Erigone dentigera* | A | BIOUG00628-H04 | SPIRU1155-11 | KF368027 | 58.7718 | -93.8439 |
| *Erigone dentigera* | I | CHU-SPI-487 | KKCHE690-07 | KF368026 | 58.7540 | -93.9130 |
| *Erigone tirolensis* | I | 07CHU-AR-0093 | KKCHE1005-09 | GU683692 | 58.1280 | -92.8560 |
| *Erigone tirolensis* | I | 07CHU-AR-0095 | KKCHE1007-09 | GU683698 | 58.1280 | -92.8560 |
| *Erigone tirolensis* | A | BIOUG00627-F02 | SPIRU1034-11 | KF368029 | 58.6329 | -93.7869 |
| *Erigone tirolensis* | A | BIOUG00627-F08 | SPIRU1040-11 | KF368030 | 58.6329 | -93.7869 |
| *Erigone tirolensis* | A | BIOUG00630-C02 | SPIRU1283-11 | KF368028 | 58.7710 | -93.8510 |
| *Estrandia grandaeva* | A | 07PROBE-04590 | ERSCH045-07 | KF368042 | 58.7310 | -93.7800 |
| *Estrandia grandaeva* | I | 07PROBE-04593 | ERSCH048-07 | KF368060 | 58.7310 | -93.7800 |
| *Estrandia grandaeva* | A | 07PROBE-04597 | ERSCH052-07 | KF368081 | 58.7310 | -93.7800 |
| *Estrandia grandaeva* | A | 07PROBE-04752 | ERSCH117-07 | KF368055 | 58.7350 | -94.1090 |
| *Estrandia grandaeva* | A | 07PROBE-04784 | ERSCH149-07 | KF368077 | 58.6300 | -93.7980 |
| *Estrandia grandaeva* | I | 09PROBE-01597 | SWSWE027-09 | GU681061 | 58.7300 | -93.7800 |
| *Estrandia grandaeva* | I | 09PROBE-01605 | SWSWE035-09 | GU681055 | 58.6620 | -94.1650 |
| *Estrandia grandaeva* | I | 09PROBE-01609 | SWSWE039-09 | GU681053 | 58.6620 | -94.1650 |
| *Estrandia grandaeva* | I | 09PROBE-01629 | SWSWE059-09 | GU681036 | 58.6190 | -93.8290 |
| *Estrandia grandaeva* | I | 09PROBE-01637 | SWSWE067-09 | GU681030 | 58.6190 | -93.8290 |
| *Estrandia grandaeva* | I | 09-PROBE-08136 | JDTGS040-09 | GU679883 | 58.6250 | -93.8190 |
| *Estrandia grandaeva* | I | 09-PROBE-08156 | JDTGS060-09 | GU679867 | 58.6250 | -93.8190 |
| *Estrandia grandaeva* | I | 09-PROBE-08157 | JDTGS061-09 | GU679835 | 58.6180 | -93.8290 |
| *Estrandia grandaeva* | I | 09-PROBE-08163 | JDTGS067-09 | GU679860 | 58.6180 | -93.8290 |
| *Estrandia grandaeva* | I | 09-PROBE-08172 | JDTGS076-09 | GU679855 | 58.6250 | -93.8190 |
| *Estrandia grandaeva* | I | 09-PROBE-08183 | JDTGS087-09 | GU679848 | 58.6180 | -93.8290 |
| *Estrandia grandaeva* | I | 09-PROBE-08185 | JDTGS089-09 | GU679846 | 58.7380 | -93.8180 |
| *Estrandia grandaeva* | I | 09PROBE-1978-492 | SPICH735-09 | GU684387 | 58.6300 | -94.2300 |
| *Estrandia grandaeva* | I | 09PROBE-641 | SPICH884-09 | GU684457 | 58.7310 | -93.7800 |
| *Estrandia grandaeva* | I | 09PROBE-666 | SPICH909-09 | GU684490 | 58.7310 | -93.7800 |
| *Estrandia grandaeva* | A | 09PROBE-791 | SPICH1012-09 | GU684700 | 58.6300 | -93.7980 |
| *Estrandia grandaeva* | A | 09PROBE-913 | SPICH1134-09 | GU684652 | 58.6300 | -93.7980 |
| *Estrandia grandaeva* | A | 09PROBE-915 | SPICH1136-09 | GU684651 | 58.6300 | -93.7980 |
| *Estrandia grandaeva* | A | 09PROBE-916 | SPICH1137-09 | GU684657 | 58.6300 | -93.7980 |
| *Estrandia grandaeva* | A | 09PROBE-917 | SPICH1138-09 | GU684653 | 58.6300 | -93.7980 |
| *Estrandia grandaeva* | A | 09PROBE-918 | SPICH1139-09 | GU684654 | 58.6300 | -93.7980 |
| *Estrandia grandaeva* | A | 09PROBE-919 | SPICH1140-09 | GU684655 | 58.6300 | -93.7980 |
| *Estrandia grandaeva* | A | 09PROBE-920 | SPICH1141-09 | GU684656 | 58.6300 | -93.7980 |
| *Estrandia grandaeva* | A | 09PROBE-921 | SPICH1142-09 | GU684658 | 58.6300 | -93.7980 |
| *Estrandia grandaeva* | A | 09PROBE-922 | SPICH1143-09 | GU684659 | 58.6300 | -93.7980 |
| *Estrandia grandaeva* | I | 10PROBE-21123 | GBADC003-10 | HQ956667 | 58.7340 | -94.1120 |
| *Estrandia grandaeva* | I | 10PROBE-21127 | GBADC007-10 | HQ956671 | 58.7340 | -94.1120 |
| *Estrandia grandaeva* | I | 10PROBE-21131 | GBADC011-10 | HQ956675 | 58.7340 | -94.1120 |
| *Estrandia grandaeva* | I | 10PROBE-21134 | GBADC014-10 | HQ956678 | 58.7340 | -94.1120 |
| *Estrandia grandaeva* | I | 10PROBE-21140 | GBADC020-10 | HQ956683 | 58.7340 | -94.1120 |
| *Estrandia grandaeva* | I | 10PROBE-21141 | GBADC021-10 | HQ956684 | 58.7340 | -94.1120 |
| *Estrandia grandaeva* | I | 10PROBE-21148 | GBADC028-10 | HQ956691 | 58.7340 | -94.1120 |
| *Estrandia grandaeva* | I | 10PROBE-21154 | GBADC034-10 | HQ956697 | 58.7340 | -94.1120 |
| *Estrandia grandaeva* | A | BIOUG00627-B01 | SPIRU985-11 | KF368058 | 58.7304 | -93.7805 |
| *Estrandia grandaeva* | A | BIOUG00627-F01 | SPIRU1033-11 | KF368082 | 58.6192 | -93.8291 |
| *Estrandia grandaeva* | A | BIOUG00628-G03 | SPIRU1142-11 | KF368083 | 58.6350 | -93.7999 |
| *Estrandia grandaeva* | A | BIOUG00628-G04 | SPIRU1143-11 | KF368071 | 58.6350 | -93.7999 |
| *Estrandia grandaeva* | A | BIOUG00628-G05 | SPIRU1144-11 | KF368046 | 58.6350 | -93.7999 |
| *Estrandia grandaeva* | A | BIOUG00628-G06 | SPIRU1145-11 | KF368050 | 58.6350 | -93.7999 |
| *Estrandia grandaeva* | A | BIOUG00628-G07 | SPIRU1146-11 | KF368036 | 58.6350 | -93.7999 |
| *Estrandia grandaeva* | A | BIOUG00628-G08 | SPIRU1147-11 | KF368065 | 58.6350 | -93.7999 |
| *Estrandia grandaeva* | A | BIOUG00630-F09 | SPIRU1326-11 | KF368063 | 58.7305 | -93.7805 |
| *Estrandia grandaeva* | I | CHU-SPI-001 | KKCHE110-06 | KF368075 | 58.6180 | -93.8230 |
| *Estrandia grandaeva* | A | CHU-SPI-114 | KKCHE317-07 | KF368052 | 58.6220 | -93.8100 |
| *Estrandia grandaeva* | I | CHU-SPI-115 | KKCHE318-07 | KF368037 | 58.6220 | -93.8100 |
| *Estrandia grandaeva* | I | CHU-SPI-116 | KKCHE319-07 | KF368040 | 58.6220 | -93.8100 |
| *Estrandia grandaeva* | I | CHU-SPI-117 | KKCHE320-07 | KF368044 | 58.6220 | -93.8100 |
| *Estrandia grandaeva* | I | CHU-SPI-118 | KKCHE321-07 | KF368072 | 58.6220 | -93.8100 |
| *Estrandia grandaeva* | A | CHU-SPI-120 | KKCHE323-07 | KF368074 | 58.6170 | -93.8210 |
| *Estrandia grandaeva* | A | CHU-SPI-126 | KKCHE329-07 | KF368073 | 58.6180 | -93.8230 |
| *Estrandia grandaeva* | I | CHU-SPI-127 | KKCHE330-07 | KF368061 | 58.6220 | -93.8100 |
| *Estrandia grandaeva* | A | CHU-SPI-128 | KKCHE331-07 | KF368049 | 58.6220 | -93.8100 |
| *Estrandia grandaeva* | I | CHU-SPI-129 | KKCHE332-07 | KF368085 | 58.6220 | -93.8100 |
| *Estrandia grandaeva* | A | CHU-SPI-138 | KKCHE341-07 | KF368048 | 58.6180 | -93.8230 |
| *Estrandia grandaeva* | I | CHU-SPI-139 | KKCHE342-07 | KF368045 | 58.6220 | -93.8100 |
| *Estrandia grandaeva* | A | CHU-SPI-140 | KKCHE343-07 | KF368041 | 58.6220 | -93.8100 |
| *Estrandia grandaeva* | I | CHU-SPI-150 | KKCHE353-07 | KF368087 | 58.6180 | -93.8230 |
| *Estrandia grandaeva* | A | CHU-SPI-152 | KKCHE355-07 | KF368039 | 58.6220 | -93.8100 |
| *Estrandia grandaeva* | I | CHU-SPI-162 | KKCHE365-07 | KF368079 | 58.6180 | -93.8230 |
| *Estrandia grandaeva* | I | CHU-SPI-163 | KKCHE366-07 | KF368066 | 58.6220 | -93.8100 |
| *Estrandia grandaeva* | I | CHU-SPI-174 | KKCHE377-07 | KF368038 | 58.6180 | -93.8230 |
| *Estrandia grandaeva* | I | CHU-SPI-175 | KKCHE378-07 | KF368051 | 58.6220 | -93.8100 |
| *Estrandia grandaeva* | I | CHU-SPI-186 | KKCHE389-07 | KF368070 | 58.6180 | -93.8230 |
| *Estrandia grandaeva* | I | CHU-SPI-187 | KKCHE390-07 | KF368047 | 58.6220 | -93.8100 |
| *Estrandia grandaeva* | I | CHU-SPI-198 | KKCHE401-07 | KF368086 | 58.6180 | -93.8230 |
| *Estrandia grandaeva* | I | CHU-SPI-199 | KKCHE402-07 | KF368064 | 58.6220 | -93.8100 |
| *Estrandia grandaeva* | I | CHU-SPI-200 | KKCHE403-07 | KF368067 | 58.6220 | -93.8100 |
| *Estrandia grandaeva* | I | CHU-SPI-204 | KKCHE407-07 | KF368069 | 58.6220 | -93.8100 |
| *Estrandia grandaeva* | I | CHU-SPI-216 | KKCHE419-07 | KF368080 | 58.6220 | -93.8100 |
| *Estrandia grandaeva* | A | CHU-SPI-228 | KKCHE431-07 | KF368068 | 58.6220 | -93.8100 |
| *Estrandia grandaeva* | I | CHU-SPI-229 | KKCHE432-07 | KF368084 | 58.6220 | -93.8100 |
| *Estrandia grandaeva* | I | CHU-SPI-239 | KKCHE442-07 | KF368076 | 58.6220 | -93.8100 |
| *Estrandia grandaeva* | I | CHU-SPI-240 | KKCHE443-07 | KF368062 | 58.6220 | -93.8100 |
| *Estrandia grandaeva* | I | CHU-SPI-251 | KKCHE454-07 | KF368057 | 58.6220 | -93.8100 |
| *Estrandia grandaeva* | A | CHU-SPI-252 | KKCHE455-07 | KF368054 | 58.6220 | -93.8100 |
| *Estrandia grandaeva* | I | CHU-SPI-255 | KKCHE458-07 | KF368078 | 58.6910 | -94.1790 |
| *Estrandia grandaeva* | I | CHU-SPI-263 | KKCHE466-07 | KF368056 | 58.6220 | -93.8100 |
| *Estrandia grandaeva* | I | CHU-SPI-264 | KKCHE467-07 | KF368043 | 58.6220 | -93.8100 |
| *Estrandia grandaeva* | I | CHU-SPI-275 | KKCHE478-07 | KF368053 | 58.6220 | -93.8100 |
| *Estrandia grandaeva* | I | CHU-SPI-287 | KKCHE490-07 | KF368059 | 58.6220 | -93.8100 |
| *Floricomus rostratus* | A | BIOUG00630-A09 | SPIRU1266-11 | KF368088 | 58.6610 | -93.8320 |
| *Gnaphosa borea* | I | 07CHU-AR-0221 | KKCHE1028-09 | GU683711 | 58.7630 | -93.8850 |
| *Gnaphosa borea* | A | 07CHU-AR-0253 | KKCHE1060-09 | GU683738 | 58.7600 | -94.0860 |
| *Gnaphosa borea* | I | 09-PROBE-08105 | JDTGS009-09 | GU679903 | 58.6250 | -93.8190 |
| *Gnaphosa borea* | I | 09PROBE-1705-01 | SPICH152-09 | GU683782 | 58.6300 | -93.8190 |
| *Gnaphosa borea* | I | 09PROBE-1705-02 | SPICH153-09 | GU683783 | 58.6300 | -93.8190 |
| *Gnaphosa borea* | A | 09PROBE-1978-374 | SPICH617-09 | GU684129 | 58.7640 | -93.8970 |
| *Gnaphosa borea* | A | 09PROBE-1978-479 | SPICH722-09 | GU684403 | 58.7640 | -93.8970 |
| *Gnaphosa borea* | A | BIOUG00629-F09 | SPIRU1231-11 | KF368092 | 58.7720 | -93.8431 |
| *Gnaphosa borea* | A | BIOUG00630-G03 | SPIRU1332-11 | KF368093 | 58.7690 | -93.8620 |
| *Gnaphosa borea* | A | BIOUG00630-G08 | SPIRU1337-11 | KF368090 | 58.6340 | -93.7860 |
| *Gnaphosa borea* | I | CHU-SPI-433 | KKCHE636-07 | KF368091 | 58.7380 | -93.8190 |
| *Gnaphosa borea* | I | SD2707HE603 | SAPIT027-08 | KF368089 | 58.7300 | -93.8000 |
| *Gnaphosa brumalis* | I | 09PROBE-1803-01 | SPICH213-09 | GU683954 | 58.6300 | -93.8190 |
| *Gnaphosa microps* | I | 09PROBE-1706-01 | SPICH155-09 | GU684022 | 58.6300 | -93.8190 |
| *Gnaphosa microps* | I | 09PROBE-1706-02 | SPICH156-09 | GU684021 | 58.6300 | -93.8190 |
| *Gnaphosa microps* | A | 09PROBE-1789-01 | SPICH203-09 | GU683955 | 58.6300 | -93.8190 |
| *Gnaphosa microps* | A | 09PROBE-1802-01 | SPICH212-09 | GU683956 | 58.6300 | -93.8190 |
| *Gnaphosa microps* | I | CHU-SPI-455 | KKCHE658-07 | KF368098 | 58.7380 | -93.8190 |
| *Gnaphosa microps* | I | CHU-SPI-552 | KKCHE755-07 | KF368100 | 58.7470 | -94.1340 |
| *Gnaphosa microps* | I | HLC-26802 | KKCHE833-09 | KF368099 | 58.7800 | -94.1860 |
| *Gnaphosa microps* | I | HLC-26838 | KKCHE869-09 | KF368094 | 58.7800 | -94.1860 |
| *Gnaphosa microps* | I | HLC-26852 | KKCHE883-09 | KF368097 | 58.6200 | -93.7800 |
| *Gnaphosa microps* | I | JBWM0234314 | KKCHE294-07 | KF368095 | 58.6290 | -93.7980 |
| *Gnaphosa microps* | A | SD2707SG212 | SAPIT084-08 | KF368096 | 58.7300 | -93.8000 |
| *Gnaphosa muscorum* | I | 07PROBE-06510 | TWSC024-07 | KF368102 | 58.6170 | -93.8140 |
| *Gnaphosa muscorum* | I | 07PROBE-06511 | TWSC025-07 | KF368101 | 58.6170 | -93.8140 |
| *Gnaphosa muscorum* | I | 09PROBE-1693-01 | SPICH135-09 | GU683784 | 58.6300 | -93.8190 |
| *Gnaphosa muscorum* | I | 09PROBE-1693-02 | SPICH136-09 | GU683785 | 58.6300 | -93.8190 |
| *Gnaphosa muscorum* | I | 09PROBE-1881-01 | SPICH047-09 | GU683906 | 58.7500 | -94.0840 |
| *Gnaphosa orites* | A | BIOUG00630-D05 | SPIRU1298-11 | KF368103 | 58.7305 | -93.7805 |
| *Gnaphosa orites* | I | BIOUG00632-B01 | SPIRU1365-11 | KF368104 | 58.7690 | -93.8620 |
| *Gnaphosa orites* | I | JBWM0005976 | KKCHE228-07 | KF368105 | 58.7310 | -93.7800 |
| *Gnaphosa parvula* | I | 09PROBE-1704-01 | SPICH151-09 | GU683780 | 58.6690 | -94.1600 |
| *Gnaphosa parvula* | I | CHU-SPI-347 | KKCHE510-07 | KF368108 | 58.6320 | -93.7860 |
| *Gnaphosa parvula* | I | HLC-26871 | KKCHE902-09 | KF368106 | 58.7650 | -93.9970 |
| *Gnaphosa parvula* | A | JBWM0005983 | KKCHE229-07 | KF368107 | 58.7540 | -93.9130 |
| *Gonatium crassipalpum* | I | BIOUG00628-A07 | SPIRU1074-11 | KF368109 | 58.6750 | -93.8416 |
| *Gonatium crassipalpum* | A | BIOUG00630-A07 | SPIRU1264-11 | KF368111 | 58.6610 | -93.8320 |
| *Gonatium crassipalpum* | A | BIOUG00630-H11 | SPIRU1352-11 | KF368112 | 58.6610 | -93.8320 |
| *Gonatium crassipalpum* | I | CHU-SPI-193 | KKCHE396-07 | KF368110 | 58.6170 | -93.8210 |
| *Grammonota angusta* | I | 09-PROBE-08147 | JDTGS051-09 | GU679837 | 58.6180 | -93.8290 |
| *Grammonota angusta* | I | BIOUG00628-B08 | SPIRU1087-11 | KF368113 | 58.7340 | -94.1120 |
| *Grammonota gentilis* | I | 09PROBE-1736-01 | SPICH175-09 | GU684010 | 58.7640 | -93.8970 |
| *Grammonota gentilis* | I | 09PROBE-1738-01 | SPICH177-09 | GU684011 | 58.7640 | -93.8970 |
| *Grammonota gentilis* | A | 09PROBE-1916-01 | SPICH068-09 | GU683862 | 58.7630 | -93.8660 |
| *Grammonota gentilis* | A | 09PROBE-1918-01 | SPICH070-09 | GU683829 | 58.7630 | -93.8660 |
| *Grammonota gentilis* | A | 09PROBE-1943-01 | SPICH082-09 | GU683861 | 58.7640 | -93.8970 |
| *Grammonota gentilis* | A | 09PROBE-1978-358 | SPICH601-09 | KF368118 | 58.7640 | -93.8970 |
| *Grammonota gentilis* | I | 09PROBE-1978-361 | SPICH604-09 | GU684157 | 58.7640 | -93.8970 |
| *Grammonota gentilis* | I | 09PROBE-1978-372 | SPICH615-09 | GU684158 | 58.7640 | -93.8970 |
| *Grammonota gentilis* | A | 09PROBE-1978-404 | SPICH647-09 | GU684561 | 58.7640 | -93.8970 |
| *Grammonota gentilis* | A | 09PROBE-1978-406 | SPICH649-09 | GU684562 | 58.7640 | -93.8970 |
| *Grammonota gentilis* | A | 09PROBE-1978-409 | SPICH652-09 | GU684563 | 58.7640 | -93.8970 |
| *Grammonota gentilis* | A | 09PROBE-1978-411 | SPICH654-09 | GU684556 | 58.7640 | -93.8970 |
| *Grammonota gentilis* | A | 09PROBE-1978-412 | SPICH655-09 | GU684564 | 58.7640 | -93.8970 |
| *Grammonota gentilis* | A | 09PROBE-1978-414 | SPICH657-09 | GU684565 | 58.7640 | -93.8970 |
| *Grammonota gentilis* | A | 09PROBE-1978-415 | SPICH658-09 | GU684566 | 58.7640 | -93.8970 |
| *Grammonota gentilis* | I | 09PROBE-1978-416 | SPICH659-09 | KF368125 | 58.7640 | -93.8970 |
| *Grammonota gentilis* | A | 09PROBE-1978-420 | SPICH663-09 | GU684567 | 58.7640 | -93.8970 |
| *Grammonota gentilis* | A | 09PROBE-1978-425 | SPICH668-09 | GU684568 | 58.7640 | -93.8970 |
| *Grammonota gentilis* | A | 09PROBE-1978-426 | SPICH669-09 | GU684559 | 58.7640 | -93.8970 |
| *Grammonota gentilis* | A | 09PROBE-1978-428 | SPICH671-09 | GU684569 | 58.7640 | -93.8970 |
| *Grammonota gentilis* | I | 09PROBE-1978-435 | SPICH678-09 | GU684570 | 58.7640 | -93.8970 |
| *Grammonota gentilis* | I | 09PROBE-1978-436 | SPICH679-09 | GU684571 | 58.7640 | -93.8970 |
| *Grammonota gentilis* | A | 09PROBE-1978-437 | SPICH680-09 | GU684572 | 58.7640 | -93.8970 |
| *Grammonota gentilis* | A | 09PROBE-1978-438 | SPICH681-09 | GU684573 | 58.7640 | -93.8970 |
| *Grammonota gentilis* | A | 09PROBE-1978-445 | SPICH688-09 | GU684574 | 58.7640 | -93.8970 |
| *Grammonota gentilis* | A | 09PROBE-1978-447 | SPICH690-09 | GU684560 | 58.7640 | -93.8970 |
| *Grammonota gentilis* | A | 09PROBE-1978-448 | SPICH691-09 | GU684557 | 58.7640 | -93.8970 |
| *Grammonota gentilis* | A | 09PROBE-1978-454 | SPICH697-09 | GU684575 | 58.7640 | -93.8970 |
| *Grammonota gentilis* | A | 09PROBE-1978-456 | SPICH699-09 | GU684576 | 58.7640 | -93.8970 |
| *Grammonota gentilis* | A | 09PROBE-1978-460 | SPICH703-09 | GU684513 | 58.7640 | -93.8970 |
| *Grammonota gentilis* | A | 09PROBE-1978-463 | SPICH706-09 | GU684577 | 58.7640 | -93.8970 |
| *Grammonota gentilis* | A | 09PROBE-1978-464 | SPICH707-09 | GU684514 | 58.7640 | -93.8970 |
| *Grammonota gentilis* | A | 09PROBE-1978-465 | SPICH708-09 | GU684558 | 58.7640 | -93.8970 |
| *Grammonota gentilis* | A | 09PROBE-1978-470 | SPICH713-09 | GU684515 | 58.7640 | -93.8970 |
| *Grammonota gentilis* | A | 09PROBE-1978-471 | SPICH714-09 | GU684578 | 58.7640 | -93.8970 |
| *Grammonota gentilis* | A | BIOUG00627-H01 | SPIRU1057-11 | KF368123 | 58.7710 | -93.8510 |
| *Grammonota gentilis* | A | BIOUG00629-H09 | SPIRU1255-11 | KF368127 | 58.7710 | -93.8510 |
| *Grammonota gentilis* | A | BIOUG00630-A01 | SPIRU1258-11 | KF368128 | 58.7710 | -93.8510 |
| *Grammonota gentilis* | A | BIOUG00630-A02 | SPIRU1259-11 | KF368120 | 58.7710 | -93.8510 |
| *Grammonota gentilis* | A | BIOUG00630-A03 | SPIRU1260-11 | KF368117 | 58.7710 | -93.8510 |
| *Grammonota gentilis* | A | BIOUG00630-B04 | SPIRU1273-11 | KF368121 | 58.7710 | -93.8510 |
| *Grammonota gentilis* | I | BIOUG00630-B05 | SPIRU1274-11 | KF368116 | 58.7710 | -93.8510 |
| *Grammonota gentilis* | A | BIOUG00630-B06 | SPIRU1275-11 | KF368126 | 58.7710 | -93.8510 |
| *Grammonota gentilis* | A | BIOUG00632-B11 | SPIRU1375-11 | KF368115 | 58.7710 | -93.8510 |
| *Grammonota gentilis* | A | BIOUG00632-C01 | SPIRU1377-11 | KF368122 | 58.7710 | -93.8510 |
| *Grammonota gentilis* | A | BIOUG00632-C05 | SPIRU1381-11 | KF368114 | 58.7710 | -93.8510 |
| *Grammonota gentilis* | A | BIOUG00632-C06 | SPIRU1382-11 | KF368119 | 58.7710 | -93.8510 |
| *Grammonota gentilis* | A | BIOUG00632-C12 | SPIRU1388-11 | KF368124 | 58.7710 | -93.8510 |
| *Grammonota maritima* | A | 07CHU-AR-0007 | KKCHE919-09 | GU683628 | 58.1280 | -92.8560 |
| *Grammonota maritima* | A | 07CHU-AR-0008 | KKCHE920-09 | GU683627 | 58.1280 | -92.8560 |
| *Grammonota maritima* | I | 07CHU-AR-0009 | KKCHE921-09 | GU683630 | 58.1280 | -92.8560 |
| *Grammonota maritima* | A | 07CHU-AR-0010 | KKCHE922-09 | GU683629 | 58.1280 | -92.8560 |
| *Grammonota maritima* | I | 07CHU-AR-0026 | KKCHE938-09 | GU683640 | 58.7600 | -93.9620 |
| *Grammonota maritima* | I | 07CHU-AR-0028 | KKCHE940-09 | GU683643 | 58.7600 | -93.9620 |
| *Grammonota maritima* | I | 07CHU-AR-0029 | KKCHE941-09 | HM862435 | 58.7050 | -94.0540 |
| *Grammonota maritima* | I | 07CHU-AR-0041 | KKCHE953-09 | GU683656 | 58.6620 | -93.1890 |
| *Grammonota maritima* | A | 07CHU-AR-0042 | KKCHE954-09 | GU683655 | 58.6620 | -93.1890 |
| *Grammonota maritima* | I | 07CHU-AR-0043 | KKCHE955-09 | GU683658 | 58.6620 | -93.1890 |
| *Grammonota maritima* | I | 07CHU-AR-0044 | KKCHE956-09 | GU683657 | 58.6620 | -93.1890 |
| *Grammonota maritima* | I | 07CHU-AR-0045 | KKCHE957-09 | GU683660 | 58.6620 | -93.1890 |
| *Grammonota maritima* | A | 07CHU-AR-0058 | KKCHE970-09 | GU683669 | 58.7710 | -93.8430 |
| *Grammonota maritima* | A | 07CHU-AR-0068 | KKCHE980-09 | GU683680 | 58.7660 | -93.8680 |
| *Grammonota maritima* | A | 09PROBE-1942-01 | SPICH081-09 | GU683860 | 58.7640 | -93.8970 |
| *Grammonota maritima* | A | 09PROBE-1978-184 | SPICH427-09 | GU684205 | 58.6260 | -94.2300 |
| *Grammonota maritima* | I | 09PROBE-1978-311 | SPICH554-09 | GU684180 | 58.6690 | -94.1600 |
| *Grammonota maritima* | I | 09PROBE-1978-444 | SPICH687-09 | GU684554 | 58.7640 | -93.8970 |
| *Grammonota maritima* | A | 09PROBE-1978-449 | SPICH692-09 | GU684553 | 58.7640 | -93.8970 |
| *Grammonota maritima* | A | 09PROBE-1978-452 | SPICH695-09 | GU684552 | 58.7640 | -93.8970 |
| *Grammonota maritima* | A | 09PROBE-1978-455 | SPICH698-09 | GU684555 | 58.7640 | -93.8970 |
| *Grammonota maritima* | A | BIOUG00627-E07 | SPIRU1027-11 | KF368131 | 58.7544 | -93.9980 |
| *Grammonota maritima* | A | BIOUG00632-A10 | SPIRU1362-11 | KF368134 | 58.7690 | -93.8620 |
| *Grammonota maritima* | I | CHU-SPI-346 | KKCHE509-07 | KF368130 | 58.6320 | -93.7860 |
| *Grammonota maritima* | I | CHU-SPI-358 | KKCHE533-07 | KF368133 | 58.6320 | -93.7860 |
| *Grammonota maritima* | I | CHU-SPI-385 | KKCHE583-07 | KF368132 | 58.7540 | -93.9490 |
| *Grammonota maritima* | I | SD2707HE504 | SAPIT061-08 | KF368135 | 58.7300 | -93.8000 |
| *Grammonota maritima* | I | SD2707SH601 | SAPIT001-08 | KF368129 | 58.7300 | -93.8000 |
| *Hackmania prominula* | I | 09PROBE-1964-01 | SPICH097-09 | HM432624 | 58.6300 | -93.8190 |
| *Hahnia cinerea* | A | 09PROBE-1661-01 | SPISH017-09 | GU683967 | 58.6300 | -93.7980 |
| *Hahnia cinerea* | A | 09PROBE-1661-02 | SPISH018-09 | GU683966 | 58.6300 | -93.7980 |
| *Hahnia cinerea* | A | 09PROBE-1661-03 | SPISH019-09 | GU683963 | 58.6300 | -93.7980 |
| *Hahnia cinerea* | A | 09PROBE-1661-04 | SPISH020-09 | GU683917 | 58.6300 | -93.7980 |
| *Hahnia cinerea* | A | 09PROBE-1661-05 | SPISH021-09 | GU683964 | 58.6300 | -93.7980 |
| *Hahnia cinerea* | A | 09PROBE-1661-06 | SPISH022-09 | GU683965 | 58.6300 | -93.7980 |
| *Hahnia cinerea* | A | 09PROBE-1661-07 | SPISH023-09 | GU683962 | 58.6300 | -93.7980 |
| *Hahnia cinerea* | A | 09PROBE-1661-08 | SPISH024-09 | GU683968 | 58.6300 | -93.7980 |
| *Hahnia cinerea* | I | 09PROBE-1698-01 | SPICH144-09 | GU684024 | 58.7310 | -93.7800 |
| *Hahnia cinerea* | I | 09PROBE-1750-01 | SPICH184-09 | GU684016 | 58.7310 | -93.7800 |
| *Hahnia cinerea* | A | 09PROBE-1836-01 | SPICH005-09 | GU683826 | 58.6300 | -93.8190 |
| *Hahnia cinerea* | A | 09PROBE-1878-01 | SPICH044-09 | GU683831 | 58.7500 | -94.0840 |
| *Hahnia cinerea* | A | 09PROBE-1978-207 | SPICH450-09 | GU684116 | 58.6300 | -93.7980 |
| *Hahnia cinerea* | A | BIOUG00629-F12 | SPIRU1234-11 | KF368138 | 58.7720 | -93.8431 |
| *Hahnia cinerea* | I | BIOUG00629-G01 | SPIRU1235-11 | KF368136 | 58.7720 | -93.8431 |
| *Hahnia cinerea* | A | BIOUG00629-G02 | SPIRU1236-11 | KF368141 | 58.7720 | -93.8431 |
| *Hahnia cinerea* | A | BIOUG00630-B01 | SPIRU1270-11 | KF368137 | 58.7710 | -93.8510 |
| *Hahnia cinerea* | A | BIOUG00630-H01 | SPIRU1342-11 | KF368139 | 58.6173 | -93.8123 |
| *Hahnia cinerea* | A | BIOUG00630-H02 | SPIRU1343-11 | KF368140 | 58.6173 | -93.8123 |
| *Haplodrassus hiemalis* | A | 07PROBE-06503 | TWSC017-07 | KF368143 | 58.7370 | -93.8230 |
| *Haplodrassus hiemalis* | A | 07PROBE-06506 | TWSC020-07 | KF368145 | 58.7050 | -94.0520 |
| *Haplodrassus hiemalis* | I | 09PROBE-1971-01 | SPISH036-09 | GU683958 | 58.6260 | -94.2300 |
| *Haplodrassus hiemalis* | I | CHU-SPI-495 | KKCHE698-07 | KF368146 | 58.7470 | -94.1340 |
| *Haplodrassus hiemalis* | I | CHU-SPI-499 | KKCHE702-07 | KF368144 | 58.7380 | -93.8190 |
| *Haplodrassus hiemalis* | A | CHU-SPI-581 | KKCHE784-07 | KF368142 | 58.7380 | -93.8190 |
| *Haplodrassus signifer* | A | 09PROBE-1653-01 | SPISH010-09 | GU683960 | 58.6180 | -93.8290 |
| *Haplodrassus signifer* | I | 09PROBE-1699-01 | SPICH145-09 | GU683772 | 58.6180 | -93.8290 |
| *Haplodrassus signifer* | I | 09PROBE-1703-01 | SPICH149-09 | GU683770 | 58.6180 | -93.8290 |
| *Haplodrassus signifer* | I | 09PROBE-1703-02 | SPICH150-09 | GU683773 | 58.6180 | -93.8290 |
| *Haplodrassus signifer* | A | 09PROBE-1805-01 | SPICH214-09 | GU683959 | 58.6300 | -93.8190 |
| *Haplodrassus signifer* | A | JBWM0005984 | KKCHE230-07 | KF368147 | 58.6260 | -94.2280 |
| *Hilaira canaliculata* | I | 09PROBE-1853-01 | SPICH022-09 | HM432622 | 58.6300 | -93.8190 |
| *Hilaira canaliculata* | A | BIOUG00627-F06 | SPIRU1038-11 | KF368148 | 58.6329 | -93.7869 |
| *Horcotes quadricristatus* | I | 09PROBE-1978-550 | SPICH793-09 | HM416914 | 58.7870 | -93.7140 |
| *Horcotes quadricristatus* | A | BIOUG00629-H11 | SPIRU1257-11 | KF368149 | 58.7710 | -93.8510 |
| *Horcotes quadricristatus* | A | BIOUG00630-B02 | SPIRU1271-11 | KF368150 | 58.7710 | -93.8510 |
| *Horcotes quadricristatus* | A | BIOUG00630-H06 | SPIRU1347-11 | KF368151 | 58.6300 | -93.8190 |
| *Hybauchenidium gibbosum* | A | 07CHU-AR-0014 | KKCHE926-09 | GU683631 | 58.6300 | -93.7980 |
| *Hybauchenidium gibbosum* | A | 07CHU-AR-0015 | KKCHE927-09 | GU683635 | 58.6300 | -93.7980 |
| *Hybauchenidium gibbosum* | I | 07CHU-AR-0077 | KKCHE989-09 | GU683681 | 58.7550 | -93.9990 |
| *Hybauchenidium gibbosum* | I | 07CHU-AR-0078 | KKCHE990-09 | GU683683 | 58.7550 | -93.9990 |
| *Hybauchenidium gibbosum* | I | 07CHU-AR-0079 | KKCHE991-09 | GU683682 | 58.7550 | -93.9990 |
| *Hybauchenidium gibbosum* | A | 07CHU-AR-0080 | KKCHE992-09 | GU683686 | 58.7550 | -93.9990 |
| *Hybauchenidium gibbosum* | I | 07CHU-AR-0081 | KKCHE993-09 | GU683685 | 58.7550 | -93.9990 |
| *Hybauchenidium gibbosum* | I | 07CHU-AR-0082 | KKCHE994-09 | GU683684 | 58.7550 | -93.9990 |
| *Hybauchenidium gibbosum* | I | 07CHU-AR-0083 | KKCHE995-09 | GU683689 | 58.7550 | -93.9990 |
| *Hybauchenidium gibbosum* | I | 07CHU-AR-0090 | KKCHE1002-09 | GU683694 | 58.7550 | -93.9990 |
| *Hybauchenidium gibbosum* | A | 07CHU-AR-0091 | KKCHE1003-09 | GU683693 | 58.7550 | -93.9990 |
| *Hybauchenidium gibbosum* | I | 09PROBE-1852-01 | SPICH021-09 | GU683885 | 58.6300 | -93.8190 |
| *Hybauchenidium gibbosum* | A | 09PROBE-1974-01 | SPISH039-09 | GU683982 | 58.6260 | -94.2300 |
| *Hybauchenidium gibbosum* | A | 09PROBE-1975-01 | SPISH040-09 | GU683984 | 58.6260 | -94.2300 |
| *Hybauchenidium gibbosum* | A | 09PROBE-1976-01 | SPISH041-09 | GU683981 | 58.6260 | -94.2300 |
| *Hybauchenidium gibbosum* | A | 09PROBE-1977-01 | SPISH042-09 | GU683983 | 58.6260 | -94.2300 |
| *Hybauchenidium gibbosum* | A | 09PROBE-1978-175 | SPICH418-09 | GU684198 | 58.6260 | -94.2300 |
| *Hybauchenidium gibbosum* | I | 09PROBE-1978-341 | SPICH584-09 | KF368153 | 58.6260 | -94.2300 |
| *Hybauchenidium gibbosum* | A | 09PROBE-746 | SPICH989-09 | GU684472 | 58.6600 | -94.1700 |
| *Hybauchenidium gibbosum* | A | 09PROBE-763 | SPISH045-09 | GU684473 | 58.7310 | -93.7800 |
| *Hybauchenidium gibbosum* | I | JBWM0006086 | KKCHE261-07 | KF368152 | 58.6260 | -94.2280 |
| *Hypomma marxi* | A | 09PROBE-1978-07 | SPICH250-09 | GU684287 | 58.6690 | -94.1600 |
| *Hypomma marxi* | A | 09PROBE-1978-08 | SPICH251-09 | GU684284 | 58.6690 | -94.1600 |
| *Hypomma marxi* | A | 09PROBE-1978-10 | SPICH253-09 | GU684286 | 58.6690 | -94.1600 |
| *Hypomma marxi* | A | 09PROBE-1978-238 | SPICH481-09 | GU684082 | 58.6630 | -94.1670 |
| *Hypomma marxi* | A | 09PROBE-1978-348 | SPICH591-09 | GU684161 | 58.7610 | -93.9540 |
| *Hypomma marxi* | A | 09PROBE-1978-349 | SPICH592-09 | GU684176 | 58.7610 | -93.9540 |
| *Hypomma marxi* | A | 09PROBE-1978-350 | SPICH593-09 | GU684159 | 58.7610 | -93.9540 |
| *Hypomma marxi* | A | 09PROBE-1978-56 | SPICH299-09 | GU684346 | 58.6690 | -94.1600 |
| *Hypomma marxi* | A | 09PROBE-849 | SPICH1070-09 | GU684696 | 58.6630 | -94.1670 |
| *Hypomma marxi* | A | 09PROBE-866 | SPICH1087-09 | GU684619 | 58.6630 | -94.1670 |
| *Hypomma marxi* | A | 09PROBE-871 | SPICH1092-09 | GU684620 | 58.6630 | -94.1670 |
| *Hypomma marxi* | A | 09PROBE-872 | SPICH1093-09 | GU684618 | 58.6630 | -94.1670 |
| *Hypomma marxi* | A | 09PROBE-932 | SPICH1153-09 | GU684622 | 58.6260 | -94.2300 |
| *Hypomma marxi* | A | 09PROBE-934 | SPICH1155-09 | GU684621 | 58.6260 | -94.2300 |
| *Hypomma marxi* | A | 09PROBE-936 | SPICH1157-09 | GU684623 | 58.6260 | -94.2300 |
| *Hypomma marxi* | A | BIOUG00628-D08 | SPIRU1111-11 | KF368154 | 58.7055 | -94.0536 |
| *Hypselistes semiflavus* | A | 09PROBE-1978-18 | SPICH261-09 | GU684278 | 58.6690 | -94.1600 |
| *Hypselistes semiflavus* | A | 09PROBE-1978-289 | SPICH532-09 | GU684154 | 58.6690 | -94.1600 |
| *Hypselistes semiflavus* | I | BIOUG00628-A02 | SPIRU1069-11 | KF368157 | 58.6750 | -93.8416 |
| *Hypselistes semiflavus* | I | BIOUG00628-A03 | SPIRU1070-11 | KF368156 | 58.6750 | -93.8416 |
| *Hypselistes semiflavus* | A | BIOUG00629-A06 | SPIRU1168-11 | KF368155 | 58.7306 | -93.7804 |
| *Hypsosinga pygmaea* | I | 08BBARAC-0159 | ARSO283-08 | KF408103 | 51.1710 | -115.5860 |
| *Hypsosinga pygmaea* | A | 08SOAR-0003 | ARSO003-08 | KF408106 | 45.1600 | -81.5700 |
| *Hypsosinga pygmaea* | A | 08SOAR-0144 | ARSO144-08 | KF408100 | 44.3800 | -80.5700 |
| *Hypsosinga pygmaea* | I | 10PROBE-21155 | GBADC035-10 | HQ956698 | 58.7340 | -94.1120 |
| *Hypsosinga pygmaea* | I | 10PROBE-21168 | GBADC048-10 | HQ956709 | 58.7340 | -94.1120 |
| *Hypsosinga pygmaea* | I | 10PROBE-21215 | GBADC095-10 | HQ956752 | 58.7180 | -94.1240 |
| *Hypsosinga pygmaea* | A | BIOUG00518-E05 | SPICA1564-10 | JF885143 | 52.9157 | -118.1040 |
| *Hypsosinga pygmaea* | I | BIOUG00627-D02 | SPIRU1010-11 | KF368166 | 58.7544 | -93.9980 |
| *Hypsosinga pygmaea* | A | BIOUG00627-D07 | SPIRU1015-11 | KF368164 | 58.7544 | -93.9980 |
| *Hypsosinga pygmaea* | A | BIOUG00627-D08 | SPIRU1016-11 | KF368162 | 58.7544 | -93.9980 |
| *Hypsosinga pygmaea* | A | BIOUG00628-A04 | SPIRU1071-11 | KF368159 | 58.6750 | -93.8416 |
| *Hypsosinga pygmaea* | A | BIOUG00628-A05 | SPIRU1072-11 | KF368158 | 58.6750 | -93.8416 |
| *Hypsosinga pygmaea* | A | BIOUG00628-A08 | SPIRU1075-11 | KF368160 | 58.6329 | -93.7869 |
| *Hypsosinga pygmaea* | A | BIOUG00628-B03 | SPIRU1082-11 | KF368167 | 58.7544 | -93.9980 |
| *Hypsosinga pygmaea* | A | BIOUG00628-C07 | SPIRU1098-11 | KF368161 | 58.6329 | -93.7869 |
| *Hypsosinga pygmaea* | A | BIOUG00628-C08 | SPIRU1099-11 | KF368163 | 58.6329 | -93.7869 |
| *Hypsosinga pygmaea* | I | CCDB-04290-C02 | ARONT502-10 | HQ924396 | 43.5354 | -80.2052 |
| *Hypsosinga pygmaea* | I | CCDB-04290-C03 | ARONT503-10 | HQ924397 | 43.5354 | -80.2052 |
| *Hypsosinga pygmaea* | I | CCDB-04290-C04 | ARONT504-10 | HQ924398 | 43.5354 | -80.2052 |
| *Hypsosinga pygmaea* | I | CCDB-04290-C05 | ARONT505-10 | HQ924399 | 43.5354 | -80.2052 |
| *Hypsosinga pygmaea* | I | SD0406SG403 | SAPIT178-08 | KF368165 | 58.7300 | -93.8000 |
| *Improphantes complicatus* | I | 09-PROBE-08146 | JDTGS050-09 | GU679875 | 58.7380 | -93.8190 |
| *Improphantes complicatus* | I | 09PROBE-1882-01 | SPICH048-09 | GU683846 | 58.7500 | -94.0840 |
| *Improphantes complicatus* | I | 09PROBE-1978-186 | SPICH429-09 | GU684236 | 58.6260 | -94.2300 |
| *Improphantes complicatus* | A | 09PROBE-1978-191 | SPICH434-09 | GU684085 | 58.6300 | -93.7980 |
| *Improphantes complicatus* | A | 09PROBE-1978-220 | SPICH463-09 | GU684087 | 58.6300 | -93.7980 |
| *Improphantes complicatus* | A | 09PROBE-1978-226 | SPICH469-09 | GU684088 | 58.6300 | -93.7980 |
| *Improphantes complicatus* | A | 09PROBE-1978-230 | SPICH473-09 | GU684090 | 58.6300 | -93.7980 |
| *Improphantes complicatus* | A | 09PROBE-575 | SPICH818-09 | GU684435 | 58.7610 | -93.9540 |
| *Improphantes complicatus* | A | 09PROBE-576 | SPICH819-09 | GU684434 | 58.7610 | -93.9540 |
| *Improphantes complicatus* | A | 09PROBE-577 | SPICH820-09 | GU684436 | 58.7610 | -93.9540 |
| *Improphantes complicatus* | A | 09PROBE-578 | SPICH821-09 | GU684433 | 58.7610 | -93.9540 |
| *Improphantes complicatus* | A | 09PROBE-579 | SPICH822-09 | GU684438 | 58.7610 | -93.9540 |
| *Improphantes complicatus* | A | 09PROBE-628 | SPICH871-09 | GU684437 | 58.7900 | -94.2270 |
| *Improphantes complicatus* | I | 09PROBE-890 | SPICH1111-09 | GU684614 | 58.6260 | -94.2300 |
| *Improphantes complicatus* | I | 09PROBE-891 | SPICH1112-09 | GU684616 | 58.6260 | -94.2300 |
| *Improphantes complicatus* | A | 09PROBE-894 | SPICH1115-09 | GU684612 | 58.6260 | -94.2300 |
| *Improphantes complicatus* | A | 09PROBE-895 | SPICH1116-09 | GU684617 | 58.6260 | -94.2300 |
| *Improphantes complicatus* | A | 09PROBE-896 | SPICH1117-09 | GU684615 | 58.6260 | -94.2300 |
| *Improphantes complicatus* | A | 09PROBE-900 | SPICH1121-09 | GU684613 | 58.6260 | -94.2300 |
| *Improphantes complicatus* | I | BIOUG00630-G10 | SPIRU1339-11 | KF368169 | 58.6173 | -93.8123 |
| *Improphantes complicatus* | A | BIOUG00630-H05 | SPIRU1346-11 | KF368173 | 58.7920 | -93.7510 |
| *Improphantes complicatus* | A | BIOUG00630-H08 | SPIRU1349-11 | KF368170 | 58.6300 | -93.8190 |
| *Improphantes complicatus* | A | BIOUG00632-A04 | SPIRU1356-11 | KF368172 | 58.6300 | -93.8190 |
| *Improphantes complicatus* | I | BIOUG00632-A05 | SPIRU1357-11 | KF368174 | 58.6300 | -93.8190 |
| *Improphantes complicatus* | A | BIOUG00632-A06 | SPIRU1358-11 | KF368171 | 58.6300 | -93.8190 |
| *Improphantes complicatus* | A | BIOUG00632-A07 | SPIRU1359-11 | KF368168 | 58.6173 | -93.8123 |
| *Incestophantes washingtoni* | I | 07CHU-AR-0027 | KKCHE939-09 | GU683644 | 58.7600 | -93.9620 |
| *Incestophantes washingtoni* | I | 07CHU-AR-0040 | KKCHE952-09 | GU683653 | 58.6620 | -93.1890 |
| *Incestophantes washingtoni* | I | 09PROBE-1810-01 | SPICH222-09 | GU683969 | 58.6300 | -93.8190 |
| *Incestophantes washingtoni* | A | 09PROBE-1978-109 | SPICH352-09 | GU684253 | 58.6690 | -94.1600 |
| *Incestophantes washingtoni* | A | 09PROBE-1978-126 | SPICH369-09 | GU684254 | 58.6690 | -94.1600 |
| *Incestophantes washingtoni* | I | 09PROBE-1978-131 | SPICH374-09 | GU684251 | 58.6690 | -94.1600 |
| *Incestophantes washingtoni* | A | 09PROBE-1978-30 | SPICH273-09 | GU684324 | 58.6690 | -94.1600 |
| *Incestophantes washingtoni* | I | 09PROBE-1978-46 | SPICH289-09 | GU684327 | 58.6690 | -94.1600 |
| *Incestophantes washingtoni* | A | 09PROBE-1978-47 | SPICH290-09 | GU684325 | 58.6690 | -94.1600 |
| *Incestophantes washingtoni* | I | 09PROBE-1978-86 | SPICH329-09 | GU684322 | 58.6690 | -94.1600 |
| *Incestophantes washingtoni* | A | 09PROBE-753 | SPICH996-09 | GU684465 | 58.6180 | -93.8300 |
| *Incestophantes washingtoni* | A | BIOUG00629-G04 | SPIRU1238-11 | KF368175 | 58.7720 | -93.8431 |
| *Incestophantes washingtoni* | I | CHU-SPI-256 | KKCHE459-07 | KF368176 | 58.6910 | -94.1790 |
| *Islandiana falsifica* | A | 09PROBE-1978-278 | SPICH521-09 | GU684070 | 58.6690 | -94.1600 |
| *Islandiana falsifica* | A | 09PROBE-1978-418 | SPICH661-09 | GU684536 | 58.7640 | -93.8970 |
| *Islandiana falsifica* | A | 09PROBE-1978-501 | SPICH744-09 | GU684385 | 58.7890 | -93.7090 |
| *Islandiana falsifica* | A | 09PROBE-1978-518 | SPICH761-09 | GU684386 | 58.7960 | -93.7540 |
| *Islandiana falsifica* | I | 09PROBE-654 | SPICH897-09 | GU684415 | 58.7870 | -93.7140 |
| *Islandiana falsifica* | A | 09PROBE-725 | SPICH968-09 | GU684488 | 58.7900 | -94.2270 |
| *Islandiana falsifica* | A | 09PROBE-726 | SPICH969-09 | GU684486 | 58.7900 | -94.2270 |
| *Islandiana falsifica* | I | 09PROBE-727 | SPICH970-09 | GU684487 | 58.7900 | -94.2270 |
| *Islandiana falsifica* | A | 09PROBE-728 | SPICH971-09 | GU684485 | 58.7900 | -94.2270 |
| *Islandiana falsifica* | A | 09PROBE-729 | SPICH972-09 | GU684484 | 58.7900 | -94.2270 |
| *Islandiana falsifica* | A | 09PROBE-731 | SPICH974-09 | GU684483 | 58.7900 | -94.2270 |
| *Islandiana holmi* | A | 09PROBE-1776-01 | SPICH198-09 | GU684002 | 58.6300 | -93.8190 |
| *Islandiana holmi* | A | 09PROBE-1779-01 | SPICH201-09 | GU683976 | 58.6300 | -93.8190 |
| *Islandiana holmi* | A | 09PROBE-1795-01 | SPICH206-09 | GU683977 | 58.7610 | -93.9540 |
| *Islandiana holmi* | A | 09PROBE-1834-01 | SPICH003-09 | GU683875 | 58.6300 | -93.8190 |
| *Islandiana holmi* | I | 09PROBE-1883-01 | SPICH049-09 | GU683872 | 58.7500 | -94.0840 |
| *Islandiana holmi* | A | 09PROBE-1884-01 | SPICH050-09 | GU683873 | 58.7500 | -94.0840 |
| *Islandiana holmi* | A | 09PROBE-1886-01 | SPICH052-09 | GU683874 | 58.7500 | -94.0840 |
| *Islandiana holmi* | A | 09PROBE-1978-11 | SPICH254-09 | GU684283 | 58.6690 | -94.1600 |
| *Islandiana holmi* | A | 09PROBE-1978-26 | SPICH269-09 | GU684280 | 58.6690 | -94.1600 |
| *Islandiana holmi* | A | 09PROBE-1978-277 | SPICH520-09 | GU684073 | 58.6690 | -94.1600 |
| *Islandiana holmi* | A | 09PROBE-1978-292 | SPICH535-09 | GU684182 | 58.6690 | -94.1600 |
| *Islandiana holmi* | I | 09PROBE-1978-295 | SPICH538-09 | GU684132 | 58.6690 | -94.1600 |
| *Islandiana holmi* | A | 09PROBE-1978-296 | SPICH539-09 | GU684135 | 58.6690 | -94.1600 |
| *Islandiana holmi* | A | 09PROBE-1978-300 | SPICH543-09 | GU684133 | 58.6690 | -94.1600 |
| *Islandiana holmi* | A | 09PROBE-1978-301 | SPICH544-09 | GU684136 | 58.6690 | -94.1600 |
| *Islandiana holmi* | I | 09PROBE-1978-302 | SPICH545-09 | GU684131 | 58.6690 | -94.1600 |
| *Islandiana holmi* | I | 09PROBE-1978-309 | SPICH552-09 | GU684134 | 58.6690 | -94.1600 |
| *Islandiana holmi* | A | 09PROBE-1978-369 | SPICH612-09 | GU684130 | 58.7640 | -93.8970 |
| *Islandiana holmi* | A | 09PROBE-1978-466 | SPICH709-09 | GU684537 | 58.7640 | -93.8970 |
| *Islandiana holmi* | A | 09PROBE-1978-500 | SPICH743-09 | GU684351 | 58.7890 | -93.7090 |
| *Islandiana holmi* | A | 09PROBE-1978-503 | SPICH746-09 | GU684382 | 58.7890 | -93.7090 |
| *Islandiana holmi* | A | 09PROBE-1978-504 | SPICH747-09 | GU684383 | 58.7890 | -93.7090 |
| *Islandiana holmi* | A | 09PROBE-1978-507 | SPICH750-09 | GU684378 | 58.7960 | -93.7540 |
| *Islandiana holmi* | A | 09PROBE-1978-513 | SPICH756-09 | GU684384 | 58.7960 | -93.7540 |
| *Islandiana holmi* | A | 09PROBE-1978-529 | SPICH772-09 | GU684379 | 58.7960 | -93.7540 |
| *Islandiana holmi* | A | 09PROBE-1978-538 | SPICH781-09 | GU684380 | 58.7960 | -93.7540 |
| *Islandiana holmi* | I | 09PROBE-1978-552 | SPICH795-09 | GU684381 | 58.7870 | -93.7140 |
| *Islandiana holmi* | A | 09PROBE-1978-57 | SPICH300-09 | GU684285 | 58.6690 | -94.1600 |
| *Islandiana holmi* | A | 09PROBE-1978-61 | SPICH304-09 | GU684281 | 58.6690 | -94.1600 |
| *Islandiana holmi* | I | 09PROBE-1978-89 | SPICH332-09 | GU684282 | 58.6690 | -94.1600 |
| *Islandiana holmi* | A | 09PROBE-610 | SPICH853-09 | GU684453 | 58.7900 | -94.2270 |
| *Islandiana holmi* | A | 09PROBE-611 | SPICH854-09 | GU684409 | 58.7900 | -94.2270 |
| *Islandiana holmi* | I | 09PROBE-681 | SPICH924-09 | GU684477 | 58.7960 | -93.7540 |
| *Islandiana holmi* | A | 09PROBE-682 | SPICH925-09 | GU684476 | 58.7960 | -93.7540 |
| *Islandiana holmi* | A | 09PROBE-683 | SPICH926-09 | GU684481 | 58.7960 | -93.7540 |
| *Islandiana holmi* | A | 09PROBE-684 | SPICH927-09 | GU684475 | 58.7960 | -93.7540 |
| *Islandiana holmi* | A | 09PROBE-685 | SPICH928-09 | GU684480 | 58.7960 | -93.7540 |
| *Islandiana holmi* | A | 09PROBE-686 | SPICH929-09 | GU684482 | 58.7960 | -93.7540 |
| *Islandiana holmi* | A | 09PROBE-730 | SPICH973-09 | GU684478 | 58.7900 | -94.2270 |
| *Islandiana holmi* | A | 09PROBE-733 | SPICH976-09 | GU684479 | 58.7900 | -94.2270 |
| *Islandiana holmi* | A | 09PROBE-869 | SPICH1090-09 | GU684636 | 58.6630 | -94.1670 |
| *Islandiana holmi* | A | BIOUG00630-D04 | SPIRU1297-11 | KF368177 | 58.7305 | -93.7805 |
| *Kaestneria pullata* | A | 09PROBE-1978-05 | SPICH248-09 | GU684343 | 58.6690 | -94.1600 |
| *Kaestneria pullata* | A | 09PROBE-1978-107 | SPICH350-09 | GU684269 | 58.6690 | -94.1600 |
| *Kaestneria pullata* | I | 09PROBE-1978-137 | SPICH380-09 | GU684266 | 58.6690 | -94.1600 |
| *Kaestneria pullata* | A | 09PROBE-1978-237 | SPICH480-09 | GU684044 | 58.6630 | -94.1670 |
| *Kaestneria pullata* | A | 09PROBE-1978-240 | SPICH483-09 | GU684041 | 58.6630 | -94.1670 |
| *Kaestneria pullata* | A | 09PROBE-1978-246 | SPICH489-09 | GU684042 | 58.6630 | -94.1670 |
| *Kaestneria pullata* | A | 09PROBE-1978-248 | SPICH491-09 | GU684046 | 58.6630 | -94.1670 |
| *Kaestneria pullata* | I | 09PROBE-1978-253 | SPICH496-09 | GU684043 | 58.6630 | -94.1670 |
| *Kaestneria pullata* | I | 09PROBE-1978-260 | SPICH503-09 | GU684039 | 58.6630 | -94.1670 |
| *Kaestneria pullata* | A | 09PROBE-1978-28 | SPICH271-09 | GU684340 | 58.6690 | -94.1600 |
| *Kaestneria pullata* | A | 09PROBE-1978-287 | SPICH530-09 | GU683994 | 58.6690 | -94.1600 |
| *Kaestneria pullata* | A | 09PROBE-1978-29 | SPICH272-09 | GU684341 | 58.6690 | -94.1600 |
| *Kaestneria pullata* | A | 09PROBE-1978-338 | SPICH581-09 | GU683993 | 58.6260 | -94.2300 |
| *Kaestneria pullata* | A | 09PROBE-1978-60 | SPICH303-09 | GU684342 | 58.6690 | -94.1600 |
| *Kaestneria pullata* | A | 09PROBE-863 | SPICH1084-09 | GU684646 | 58.6630 | -94.1670 |
| *Kaestneria pullata* | A | 09PROBE-875 | SPICH1096-09 | GU684647 | 58.6630 | -94.1670 |
| *Kaestneria pullata* | A | 09PROBE-878 | SPICH1099-09 | GU684649 | 58.6630 | -94.1670 |
| *Kaestneria pullata* | A | 09PROBE-881 | SPICH1102-09 | GU684648 | 58.6630 | -94.1670 |
| *Kaestneria pullata* | A | 09PROBE-885 | SPICH1106-09 | GU684650 | 58.6630 | -94.1670 |
| *Kaestneria pullata* | I | BIOUG00627-C01 | SPIRU997-11 | KF368179 | 58.7920 | -93.7510 |
| *Kaestneria pullata* | A | BIOUG00627-F04 | SPIRU1036-11 | KF368178 | 58.6329 | -93.7869 |
| *Kaestneria rufula* | I | 09PROBE-1978-331 | SPICH574-09 | GU684179 | 58.6690 | -94.1600 |
| *Kaestneria rufula* | A | 09PROBE-642 | SPICH885-09 | GU684458 | 58.7310 | -93.7800 |
| *Kaestneria rufula* | A | BIOUG00627-A01 | SPIRU973-11 | KF368182 | 58.6340 | -93.7860 |
| *Kaestneria rufula* | A | BIOUG00629-D05 | SPIRU1203-11 | KF368183 | 58.6340 | -93.7860 |
| *Kaestneria rufula* | A | BIOUG00630-F06 | SPIRU1323-11 | KF368180 | 58.7305 | -93.7805 |
| *Kaestneria rufula* | A | BIOUG00630-F07 | SPIRU1324-11 | KF368181 | 58.7305 | -93.7805 |
| *Larinioides cornutus* | I | 09PROBE-01604 | SWSWE034-09 | GU681058 | 58.6260 | -94.2290 |
| *Larinioides cornutus* | I | 09PROBE-01606 | SWSWE036-09 | GU681056 | 58.6260 | -94.2290 |
| *Larinioides cornutus* | I | 09PROBE-01612 | SWSWE042-09 | GU681052 | 58.6260 | -94.2290 |
| *Larinioides cornutus* | I | 09PROBE-01613 | SWSWE043-09 | GU681050 | 58.6260 | -94.2290 |
| *Larinioides cornutus* | I | 09PROBE-01614 | SWSWE044-09 | GU681051 | 58.7730 | -93.8390 |
| *Larinioides cornutus* | I | 09PROBE-01648 | SWSWE078-09 | GU681019 | 58.6300 | -93.8190 |
| *Larinioides cornutus* | I | 09PROBE-01649 | SWSWE079-09 | GU681020 | 58.7520 | -93.9170 |
| *Larinioides cornutus* | I | 09PROBE-01650 | SWSWE080-09 | GU681021 | 58.7520 | -93.9170 |
| *Larinioides cornutus* | A | 09PROBE-1669-01 | SPISH027-09 | GU683921 | 58.7610 | -94.0080 |
| *Larinioides cornutus* | A | 09PROBE-1690-01 | SPICH129-09 | GU684017 | 58.7310 | -93.7800 |
| *Larinioides cornutus* | A | 09PROBE-1978-352 | SPICH595-09 | GU684128 | 58.7610 | -93.9540 |
| *Larinioides cornutus* | A | 09PROBE-1978-481 | SPICH724-09 | GU684356 | 58.6300 | -94.2300 |
| *Larinioides cornutus* | I | 09PROBE-1978-490 | SPICH733-09 | GU684357 | 58.6300 | -94.2300 |
| *Larinioides cornutus* | A | 09PROBE-835 | SPICH1056-09 | GU684718 | 58.7730 | -93.8390 |
| *Larinioides cornutus* | I | 10PROBE-21173 | GBADC053-10 | HQ956714 | 58.6340 | -93.7860 |
| *Larinioides cornutus* | I | 10PROBE-21175 | GBADC055-10 | HQ956716 | 58.6340 | -93.7860 |
| *Larinioides cornutus* | I | 10PROBE-21178 | GBADC058-10 | HQ956719 | 58.6340 | -93.7860 |
| *Larinioides cornutus* | I | 10PROBE-21187 | GBADC067-10 | HQ956727 | 58.6610 | -93.8320 |
| *Larinioides cornutus* | I | 10PROBE-21190 | GBADC070-10 | HQ956730 | 58.6610 | -93.8320 |
| *Larinioides cornutus* | I | 10PROBE-21193 | GBADC073-10 | HQ956733 | 58.6610 | -93.8320 |
| *Larinioides cornutus* | I | 10PROBE-21195 | GBADC075-10 | HQ956735 | 58.6610 | -93.8320 |
| *Larinioides cornutus* | I | 10PROBE-21196 | GBADC076-10 | HQ956736 | 58.6610 | -93.8320 |
| *Larinioides cornutus* | I | 10PROBE-21197 | GBADC077-10 | HQ956737 | 58.6610 | -93.8320 |
| *Larinioides cornutus* | I | 10PROBE-21201 | GBADC081-10 | HQ956741 | 58.7180 | -94.1240 |
| *Larinioides cornutus* | I | 10PROBE-21205 | GBADC085-10 | HQ956744 | 58.7180 | -94.1240 |
| *Larinioides cornutus* | A | BIOUG00041-A11 | SPIEU834-10 | HQ975384 | 52.1704 | 22.2784 |
| *Larinioides cornutus* | I | BIOUG00518-E10 | SPICA1569-10 | JF885148 | 53.7301 | -106.7220 |
| *Larinioides cornutus* | A | BIOUG00519-G01 | SPICA1679-10 | JF885258 | 53.6842 | -112.8630 |
| *Larinioides cornutus* | A | BIOUG00608-E01 | SPIRU861-11 | JF886981 | 59.2500 | 163.0670 |
| *Larinioides cornutus* | A | BIOUG00624-C09 | PPELE285-11 | JN308502 | 41.9360 | -82.5160 |
| *Larinioides cornutus* | I | BIOUG00627-G01 | SPIRU1045-11 | KF368193 | 58.7713 | -93.8509 |
| *Larinioides cornutus* | A | BIOUG00628-C01 | SPIRU1092-11 | KF368188 | 58.6329 | -93.7869 |
| *Larinioides cornutus* | A | BIOUG00890-A02 | BBUSE097-11 | KF408096 | 42.9340 | 22.9540 |
| *Larinioides cornutus* | I | BIOUG01960-H11 | BBUSE2368-12 | KF408099 | 29.3810 | -95.5950 |
| *Larinioides cornutus* | A | BIOUG01961-A09 | BBUSE2377-12 | KF408105 | 41.8640 | -83.4040 |
| *Larinioides cornutus* | I | CCDB-04551-E12 | BBCAN345-09 | GU683051 | 46.7420 | -60.8270 |
| *Larinioides cornutus* | I | CCDB-05302-G01 | BBCAN822-09 | GU683000 | 46.8270 | -64.9150 |
| *Larinioides cornutus* | A | CCDB-08491-B05 | TURAR646-10 | HQ982830 | 38.7890 | 30.0360 |
| *Larinioides cornutus* | I | CHU-SPI-295 | KKCHE498-07 | KF368189 | 58.6180 | -93.8230 |
| *Larinioides cornutus* | A | CHU-SPI-507 | KKCHE710-07 | KF368186 | 58.7470 | -94.1340 |
| *Larinioides cornutus* | I | CHU-SPI-508 | KKCHE711-07 | KF368184 | 58.7470 | -94.1340 |
| *Larinioides cornutus* | I | CHU-SPI-533 | KKCHE736-07 | KF368191 | 58.7470 | -94.1340 |
| *Larinioides cornutus* | A | CHU-SPI-555 | KKCHE758-07 | KF368187 | 58.7470 | -94.1340 |
| *Larinioides cornutus* | I | CHU-SPI-589 | KKCHE792-07 | KF368192 | 58.7470 | -94.1340 |
| *Larinioides cornutus* | I | CHU-SPI-623 | KKCHE826-07 | KF368185 | 58.7470 | -94.1340 |
| *Larinioides cornutus* | I | JBWM0005944f | KKCHE218-07 | KF368190 | 58.7460 | -94.1130 |
| *Larinioides cornutus* | I | JBWM0005944m | KKCHE217-07 | KF368194 | 58.7460 | -94.1130 |
| *Larinioides cornutus* | A | ZMUO.000371 | CHEFI561-12 | KF408095 | 65.0190 | 25.4410 |
| *Larinioides cornutus* | A | ZMUO_00097 | CHEFI097-11 | KF408098 | 63.4300 | 21.0720 |
| *Larinioides patagiatus* | I | 07PROBE-02843 | TWSC229-08 | KF368224 | 58.7300 | -93.7840 |
| *Larinioides patagiatus* | I | 07PROBE-04557 | ERSCH012-07 | KF368215 | 58.7310 | -93.7800 |
| *Larinioides patagiatus* | I | 07PROBE-04564 | ERSCH019-07 | KF368211 | 58.7310 | -93.7800 |
| *Larinioides patagiatus* | I | 07PROBE-04565 | ERSCH020-07 | KF368237 | 58.7310 | -93.7800 |
| *Larinioides patagiatus* | I | 07PROBE-04567 | ERSCH022-07 | KF368236 | 58.7310 | -93.7800 |
| *Larinioides patagiatus* | I | 07PROBE-04568 | ERSCH023-07 | KF368212 | 58.7310 | -93.7800 |
| *Larinioides patagiatus* | I | 07PROBE-04573 | ERSCH028-07 | KF368242 | 58.7310 | -93.7800 |
| *Larinioides patagiatus* | I | 07PROBE-04580 | ERSCH035-07 | KF368244 | 58.7310 | -93.7800 |
| *Larinioides patagiatus* | I | 07PROBE-04581 | ERSCH036-07 | KF368255 | 58.7310 | -93.7800 |
| *Larinioides patagiatus* | I | 07PROBE-04582 | ERSCH037-07 | KF368210 | 58.7310 | -93.7800 |
| *Larinioides patagiatus* | I | 07PROBE-04584 | ERSCH039-07 | KF368196 | 58.7310 | -93.7800 |
| *Larinioides patagiatus* | I | 07PROBE-04585 | ERSCH040-07 | KF368220 | 58.7310 | -93.7800 |
| *Larinioides patagiatus* | I | 07PROBE-04588 | ERSCH043-07 | KF368219 | 58.7310 | -93.7800 |
| *Larinioides patagiatus* | I | 07PROBE-04589 | ERSCH044-07 | KF368233 | 58.7310 | -93.7800 |
| *Larinioides patagiatus* | I | 07PROBE-04591 | ERSCH046-07 | KF368247 | 58.7310 | -93.7800 |
| *Larinioides patagiatus* | I | 07PROBE-04596 | ERSCH051-07 | KF368204 | 58.7310 | -93.7800 |
| *Larinioides patagiatus* | I | 07PROBE-04602 | ERSCH057-07 | KF368199 | 58.7330 | -93.8200 |
| *Larinioides patagiatus* | I | 07PROBE-04607 | ERSCH062-07 | KF368205 | 58.7330 | -93.8200 |
| *Larinioides patagiatus* | I | 07PROBE-04611 | ERSCH066-07 | KF368230 | 58.7330 | -93.8200 |
| *Larinioides patagiatus* | I | 07PROBE-04616 | ERSCH071-07 | KF368197 | 58.7330 | -93.8200 |
| *Larinioides patagiatus* | I | 07PROBE-04617 | ERSCH072-07 | KF368213 | 58.7330 | -93.8200 |
| *Larinioides patagiatus* | I | 07PROBE-04618 | ERSCH073-07 | KF368240 | 58.7330 | -93.8200 |
| *Larinioides patagiatus* | I | 07PROBE-04620 | ERSCH075-07 | KF368245 | 58.7330 | -93.8200 |
| *Larinioides patagiatus* | I | 07PROBE-04622 | ERSCH077-07 | KF368208 | 58.6300 | -93.7980 |
| *Larinioides patagiatus* | I | 07PROBE-04623 | ERSCH078-07 | KF368225 | 58.6300 | -93.7980 |
| *Larinioides patagiatus* | I | 07PROBE-04624 | ERSCH079-07 | KF368253 | 58.6300 | -93.7980 |
| *Larinioides patagiatus* | I | 07PROBE-04628 | ERSCH083-07 | KF368206 | 58.6300 | -93.7980 |
| *Larinioides patagiatus* | I | 07PROBE-04631 | ERSCH086-07 | KF368249 | 58.6300 | -93.7980 |
| *Larinioides patagiatus* | I | 07PROBE-04635 | ERSCH090-07 | KF368207 | 58.6300 | -93.7980 |
| *Larinioides patagiatus* | I | 07PROBE-04738 | ERSCH103-07 | KF368203 | 58.7350 | -94.1090 |
| *Larinioides patagiatus* | I | 07PROBE-04754 | ERSCH119-07 | KF368198 | 58.7350 | -94.1090 |
| *Larinioides patagiatus* | I | 09PROBE-01572 | SWSWE002-09 | GU681073 | 58.7300 | -93.7800 |
| *Larinioides patagiatus* | I | 09PROBE-01575 | SWSWE005-09 | GU681074 | 58.6900 | -93.8500 |
| *Larinioides patagiatus* | I | 09PROBE-01580 | SWSWE010-09 | GU681076 | 58.6300 | -93.8190 |
| *Larinioides patagiatus* | I | 09PROBE-01583 | SWSWE013-09 | GU681075 | 58.6320 | -93.7860 |
| *Larinioides patagiatus* | I | 09PROBE-01584 | SWSWE014-09 | GU681072 | 58.6320 | -93.7860 |
| *Larinioides patagiatus* | I | 09PROBE-01590 | SWSWE020-09 | GU681077 | 58.6320 | -93.7860 |
| *Larinioides patagiatus* | I | 09PROBE-01594 | SWSWE024-09 | GU681062 | 58.7300 | -93.7800 |
| *Larinioides patagiatus* | I | 09PROBE-01615 | SWSWE045-09 | GU681048 | 58.6190 | -93.8290 |
| *Larinioides patagiatus* | I | 09PROBE-01616 | SWSWE046-09 | GU681049 | 58.6320 | -93.7860 |
| *Larinioides patagiatus* | I | 09PROBE-01622 | SWSWE052-09 | GU681045 | 58.6320 | -93.7860 |
| *Larinioides patagiatus* | I | 09PROBE-01623 | SWSWE053-09 | GU681042 | 58.6620 | -94.1650 |
| *Larinioides patagiatus* | I | 09PROBE-01632 | SWSWE062-09 | GU681034 | 58.6320 | -93.7860 |
| *Larinioides patagiatus* | I | 09PROBE-01636 | SWSWE066-09 | GU681033 | 58.6190 | -93.8290 |
| *Larinioides patagiatus* | I | 09PROBE-01644 | SWSWE074-09 | GU681025 | 58.6300 | -93.8190 |
| *Larinioides patagiatus* | I | 09PROBE-01645 | SWSWE075-09 | GU681022 | 58.6600 | -93.8300 |
| *Larinioides patagiatus* | I | 09-PROBE-08124 | JDTGS028-09 | GU679892 | 58.6180 | -93.8290 |
| *Larinioides patagiatus* | I | 09-PROBE-08150 | JDTGS054-09 | GU679870 | 58.6300 | -93.8190 |
| *Larinioides patagiatus* | I | 09-PROBE-08158 | JDTGS062-09 | GU679864 | 58.6250 | -93.8190 |
| *Larinioides patagiatus* | I | 09-PROBE-08186 | JDTGS090-09 | GU679847 | 58.7310 | -93.7800 |
| *Larinioides patagiatus* | I | 09-PROBE-08188 | JDTGS092-09 | GU679845 | 58.6300 | -93.8190 |
| *Larinioides patagiatus* | I | 09-PROBE-08189 | JDTGS093-09 | GU679842 | 58.6250 | -93.8190 |
| *Larinioides patagiatus* | I | 09-PROBE-08190 | JDTGS094-09 | GU679843 | 58.6300 | -93.8190 |
| *Larinioides patagiatus* | A | 09PROBE-1978-482 | SPICH725-09 | KF368200 | 58.6300 | -94.2300 |
| *Larinioides patagiatus* | I | 09PROBE-1978-491 | SPICH734-09 | GU684355 | 58.6300 | -94.2300 |
| *Larinioides patagiatus* | I | 09PROBE-656 | SPICH899-09 | GU684459 | 58.7870 | -93.7140 |
| *Larinioides patagiatus* | I | 09PROBE-657 | SPICH900-09 | GU684460 | 58.7870 | -93.7140 |
| *Larinioides patagiatus* | I | 10PROBE-21182 | GBADC062-10 | HQ956723 | 58.6340 | -93.7860 |
| *Larinioides patagiatus* | I | 10PROBE-21186 | GBADC066-10 | HQ956726 | 58.6610 | -93.8320 |
| *Larinioides patagiatus* | A | BIOUG00627-A04 | SPIRU976-11 | KF368254 | 58.7306 | -93.7804 |
| *Larinioides patagiatus* | I | BIOUG00627-G10 | SPIRU1054-11 | KF368256 | 58.7614 | -94.0115 |
| *Larinioides patagiatus* | A | BIOUG00628-C10 | SPIRU1101-11 | KF368226 | 58.7304 | -93.7805 |
| *Larinioides patagiatus* | I | BIOUG00628-C11 | SPIRU1102-11 | KF368217 | 58.7304 | -93.7805 |
| *Larinioides patagiatus* | I | CHU-SPI-003 | KKCHE112-06 | KF368246 | 58.6180 | -93.8230 |
| *Larinioides patagiatus* | I | CHU-SPI-006 | KKCHE115-06 | KF368232 | 58.6180 | -93.8230 |
| *Larinioides patagiatus* | I | CHU-SPI-027 | KKCHE136-06 | KF368235 | 58.6180 | -93.8230 |
| *Larinioides patagiatus* | I | CHU-SPI-055 | KKCHE164-06 | KF368239 | 58.6220 | -93.8100 |
| *Larinioides patagiatus* | I | CHU-SPI-133 | KKCHE336-07 | KF368228 | 58.6170 | -93.8210 |
| *Larinioides patagiatus* | I | CHU-SPI-143 | KKCHE346-07 | KF368229 | 58.6170 | -93.8210 |
| *Larinioides patagiatus* | I | CHU-SPI-214 | KKCHE417-07 | KF368209 | 58.6910 | -94.1790 |
| *Larinioides patagiatus* | I | CHU-SPI-238 | KKCHE441-07 | KF368252 | 58.6910 | -94.1790 |
| *Larinioides patagiatus* | I | CHU-SPI-250 | KKCHE453-07 | KF368250 | 58.6910 | -94.1790 |
| *Larinioides patagiatus* | I | CHU-SPI-262 | KKCHE465-07 | KF368202 | 58.6910 | -94.1790 |
| *Larinioides patagiatus* | I | CHU-SPI-297 | KKCHE500-07 | KF368218 | 58.6910 | -94.1790 |
| *Larinioides patagiatus* | I | CHU-SPI-305 | KKCHE514-07 | KF368238 | 58.6910 | -94.1790 |
| *Larinioides patagiatus* | I | CHU-SPI-317 | KKCHE538-07 | KF368216 | 58.6910 | -94.1790 |
| *Larinioides patagiatus* | I | CHU-SPI-361 | KKCHE536-07 | KF368223 | 58.6320 | -93.7860 |
| *Larinioides patagiatus* | I | CHU-SPI-402 | KKCHE605-07 | KF368222 | 58.7050 | -94.0520 |
| *Larinioides patagiatus* | I | CHU-SPI-415 | KKCHE618-07 | KF368195 | 58.7540 | -93.9130 |
| *Larinioides patagiatus* | A | CHU-SPI-427 | KKCHE630-07 | KF368227 | 58.7540 | -93.9130 |
| *Larinioides patagiatus* | I | CHU-SPI-428 | KKCHE631-07 | KF368221 | 58.7540 | -93.9130 |
| *Larinioides patagiatus* | I | CHU-SPI-439 | KKCHE642-07 | KF368201 | 58.7540 | -93.9130 |
| *Larinioides patagiatus* | I | CHU-SPI-473 | KKCHE676-07 | KF368214 | 58.7050 | -94.0520 |
| *Larinioides patagiatus* | I | CHU-SPI-475 | KKCHE678-07 | KF368251 | 58.7540 | -93.9130 |
| *Larinioides patagiatus* | I | INV0046 | KKCHE061-06 | KF368231 | 58.7310 | -93.7800 |
| *Larinioides patagiatus* | I | INV0235b | KKCHE106-06 | KF368241 | 58.7540 | -93.9140 |
| *Larinioides patagiatus* | I | INV0313 | KKCHE060-06 | KF368248 | 58.7550 | -93.9130 |
| *Larinioides patagiatus* | I | JBWM0005948 | KKCHE219-07 | KF368243 | 58.6290 | -93.7980 |
| *Larinioides patagiatus* | I | JBWM0005949 | KKCHE220-07 | KF368234 | 58.7710 | -93.8420 |
| *Lepthyphantes alpinus* | I | 07CHU-AR-0036 | KKCHE948-09 | GU683648 | 58.6620 | -93.1890 |
| *Lepthyphantes alpinus* | A | 09PROBE-1828-01 | SPICH240-09 | GU683916 | 58.6300 | -93.8190 |
| *Lepthyphantes alpinus* | A | 09PROBE-1978-03 | SPICH246-09 | GU684303 | 58.6690 | -94.1600 |
| *Lepthyphantes alpinus* | A | 09PROBE-1978-09 | SPICH252-09 | GU684321 | 58.6690 | -94.1600 |
| *Lepthyphantes alpinus* | A | 09PROBE-1978-101 | SPICH344-09 | GU684245 | 58.6690 | -94.1600 |
| *Lepthyphantes alpinus* | I | 09PROBE-1978-104 | SPICH347-09 | GU684243 | 58.6690 | -94.1600 |
| *Lepthyphantes alpinus* | A | 09PROBE-1978-106 | SPICH349-09 | GU684241 | 58.6690 | -94.1600 |
| *Lepthyphantes alpinus* | A | 09PROBE-1978-108 | SPICH351-09 | GU684247 | 58.6690 | -94.1600 |
| *Lepthyphantes alpinus* | A | 09PROBE-1978-115 | SPICH358-09 | GU684252 | 58.6690 | -94.1600 |
| *Lepthyphantes alpinus* | I | 09PROBE-1978-127 | SPICH370-09 | GU684242 | 58.6690 | -94.1600 |
| *Lepthyphantes alpinus* | I | 09PROBE-1978-129 | SPICH372-09 | GU684250 | 58.6690 | -94.1600 |
| *Lepthyphantes alpinus* | I | 09PROBE-1978-136 | SPICH379-09 | GU684239 | 58.6690 | -94.1600 |
| *Lepthyphantes alpinus* | I | 09PROBE-1978-138 | SPICH381-09 | GU684246 | 58.6690 | -94.1600 |
| *Lepthyphantes alpinus* | I | 09PROBE-1978-139 | SPICH382-09 | GU684244 | 58.6690 | -94.1600 |
| *Lepthyphantes alpinus* | I | 09PROBE-1978-140 | SPICH383-09 | GU684248 | 58.6690 | -94.1600 |
| *Lepthyphantes alpinus* | A | 09PROBE-1978-16 | SPICH259-09 | GU684298 | 58.6690 | -94.1600 |
| *Lepthyphantes alpinus* | A | 09PROBE-1978-17 | SPICH260-09 | GU684311 | 58.6690 | -94.1600 |
| *Lepthyphantes alpinus* | A | 09PROBE-1978-21 | SPICH264-09 | GU684318 | 58.6690 | -94.1600 |
| *Lepthyphantes alpinus* | A | 09PROBE-1978-239 | SPICH482-09 | GU684097 | 58.6630 | -94.1670 |
| *Lepthyphantes alpinus* | A | 09PROBE-1978-24 | SPICH267-09 | GU684317 | 58.6690 | -94.1600 |
| *Lepthyphantes alpinus* | A | 09PROBE-1978-243 | SPICH486-09 | GU684111 | 58.6630 | -94.1670 |
| *Lepthyphantes alpinus* | A | 09PROBE-1978-245 | SPICH488-09 | GU684107 | 58.6630 | -94.1670 |
| *Lepthyphantes alpinus* | A | 09PROBE-1978-247 | SPICH490-09 | GU684103 | 58.6630 | -94.1670 |
| *Lepthyphantes alpinus* | A | 09PROBE-1978-250 | SPICH493-09 | GU684115 | 58.6630 | -94.1670 |
| *Lepthyphantes alpinus* | A | 09PROBE-1978-251 | SPICH494-09 | GU684104 | 58.6630 | -94.1670 |
| *Lepthyphantes alpinus* | A | 09PROBE-1978-252 | SPICH495-09 | GU684101 | 58.6630 | -94.1670 |
| *Lepthyphantes alpinus* | I | 09PROBE-1978-254 | SPICH497-09 | GU684113 | 58.6630 | -94.1670 |
| *Lepthyphantes alpinus* | I | 09PROBE-1978-255 | SPICH498-09 | GU684099 | 58.6630 | -94.1670 |
| *Lepthyphantes alpinus* | I | 09PROBE-1978-261 | SPICH504-09 | GU684108 | 58.6630 | -94.1670 |
| *Lepthyphantes alpinus* | I | 09PROBE-1978-262 | SPICH505-09 | GU684112 | 58.6630 | -94.1670 |
| *Lepthyphantes alpinus* | I | 09PROBE-1978-263 | SPICH506-09 | GU684105 | 58.6630 | -94.1670 |
| *Lepthyphantes alpinus* | I | 09PROBE-1978-264 | SPICH507-09 | GU684114 | 58.6630 | -94.1670 |
| *Lepthyphantes alpinus* | I | 09PROBE-1978-265 | SPICH508-09 | GU684110 | 58.6630 | -94.1670 |
| *Lepthyphantes alpinus* | I | 09PROBE-1978-266 | SPICH509-09 | GU684098 | 58.6630 | -94.1670 |
| *Lepthyphantes alpinus* | I | 09PROBE-1978-267 | SPICH510-09 | GU684100 | 58.6630 | -94.1670 |
| *Lepthyphantes alpinus* | I | 09PROBE-1978-268 | SPICH511-09 | GU684109 | 58.6630 | -94.1670 |
| *Lepthyphantes alpinus* | A | 09PROBE-1978-269 | SPICH512-09 | GU684106 | 58.6630 | -94.1670 |
| *Lepthyphantes alpinus* | A | 09PROBE-1978-270 | SPICH513-09 | GU684096 | 58.6630 | -94.1670 |
| *Lepthyphantes alpinus* | A | 09PROBE-1978-274 | SPICH517-09 | GU684102 | 58.6630 | -94.1670 |
| *Lepthyphantes alpinus* | A | 09PROBE-1978-31 | SPICH274-09 | GU684314 | 58.6690 | -94.1600 |
| *Lepthyphantes alpinus* | A | 09PROBE-1978-32 | SPICH275-09 | GU684309 | 58.6690 | -94.1600 |
| *Lepthyphantes alpinus* | A | 09PROBE-1978-37 | SPICH280-09 | GU684312 | 58.6690 | -94.1600 |
| *Lepthyphantes alpinus* | I | 09PROBE-1978-39 | SPICH282-09 | GU684315 | 58.6690 | -94.1600 |
| *Lepthyphantes alpinus* | I | 09PROBE-1978-42 | SPICH285-09 | GU684316 | 58.6690 | -94.1600 |
| *Lepthyphantes alpinus* | I | 09PROBE-1978-43 | SPICH286-09 | GU684304 | 58.6690 | -94.1600 |
| *Lepthyphantes alpinus* | I | 09PROBE-1978-44 | SPICH287-09 | GU684319 | 58.6690 | -94.1600 |
| *Lepthyphantes alpinus* | I | 09PROBE-1978-45 | SPICH288-09 | GU684301 | 58.6690 | -94.1600 |
| *Lepthyphantes alpinus* | A | 09PROBE-1978-50 | SPICH293-09 | GU684299 | 58.6690 | -94.1600 |
| *Lepthyphantes alpinus* | A | 09PROBE-1978-52 | SPICH295-09 | GU684305 | 58.6690 | -94.1600 |
| *Lepthyphantes alpinus* | A | 09PROBE-1978-555 | SPICH798-09 | GU684367 | 58.6690 | -94.1600 |
| *Lepthyphantes alpinus* | A | 09PROBE-1978-557 | SPICH800-09 | GU684366 | 58.6690 | -94.1600 |
| *Lepthyphantes alpinus* | A | 09PROBE-1978-558 | SPICH801-09 | GU684360 | 58.6690 | -94.1600 |
| *Lepthyphantes alpinus* | A | 09PROBE-1978-559 | SPICH802-09 | GU684363 | 58.6690 | -94.1600 |
| *Lepthyphantes alpinus* | A | 09PROBE-1978-561 | SPICH804-09 | GU684364 | 58.6690 | -94.1600 |
| *Lepthyphantes alpinus* | A | 09PROBE-1978-562 | SPICH805-09 | GU684361 | 58.6690 | -94.1600 |
| *Lepthyphantes alpinus* | A | 09PROBE-1978-563 | SPICH806-09 | GU684368 | 58.6690 | -94.1600 |
| *Lepthyphantes alpinus* | A | 09PROBE-1978-564 | SPICH807-09 | GU684369 | 58.6690 | -94.1600 |
| *Lepthyphantes alpinus* | A | 09PROBE-1978-565 | SPICH808-09 | GU684359 | 58.6690 | -94.1600 |
| *Lepthyphantes alpinus* | I | 09PROBE-1978-566 | SPICH809-09 | GU684370 | 58.6690 | -94.1600 |
| *Lepthyphantes alpinus* | I | 09PROBE-1978-567 | SPICH810-09 | GU684362 | 58.6690 | -94.1600 |
| *Lepthyphantes alpinus* | I | 09PROBE-1978-568 | SPICH811-09 | GU684358 | 58.6690 | -94.1600 |
| *Lepthyphantes alpinus* | I | 09PROBE-1978-570 | SPICH813-09 | GU684365 | 58.6690 | -94.1600 |
| *Lepthyphantes alpinus* | A | 09PROBE-1978-59 | SPICH302-09 | GU684313 | 58.6690 | -94.1600 |
| *Lepthyphantes alpinus* | A | 09PROBE-1978-84 | SPICH327-09 | GU684307 | 58.6690 | -94.1600 |
| *Lepthyphantes alpinus* | A | 09PROBE-1978-85 | SPICH328-09 | GU684323 | 58.6690 | -94.1600 |
| *Lepthyphantes alpinus* | I | 09PROBE-1978-87 | SPICH330-09 | GU684302 | 58.6690 | -94.1600 |
| *Lepthyphantes alpinus* | I | 09PROBE-1978-88 | SPICH331-09 | GU684306 | 58.6690 | -94.1600 |
| *Lepthyphantes alpinus* | I | 09PROBE-1978-91 | SPICH334-09 | GU684320 | 58.6690 | -94.1600 |
| *Lepthyphantes alpinus* | I | 09PROBE-1978-92 | SPICH335-09 | GU684310 | 58.6690 | -94.1600 |
| *Lepthyphantes alpinus* | I | 09PROBE-1978-93 | SPICH336-09 | GU684300 | 58.6690 | -94.1600 |
| *Lepthyphantes alpinus* | I | 09PROBE-1978-94 | SPICH337-09 | GU684308 | 58.6690 | -94.1600 |
| *Lepthyphantes alpinus* | I | 09PROBE-1978-99 | SPICH342-09 | GU684249 | 58.6690 | -94.1600 |
| *Lepthyphantes alpinus* | A | 09PROBE-800 | SPICH1021-09 | GU684730 | 58.6260 | -94.2300 |
| *Lepthyphantes alpinus* | A | 09PROBE-804 | SPICH1025-09 | GU684729 | 58.6260 | -94.2300 |
| *Lepthyphantes alpinus* | A | 09PROBE-805 | SPICH1026-09 | GU684727 | 58.6260 | -94.2300 |
| *Lepthyphantes alpinus* | A | 09PROBE-806 | SPICH1027-09 | GU684725 | 58.6260 | -94.2300 |
| *Lepthyphantes alpinus* | A | 09PROBE-807 | SPICH1028-09 | GU684728 | 58.6260 | -94.2300 |
| *Lepthyphantes alpinus* | A | 09PROBE-808 | SPICH1029-09 | GU684726 | 58.6260 | -94.2300 |
| *Lepthyphantes alpinus* | A | 09PROBE-811 | SPICH1032-09 | GU684723 | 58.6260 | -94.2300 |
| *Lepthyphantes alpinus* | A | 09PROBE-813 | SPICH1034-09 | GU684724 | 58.6260 | -94.2300 |
| *Lepthyphantes alpinus* | I | 09PROBE-892 | SPICH1113-09 | GU684605 | 58.6260 | -94.2300 |
| *Lepthyphantes alpinus* | A | 09PROBE-893 | SPICH1114-09 | GU684603 | 58.6260 | -94.2300 |
| *Lepthyphantes alpinus* | A | 09PROBE-897 | SPICH1118-09 | GU684600 | 58.6260 | -94.2300 |
| *Lepthyphantes alpinus* | A | 09PROBE-898 | SPICH1119-09 | GU684606 | 58.6260 | -94.2300 |
| *Lepthyphantes alpinus* | A | 09PROBE-899 | SPICH1120-09 | GU684601 | 58.6260 | -94.2300 |
| *Lepthyphantes alpinus* | A | 09PROBE-901 | SPICH1122-09 | GU684602 | 58.6260 | -94.2300 |
| *Lepthyphantes alpinus* | A | 09PROBE-902 | SPICH1123-09 | GU684604 | 58.6260 | -94.2300 |
| *Lepthyphantes alpinus* | A | BIOUG00630-A10 | SPIRU1267-11 | KF368259 | 58.6319 | -93.7884 |
| *Lepthyphantes alpinus* | I | BIOUG00630-H09 | SPIRU1350-11 | KF368258 | 58.6300 | -93.8190 |
| *Lepthyphantes alpinus* | I | BIOUG00630-H10 | SPIRU1351-11 | KF368257 | 58.6300 | -93.8190 |
| *Mecynargus paetulus* | A | 09PROBE-1856-01 | SPICH025-09 | GU683878 | 58.6300 | -93.8190 |
| *Mecynargus paetulus* | A | 09PROBE-1859-01 | SPICH028-09 | GU683880 | 58.6300 | -93.8190 |
| *Mecynargus paetulus* | A | 09PROBE-1860-01 | SPICH029-09 | GU683877 | 58.6300 | -93.8190 |
| *Mecynargus paetulus* | A | 09PROBE-1867-01 | SPICH036-09 | GU683879 | 58.6300 | -93.8190 |
| *Mecynargus paetulus* | A | 09PROBE-1931-01 | SPICH077-09 | GU683876 | 58.6260 | -94.2300 |
| *Mecynargus paetulus* | A | 09PROBE-1978-100 | SPICH343-09 | GU684199 | 58.6690 | -94.1600 |
| *Mecynargus paetulus* | A | 09PROBE-1978-179 | SPICH422-09 | GU684201 | 58.6260 | -94.2300 |
| *Mecynargus paetulus* | A | 09PROBE-1978-181 | SPICH424-09 | GU684202 | 58.6260 | -94.2300 |
| *Mecynargus paetulus* | A | 09PROBE-1978-185 | SPICH428-09 | GU684200 | 58.6260 | -94.2300 |
| *Mecynargus paetulus* | A | 09PROBE-1978-249 | SPICH492-09 | GU684078 | 58.6630 | -94.1670 |
| *Mecynargus paetulus* | I | 09PROBE-1978-294 | SPICH537-09 | GU684145 | 58.6690 | -94.1600 |
| *Mecynargus paetulus* | A | 09PROBE-1978-299 | SPICH542-09 | GU684139 | 58.6690 | -94.1600 |
| *Mecynargus paetulus* | A | 09PROBE-1978-307 | SPICH550-09 | GU684144 | 58.6690 | -94.1600 |
| *Mecynargus paetulus* | I | 09PROBE-1978-320 | SPICH563-09 | GU684146 | 58.6690 | -94.1600 |
| *Mecynargus paetulus* | A | 09PROBE-1978-336 | SPICH579-09 | GU684143 | 58.6260 | -94.2300 |
| *Mecynargus paetulus* | A | 09PROBE-1978-340 | SPICH583-09 | GU684142 | 58.6260 | -94.2300 |
| *Mecynargus paetulus* | A | 09PROBE-1978-342 | SPICH585-09 | GU684141 | 58.6260 | -94.2300 |
| *Mecynargus paetulus* | A | 09PROBE-1978-54 | SPICH297-09 | GU684289 | 58.6690 | -94.1600 |
| *Mecynargus paetulus* | A | 09PROBE-627 | SPICH870-09 | GU684451 | 58.7900 | -94.2270 |
| *Mecynargus paetulus* | A | BIOUG00627-C05 | SPIRU1001-11 | KF368260 | 58.7544 | -93.9980 |
| *Mecynargus paetulus* | A | BIOUG00628-F07 | SPIRU1134-11 | KF368261 | 58.7544 | -93.9980 |
| *Mecynargus paetulus* | I | BIOUG00630-G02 | SPIRU1331-11 | KF368262 | 58.7690 | -93.8620 |
| *Mecynargus paetulus* | A | BIOUG00632-A11 | SPIRU1363-11 | KF368263 | 58.7690 | -93.8620 |
| *Agyneta amersaxatilis* | I | 09PROBE-874 | SPICH1095-09 | HM416913 | 58.6630 | -94.1670 |
| *Metopobactrus prominulus* | A | 07CHU-AR-0069 | KKCHE981-09 | GU683679 | 58.7630 | -93.8850 |
| *Micaria aenea* | I | 09PROBE-1707-01 | SPICH157-09 | GU683781 | 58.7310 | -93.7800 |
| *Micaria aenea* | I | 09PROBE-1756-01 | SPICH187-09 | GU683778 | 58.7310 | -93.7800 |
| *Micaria aenea* | A | 09PROBE-1808-01 | SPICH220-09 | GU683946 | 58.6300 | -93.8190 |
| *Micaria alpina* | A | 09PROBE-1970-01 | SPISH035-09 | GU683953 | 58.6260 | -94.2300 |
| *Micaria alpina* | A | 09PROBE-747 | SPICH990-09 | GU684496 | 58.6600 | -94.1700 |
| *Micaria alpina* | I | JBWM0005985 | KKCHE231-07 | KF368267 | 58.7380 | -93.8190 |
| *Micaria constricta* | I | 07CHU-AR-0049 | KKCHE961-09 | GU683662 | 58.7630 | -93.8680 |
| *Micaria constricta* | I | 07CHU-AR-0051 | KKCHE963-09 | GU683665 | 58.7600 | -94.0860 |
| *Micaria constricta* | I | 07CHU-AR-0053 | KKCHE965-09 | GU683664 | 58.7600 | -94.0860 |
| *Micaria constricta* | I | 07CHU-AR-0054 | KKCHE966-09 | HM862436 | 58.7600 | -94.0860 |
| *Micaria constricta* | I | 07CHU-AR-0056 | KKCHE968-09 | GU683666 | 58.7640 | -94.0870 |
| *Micaria constricta* | I | 07CHU-AR-0057 | KKCHE969-09 | GU683670 | 58.7710 | -93.8430 |
| *Micaria constricta* | A | 07CHU-AR-0089 | KKCHE1001-09 | GU683690 | 58.7550 | -93.9990 |
| *Micaria constricta* | I | 07CHU-AR-0234 | KKCHE1041-09 | GU683725 | 58.7500 | -93.9100 |
| *Micaria constricta* | I | 07CHU-AR-0235 | KKCHE1042-09 | GU683724 | 58.7500 | -93.9100 |
| *Micaria constricta* | I | 07PROBE-02834 | TWSC220-08 | KF368285 | 58.7600 | -94.0860 |
| *Micaria constricta* | A | 09PROBE-1674-01 | SPICH102-09 | GU683776 | 58.6180 | -93.8290 |
| *Micaria constricta* | I | 09PROBE-1697-01 | SPICH142-09 | GU683777 | 58.7310 | -93.7800 |
| *Micaria constricta* | I | 09PROBE-1697-02 | SPICH143-09 | GU683779 | 58.7310 | -93.7800 |
| *Micaria constricta* | A | 09PROBE-1807-01 | SPICH219-09 | GU683945 | 58.6300 | -93.8190 |
| *Micaria constricta* | I | 09PROBE-1894-01 | SPICH059-09 | GU683833 | 58.7700 | -93.8430 |
| *Micaria constricta* | A | 09PROBE-1904-01 | SPICH062-09 | GU683905 | 58.6180 | -93.8290 |
| *Micaria constricta* | A | BIOUG00629-C10 | SPIRU1196-11 | KF368270 | 58.7544 | -93.9980 |
| *Micaria constricta* | I | BIOUG00629-G07 | SPIRU1241-11 | KF368280 | 58.7710 | -93.8510 |
| *Micaria constricta* | A | BIOUG00629-H02 | SPIRU1248-11 | KF368279 | 58.7710 | -93.8510 |
| *Micaria constricta* | A | BIOUG00630-A11 | SPIRU1268-11 | KF368277 | 58.7710 | -93.8510 |
| *Micaria constricta* | A | BIOUG00630-E08 | SPIRU1313-11 | KF368268 | 58.7305 | -93.7805 |
| *Micaria constricta* | A | BIOUG00632-B04 | SPIRU1368-11 | KF368290 | 58.7710 | -93.8510 |
| *Micaria constricta* | A | BIOUG00632-B05 | SPIRU1369-11 | KF368275 | 58.7710 | -93.8510 |
| *Micaria constricta* | I | CHU-SPI-400 | KKCHE603-07 | KF368272 | 58.7380 | -93.8190 |
| *Micaria constricta* | I | CHU-SPI-401 | KKCHE604-07 | KF368284 | 58.7380 | -93.8190 |
| *Micaria constricta* | I | CHU-SPI-412 | KKCHE615-07 | KF368276 | 58.7380 | -93.8190 |
| *Micaria constricta* | I | CHU-SPI-413 | KKCHE616-07 | KF368288 | 58.7380 | -93.8190 |
| *Micaria constricta* | I | CHU-SPI-424 | KKCHE627-07 | KF368282 | 58.7380 | -93.8190 |
| *Micaria constricta* | I | CHU-SPI-471 | KKCHE674-07 | KF368278 | 58.7380 | -93.8190 |
| *Micaria constricta* | I | CHU-SPI-472 | KKCHE675-07 | KF368283 | 58.7380 | -93.8190 |
| *Micaria constricta* | I | CHU-SPI-510 | KKCHE713-07 | KF368286 | 58.7380 | -93.8190 |
| *Micaria constricta* | I | CHU-SPI-586 | KKCHE789-07 | KF368274 | 58.7400 | -93.8200 |
| *Micaria constricta* | A | CHU-SPI-591 | KKCHE794-07 | KF368281 | 58.7400 | -93.8200 |
| *Micaria constricta* | I | CHU-SPI-595 | KKCHE798-07 | KF368287 | 58.7400 | -93.8200 |
| *Micaria constricta* | I | CHU-SPI-596 | KKCHE799-07 | KF368291 | 58.7400 | -93.8200 |
| *Micaria constricta* | I | CHU-SPI-610 | KKCHE813-07 | KF368269 | 58.7400 | -93.8200 |
| *Micaria constricta* | I | CHU-SPI-616 | KKCHE819-07 | KF368289 | 58.7400 | -93.8200 |
| *Micaria constricta* | I | CHU-SPI-621 | KKCHE824-07 | KF368273 | 58.7400 | -93.8200 |
| *Micaria constricta* | I | JBWM0005986 | KKCHE232-07 | KF368271 | 58.7540 | -93.9130 |
| *Micaria pulicaria* | A | 09PROBE-1806-01 | SPICH215-09 | GU683944 | 58.6300 | -93.8190 |
| *Micaria pulicaria* | A | 09PROBE-1806-02 | SPICH216-09 | GU683941 | 58.6300 | -93.8190 |
| *Micaria pulicaria* | A | 09PROBE-1806-03 | SPICH217-09 | GU683942 | 58.6300 | -93.8190 |
| *Micaria pulicaria* | A | 09PROBE-1806-04 | SPICH218-09 | GU683943 | 58.6300 | -93.8190 |
| *Micaria pulicaria* | I | CHU-SPI-425 | KKCHE628-07 | KF368295 | 58.7380 | -93.8190 |
| *Micaria pulicaria* | I | CHU-SPI-437 | KKCHE640-07 | KF368293 | 58.7380 | -93.8190 |
| *Micaria pulicaria* | I | CHU-SPI-460 | KKCHE663-07 | KF368294 | 58.7380 | -93.8190 |
| *Micaria pulicaria* | I | CHU-SPI-620 | KKCHE823-07 | KF368292 | 58.7400 | -93.8200 |
| *Microlinyphia pusilla* | A | 09PROBE-1978-38 | SPICH281-09 | HM432629 | 58.6690 | -94.1600 |
| *Microlinyphia pusilla* | A | BIOUG00627-F03 | SPIRU1035-11 | KF368296 | 58.6329 | -93.7869 |
| *Mughiphantes* sp. 1GAB | I | 09-PROBE-08161 | JDTGS065-09 | GU679862 | 58.6180 | -93.8290 |
| *Mughiphantes* sp. 1GAB | I | 09-PROBE-08170 | JDTGS074-09 | GU679857 | 58.6300 | -93.8190 |
| *Mughiphantes* sp. 1GAB | A | 09PROBE-687 | SPICH930-09 | GU684464 | 58.7310 | -93.7800 |
| *Oedothorax trilobatus* | A | 09PROBE-1978-312 | SPICH555-09 | GU684170 | 58.6690 | -94.1600 |
| *Oedothorax trilobatus* | I | 09PROBE-882 | SPICH1103-09 | GU684608 | 58.6630 | -94.1670 |
| *Oedothorax trilobatus* | A | 09PROBE-883 | SPICH1104-09 | GU684609 | 58.6630 | -94.1670 |
| *Oedothorax trilobatus* | A | 09PROBE-884 | SPICH1105-09 | GU684610 | 58.6630 | -94.1670 |
| *Oedothorax trilobatus* | A | 09PROBE-939 | SPICH1160-09 | GU684607 | 58.6260 | -94.2300 |
| *Ohlertidion ohlerti* | A | 09PROBE-904 | SPICH1125-09 | GU684643 | 58.6300 | -93.7980 |
| *Ohlertidion ohlerti* | A | 09PROBE-925 | SPICH1146-09 | GU684644 | 58.6300 | -93.7980 |
| *Ohlertidion ohlerti* | A | 09PROBE-926 | SPICH1147-09 | GU684645 | 58.6300 | -93.7980 |
| *Ohlertidion ohlerti* | I | 10PROBE-21122 | GBADC002-10 | HQ956666 | 58.7340 | -94.1120 |
| *Ohlertidion ohlerti* | I | 10PROBE-21128 | GBADC008-10 | HQ956672 | 58.7340 | -94.1120 |
| *Ohlertidion ohlerti* | I | 10PROBE-21130 | GBADC010-10 | HQ956674 | 58.7340 | -94.1120 |
| *Ohlertidion ohlerti* | I | 10PROBE-21135 | GBADC015-10 | HQ956679 | 58.7340 | -94.1120 |
| *Ohlertidion ohlerti* | I | 10PROBE-21152 | GBADC032-10 | HQ956695 | 58.7340 | -94.1120 |
| *Ohlertidion ohlerti* | A | BIOUG00627-A07 | SPIRU979-11 | KF368297 | 58.6921 | -94.1320 |
| *Ohlertidion ohlerti* | A | BIOUG00629-B06 | SPIRU1180-11 | KF368303 | 58.6186 | -93.8291 |
| *Ohlertidion ohlerti* | A | BIOUG00629-B07 | SPIRU1181-11 | KF368299 | 58.6186 | -93.8291 |
| *Ohlertidion ohlerti* | A | BIOUG00629-B08 | SPIRU1182-11 | KF368302 | 58.6186 | -93.8291 |
| *Ohlertidion ohlerti* | A | BIOUG00629-C07 | SPIRU1193-11 | KF368301 | 58.7179 | -94.1221 |
| *Ohlertidion ohlerti* | A | BIOUG00629-C08 | SPIRU1194-11 | KF368298 | 58.7179 | -94.1221 |
| *Ohlertidion ohlerti* | A | BIOUG00630-F11 | SPIRU1328-11 | KF368300 | 58.7305 | -93.7805 |
| *Oreoneta leviceps* | A | BIOUG00629-E10 | SPIRU1220-11 | KF368304 | 58.7710 | -93.8510 |
| *Oreonetides vaginatus* | I | 07CHU-AR-0039 | KKCHE951-09 | GU683654 | 58.6620 | -93.1890 |
| *Oreonetides vaginatus* | I | 09PROBE-1708-01 | SPICH158-09 | GU684018 | 58.6300 | -93.8190 |
| *Oreonetides vaginatus* | I | 09PROBE-1749-01 | SPICH182-09 | GU684013 | 58.7310 | -93.7800 |
| *Oreonetides vaginatus* | I | 09PROBE-1941-01 | SPICH080-09 | GU683850 | 58.7640 | -93.8970 |
| *Oreonetides vaginatus* | I | 09PROBE-1978-144 | SPICH387-09 | GU684220 | 58.7380 | -93.8190 |
| *Oreonetides vaginatus* | A | 09PROBE-1978-158 | SPICH401-09 | GU684217 | 58.7310 | -93.7800 |
| *Oreonetides vaginatus* | I | 09PROBE-1978-382 | SPICH625-09 | GU684532 | 58.6250 | -93.8160 |
| *Oreonetides vaginatus* | A | 09PROBE-801 | SPICH1022-09 | GU684674 | 58.6260 | -94.2300 |
| *Oreonetides vaginatus* | A | BIOUG00632-A08 | SPIRU1360-11 | KF368305 | 58.6173 | -93.8123 |
| *Ozyptila arctica* | A | 07CHU-AR-0016 | KKCHE928-09 | GU683634 | 58.7600 | -94.0860 |
| *Ozyptila arctica* | A | 07CHU-AR-0017 | KKCHE929-09 | GU683633 | 58.7600 | -94.0860 |
| *Ozyptila arctica* | A | 07CHU-AR-0018 | KKCHE930-09 | GU683639 | 58.6260 | -94.2300 |
| *Ozyptila arctica* | I | 07CHU-AR-0019 | KKCHE931-09 | GU683638 | 58.6260 | -94.2300 |
| *Ozyptila arctica* | I | 07CHU-AR-0020 | KKCHE932-09 | GU683637 | 58.6260 | -94.2300 |
| *Ozyptila arctica* | I | 07CHU-AR-0021 | KKCHE933-09 | GU683636 | 58.6260 | -94.2300 |
| *Ozyptila arctica* | I | 07CHU-AR-0022 | KKCHE934-09 | GU683642 | 58.6260 | -94.2300 |
| *Ozyptila arctica* | I | 07CHU-AR-0030 | KKCHE942-09 | GU683646 | 58.7050 | -94.0540 |
| *Ozyptila arctica* | I | 07CHU-AR-0055 | KKCHE967-09 | GU683667 | 58.7710 | -93.8430 |
| *Ozyptila arctica* | I | 07CHU-AR-0071 | KKCHE983-09 | GU683678 | 58.7380 | -93.8190 |
| *Ozyptila arctica* | I | 07CHU-AR-0087 | KKCHE999-09 | GU683687 | 58.7550 | -93.9990 |
| *Ozyptila arctica* | I | 07CHU-AR-0088 | KKCHE1000-09 | GU683691 | 58.7550 | -93.9990 |
| *Ozyptila arctica* | I | 09-PROBE-08106 | JDTGS010-09 | GU679904 | 58.6250 | -93.8190 |
| *Ozyptila arctica* | A | 09PROBE-1688-01 | SPICH127-09 | GU683805 | 58.6180 | -93.8290 |
| *Ozyptila arctica* | A | BIOUG00630-D08 | SPIRU1301-11 | KF368306 | 58.7707 | -94.1808 |
| *Ozyptila arctica* | A | BIOUG00630-E02 | SPIRU1307-11 | KF368310 | 58.7305 | -93.7805 |
| *Ozyptila arctica* | A | BIOUG00630-E11 | SPIRU1316-11 | KF368307 | 58.7720 | -93.8431 |
| *Ozyptila arctica* | I | CHU-SPI-411 | KKCHE614-07 | KF368309 | 58.7380 | -93.8190 |
| *Ozyptila arctica* | I | CHU-SPI-448 | KKCHE651-07 | KF368308 | 58.7380 | -93.8190 |
| *Ozyptila arctica* | I | CHU-SPI-539 | KKCHE742-07 | KF368311 | 58.7470 | -94.1340 |
| *Ozyptila gertschi* | A | 09PROBE-862 | SPICH1083-09 | GU684593 | 58.6630 | -94.1670 |
| *Pachygnatha clercki* | A | 09PROBE-1662-01 | SPISH025-09 | GU683918 | 58.6690 | -94.1600 |
| *Pachygnatha clercki* | A | 09PROBE-1681-01 | SPICH115-09 | GU684027 | 58.6630 | -94.1670 |
| *Pachygnatha clercki* | A | 09PROBE-1978-102 | SPICH345-09 | GU684272 | 58.6690 | -94.1600 |
| *Pachygnatha clercki* | A | 09PROBE-1978-105 | SPICH348-09 | GU684273 | 58.6690 | -94.1600 |
| *Pachygnatha clercki* | I | 09PROBE-1978-314 | SPICH557-09 | GU684184 | 58.6690 | -94.1600 |
| *Pachygnatha clercki* | I | 09PROBE-1978-315 | SPICH558-09 | GU684173 | 58.6690 | -94.1600 |
| *Pachygnatha clercki* | I | 09PROBE-1978-316 | SPICH559-09 | GU684174 | 58.6690 | -94.1600 |
| *Pachygnatha clercki* | I | 09PROBE-1978-321 | SPICH564-09 | GU684171 | 58.6690 | -94.1600 |
| *Pachygnatha clercki* | A | 09PROBE-857 | SPICH1078-09 | GU684581 | 58.6630 | -94.1670 |
| *Pachygnatha clercki* | A | 09PROBE-858 | SPICH1079-09 | GU684582 | 58.6630 | -94.1670 |
| *Pachygnatha clercki* | A | 09PROBE-859 | SPICH1080-09 | GU684579 | 58.6630 | -94.1670 |
| *Pachygnatha clercki* | A | 09PROBE-860 | SPICH1081-09 | GU684583 | 58.6630 | -94.1670 |
| *Pachygnatha clercki* | A | 09PROBE-861 | SPICH1082-09 | GU684580 | 58.6630 | -94.1670 |
| *Pardosa dromaea* | I | 07CHU-AR-0207 | KKCHE1014-09 | GU683703 | 58.7380 | -93.8190 |
| *Pardosa dromaea* | I | 07CHU-AR-0222 | KKCHE1029-09 | GU683715 | 58.7630 | -93.8680 |
| *Pardosa dromaea* | I | 07CHU-AR-0226 | KKCHE1033-09 | GU683718 | 58.7940 | -94.2170 |
| *Pardosa dromaea* | I | 07CHU-AR-0239 | KKCHE1046-09 | GU683727 | 58.7800 | -94.1960 |
| *Pardosa dromaea* | I | 07CHU-AR-0254 | KKCHE1061-09 | GU683737 | 58.7600 | -94.0860 |
| *Pardosa dromaea* | A | 07CHU-AR-0262 | KKCHE1069-09 | GU683748 | 58.7700 | -93.8400 |
| *Pardosa dromaea* | A | 07CHU-AR-0275 | KKCHE1082-09 | GU683595 | 58.7950 | -94.2200 |
| *Pardosa dromaea* | I | 07CHU-AR-0276 | KKCHE1083-09 | GU683598 | 58.7710 | -93.8430 |
| *Pardosa dromaea* | I | 07CHU-AR-0293 | KKCHE1100-09 | GU683602 | 58.7650 | -93.8670 |
| *Pardosa dromaea* | I | 07PROBE-04093 | TWSC278-08 | KF368318 | 58.7760 | -93.7360 |
| *Pardosa dromaea* | I | 09-PROBE-08120 | JDTGS024-09 | GU679897 | 58.7890 | -93.7100 |
| *Pardosa dromaea* | I | 09-PROBE-08121 | JDTGS025-09 | GU679836 | 58.7890 | -93.7100 |
| *Pardosa dromaea* | I | BIOUG00633-A11 | SPIRU1399-11 | KF368312 | 58.7710 | -93.8510 |
| *Pardosa dromaea* | A | BIOUG00633-B03 | SPIRU1403-11 | KF368316 | 58.6290 | -93.7980 |
| *Pardosa dromaea* | A | BIOUG00633-B11 | SPIRU1411-11 | KF368314 | 58.7710 | -93.8510 |
| *Pardosa dromaea* | A | BIOUG00633-B12 | SPIRU1412-11 | KF368315 | 58.7710 | -93.8510 |
| *Pardosa dromaea* | I | HLC14154 | KKCHE020-06 | KF368317 | 58.7640 | -93.8680 |
| *Pardosa dromaea* | I | INV0327 | KKCHE038-06 | KF368319 | 58.6580 | -94.1670 |
| *Pardosa dromaea* | I | JBWM0234323 | KKCHE309-07 | KF368313 | 58.7710 | -93.8420 |
| *Pardosa furcifera* | I | 07CHU-AR-0244 | KKCHE1051-09 | GU683731 | 58.7850 | -94.2030 |
| *Pardosa furcifera* | I | 07CHU-AR-0246 | KKCHE1053-09 | GU683730 | 58.7850 | -94.2030 |
| *Pardosa furcifera* | I | 07CHU-AR-0247 | KKCHE1054-09 | GU683729 | 58.7850 | -94.2030 |
| *Pardosa furcifera* | A | 07PROBE-02748 | TWSC135-08 | KF368398 | 58.7050 | -94.0520 |
| *Pardosa furcifera* | I | 07PROBE-02752 | TWSC139-08 | KF368342 | 58.7050 | -94.0520 |
| *Pardosa furcifera* | I | 07PROBE-02753 | TWSC140-08 | KF368362 | 58.7380 | -93.8190 |
| *Pardosa furcifera* | A | 07PROBE-02755 | TWSC142-08 | KF368341 | 58.7380 | -93.8190 |
| *Pardosa furcifera* | A | 07PROBE-02761 | TWSC148-08 | KF368349 | 58.7380 | -93.8190 |
| *Pardosa furcifera* | I | 07PROBE-02764 | TWSC151-08 | KF368386 | 58.7050 | -94.0520 |
| *Pardosa furcifera* | A | 07PROBE-02766 | TWSC153-08 | KF368374 | 58.7050 | -94.0520 |
| *Pardosa furcifera* | I | 07PROBE-02767 | TWSC154-08 | KF368360 | 58.7380 | -93.8190 |
| *Pardosa furcifera* | A | 07PROBE-02769 | TWSC156-08 | KF368372 | 58.7050 | -94.0520 |
| *Pardosa furcifera* | A | 07PROBE-02771 | TWSC158-08 | KF368371 | 58.7050 | -94.0520 |
| *Pardosa furcifera* | I | 07PROBE-02780 | TWSC166-08 | KF368323 | 58.7380 | -93.8190 |
| *Pardosa furcifera* | A | 07PROBE-02783 | TWSC169-08 | KF368350 | 58.7050 | -94.0520 |
| *Pardosa furcifera* | A | 07PROBE-02786 | TWSC172-08 | KF368340 | 58.7050 | -94.0520 |
| *Pardosa furcifera* | A | 07PROBE-02789 | TWSC175-08 | KF368368 | 58.7570 | -93.9840 |
| *Pardosa furcifera* | A | 07PROBE-02795 | TWSC181-08 | KF368395 | 58.7050 | -94.0520 |
| *Pardosa furcifera* | A | 07PROBE-02812 | TWSC198-08 | KF368393 | 58.7570 | -93.9840 |
| *Pardosa furcifera* | A | 07PROBE-02827 | TWSC213-08 | KF368334 | 58.7310 | -93.7800 |
| *Pardosa furcifera* | A | 07PROBE-02831 | TWSC217-08 | KF368367 | 58.7300 | -93.7840 |
| *Pardosa furcifera* | I | 07PROBE-02886 | TWSC272-08 | KF368355 | 58.7300 | -93.7840 |
| *Pardosa furcifera* | I | 07PROBE-06490 | TWSC004-07 | KF368322 | 58.7050 | -94.0520 |
| *Pardosa furcifera* | A | 07PROBE-06559 | TWSC073-07 | KF368366 | 58.7050 | -94.0520 |
| *Pardosa furcifera* | A | 07PROBE-06560 | TWSC074-07 | KF368347 | 58.7310 | -93.7800 |
| *Pardosa furcifera* | A | 07PROBE-06562 | TWSC076-07 | KF368344 | 58.6170 | -93.8140 |
| *Pardosa furcifera* | I | 07PROBE-06569 | TWSC083-07 | KF368351 | 58.6300 | -93.8190 |
| *Pardosa furcifera* | A | 07PROBE-06571 | TWSC085-07 | KF368399 | 58.7050 | -94.0520 |
| *Pardosa furcifera* | A | 07PROBE-06572 | TWSC086-07 | KF368376 | 58.7050 | -94.0520 |
| *Pardosa furcifera* | A | 07PROBE-06575 | TWSC089-07 | KF368338 | 58.7050 | -94.0520 |
| *Pardosa furcifera* | A | 07PROBE-06579 | TWSC093-07 | KF368343 | 58.7370 | -93.8230 |
| *Pardosa furcifera* | A | 07PROBE-06580 | TWSC094-07 | KF368345 | 58.7050 | -94.0520 |
| *Pardosa furcifera* | A | 09-PROBE-08098 | JDTGS002-09 | GU679911 | 58.6250 | -93.8190 |
| *Pardosa furcifera* | A | 09-PROBE-08108 | JDTGS012-09 | GU679902 | 58.6250 | -93.8190 |
| *Pardosa furcifera* | A | 09-PROBE-08123 | JDTGS027-09 | GU679895 | 58.7300 | -93.7840 |
| *Pardosa furcifera* | A | 09PROBE-1589-01 | SPISH003-09 | GU683935 | 58.7900 | -94.2270 |
| *Pardosa furcifera* | A | 09PROBE-1978-505 | SPICH748-09 | GU684394 | 58.7960 | -93.7540 |
| *Pardosa furcifera* | I | BIOUG00629-G10 | SPIRU1244-11 | KF368385 | 58.7710 | -93.8510 |
| *Pardosa furcifera* | A | BIOUG00633-A01 | SPIRU1389-11 | KF368353 | 58.6329 | -93.7869 |
| *Pardosa furcifera* | A | BIOUG00633-A02 | SPIRU1390-11 | KF368381 | 58.6329 | -93.7869 |
| *Pardosa furcifera* | A | BIOUG00633-A03 | SPIRU1391-11 | KF368336 | 58.6329 | -93.7869 |
| *Pardosa furcifera* | I | BIOUG00633-A04 | SPIRU1392-11 | KF368335 | 58.7544 | -93.9980 |
| *Pardosa furcifera* | I | BIOUG00633-A05 | SPIRU1393-11 | KF368378 | 58.7544 | -93.9980 |
| *Pardosa furcifera* | A | BIOUG00633-A08 | SPIRU1396-11 | KF368380 | 58.7544 | -93.9980 |
| *Pardosa furcifera* | A | BIOUG00633-B06 | SPIRU1406-11 | KF368332 | 58.6610 | -93.8320 |
| *Pardosa furcifera* | A | BIOUG00633-B09 | SPIRU1409-11 | KF368329 | 58.6610 | -93.8320 |
| *Pardosa furcifera* | I | CHU-SPI-369 | KKCHE556-07 | KF368337 | 58.6320 | -93.7860 |
| *Pardosa furcifera* | I | CHU-SPI-395 | KKCHE598-07 | KF368354 | 58.7380 | -93.8190 |
| *Pardosa furcifera* | I | CHU-SPI-396 | KKCHE599-07 | KF368394 | 58.7380 | -93.8190 |
| *Pardosa furcifera* | I | CHU-SPI-397 | KKCHE600-07 | KF368379 | 58.7380 | -93.8190 |
| *Pardosa furcifera* | I | CHU-SPI-405 | KKCHE608-07 | KF368328 | 58.7380 | -93.8190 |
| *Pardosa furcifera* | I | CHU-SPI-406 | KKCHE609-07 | KF368321 | 58.7380 | -93.8190 |
| *Pardosa furcifera* | I | CHU-SPI-407 | KKCHE610-07 | KF368401 | 58.7380 | -93.8190 |
| *Pardosa furcifera* | I | CHU-SPI-409 | KKCHE612-07 | KF368400 | 58.7380 | -93.8190 |
| *Pardosa furcifera* | I | CHU-SPI-417 | KKCHE620-07 | KF368325 | 58.7380 | -93.8190 |
| *Pardosa furcifera* | I | CHU-SPI-419 | KKCHE622-07 | KF368324 | 58.7380 | -93.8190 |
| *Pardosa furcifera* | I | CHU-SPI-421 | KKCHE624-07 | KF368392 | 58.7380 | -93.8190 |
| *Pardosa furcifera* | I | CHU-SPI-429 | KKCHE632-07 | KF368330 | 58.7380 | -93.8190 |
| *Pardosa furcifera* | I | CHU-SPI-430 | KKCHE633-07 | KF368365 | 58.7380 | -93.8190 |
| *Pardosa furcifera* | I | CHU-SPI-441 | KKCHE644-07 | KF368389 | 58.7380 | -93.8190 |
| *Pardosa furcifera* | I | CHU-SPI-442 | KKCHE645-07 | KF368396 | 58.7380 | -93.8190 |
| *Pardosa furcifera* | I | CHU-SPI-443 | KKCHE646-07 | KF368331 | 58.7380 | -93.8190 |
| *Pardosa furcifera* | I | CHU-SPI-444 | KKCHE647-07 | KF368388 | 58.7380 | -93.8190 |
| *Pardosa furcifera* | I | CHU-SPI-453 | KKCHE656-07 | KF368402 | 58.7380 | -93.8190 |
| *Pardosa furcifera* | I | CHU-SPI-459 | KKCHE662-07 | KF368357 | 58.7380 | -93.8190 |
| *Pardosa furcifera* | I | CHU-SPI-470 | KKCHE673-07 | KF368397 | 58.7380 | -93.8190 |
| *Pardosa furcifera* | I | CHU-SPI-478 | KKCHE681-07 | KF368369 | 58.7380 | -93.8190 |
| *Pardosa furcifera* | I | CHU-SPI-501 | KKCHE704-07 | KF368358 | 58.7470 | -94.1340 |
| *Pardosa furcifera* | I | CHU-SPI-513 | KKCHE716-07 | KF368382 | 58.7470 | -94.1340 |
| *Pardosa furcifera* | I | CHU-SPI-516 | KKCHE719-07 | KF368363 | 58.7470 | -94.1340 |
| *Pardosa furcifera* | I | CHU-SPI-526 | KKCHE729-07 | KF368370 | 58.7470 | -94.1340 |
| *Pardosa furcifera* | I | CHU-SPI-528 | KKCHE731-07 | KF368348 | 58.7470 | -94.1340 |
| *Pardosa furcifera* | I | CHU-SPI-529 | KKCHE732-07 | KF368326 | 58.7470 | -94.1340 |
| *Pardosa furcifera* | A | CHU-SPI-551 | KKCHE754-07 | KF368377 | 58.7470 | -94.1340 |
| *Pardosa furcifera* | A | CHU-SPI-561 | KKCHE764-07 | KF368387 | 58.7470 | -94.1340 |
| *Pardosa furcifera* | I | CHU-SPI-564 | KKCHE767-07 | KF368364 | 58.7470 | -94.1340 |
| *Pardosa furcifera* | I | CHU-SPI-575 | KKCHE778-07 | KF368320 | 58.7470 | -94.1340 |
| *Pardosa furcifera* | A | CHU-SPI-577 | KKCHE780-07 | KF368390 | 58.7470 | -94.1340 |
| *Pardosa furcifera* | A | CHU-SPI-587 | KKCHE790-07 | KF368346 | 58.7400 | -93.8200 |
| *Pardosa furcifera* | I | HLC13386 | KKCHE034-06 | KF368359 | 58.7680 | -93.8680 |
| *Pardosa furcifera* | I | HLC13432 | KKCHE039-06 | KF368384 | 58.7680 | -93.8680 |
| *Pardosa furcifera* | I | HLC-26811 | KKCHE842-09 | KF368339 | 58.7800 | -94.1860 |
| *Pardosa furcifera* | I | HLC-26842 | KKCHE873-09 | KF368391 | 58.7800 | -94.1860 |
| *Pardosa furcifera* | I | HLC-26876 | KKCHE907-09 | KF368361 | 58.7310 | -93.7800 |
| *Pardosa furcifera* | I | INV0224 | KKCHE050-06 | KF368333 | 58.6320 | -93.7860 |
| *Pardosa furcifera* | A | INV0304 | KKCHE045-06 | KF368383 | 58.6320 | -93.7860 |
| *Pardosa furcifera* | I | JBWM0006133 | KKCHE268-07 | KF368356 | 58.7540 | -93.9130 |
| *Pardosa furcifera* | I | JBWM0006138 | KKCHE270-07 | KF368375 | 58.1170 | -92.8500 |
| *Pardosa furcifera* | A | SD0406HE301 | SAPIT162-08 | KF368352 | 58.7300 | -93.8000 |
| *Pardosa furcifera* | I | SD2707HE602 | SAPIT026-08 | KF368373 | 58.7300 | -93.8000 |
| *Pardosa furcifera* | I | SD2707SG502 | SAPIT050-08 | KF368327 | 58.7300 | -93.8000 |
| *Pardosa fuscula* | I | 07CHU-AR-0233 | KKCHE1040-09 | GU683721 | 58.7600 | -93.9620 |
| *Pardosa fuscula* | I | 07PROBE-06513 | TWSC027-07 | KF368420 | 58.7570 | -93.9840 |
| *Pardosa fuscula* | A | 09PROBE-1929-01 | SPICH075-09 | GU683901 | 58.6260 | -94.2300 |
| *Pardosa fuscula* | A | 09PROBE-1929-02 | SPICH076-09 | GU683902 | 58.6260 | -94.2300 |
| *Pardosa fuscula* | A | 09PROBE-750 | SPICH993-09 | GU684505 | 58.6260 | -94.2300 |
| *Pardosa fuscula* | I | BIOUG00633-A06 | SPIRU1394-11 | KF368419 | 58.6329 | -93.7869 |
| *Pardosa fuscula* | A | BIOUG00633-B07 | SPIRU1407-11 | KF368424 | 58.6610 | -93.8320 |
| *Pardosa fuscula* | A | BIOUG00633-B08 | SPIRU1408-11 | KF368409 | 58.6610 | -93.8320 |
| *Pardosa fuscula* | I | CHU-SPI-121 | KKCHE324-07 | KF368405 | 58.6170 | -93.8210 |
| *Pardosa fuscula* | I | CHU-SPI-304 | KKCHE507-07 | KF368408 | 58.6910 | -94.1790 |
| *Pardosa fuscula* | I | CHU-SPI-310 | KKCHE519-07 | KF368410 | 58.6910 | -94.1790 |
| *Pardosa fuscula* | I | CHU-SPI-316 | KKCHE531-07 | KF368407 | 58.6910 | -94.1790 |
| *Pardosa fuscula* | I | CHU-SPI-410 | KKCHE613-07 | KF368406 | 58.7380 | -93.8190 |
| *Pardosa fuscula* | A | CHU-SPI-479 | KKCHE682-07 | KF368403 | 58.7380 | -93.8190 |
| *Pardosa fuscula* | I | CHU-SPI-538 | KKCHE741-07 | KF368415 | 58.7470 | -94.1340 |
| *Pardosa fuscula* | I | HLC13358 | KKCHE046-06 | KF368421 | 58.7680 | -93.8680 |
| *Pardosa fuscula* | I | HLC13375 | KKCHE019-06 | KF368412 | 58.7640 | -93.8680 |
| *Pardosa fuscula* | I | HLC14139 | KKCHE025-06 | KF368404 | 58.7640 | -93.8680 |
| *Pardosa fuscula* | I | HLC14151 | KKCHE027-06 | KF368414 | 58.7640 | -93.8680 |
| *Pardosa fuscula* | I | HLC-26837 | KKCHE868-09 | KF368413 | 58.7800 | -94.1860 |
| *Pardosa fuscula* | I | HLC-26849 | KKCHE880-09 | KF368411 | 58.6300 | -93.8190 |
| *Pardosa fuscula* | A | INV0131 | KKCHE032-06 | KF368417 | 58.6320 | -93.7860 |
| *Pardosa fuscula* | I | INV0291 | KKCHE086-06 | KF368418 | 58.6620 | -93.8350 |
| *Pardosa fuscula* | I | JBWM0006136 | KKCHE269-07 | KF368423 | 58.6260 | -94.2280 |
| *Pardosa fuscula* | I | SD2406SG204 | SAPIT122-08 | KF368422 | 58.7300 | -93.8000 |
| *Pardosa fuscula* | I | SD2707SG501 | SAPIT049-08 | KF368416 | 58.7300 | -93.8000 |
| *Pardosa glacialis* | A | BIOUG01969-G03 | SPUCH001-12 | KF368428 | 58.7300 | -93.7950 |
| *Pardosa glacialis* | A | BIOUG01969-G04 | SPUCH002-12 | KF368426 | 58.7300 | -93.7950 |
| *Pardosa glacialis* | A | BIOUG01969-G05 | SPUCH003-12 | KF368427 | 58.7300 | -93.7950 |
| *Pardosa glacialis* | A | BIOUG01969-G06 | SPUCH004-12 | KF368425 | 58.7300 | -93.7950 |
| *Pardosa groenlandica* | A | 07CHU-AR-0223 | KKCHE1030-09 | GU683714 | 58.7630 | -93.8680 |
| *Pardosa groenlandica* | I | 07CHU-AR-0248 | KKCHE1055-09 | GU683732 | 58.6260 | -94.2290 |
| *Pardosa groenlandica* | A | 07CHU-AR-0249 | KKCHE1056-09 | GU683736 | 58.6260 | -94.2290 |
| *Pardosa groenlandica* | A | 07CHU-AR-0263 | KKCHE1070-09 | GU683747 | 58.7700 | -93.8400 |
| *Pardosa groenlandica* | I | 07CHU-AR-0294 | KKCHE1101-09 | GU683601 | 58.6750 | -94.1670 |
| *Pardosa groenlandica* | I | BIOUG00633-A12 | SPIRU1400-11 | KF368431 | 58.7710 | -93.8510 |
| *Pardosa groenlandica* | A | BIOUG00633-B10 | SPIRU1410-11 | KF368430 | 58.7710 | -93.8510 |
| *Pardosa groenlandica* | A | HLC13357 | KKCHE035-06 | KF368432 | 58.7680 | -93.8680 |
| *Pardosa groenlandica* | I | JBWM0006156m | KKCHE273-07 | KF368429 | 58.7850 | -94.2010 |
| *Pardosa hyperborea* | A | 07PROBE-02747 | TWSC134-08 | KF368442 | 58.6300 | -93.8190 |
| *Pardosa hyperborea* | A | 07PROBE-02751 | TWSC138-08 | KF368438 | 58.7380 | -93.8190 |
| *Pardosa hyperborea* | A | 07PROBE-02754 | TWSC141-08 | KF368443 | 58.7380 | -93.8190 |
| *Pardosa hyperborea* | A | 07PROBE-02758 | TWSC145-08 | KF368462 | 58.7380 | -93.8190 |
| *Pardosa hyperborea* | A | 07PROBE-02759 | TWSC146-08 | KF368441 | 58.6300 | -93.8190 |
| *Pardosa hyperborea* | A | 07PROBE-02760 | TWSC147-08 | KF368464 | 58.7570 | -93.9840 |
| *Pardosa hyperborea* | A | 07PROBE-02763 | TWSC150-08 | KF368457 | 58.6300 | -93.8190 |
| *Pardosa hyperborea* | A | 07PROBE-02765 | TWSC152-08 | KF368439 | 58.6300 | -93.8190 |
| *Pardosa hyperborea* | A | 07PROBE-02772 | TWSC159-08 | KF368445 | 58.7380 | -93.8190 |
| *Pardosa hyperborea* | A | 07PROBE-02776 | TWSC162-08 | KF368459 | 58.7380 | -93.8190 |
| *Pardosa hyperborea* | A | 07PROBE-02779 | TWSC165-08 | KF368453 | 58.6300 | -93.8190 |
| *Pardosa hyperborea* | A | 07PROBE-02781 | TWSC167-08 | KF368471 | 58.6300 | -93.8190 |
| *Pardosa hyperborea* | A | 07PROBE-02787 | TWSC173-08 | KF368448 | 58.6300 | -93.8190 |
| *Pardosa hyperborea* | A | 07PROBE-02788 | TWSC174-08 | KF368435 | 58.6300 | -93.8190 |
| *Pardosa hyperborea* | A | 07PROBE-02790 | TWSC176-08 | KF368463 | 58.7050 | -94.0520 |
| *Pardosa hyperborea* | I | 07PROBE-02791 | TWSC177-08 | KF368466 | 58.6300 | -93.8190 |
| *Pardosa hyperborea* | A | 07PROBE-02793 | TWSC179-08 | KF368458 | 58.7570 | -93.9840 |
| *Pardosa hyperborea* | A | 07PROBE-02794 | TWSC180-08 | KF368433 | 58.6300 | -93.8190 |
| *Pardosa hyperborea* | A | 07PROBE-06489 | TWSC003-07 | KF368449 | 58.7570 | -93.9840 |
| *Pardosa hyperborea* | A | 07PROBE-06514 | TWSC028-07 | KF368460 | 58.6300 | -93.8190 |
| *Pardosa hyperborea* | A | 07PROBE-06515 | TWSC029-07 | KF368446 | 58.6300 | -93.8190 |
| *Pardosa hyperborea* | A | 07PROBE-06516 | TWSC030-07 | KF368451 | 58.6300 | -93.8190 |
| *Pardosa hyperborea* | A | 07PROBE-06517 | TWSC031-07 | KF368468 | 58.6300 | -93.8190 |
| *Pardosa hyperborea* | A | 07PROBE-06566 | TWSC080-07 | KF368447 | 58.6300 | -93.8190 |
| *Pardosa hyperborea* | A | 07PROBE-06567 | TWSC081-07 | KF368469 | 58.6300 | -93.8190 |
| *Pardosa hyperborea* | A | 07PROBE-06576 | TWSC090-07 | KF368444 | 58.6300 | -93.8190 |
| *Pardosa hyperborea* | A | 07PROBE-06577 | TWSC091-07 | KF368455 | 58.7050 | -94.0520 |
| *Pardosa hyperborea* | I | 09-PROBE-08099 | JDTGS003-09 | GU679909 | 58.6250 | -93.8190 |
| *Pardosa hyperborea* | I | 09-PROBE-08100 | JDTGS004-09 | GU679910 | 58.6250 | -93.8190 |
| *Pardosa hyperborea* | I | 09-PROBE-08102 | JDTGS006-09 | GU679908 | 58.6250 | -93.8190 |
| *Pardosa hyperborea* | A | BIOUG00629-E06 | SPIRU1216-11 | KF368474 | 58.7660 | -93.8680 |
| *Pardosa hyperborea* | A | BIOUG00629-F10 | SPIRU1232-11 | KF368437 | 58.7720 | -93.8431 |
| *Pardosa hyperborea* | I | BIOUG00629-F11 | SPIRU1233-11 | KF368436 | 58.7720 | -93.8431 |
| *Pardosa hyperborea* | A | BIOUG00633-A07 | SPIRU1395-11 | KF368461 | 58.6329 | -93.7869 |
| *Pardosa hyperborea* | A | BIOUG00633-B05 | SPIRU1405-11 | KF368440 | 58.7720 | -93.8431 |
| *Pardosa hyperborea* | I | CHU-SPI-491 | KKCHE694-07 | KF368454 | 58.7470 | -94.1340 |
| *Pardosa hyperborea* | I | CHU-SPI-514 | KKCHE717-07 | KF368470 | 58.7470 | -94.1340 |
| *Pardosa hyperborea* | I | CHU-SPI-535 | KKCHE738-07 | KF368473 | 58.7380 | -93.8190 |
| *Pardosa hyperborea* | A | CHU-SPI-569 | KKCHE772-07 | KF368467 | 58.7380 | -93.8190 |
| *Pardosa hyperborea* | A | CHU-SPI-582 | KKCHE785-07 | KF368434 | 58.7380 | -93.8190 |
| *Pardosa hyperborea* | I | HLC13355 | KKCHE017-06 | KF368472 | 58.7640 | -93.8680 |
| *Pardosa hyperborea* | I | HLC13433 | KKCHE047-06 | KF368475 | 58.7680 | -93.8680 |
| *Pardosa hyperborea* | I | HLC-26851 | KKCHE882-09 | KF368450 | 58.6300 | -93.8190 |
| *Pardosa hyperborea* | I | HLC-26856 | KKCHE887-09 | KF368452 | 58.6300 | -93.8190 |
| *Pardosa hyperborea* | I | INV0132 | KKCHE055-06 | KF368456 | 58.6320 | -93.7860 |
| *Pardosa hyperborea* | I | JBWM0006145 | KKCHE271-07 | KF368465 | 58.7310 | -93.7800 |
| *Pardosa lapponica* | I | 07CHU-AR-0218 | KKCHE1025-09 | GU683710 | 58.7630 | -93.8850 |
| *Pardosa lapponica* | A | 07CHU-AR-0219 | KKCHE1026-09 | GU683713 | 58.6260 | -94.2300 |
| *Pardosa lapponica* | I | 07CHU-AR-0220 | KKCHE1027-09 | GU683712 | 58.6260 | -94.2300 |
| *Pardosa lapponica* | I | 07CHU-AR-0227 | KKCHE1034-09 | GU683717 | 58.7940 | -94.2170 |
| *Pardosa lapponica* | A | 07PROBE-02708 | TWSC095-08 | KF368529 | 58.7310 | -93.7800 |
| *Pardosa lapponica* | A | 07PROBE-02709 | TWSC096-08 | KF368504 | 58.7570 | -93.9840 |
| *Pardosa lapponica* | A | 07PROBE-02710 | TWSC097-08 | KF368634 | 58.7310 | -93.7800 |
| *Pardosa lapponica* | A | 07PROBE-02711 | TWSC098-08 | KF368545 | 58.7570 | -93.9840 |
| *Pardosa lapponica* | A | 07PROBE-02712 | TWSC099-08 | KF368511 | 58.7600 | -94.0860 |
| *Pardosa lapponica* | A | 07PROBE-02714 | TWSC101-08 | KF368481 | 58.7570 | -93.9840 |
| *Pardosa lapponica* | A | 07PROBE-02715 | TWSC102-08 | KF368519 | 58.6300 | -93.8190 |
| *Pardosa lapponica* | A | 07PROBE-02717 | TWSC104-08 | KF368495 | 58.7600 | -94.0860 |
| *Pardosa lapponica* | A | 07PROBE-02718 | TWSC105-08 | KF368570 | 58.7050 | -94.0520 |
| *Pardosa lapponica* | A | 07PROBE-02719 | TWSC106-08 | KF368484 | 58.7600 | -94.0860 |
| *Pardosa lapponica* | A | 07PROBE-02720 | TWSC107-08 | KF368615 | 58.7600 | -94.0860 |
| *Pardosa lapponica* | A | 07PROBE-02721 | TWSC108-08 | KF368518 | 58.7310 | -93.7800 |
| *Pardosa lapponica* | A | 07PROBE-02722 | TWSC109-08 | KF368587 | 58.7050 | -94.0520 |
| *Pardosa lapponica* | A | 07PROBE-02723 | TWSC110-08 | KF368558 | 58.7050 | -94.0520 |
| *Pardosa lapponica* | A | 07PROBE-02724 | TWSC111-08 | KF368617 | 58.7300 | -93.7840 |
| *Pardosa lapponica* | A | 07PROBE-02725 | TWSC112-08 | KF368624 | 58.7600 | -94.0860 |
| *Pardosa lapponica* | A | 07PROBE-02726 | TWSC113-08 | KF368582 | 58.7600 | -94.0860 |
| *Pardosa lapponica* | A | 07PROBE-02727 | TWSC114-08 | KF368630 | 58.7050 | -94.0520 |
| *Pardosa lapponica* | A | 07PROBE-02728 | TWSC115-08 | KF368599 | 58.7600 | -94.0860 |
| *Pardosa lapponica* | A | 07PROBE-02729 | TWSC116-08 | KF368611 | 58.7600 | -94.0860 |
| *Pardosa lapponica* | A | 07PROBE-02731 | TWSC118-08 | KF368637 | 58.7050 | -94.0520 |
| *Pardosa lapponica* | A | 07PROBE-02732 | TWSC119-08 | KF368533 | 58.7050 | -94.0520 |
| *Pardosa lapponica* | A | 07PROBE-02733 | TWSC120-08 | KF368485 | 58.7050 | -94.0520 |
| *Pardosa lapponica* | A | 07PROBE-02734 | TWSC121-08 | KF368500 | 58.7050 | -94.0520 |
| *Pardosa lapponica* | A | 07PROBE-02735 | TWSC122-08 | KF368563 | 58.7310 | -93.7800 |
| *Pardosa lapponica* | A | 07PROBE-02737 | TWSC124-08 | KF368628 | 58.7380 | -93.8190 |
| *Pardosa lapponica* | A | 07PROBE-02738 | TWSC125-08 | KF368534 | 58.7050 | -94.0520 |
| *Pardosa lapponica* | A | 07PROBE-02740 | TWSC127-08 | KF368596 | 58.7380 | -93.8190 |
| *Pardosa lapponica* | A | 07PROBE-02741 | TWSC128-08 | KF368537 | 58.7600 | -94.0860 |
| *Pardosa lapponica* | A | 07PROBE-02742 | TWSC129-08 | KF368556 | 58.7050 | -94.0520 |
| *Pardosa lapponica* | A | 07PROBE-02743 | TWSC130-08 | KF368625 | 58.7050 | -94.0520 |
| *Pardosa lapponica* | A | 07PROBE-02744 | TWSC131-08 | KF368600 | 58.7570 | -93.9840 |
| *Pardosa lapponica* | A | 07PROBE-02746 | TWSC133-08 | KF368479 | 58.7600 | -94.0860 |
| *Pardosa lapponica* | A | 07PROBE-02756 | TWSC143-08 | KF368523 | 58.7380 | -93.8190 |
| *Pardosa lapponica* | A | 07PROBE-02757 | TWSC144-08 | KF368621 | 58.7050 | -94.0520 |
| *Pardosa lapponica* | A | 07PROBE-02762 | TWSC149-08 | KF368536 | 58.7600 | -94.0860 |
| *Pardosa lapponica* | A | 07PROBE-02768 | TWSC155-08 | KF368551 | 58.7600 | -94.0860 |
| *Pardosa lapponica* | I | 07PROBE-02773 | TWSC160-08 | KF368539 | 58.7310 | -93.7800 |
| *Pardosa lapponica* | I | 07PROBE-02774 | TWSC161-08 | KF368577 | 58.7050 | -94.0520 |
| *Pardosa lapponica* | I | 07PROBE-02778 | TWSC164-08 | KF368586 | 58.7310 | -93.7800 |
| *Pardosa lapponica* | A | 07PROBE-02782 | TWSC168-08 | KF368516 | 58.7600 | -94.0860 |
| *Pardosa lapponica* | A | 07PROBE-02784 | TWSC170-08 | KF368593 | 58.7600 | -94.0860 |
| *Pardosa lapponica* | I | 07PROBE-02792 | TWSC178-08 | KF368604 | 58.7600 | -94.0860 |
| *Pardosa lapponica* | I | 07PROBE-02796 | TWSC182-08 | KF368607 | 58.7300 | -93.7800 |
| *Pardosa lapponica* | A | 07PROBE-02801 | TWSC187-08 | KF368548 | 58.7600 | -94.0860 |
| *Pardosa lapponica* | A | 07PROBE-02802 | TWSC188-08 | KF368547 | 58.7600 | -94.0860 |
| *Pardosa lapponica* | A | 07PROBE-02817 | TWSC203-08 | KF368568 | 58.7050 | -94.0520 |
| *Pardosa lapponica* | I | 07PROBE-02818 | TWSC204-08 | KF368606 | 58.7600 | -94.0860 |
| *Pardosa lapponica* | A | 07PROBE-02825 | TWSC211-08 | KF368535 | 58.7600 | -94.0860 |
| *Pardosa lapponica* | A | 07PROBE-02826 | TWSC212-08 | KF368619 | 58.7600 | -94.0860 |
| *Pardosa lapponica* | A | 07PROBE-02832 | TWSC218-08 | KF368486 | 58.7600 | -94.0860 |
| *Pardosa lapponica* | A | 07PROBE-02833 | TWSC219-08 | KF368608 | 58.7600 | -94.0860 |
| *Pardosa lapponica* | I | 07PROBE-02835 | TWSC221-08 | KF368524 | 58.7300 | -93.7840 |
| *Pardosa lapponica* | I | 07PROBE-02837 | TWSC223-08 | KF368553 | 58.7600 | -94.0860 |
| *Pardosa lapponica* | I | 07PROBE-02839 | TWSC225-08 | KF368578 | 58.7300 | -93.7840 |
| *Pardosa lapponica* | I | 07PROBE-02841 | TWSC227-08 | KF368499 | 58.7600 | -94.0860 |
| *Pardosa lapponica* | A | 07PROBE-02844 | TWSC230-08 | KF368546 | 58.7600 | -94.0860 |
| *Pardosa lapponica* | A | 07PROBE-02846 | TWSC232-08 | KF368525 | 58.7600 | -94.0860 |
| *Pardosa lapponica* | I | 07PROBE-02849 | TWSC235-08 | KF368527 | 58.7300 | -93.7840 |
| *Pardosa lapponica* | A | 07PROBE-02850 | TWSC236-08 | KF368554 | 58.7600 | -94.0860 |
| *Pardosa lapponica* | A | 07PROBE-02851 | TWSC237-08 | KF368550 | 58.7600 | -94.0860 |
| *Pardosa lapponica* | A | 07PROBE-02855 | TWSC241-08 | KF368623 | 58.7600 | -94.0860 |
| *Pardosa lapponica* | I | 07PROBE-02860 | TWSC246-08 | KF368602 | 58.7600 | -94.0860 |
| *Pardosa lapponica* | I | 07PROBE-02861 | TWSC247-08 | KF368490 | 58.7910 | -93.7540 |
| *Pardosa lapponica* | I | 07PROBE-02862 | TWSC248-08 | KF368478 | 58.7770 | -93.7360 |
| *Pardosa lapponica* | I | 07PROBE-02863 | TWSC249-08 | KF368497 | 58.7770 | -93.7360 |
| *Pardosa lapponica* | I | 07PROBE-02864 | TWSC250-08 | KF368522 | 58.7770 | -93.7360 |
| *Pardosa lapponica* | A | 07PROBE-02866 | TWSC252-08 | KF368480 | 58.7910 | -93.7540 |
| *Pardosa lapponica* | I | 07PROBE-02867 | TWSC253-08 | KF368543 | 58.7770 | -93.7360 |
| *Pardosa lapponica* | I | 07PROBE-02869 | TWSC255-08 | KF368592 | 58.7770 | -93.7360 |
| *Pardosa lapponica* | I | 07PROBE-02870 | TWSC256-08 | KF368502 | 58.7910 | -93.7540 |
| *Pardosa lapponica* | I | 07PROBE-02871 | TWSC257-08 | KF368569 | 58.7770 | -93.7360 |
| *Pardosa lapponica* | I | 07PROBE-02874 | TWSC260-08 | KF368581 | 58.7770 | -93.7360 |
| *Pardosa lapponica* | A | 07PROBE-02875 | TWSC261-08 | KF368488 | 58.7910 | -93.7540 |
| *Pardosa lapponica* | I | 07PROBE-02876 | TWSC262-08 | KF368564 | 58.7910 | -93.7540 |
| *Pardosa lapponica* | A | 07PROBE-02877 | TWSC263-08 | KF368521 | 58.7910 | -93.7540 |
| *Pardosa lapponica* | A | 07PROBE-02878 | TWSC264-08 | KF368632 | 58.7910 | -93.7540 |
| *Pardosa lapponica* | I | 07PROBE-02879 | TWSC265-08 | KF368526 | 58.7600 | -94.0860 |
| *Pardosa lapponica* | A | 07PROBE-02881 | TWSC267-08 | KF368542 | 58.7600 | -94.0860 |
| *Pardosa lapponica* | I | 07PROBE-02884 | TWSC270-08 | KF368595 | 58.7600 | -94.0860 |
| *Pardosa lapponica* | A | 07PROBE-02887 | TWSC273-08 | KF368583 | 58.7600 | -94.0860 |
| *Pardosa lapponica* | A | 07PROBE-02888 | TWSC274-08 | KF368531 | 58.7600 | -94.0860 |
| *Pardosa lapponica* | I | 07PROBE-02890 | TWSC276-08 | KF368544 | 58.7600 | -94.0860 |
| *Pardosa lapponica* | A | 07PROBE-02891 | TWSC277-08 | KF368540 | 58.7600 | -94.0860 |
| *Pardosa lapponica* | I | 07PROBE-06488 | TWSC002-07 | KF368560 | 58.7050 | -94.0520 |
| *Pardosa lapponica* | I | 07PROBE-06497 | TWSC011-07 | KF368603 | 58.7310 | -93.7800 |
| *Pardosa lapponica* | I | 07PROBE-06500 | TWSC014-07 | KF368571 | 58.7050 | -94.0520 |
| *Pardosa lapponica* | A | 07PROBE-06519 | TWSC033-07 | KF368626 | 58.7600 | -94.0860 |
| *Pardosa lapponica* | A | 07PROBE-06541 | TWSC055-07 | KF368557 | 58.7310 | -93.8150 |
| *Pardosa lapponica* | A | 07PROBE-06542 | TWSC056-07 | KF368532 | 58.7570 | -93.9840 |
| *Pardosa lapponica* | A | 07PROBE-06543 | TWSC057-07 | KF368530 | 58.7050 | -94.0520 |
| *Pardosa lapponica* | A | 07PROBE-06544 | TWSC058-07 | KF368528 | 58.7570 | -93.9840 |
| *Pardosa lapponica* | A | 07PROBE-06545 | TWSC059-07 | KF368566 | 58.7600 | -94.0860 |
| *Pardosa lapponica* | A | 07PROBE-06547 | TWSC061-07 | KF368598 | 58.7600 | -94.0860 |
| *Pardosa lapponica* | A | 07PROBE-06558 | TWSC072-07 | KF368514 | 58.7050 | -94.0520 |
| *Pardosa lapponica* | I | 07PROBE-06563 | TWSC077-07 | KF368517 | 58.7310 | -93.8150 |
| *Pardosa lapponica* | A | 07PROBE-06564 | TWSC078-07 | KF368496 | 58.6170 | -93.8140 |
| *Pardosa lapponica* | I | 07PROBE-06565 | TWSC079-07 | KF368590 | 58.7860 | -94.2020 |
| *Pardosa lapponica* | I | 07PROBE-06568 | TWSC082-07 | KF368503 | 58.7600 | -94.0860 |
| *Pardosa lapponica* | I | 07PROBE-06570 | TWSC084-07 | KF368579 | 58.7310 | -93.8150 |
| *Pardosa lapponica* | I | 07PROBE-06573 | TWSC087-07 | KF368580 | 58.7600 | -94.0860 |
| *Pardosa lapponica* | I | 07PROBE-06574 | TWSC088-07 | KF368612 | 58.7310 | -93.7800 |
| *Pardosa lapponica* | I | 07PROBE-06578 | TWSC092-07 | KF368589 | 58.7600 | -94.0860 |
| *Pardosa lapponica* | I | 09PROBE-1774-01 | SPICH196-09 | GU683788 | 58.6300 | -93.8190 |
| *Pardosa lapponica* | I | 09PROBE-1774-02 | SPICH197-09 | GU683789 | 58.6300 | -93.8190 |
| *Pardosa lapponica* | I | 09PROBE-1978-480 | SPICH723-09 | GU684393 | 58.7640 | -93.8970 |
| *Pardosa lapponica* | I | 09PROBE-636 | SPICH879-09 | GU684424 | 58.7900 | -94.2270 |
| *Pardosa lapponica* | I | 09PROBE-637 | SPICH880-09 | KF368512 | 58.7900 | -94.2270 |
| *Pardosa lapponica* | I | 09PROBE-638 | SPICH881-09 | GU684412 | 58.7900 | -94.2270 |
| *Pardosa lapponica* | I | 09PROBE-698 | SPICH941-09 | GU684510 | 58.7900 | -94.2270 |
| *Pardosa lapponica* | A | 09PROBE-700 | SPICH943-09 | GU684509 | 58.7900 | -94.2270 |
| *Pardosa lapponica* | A | 09PROBE-706 | SPICH949-09 | GU684507 | 58.7900 | -94.2270 |
| *Pardosa lapponica* | A | 09PROBE-707 | SPICH950-09 | GU684506 | 58.7900 | -94.2270 |
| *Pardosa lapponica* | A | 09PROBE-708 | SPICH951-09 | KF368505 | 58.7900 | -94.2270 |
| *Pardosa lapponica* | A | 09PROBE-709 | SPICH952-09 | GU684508 | 58.7900 | -94.2270 |
| *Pardosa lapponica* | A | BIOUG00628-A10 | SPIRU1077-11 | KF368575 | 58.7616 | -93.9295 |
| *Pardosa lapponica* | A | BIOUG00629-D12 | SPIRU1210-11 | KF368501 | 58.7655 | -93.8676 |
| *Pardosa lapponica* | A | BIOUG00629-E05 | SPIRU1215-11 | KF368562 | 58.7660 | -93.8680 |
| *Pardosa lapponica* | A | BIOUG00629-F06 | SPIRU1228-11 | KF368635 | 58.7710 | -93.8510 |
| *Pardosa lapponica* | A | BIOUG00629-F07 | SPIRU1229-11 | KF368487 | 58.7710 | -93.8510 |
| *Pardosa lapponica* | A | BIOUG00630-B11 | SPIRU1280-11 | KF368507 | 58.7710 | -93.8510 |
| *Pardosa lapponica* | A | BIOUG00633-B01 | SPIRU1401-11 | KF368622 | 58.7710 | -93.8510 |
| *Pardosa lapponica* | A | BIOUG00633-B02 | SPIRU1402-11 | KF368633 | 58.7710 | -93.8510 |
| *Pardosa lapponica* | A | BIOUG00633-C01 | SPIRU1413-11 | KF368520 | 58.7710 | -93.8510 |
| *Pardosa lapponica* | I | CHU-SPI-157 | KKCHE360-07 | KF368513 | 58.6170 | -93.8210 |
| *Pardosa lapponica* | I | CHU-SPI-393 | KKCHE596-07 | KF368552 | 58.7380 | -93.8190 |
| *Pardosa lapponica* | I | CHU-SPI-394 | KKCHE597-07 | KF368498 | 58.7380 | -93.8190 |
| *Pardosa lapponica* | I | CHU-SPI-398 | KKCHE601-07 | KF368549 | 58.7380 | -93.8190 |
| *Pardosa lapponica* | I | CHU-SPI-432 | KKCHE635-07 | KF368584 | 58.7380 | -93.8190 |
| *Pardosa lapponica* | I | CHU-SPI-434 | KKCHE637-07 | KF368492 | 58.7380 | -93.8190 |
| *Pardosa lapponica* | I | CHU-SPI-445 | KKCHE648-07 | KF368616 | 58.7380 | -93.8190 |
| *Pardosa lapponica* | A | CHU-SPI-454 | KKCHE657-07 | KF368588 | 58.7380 | -93.8190 |
| *Pardosa lapponica* | I | CHU-SPI-458 | KKCHE661-07 | KF368493 | 58.7380 | -93.8190 |
| *Pardosa lapponica* | A | CHU-SPI-461 | KKCHE664-07 | KF368629 | 58.7050 | -94.0520 |
| *Pardosa lapponica* | I | CHU-SPI-469 | KKCHE672-07 | KF368631 | 58.7380 | -93.8190 |
| *Pardosa lapponica* | I | CHU-SPI-477 | KKCHE680-07 | KF368572 | 58.7380 | -93.8190 |
| *Pardosa lapponica* | I | CHU-SPI-481 | KKCHE684-07 | KF368555 | 58.7380 | -93.8190 |
| *Pardosa lapponica* | I | CHU-SPI-493 | KKCHE696-07 | KF368639 | 58.7470 | -94.1340 |
| *Pardosa lapponica* | I | CHU-SPI-503 | KKCHE706-07 | KF368494 | 58.7470 | -94.1340 |
| *Pardosa lapponica* | I | CHU-SPI-504 | KKCHE707-07 | KF368594 | 58.7470 | -94.1340 |
| *Pardosa lapponica* | I | CHU-SPI-515 | KKCHE718-07 | KF368508 | 58.7470 | -94.1340 |
| *Pardosa lapponica* | I | CHU-SPI-517 | KKCHE720-07 | KF368482 | 58.7470 | -94.1340 |
| *Pardosa lapponica* | I | CHU-SPI-518 | KKCHE721-07 | KF368574 | 58.7470 | -94.1340 |
| *Pardosa lapponica* | I | CHU-SPI-525 | KKCHE728-07 | KF368576 | 58.7470 | -94.1340 |
| *Pardosa lapponica* | A | CHU-SPI-530 | KKCHE733-07 | KF368510 | 58.7470 | -94.1340 |
| *Pardosa lapponica* | I | CHU-SPI-537 | KKCHE740-07 | KF368515 | 58.7470 | -94.1340 |
| *Pardosa lapponica* | I | CHU-SPI-549 | KKCHE752-07 | KF368541 | 58.7470 | -94.1340 |
| *Pardosa lapponica* | I | CHU-SPI-553 | KKCHE756-07 | KF368565 | 58.7470 | -94.1340 |
| *Pardosa lapponica* | A | CHU-SPI-554 | KKCHE757-07 | KF368620 | 58.7470 | -94.1340 |
| *Pardosa lapponica* | A | CHU-SPI-574 | KKCHE777-07 | KF368559 | 58.7470 | -94.1340 |
| *Pardosa lapponica* | I | HLC13418 | KKCHE041-06 | KF368597 | 58.7680 | -93.8680 |
| *Pardosa lapponica* | I | HLC-26799 | KKCHE830-09 | KF368506 | 58.7800 | -94.1860 |
| *Pardosa lapponica* | I | HLC-26804 | KKCHE835-09 | KF368601 | 58.7800 | -94.1860 |
| *Pardosa lapponica* | A | HLC-26806 | KKCHE837-09 | KF368476 | 58.7800 | -94.1860 |
| *Pardosa lapponica* | I | HLC-26808 | KKCHE839-09 | KF368614 | 58.7800 | -94.1860 |
| *Pardosa lapponica* | I | HLC-26810 | KKCHE841-09 | KF368561 | 58.7800 | -94.1860 |
| *Pardosa lapponica* | I | HLC-26812 | KKCHE843-09 | KF368567 | 58.7800 | -94.1860 |
| *Pardosa lapponica* | I | HLC-26814 | KKCHE845-09 | KF368585 | 58.7800 | -94.1860 |
| *Pardosa lapponica* | I | HLC-26816 | KKCHE847-09 | KF368638 | 58.7800 | -94.1860 |
| *Pardosa lapponica* | I | HLC-26824 | KKCHE855-09 | KF368605 | 58.7800 | -94.1860 |
| *Pardosa lapponica* | I | HLC-26827 | KKCHE858-09 | KF368613 | 58.7800 | -94.1860 |
| *Pardosa lapponica* | I | HLC-26828 | KKCHE859-09 | KF368609 | 58.7800 | -94.1860 |
| *Pardosa lapponica* | I | HLC-26830 | KKCHE861-09 | KF368509 | 58.7800 | -94.1860 |
| *Pardosa lapponica* | I | HLC-26839 | KKCHE870-09 | KF368610 | 58.7800 | -94.1860 |
| *Pardosa lapponica* | I | HLC-26840 | KKCHE871-09 | KF368627 | 58.7800 | -94.1860 |
| *Pardosa lapponica* | I | HLC-26875 | KKCHE906-09 | KF368573 | 58.7310 | -93.7800 |
| *Pardosa lapponica* | I | HLC-26877 | KKCHE908-09 | KF368538 | 58.7310 | -93.7800 |
| *Pardosa lapponica* | I | INV0286 | KKCHE074-06 | KF368636 | 58.6620 | -93.8350 |
| *Pardosa lapponica* | I | JBWM0234317f | KKCHE300-07 | KF368489 | 58.7540 | -93.9130 |
| *Pardosa lapponica* | A | JBWM0234317m | KKCHE299-07 | KF368483 | 58.7540 | -93.9130 |
| *Pardosa lapponica* | A | SD2406SH302 | SAPIT154-08 | KF368618 | 58.7300 | -93.8000 |
| *Pardosa lapponica* | I | SD2707HE501 | SAPIT058-08 | KF368477 | 58.7300 | -93.8000 |
| *Pardosa lapponica* | I | SD2707HE502 | SAPIT059-08 | KF368591 | 58.7300 | -93.8000 |
| *Pardosa lapponica* | I | SD2707HE601 | SAPIT025-08 | KF368640 | 58.7300 | -93.8000 |
| *Pardosa lapponica* | A | SD2707SG211 | SAPIT083-08 | KF368491 | 58.7300 | -93.8000 |
| *Pardosa moesta* | I | 09PROBE-1771-01 | SPICH191-09 | GU683790 | 58.6260 | -94.2300 |
| *Pardosa moesta* | I | 09PROBE-1771-02 | SPICH192-09 | GU683792 | 58.6260 | -94.2300 |
| *Pardosa moesta* | I | 09PROBE-1771-03 | SPICH193-09 | GU683791 | 58.6260 | -94.2300 |
| *Pardosa moesta* | A | 09PROBE-1771-04 | SPICH194-09 | GU683793 | 58.6260 | -94.2300 |
| *Pardosa moesta* | A | 09PROBE-1927-01 | SPICH074-09 | GU683900 | 58.6180 | -93.8290 |
| *Pardosa moesta* | A | 09PROBE-948 | SPICH1169-09 | GU684597 | 58.6260 | -94.2300 |
| *Pardosa moesta* | A | 09PROBE-949 | SPICH1170-09 | GU684599 | 58.6260 | -94.2300 |
| *Pardosa moesta* | A | 09PROBE-950 | SPICH1171-09 | GU684598 | 58.6260 | -94.2300 |
| *Pardosa podhorskii* | A | BIOUG00629-H01 | SPIRU1247-11 | KF368642 | 58.7710 | -93.8510 |
| *Pardosa podhorskii* | I | BIOUG00633-C03 | SPIRU1415-11 | KF368641 | 58.7710 | -93.8510 |
| *Pardosa uintana* | A | 07PROBE-02716 | TWSC103-08 | KF368646 | 58.6300 | -93.8190 |
| *Pardosa uintana* | A | 07PROBE-02750 | TWSC137-08 | KF368644 | 58.6170 | -93.8120 |
| *Pardosa uintana* | I | 07PROBE-06518 | TWSC032-07 | KF368645 | 58.6300 | -93.8190 |
| *Pardosa uintana* | I | 09-PROBE-08101 | JDTGS005-09 | GU679907 | 58.6250 | -93.8190 |
| *Pardosa uintana* | I | 09-PROBE-08107 | JDTGS011-09 | GU679901 | 58.6250 | -93.8190 |
| *Pardosa uintana* | I | 09-PROBE-08155 | JDTGS059-09 | GU679866 | 58.6180 | -93.8290 |
| *Pardosa uintana* | A | 09PROBE-943 | SPICH1164-09 | GU684596 | 58.6180 | -93.8290 |
| *Pardosa uintana* | I | CHU-SPI-534 | KKCHE737-07 | KF368649 | 58.7380 | -93.8190 |
| *Pardosa uintana* | I | HLC13306 | KKCHE048-06 | KF368651 | 58.7680 | -93.8680 |
| *Pardosa uintana* | I | HLC-26847 | KKCHE878-09 | KF368648 | 58.6300 | -93.8190 |
| *Pardosa uintana* | I | INV0126 | KKCHE031-06 | KF368643 | 58.6320 | -93.7860 |
| *Pardosa uintana* | I | INV0363 | KKCHE030-06 | KF368647 | 58.7660 | -93.8680 |
| *Pardosa uintana* | I | JBWM0006153 | KKCHE272-07 | KF368650 | 58.7380 | -93.8190 |
| *Pelecopsis mengei* | A | 09PROBE-1811-01 | SPICH223-09 | GU683971 | 58.6300 | -93.8190 |
| *Pelecopsis mengei* | I | 09PROBE-1978-330 | SPICH573-09 | GU684163 | 58.6690 | -94.1600 |
| *Pelecopsis mengei* | I | 09PROBE-743 | SPICH986-09 | GU684468 | 58.6260 | -94.2300 |
| *Pelegrina montana* | A | BIOUG00627-E12 | SPIRU1032-11 | KF368652 | 58.6192 | -93.8291 |
| *Pellenes montanus* | A | 09PROBE-1645-01 | SPISH009-09 | GU683925 | 58.6180 | -93.8290 |
| *Philodromus alascensis* | A | 07CHU-AR-0264 | KKCHE1071-09 | GU683746 | 58.6340 | -93.7860 |
| *Philodromus alascensis* | A | 07CHU-AR-0265 | KKCHE1072-09 | GU683751 | 58.6340 | -93.7860 |
| *Philodromus alascensis* | I | 07PROBE-04626 | ERSCH081-07 | KF368653 | 58.6300 | -93.7980 |
| *Philodromus alascensis* | I | 09-PROBE-08129 | JDTGS033-09 | GU679888 | 58.6250 | -93.8190 |
| *Philodromus alascensis* | I | 09-PROBE-08148 | JDTGS052-09 | GU679872 | 58.6180 | -93.8290 |
| *Philodromus alascensis* | A | 09PROBE-1684-01 | SPICH119-09 | GU683824 | 58.7630 | -93.8660 |
| *Philodromus alascensis* | I | 09PROBE-1692-01 | SPICH134-09 | GU683822 | 58.6300 | -93.7980 |
| *Philodromus alascensis* | A | 09PROBE-1978-345 | SPICH588-09 | GU684172 | 58.6260 | -94.2300 |
| *Philodromus alascensis* | A | 09PROBE-584 | SPICH827-09 | GU684416 | 58.6180 | -93.8290 |
| *Philodromus alascensis* | I | 09PROBE-780 | SPICH1001-09 | GU684743 | 58.6300 | -93.7980 |
| *Philodromus alascensis* | A | 09PROBE-781 | SPICH1002-09 | GU684741 | 58.6300 | -93.7980 |
| *Philodromus alascensis* | A | 09PROBE-782 | SPICH1003-09 | GU684738 | 58.6300 | -93.7980 |
| *Philodromus alascensis* | A | 09PROBE-784 | SPICH1005-09 | GU684740 | 58.6300 | -93.7980 |
| *Philodromus alascensis* | A | BIOUG00627-G11 | SPIRU1055-11 | KF368656 | 58.7304 | -93.7805 |
| *Philodromus alascensis* | I | BIOUG00628-C05 | SPIRU1096-11 | KF368657 | 58.6761 | -94.1442 |
| *Philodromus alascensis* | I | CHU-SPI-108 | KKCHE311-07 | KF368655 | 58.6170 | -93.8210 |
| *Philodromus alascensis* | I | JBWM0006187 | KKCHE275-07 | KF368654 | 58.6170 | -93.8170 |
| *Philodromus histrio* | I | 07CHU-AR-0229 | KKCHE1036-09 | GU683720 | 58.7680 | -94.1750 |
| *Philodromus histrio* | I | BIOUG00627-G09 | SPIRU1053-11 | KF368658 | 58.7614 | -94.0115 |
| *Philodromus peninsulanus* | I | 09-PROBE-08160 | JDTGS064-09 | GU679865 | 58.6250 | -93.8190 |
| *Philodromus peninsulanus* | A | 09PROBE-1658-01 | SPISH013-09 | GU683919 | 58.6180 | -93.8290 |
| *Philodromus peninsulanus* | A | 09PROBE-1658-02 | SPISH014-09 | GU683920 | 58.6180 | -93.8290 |
| *Philodromus peninsulanus* | I | 09PROBE-757 | SPICH1000-09 | GU684463 | 58.6160 | -93.8080 |
| *Philodromus peninsulanus* | A | 09PROBE-783 | SPICH1004-09 | GU684742 | 58.6300 | -93.7980 |
| *Philodromus peninsulanus* | I | CHU-SPI-008 | KKCHE117-06 | KF368660 | 58.6220 | -93.8100 |
| *Philodromus peninsulanus* | I | CHU-SPI-107 | KKCHE310-07 | KF368659 | 58.6170 | -93.8210 |
| *Phlattothrata parva* | I | 09PROBE-1735-01 | SPICH174-09 | GU684008 | 58.7640 | -93.8970 |
| *Phlattothrata parva* | A | 09PROBE-1925-01 | SPICH073-09 | GU683881 | 58.6180 | -93.8290 |
| *Phlattothrata parva* | A | 09PROBE-1945-01 | SPICH084-09 | GU683882 | 58.7640 | -93.8970 |
| *Phlattothrata parva* | A | BIOUG00630-B03 | SPIRU1272-11 | KF368661 | 58.7710 | -93.8510 |
| *Phylloneta impressa* | A | BIOUG00627-A05 | SPIRU977-11 | KF368663 | 58.6921 | -94.1320 |
| *Phylloneta impressa* | A | BIOUG00627-A06 | SPIRU978-11 | KF368662 | 58.6921 | -94.1320 |
| *Pirata bryantae* | A | 09PROBE-1546-01 | SPISH002-09 | HM432633 | 58.7310 | -93.7800 |
| *Pirata piraticus* | I | 07CHU-AR-0035 | KKCHE947-09 | GU683649 | 58.7610 | -93.9520 |
| *Pirata piraticus* | I | 07CHU-AR-0047 | KKCHE959-09 | GU683663 | 58.6300 | -93.7980 |
| *Pirata piraticus* | I | 07CHU-AR-0084 | KKCHE996-09 | GU683688 | 58.7550 | -93.9990 |
| *Pirata piraticus* | I | 07PROBE-02853 | TWSC239-08 | KF368665 | 58.7300 | -93.7800 |
| *Pirata piraticus* | A | 07PROBE-06561 | TWSC075-07 | KF368670 | 58.7570 | -93.9840 |
| *Pirata piraticus* | A | 09PROBE-1680-01 | SPICH109-09 | GU683795 | 58.7610 | -93.9540 |
| *Pirata piraticus* | A | 09PROBE-1680-02 | SPICH110-09 | GU683794 | 58.7610 | -93.9540 |
| *Pirata piraticus* | A | 09PROBE-1680-04 | SPICH112-09 | GU683796 | 58.7610 | -93.9540 |
| *Pirata piraticus* | A | 09PROBE-1680-05 | SPICH113-09 | GU683797 | 58.7610 | -93.9540 |
| *Pirata piraticus* | A | 09PROBE-1680-06 | SPICH114-09 | GU683799 | 58.7610 | -93.9540 |
| *Pirata piraticus* | I | 09PROBE-655 | SPICH898-09 | GU684423 | 58.7870 | -93.7140 |
| *Pirata piraticus* | A | 09PROBE-856 | SPICH1077-09 | GU684594 | 58.6630 | -94.1670 |
| *Pirata piraticus* | A | 09PROBE-945 | SPICH1166-09 | GU684595 | 58.6180 | -93.8290 |
| *Pirata piraticus* | A | BIOUG00628-G09 | SPIRU1148-11 | KF368666 | 58.7304 | -93.7805 |
| *Pirata piraticus* | A | BIOUG00628-G10 | SPIRU1149-11 | KF368667 | 58.7304 | -93.7805 |
| *Pirata piraticus* | A | BIOUG00629-C03 | SPIRU1189-11 | KF368669 | 58.7179 | -94.1221 |
| *Pirata piraticus* | I | CHU-SPI-383 | KKCHE581-07 | KF368668 | 58.6180 | -93.8230 |
| *Pirata piraticus* | I | JBWM0006186 | KKCHE274-07 | KF368664 | 58.6260 | -94.2280 |
| *Piratula canadensis* | I | SD0406SG203 | SAPIT172-08 | KF368671 | 58.7300 | -93.8000 |
| *Pityohyphantes cristatus* | I | 07PROBE-04548 | ERSCH003-07 | KF368677 | 58.6170 | -93.8140 |
| *Pityohyphantes cristatus* | I | 07PROBE-04566 | ERSCH021-07 | KF368682 | 58.7310 | -93.7800 |
| *Pityohyphantes cristatus* | I | 09-PROBE-08162 | JDTGS066-09 | GU679863 | 58.6250 | -93.8190 |
| *Pityohyphantes cristatus* | I | 09-PROBE-08173 | JDTGS077-09 | GU679852 | 58.6180 | -93.8290 |
| *Pityohyphantes cristatus* | A | 09PROBE-910 | SPICH1131-09 | GU684664 | 58.6300 | -93.7980 |
| *Pityohyphantes cristatus* | A | BIOUG00628-A06 | SPIRU1073-11 | KF368674 | 58.6750 | -93.8416 |
| *Pityohyphantes cristatus* | A | BIOUG00628-F11 | SPIRU1138-11 | KF368685 | 58.6350 | -93.7999 |
| *Pityohyphantes cristatus* | A | BIOUG00629-B03 | SPIRU1177-11 | KF368681 | 58.7306 | -93.7804 |
| *Pityohyphantes cristatus* | A | BIOUG00629-B04 | SPIRU1178-11 | KF368683 | 58.7306 | -93.7804 |
| *Pityohyphantes cristatus* | I | CHU-SPI-124 | KKCHE327-07 | KF368679 | 58.6180 | -93.8230 |
| *Pityohyphantes cristatus* | A | CHU-SPI-148 | KKCHE351-07 | KF368688 | 58.6220 | -93.8100 |
| *Pityohyphantes cristatus* | I | CHU-SPI-159 | KKCHE362-07 | KF368676 | 58.6180 | -93.8230 |
| *Pityohyphantes cristatus* | I | CHU-SPI-160 | KKCHE363-07 | KF368675 | 58.6220 | -93.8100 |
| *Pityohyphantes cristatus* | I | CHU-SPI-195 | KKCHE398-07 | KF368689 | 58.6180 | -93.8230 |
| *Pityohyphantes cristatus* | I | CHU-SPI-208 | KKCHE411-07 | KF368672 | 58.6910 | -94.1790 |
| *Pityohyphantes cristatus* | I | CHU-SPI-233 | KKCHE436-07 | KF368684 | 58.6910 | -94.1790 |
| *Pityohyphantes cristatus* | I | CHU-SPI-246 | KKCHE449-07 | KF368687 | 58.6910 | -94.1790 |
| *Pityohyphantes cristatus* | A | CHU-SPI-258 | KKCHE461-07 | KF368686 | 58.6910 | -94.1790 |
| *Pityohyphantes cristatus* | I | CHU-SPI-281 | KKCHE484-07 | KF368690 | 58.6910 | -94.1790 |
| *Pityohyphantes cristatus* | I | CHU-SPI-292 | KKCHE495-07 | KF368673 | 58.6910 | -94.1790 |
| *Pityohyphantes cristatus* | A | CHU-SPI-451 | KKCHE654-07 | KF368680 | 58.7540 | -93.9130 |
| *Pityohyphantes cristatus* | A | JBWM0006084 | KKCHE259-07 | KF368678 | 58.6290 | -93.7980 |
| *Pityohyphantes limitaneus* | A | 07PROBE-04575 | ERSCH030-07 | KF368714 | 58.7310 | -93.7800 |
| *Pityohyphantes limitaneus* | I | 07PROBE-04632 | ERSCH087-07 | KF368698 | 58.6300 | -93.7980 |
| *Pityohyphantes limitaneus* | I | 07PROBE-04736 | ERSCH101-07 | KF368710 | 58.7350 | -94.1090 |
| *Pityohyphantes limitaneus* | A | 07PROBE-04739 | ERSCH104-07 | KF368716 | 58.7350 | -94.1090 |
| *Pityohyphantes limitaneus* | I | 07PROBE-04741 | ERSCH106-07 | KF368691 | 58.7350 | -94.1090 |
| *Pityohyphantes limitaneus* | I | 07PROBE-04742 | ERSCH107-07 | KF368719 | 58.7350 | -94.1090 |
| *Pityohyphantes limitaneus* | I | 07PROBE-04743 | ERSCH108-07 | KF368715 | 58.7350 | -94.1090 |
| *Pityohyphantes limitaneus* | I | 07PROBE-04744 | ERSCH109-07 | KF368704 | 58.7350 | -94.1090 |
| *Pityohyphantes limitaneus* | I | 07PROBE-04745 | ERSCH110-07 | KF368711 | 58.7350 | -94.1090 |
| *Pityohyphantes limitaneus* | I | 07PROBE-04758 | ERSCH123-07 | KF368713 | 58.7350 | -94.1090 |
| *Pityohyphantes limitaneus* | I | 07PROBE-04761 | ERSCH126-07 | KF368702 | 58.6300 | -93.7980 |
| *Pityohyphantes limitaneus* | I | 07PROBE-04763 | ERSCH128-07 | KF368717 | 58.6300 | -93.7980 |
| *Pityohyphantes limitaneus* | I | 07PROBE-04764 | ERSCH129-07 | KF368700 | 58.6300 | -93.7980 |
| *Pityohyphantes limitaneus* | I | 07PROBE-04765 | ERSCH130-07 | KF368706 | 58.6300 | -93.7980 |
| *Pityohyphantes limitaneus* | I | 07PROBE-04768 | ERSCH133-07 | KF368718 | 58.6300 | -93.7980 |
| *Pityohyphantes limitaneus* | I | 07PROBE-04770 | ERSCH135-07 | KF368705 | 58.6300 | -93.7980 |
| *Pityohyphantes limitaneus* | I | 07PROBE-04772 | ERSCH137-07 | KF368701 | 58.6300 | -93.7980 |
| *Pityohyphantes limitaneus* | I | 07PROBE-04773 | ERSCH138-07 | KF368697 | 58.6300 | -93.7980 |
| *Pityohyphantes limitaneus* | I | 07PROBE-04774 | ERSCH139-07 | KF368709 | 58.6300 | -93.7980 |
| *Pityohyphantes limitaneus* | I | 07PROBE-04776 | ERSCH141-07 | KF368708 | 58.6300 | -93.7980 |
| *Pityohyphantes limitaneus* | I | 07PROBE-04777 | ERSCH142-07 | KF368696 | 58.6300 | -93.7980 |
| *Pityohyphantes limitaneus* | I | 07PROBE-04778 | ERSCH143-07 | KF368712 | 58.6300 | -93.7980 |
| *Pityohyphantes limitaneus* | I | 07PROBE-04779 | ERSCH144-07 | KF368695 | 58.6300 | -93.7980 |
| *Pityohyphantes limitaneus* | I | 07PROBE-04780 | ERSCH145-07 | KF368692 | 58.6300 | -93.7980 |
| *Pityohyphantes limitaneus* | I | 07PROBE-04781 | ERSCH146-07 | KF368707 | 58.6300 | -93.7980 |
| *Pityohyphantes limitaneus* | I | 07PROBE-04782 | ERSCH147-07 | KF368699 | 58.6300 | -93.7980 |
| *Pityohyphantes limitaneus* | A | 07PROBE-04783 | ERSCH148-07 | KF368693 | 58.6300 | -93.7980 |
| *Pityohyphantes limitaneus* | I | 09PROBE-01618 | SWSWE048-09 | GU681047 | 58.6300 | -93.8190 |
| *Pityohyphantes limitaneus* | I | 09PROBE-01619 | SWSWE049-09 | GU681044 | 58.6300 | -93.8190 |
| *Pityohyphantes limitaneus* | I | 09-PROBE-08131 | JDTGS035-09 | GU679886 | 58.7890 | -93.7100 |
| *Pityohyphantes limitaneus* | I | 09-PROBE-08151 | JDTGS055-09 | GU679871 | 58.6180 | -93.8290 |
| *Pityohyphantes limitaneus* | I | 09-PROBE-08164 | JDTGS068-09 | GU679861 | 58.6180 | -93.8290 |
| *Pityohyphantes limitaneus* | I | 09-PROBE-08180 | JDTGS084-09 | GU679832 | 58.7310 | -93.7800 |
| *Pityohyphantes limitaneus* | I | 09-PROBE-08187 | JDTGS091-09 | GU679844 | 58.6180 | -93.8290 |
| *Pityohyphantes limitaneus* | I | 09PROBE-1978-378 | SPICH621-09 | GU684126 | 58.7310 | -93.7800 |
| *Pityohyphantes limitaneus* | A | 09PROBE-649 | SPICH892-09 | GU684414 | 58.7240 | -93.8400 |
| *Pityohyphantes limitaneus* | A | 09PROBE-650 | SPICH893-09 | GU684456 | 58.7240 | -93.8400 |
| *Pityohyphantes limitaneus* | A | 09PROBE-651 | SPICH894-09 | GU684455 | 58.7240 | -93.8400 |
| *Pityohyphantes limitaneus* | I | 09PROBE-652 | SPICH895-09 | GU684454 | 58.7240 | -93.8400 |
| *Pityohyphantes limitaneus* | A | 09PROBE-914 | SPICH1135-09 | GU684662 | 58.6300 | -93.7980 |
| *Pityohyphantes limitaneus* | A | 09PROBE-923 | SPICH1144-09 | GU684663 | 58.6300 | -93.7980 |
| *Pityohyphantes limitaneus* | A | 09PROBE-924 | SPICH1145-09 | GU684660 | 58.6300 | -93.7980 |
| *Pityohyphantes limitaneus* | A | 09PROBE-927 | SPICH1148-09 | GU684661 | 58.6300 | -93.7980 |
| *Pityohyphantes limitaneus* | I | 10PROBE-21143 | GBADC023-10 | HQ956686 | 58.7340 | -94.1120 |
| *Pityohyphantes limitaneus* | A | BIOUG00629-A07 | SPIRU1169-11 | KF368694 | 58.7306 | -93.7804 |
| *Pityohyphantes limitaneus* | I | CHU-SPI-119 | KKCHE322-07 | KF368703 | 58.6170 | -93.8210 |
| *Pityohyphantes subarcticus* | I | 07PROBE-04552 | ERSCH007-07 | KF368720 | 58.6170 | -93.8140 |
| *Pityohyphantes subarcticus* | I | 07PROBE-04554 | ERSCH009-07 | KF368740 | 58.7310 | -93.7800 |
| *Pityohyphantes subarcticus* | I | 07PROBE-04560 | ERSCH015-07 | KF368726 | 58.7310 | -93.7800 |
| *Pityohyphantes subarcticus* | I | 07PROBE-04561 | ERSCH016-07 | KF368721 | 58.7310 | -93.7800 |
| *Pityohyphantes subarcticus* | I | 07PROBE-04562 | ERSCH017-07 | KF368742 | 58.7310 | -93.7800 |
| *Pityohyphantes subarcticus* | I | 07PROBE-04563 | ERSCH018-07 | KF368732 | 58.7310 | -93.7800 |
| *Pityohyphantes subarcticus* | I | 07PROBE-04570 | ERSCH025-07 | KF368752 | 58.7310 | -93.7800 |
| *Pityohyphantes subarcticus* | I | 07PROBE-04571 | ERSCH026-07 | KF368730 | 58.7310 | -93.7800 |
| *Pityohyphantes subarcticus* | I | 07PROBE-04576 | ERSCH031-07 | KF368744 | 58.7310 | -93.7800 |
| *Pityohyphantes subarcticus* | I | 07PROBE-04587 | ERSCH042-07 | KF368731 | 58.7310 | -93.7800 |
| *Pityohyphantes subarcticus* | I | 07PROBE-04594 | ERSCH049-07 | KF368729 | 58.7310 | -93.7800 |
| *Pityohyphantes subarcticus* | A | 07PROBE-04727 | ERSCH092-07 | KF368734 | 58.7350 | -94.1090 |
| *Pityohyphantes subarcticus* | A | 07PROBE-04740 | ERSCH105-07 | KF368738 | 58.7350 | -94.1090 |
| *Pityohyphantes subarcticus* | I | 09PROBE-01588 | SWSWE018-09 | GU681071 | 58.6190 | -93.8290 |
| *Pityohyphantes subarcticus* | I | 09PROBE-01617 | SWSWE047-09 | GU681046 | 58.6320 | -93.7860 |
| *Pityohyphantes subarcticus* | I | 09PROBE-01627 | SWSWE057-09 | GU681038 | 58.6320 | -93.7860 |
| *Pityohyphantes subarcticus* | I | 09PROBE-01643 | SWSWE073-09 | GU681024 | 58.6320 | -93.7860 |
| *Pityohyphantes subarcticus* | I | 09-PROBE-08140 | JDTGS044-09 | GU679878 | 58.6250 | -93.8190 |
| *Pityohyphantes subarcticus* | I | 09-PROBE-08152 | JDTGS056-09 | GU679868 | 58.6180 | -93.8290 |
| *Pityohyphantes subarcticus* | I | 09-PROBE-08166 | JDTGS070-09 | GU679859 | 58.6300 | -93.8190 |
| *Pityohyphantes subarcticus* | I | 09-PROBE-08168 | JDTGS072-09 | GU679856 | 58.6250 | -93.8190 |
| *Pityohyphantes subarcticus* | I | 09-PROBE-08171 | JDTGS075-09 | GU679854 | 58.6180 | -93.8290 |
| *Pityohyphantes subarcticus* | I | 09-PROBE-08174 | JDTGS078-09 | GU679853 | 58.6300 | -93.8190 |
| *Pityohyphantes subarcticus* | I | 09-PROBE-08175 | JDTGS079-09 | GU679850 | 58.6180 | -93.8290 |
| *Pityohyphantes subarcticus* | A | 09PROBE-1687-02 | SPICH124-09 | GU683769 | 58.7310 | -93.7800 |
| *Pityohyphantes subarcticus* | A | 09PROBE-1687-03 | SPICH125-09 | GU683768 | 58.7310 | -93.7800 |
| *Pityohyphantes subarcticus* | A | 09PROBE-1687-04 | SPICH126-09 | GU683767 | 58.7310 | -93.7800 |
| *Pityohyphantes subarcticus* | A | 09PROBE-1978-357 | SPICH600-09 | GU684124 | 58.6180 | -93.8290 |
| *Pityohyphantes subarcticus* | A | 09PROBE-1978-375 | SPICH618-09 | GU684125 | 58.7310 | -93.7800 |
| *Pityohyphantes subarcticus* | A | 09PROBE-1978-376 | SPICH619-09 | GU684127 | 58.7310 | -93.7800 |
| *Pityohyphantes subarcticus* | I | 09PROBE-1978-377 | SPICH620-09 | GU684122 | 58.7310 | -93.7800 |
| *Pityohyphantes subarcticus* | A | 09PROBE-906 | SPICH1127-09 | GU684666 | 58.6300 | -93.7980 |
| *Pityohyphantes subarcticus* | A | 09PROBE-907 | SPICH1128-09 | GU684665 | 58.6300 | -93.7980 |
| *Pityohyphantes subarcticus* | I | 09PROBE-908 | SPICH1129-09 | GU684667 | 58.6300 | -93.7980 |
| *Pityohyphantes subarcticus* | A | 09PROBE-911 | SPICH1132-09 | GU684668 | 58.6300 | -93.7980 |
| *Pityohyphantes subarcticus* | I | 10PROBE-21126 | GBADC006-10 | HQ956670 | 58.7340 | -94.1120 |
| *Pityohyphantes subarcticus* | I | 10PROBE-21132 | GBADC012-10 | HQ956676 | 58.7340 | -94.1120 |
| *Pityohyphantes subarcticus* | I | 10PROBE-21133 | GBADC013-10 | HQ956677 | 58.7340 | -94.1120 |
| *Pityohyphantes subarcticus* | I | 10PROBE-21138 | GBADC018-10 | HQ956681 | 58.7340 | -94.1120 |
| *Pityohyphantes subarcticus* | I | BIOUG00628-D04 | SPIRU1107-11 | KF368750 | 58.7304 | -93.7805 |
| *Pityohyphantes subarcticus* | I | CHU-SPI-002 | KKCHE111-06 | KF368733 | 58.6220 | -93.8100 |
| *Pityohyphantes subarcticus* | I | CHU-SPI-111 | KKCHE314-07 | KF368735 | 58.6180 | -93.8230 |
| *Pityohyphantes subarcticus* | I | CHU-SPI-112 | KKCHE315-07 | KF368746 | 58.6180 | -93.8230 |
| *Pityohyphantes subarcticus* | I | CHU-SPI-123 | KKCHE326-07 | KF368722 | 58.6180 | -93.8230 |
| *Pityohyphantes subarcticus* | A | CHU-SPI-130 | KKCHE333-07 | KF368725 | 58.6180 | -93.8230 |
| *Pityohyphantes subarcticus* | A | CHU-SPI-135 | KKCHE338-07 | KF368745 | 58.6180 | -93.8230 |
| *Pityohyphantes subarcticus* | I | CHU-SPI-147 | KKCHE350-07 | KF368749 | 58.6180 | -93.8230 |
| *Pityohyphantes subarcticus* | I | CHU-SPI-155 | KKCHE358-07 | KF368727 | 58.6170 | -93.8210 |
| *Pityohyphantes subarcticus* | I | CHU-SPI-171 | KKCHE374-07 | KF368728 | 58.6180 | -93.8230 |
| *Pityohyphantes subarcticus* | I | CHU-SPI-183 | KKCHE386-07 | KF368753 | 58.6180 | -93.8230 |
| *Pityohyphantes subarcticus* | I | CHU-SPI-220 | KKCHE423-07 | KF368724 | 58.6910 | -94.1790 |
| *Pityohyphantes subarcticus* | I | CHU-SPI-222 | KKCHE425-07 | KF368723 | 58.6910 | -94.1790 |
| *Pityohyphantes subarcticus* | I | CHU-SPI-232 | KKCHE435-07 | KF368747 | 58.6910 | -94.1790 |
| *Pityohyphantes subarcticus* | I | CHU-SPI-268 | KKCHE471-07 | KF368743 | 58.6910 | -94.1790 |
| *Pityohyphantes subarcticus* | I | CHU-SPI-280 | KKCHE483-07 | KF368739 | 58.6910 | -94.1790 |
| *Pityohyphantes subarcticus* | I | CHU-SPI-282 | KKCHE485-07 | KF368748 | 58.6910 | -94.1790 |
| *Pityohyphantes subarcticus* | I | CHU-SPI-382 | KKCHE580-07 | KF368737 | 58.6320 | -93.7860 |
| *Pityohyphantes subarcticus* | I | CHU-SPI-404 | KKCHE607-07 | KF368736 | 58.7540 | -93.9130 |
| *Pityohyphantes subarcticus* | I | CHU-SPI-440 | KKCHE643-07 | KF368751 | 58.7540 | -93.9130 |
| *Pityohyphantes subarcticus* | I | CHU-SPI-570 | KKCHE773-07 | KF368741 | 58.7380 | -93.8190 |
| *Pocadicnemis americana* | A | 07CHU-AR-0011 | KKCHE923-09 | GU683632 | 58.6300 | -93.7980 |
| *Pocadicnemis americana* | I | 09PROBE-1741-01 | SPICH180-09 | GU684007 | 58.6180 | -93.8290 |
| *Pocadicnemis americana* | I | 09PROBE-1741-02 | SPICH181-09 | GU684009 | 58.6180 | -93.8290 |
| *Pocadicnemis americana* | I | 09PROBE-1963-01 | SPICH096-09 | GU684023 | 58.6300 | -93.8190 |
| *Pocadicnemis americana* | A | 09PROBE-1978-159 | SPICH402-09 | GU684203 | 58.6180 | -93.8290 |
| *Pocadicnemis americana* | A | 09PROBE-1978-163 | SPICH406-09 | GU684204 | 58.6180 | -93.8290 |
| *Pocadicnemis americana* | I | 09PROBE-1978-212 | SPICH455-09 | GU684080 | 58.6300 | -93.7980 |
| *Pocadicnemis americana* | A | BIOUG00632-A09 | SPIRU1361-11 | KF368754 | 58.6173 | -93.8123 |
| *Poeciloneta calcaratus* | A | 09PROBE-1978-356 | SPICH599-09 | GU684169 | 58.6180 | -93.8290 |
| *Poeciloneta variegata* | I | 09PROBE-630 | SPICH873-09 | HM416916 | 58.7900 | -94.2270 |
| *Praestigia kulczynskii* | A | 09PROBE-1829-01 | SPICH241-09 | HM432628 | 58.6300 | -93.8190 |
| *Praestigia kulczynskii* | I | BIOUG00627-H06 | SPIRU1062-11 | KF368755 | 58.6329 | -93.7869 |
| *Robertus borealis* | A | BIOUG00627-F07 | SPIRU1039-11 | KF368756 | 58.6329 | -93.7869 |
| *Robertus fuscus* | A | 07CHU-AR-0004 | KKCHE916-09 | GU683626 | 58.6190 | -93.8290 |
| *Robertus fuscus* | I | 09PROBE-1978-383 | SPICH626-09 | GU684519 | 58.6250 | -93.8160 |
| *Robertus fuscus* | A | 09PROBE-1978-384 | SPICH627-09 | GU684522 | 58.6250 | -93.8160 |
| *Robertus fuscus* | A | 09PROBE-1978-385 | SPICH628-09 | GU684523 | 58.6250 | -93.8160 |
| *Robertus fuscus* | A | 09PROBE-1978-386 | SPICH629-09 | GU684524 | 58.6250 | -93.8160 |
| *Robertus fuscus* | A | 09PROBE-1978-387 | SPICH630-09 | GU684520 | 58.6250 | -93.8160 |
| *Robertus fuscus* | A | 09PROBE-1978-388 | SPICH631-09 | GU684525 | 58.6250 | -93.8160 |
| *Robertus fuscus* | A | 09PROBE-1978-389 | SPICH632-09 | GU684526 | 58.6250 | -93.8160 |
| *Robertus fuscus* | A | 09PROBE-1978-390 | SPICH633-09 | GU684518 | 58.6250 | -93.8160 |
| *Robertus fuscus* | A | 09PROBE-1978-391 | SPICH634-09 | GU684527 | 58.6250 | -93.8160 |
| *Robertus fuscus* | A | 09PROBE-1978-392 | SPICH635-09 | GU684521 | 58.6250 | -93.8160 |
| *Robertus fuscus* | A | 09PROBE-1978-396 | SPICH639-09 | GU684528 | 58.6250 | -93.8160 |
| *Robertus fuscus* | A | 09PROBE-696 | SPICH939-09 | GU684501 | 58.6180 | -93.8300 |
| *Robertus lyrifer* | I | 07CHU-AR-0280 | KKCHE1087-09 | HM377208 | 58.7690 | -94.1600 |
| *Robertus lyrifer* | A | BIOUG00606-E11 | SPIRU691-11 | JF886909 | 59.7352 | 150.8670 |
| *Robertus lyrifer* | A | BIOUG00606-E12 | SPIRU692-11 | JF886910 | 59.7352 | 150.8670 |
| *Robertus lyrifer* | A | BIOUG00630-B08 | SPIRU1277-11 | KF368757 | 58.7690 | -93.8620 |
| *Satilatlas marxi* | I | 09PROBE-1978-343 | SPICH586-09 | GU684167 | 58.6260 | -94.2300 |
| *Satilatlas marxi* | A | 09PROBE-1978-365 | SPICH608-09 | GU684168 | 58.7640 | -93.8970 |
| *Satilatlas marxi* | A | 09PROBE-1978-407 | SPICH650-09 | GU684543 | 58.7640 | -93.8970 |
| *Satilatlas marxi* | A | 09PROBE-1978-440 | SPICH683-09 | GU684544 | 58.7640 | -93.8970 |
| *Satilatlas marxi* | A | 09PROBE-1978-443 | SPICH686-09 | GU684545 | 58.7640 | -93.8970 |
| *Satilatlas marxi* | I | 09PROBE-1978-461 | SPICH704-09 | GU684546 | 58.7640 | -93.8970 |
| *Satilatlas marxi* | A | 09PROBE-737 | SPICH980-09 | GU684466 | 58.6260 | -94.2300 |
| *Satilatlas marxi* | A | BIOUG00627-D10 | SPIRU1018-11 | KF368758 | 58.7544 | -93.9980 |
| *Satilatlas marxi* | A | BIOUG00628-F09 | SPIRU1136-11 | KF368759 | 58.7544 | -93.9980 |
| *Satilatlas monticola* | I | 09PROBE-1740-01 | SPICH179-09 | GU684014 | 58.6180 | -93.8290 |
| *Satilatlas monticola* | A | 09PROBE-1848-01 | SPICH017-09 | GU683836 | 58.6300 | -93.8190 |
| *Satilatlas monticola* | A | 09PROBE-1944-01 | SPICH083-09 | GU683842 | 58.7640 | -93.8970 |
| *Satilatlas monticola* | A | 09PROBE-1948-01 | SPICH087-09 | GU683839 | 58.7640 | -93.8970 |
| *Satilatlas monticola* | A | 09PROBE-1950-01 | SPICH089-09 | GU683840 | 58.7640 | -93.8970 |
| *Satilatlas monticola* | A | 09PROBE-1951-01 | SPICH090-09 | GU683838 | 58.7640 | -93.8970 |
| *Satilatlas monticola* | A | 09PROBE-1952-01 | SPICH091-09 | GU683837 | 58.7640 | -93.8970 |
| *Satilatlas monticola* | A | 09PROBE-1953-01 | SPICH092-09 | GU683841 | 58.7640 | -93.8970 |
| *Satilatlas monticola* | A | 09PROBE-1954-01 | SPICH093-09 | GU683843 | 58.7640 | -93.8970 |
| *Satilatlas monticola* | A | 09PROBE-1955-01 | SPICH094-09 | GU683844 | 58.7640 | -93.8970 |
| *Satilatlas monticola* | A | 09PROBE-1956-01 | SPICH095-09 | GU683845 | 58.7640 | -93.8970 |
| *Satilatlas monticola* | I | 09PROBE-1978-145 | SPICH388-09 | GU684240 | 58.6300 | -93.7980 |
| *Satilatlas monticola* | I | 09PROBE-1978-149 | SPICH392-09 | GU684237 | 58.6300 | -93.7980 |
| *Satilatlas monticola* | A | 09PROBE-1978-192 | SPICH435-09 | GU684049 | 58.6300 | -93.7980 |
| *Satilatlas monticola* | A | 09PROBE-1978-199 | SPICH442-09 | GU684051 | 58.6300 | -93.7980 |
| *Satilatlas monticola* | A | 09PROBE-1978-201 | SPICH444-09 | GU684048 | 58.6300 | -93.7980 |
| *Satilatlas monticola* | A | 09PROBE-1978-218 | SPICH461-09 | GU684047 | 58.6300 | -93.7980 |
| *Satilatlas monticola* | A | 09PROBE-1978-366 | SPICH609-09 | KF368762 | 58.7640 | -93.8970 |
| *Satilatlas monticola* | A | 09PROBE-1978-368 | SPICH611-09 | GU684166 | 58.7640 | -93.8970 |
| *Satilatlas monticola* | A | 09PROBE-1978-408 | SPICH651-09 | GU684551 | 58.7640 | -93.8970 |
| *Satilatlas monticola* | I | 09PROBE-1978-421 | SPICH664-09 | GU684547 | 58.7640 | -93.8970 |
| *Satilatlas monticola* | A | 09PROBE-1978-422 | SPICH665-09 | KF368763 | 58.7640 | -93.8970 |
| *Satilatlas monticola* | I | 09PROBE-1978-429 | SPICH672-09 | GU684548 | 58.7640 | -93.8970 |
| *Satilatlas monticola* | A | 09PROBE-1978-431 | SPICH674-09 | GU684550 | 58.7640 | -93.8970 |
| *Satilatlas monticola* | A | 09PROBE-1978-439 | SPICH682-09 | GU684512 | 58.7640 | -93.8970 |
| *Satilatlas monticola* | A | 09PROBE-1978-441 | SPICH684-09 | GU684549 | 58.7640 | -93.8970 |
| *Satilatlas monticola* | I | BIOUG00628-C06 | SPIRU1097-11 | KF368764 | 58.7640 | -93.8970 |
| *Satilatlas monticola* | I | BIOUG00628-G11 | SPIRU1150-11 | KF368770 | 58.7718 | -93.8439 |
| *Satilatlas monticola* | A | BIOUG00629-E11 | SPIRU1221-11 | KF368761 | 58.7710 | -93.8510 |
| *Satilatlas monticola* | A | BIOUG00629-E12 | SPIRU1222-11 | KF368769 | 58.7710 | -93.8510 |
| *Satilatlas monticola* | A | BIOUG00630-C03 | SPIRU1284-11 | KF368771 | 58.7710 | -93.8510 |
| *Satilatlas monticola* | A | BIOUG00630-C04 | SPIRU1285-11 | KF368768 | 58.7710 | -93.8510 |
| *Satilatlas monticola* | A | BIOUG00630-C06 | SPIRU1287-11 | KF368765 | 58.7710 | -93.8510 |
| *Satilatlas monticola* | A | BIOUG00630-C09 | SPIRU1290-11 | KF368760 | 58.7666 | -93.8530 |
| *Satilatlas monticola* | A | BIOUG00630-F02 | SPIRU1319-11 | KF368767 | 58.7305 | -93.7805 |
| *Satilatlas monticola* | A | BIOUG00632-C03 | SPIRU1379-11 | KF368766 | 58.7710 | -93.8510 |
| *Sciastes dubius* | I | CHU-SPI-359 | KKCHE534-07 | KF368772 | 58.6320 | -93.7860 |
| *Sciastes dubius* | I | CHU-SPI-367 | KKCHE548-07 | KF368773 | 58.6320 | -93.7860 |
| *Sciastes hastatus* | I | 09PROBE-1978-281 | SPICH524-09 | HM432631 | 58.6690 | -94.1600 |
| *Sciastes truncatus* | A | 09PROBE-1978-196 | SPICH439-09 | GU684093 | 58.6300 | -93.7980 |
| *Sciastes truncatus* | A | 09PROBE-1978-203 | SPICH446-09 | GU684091 | 58.6300 | -93.7980 |
| *Sciastes truncatus* | A | 09PROBE-1978-217 | SPICH460-09 | GU684092 | 58.6300 | -93.7980 |
| *Sciastes truncatus* | A | BIOUG00630-G12 | SPIRU1341-11 | KF368774 | 58.6173 | -93.8123 |
| *Sciastes truncatus* | I | BIOUG00632-A02 | SPIRU1354-11 | KF368775 | 58.6300 | -93.8190 |
| *Sciastes truncatus* | A | BIOUG00632-A03 | SPIRU1355-11 | KF368776 | 58.6300 | -93.8190 |
| *Scotinotylus alpinus* | A | 09PROBE-1978-498 | SPICH741-09 | GU684371 | 58.7890 | -93.7090 |
| *Scotinotylus alpinus* | A | 09PROBE-585 | SPICH828-09 | GU684442 | 58.7960 | -93.7540 |
| *Scotinotylus alpinus* | A | 09PROBE-586 | SPICH829-09 | GU684405 | 58.7960 | -93.7540 |
| *Scotinotylus alpinus* | A | 09PROBE-587 | SPICH830-09 | GU684441 | 58.7960 | -93.7540 |
| *Scotinotylus alpinus* | A | 09PROBE-588 | SPICH831-09 | GU684443 | 58.7960 | -93.7540 |
| *Scotinotylus sacer* | A | 09PROBE-1813-01 | SPICH225-09 | GU683970 | 58.6300 | -93.8190 |
| *Scotinotylus sacer* | A | 09PROBE-1864-01 | SPICH033-09 | GU683849 | 58.6300 | -93.8190 |
| *Scotinotylus sacer* | I | 09PROBE-1978-148 | SPICH391-09 | GU684226 | 58.6300 | -93.7980 |
| *Scotinotylus sacer* | A | 09PROBE-1978-206 | SPICH449-09 | GU684094 | 58.6300 | -93.7980 |
| *Scotinotylus* sp. 1GAB | I | 09PROBE-1965-01 | SPICH098-09 | HM432625 | 58.6300 | -93.8190 |
| *Scylaceus pallidus* | A | 09PROBE-1978-36 | SPICH279-09 | GU684297 | 58.6690 | -94.1600 |
| *Scylaceus pallidus* | A | 09PROBE-868 | SPICH1089-09 | GU684629 | 58.6630 | -94.1670 |
| *Scylaceus pallidus* | I | BIOUG00627-C07 | SPIRU1003-11 | KF368778 | 58.7544 | -93.9980 |
| *Scylaceus pallidus* | A | BIOUG00627-C09 | SPIRU1005-11 | KF368780 | 58.7544 | -93.9980 |
| *Scylaceus pallidus* | I | BIOUG00627-D11 | SPIRU1019-11 | KF368777 | 58.7544 | -93.9980 |
| *Scylaceus pallidus* | A | BIOUG00627-E02 | SPIRU1022-11 | KF368779 | 58.7544 | -93.9980 |
| *Scyletria inflata* | I | 09PROBE-1978-308 | SPICH551-09 | GU684147 | 58.6690 | -94.1600 |
| *Scyletria inflata* | A | 09PROBE-1978-339 | SPICH582-09 | GU684148 | 58.6260 | -94.2300 |
| *Semljicola lapponicus* | I | CHU-SPI-578 | KKCHE781-07 | KF368781 | 58.7470 | -94.1340 |
| *Semljicola obtusus* | I | 07CHU-AR-0038 | KKCHE950-09 | HM377209 | 58.6620 | -93.1890 |
| *Sisicottus montanus* | I | 07CHU-AR-0037 | KKCHE949-09 | GU683652 | 58.6620 | -93.1890 |
| *Sisicottus montanus* | I | 09-PROBE-08109 | JDTGS013-09 | GU679840 | 58.6300 | -93.8190 |
| *Sisicottus montanus* | I | 09PROBE-1709-01 | SPICH159-09 | GU684020 | 58.6300 | -93.8190 |
| *Sisicottus montanus* | I | 09PROBE-1766-01 | SPICH190-09 | GU683825 | 58.6300 | -93.8190 |
| *Sisicottus montanus* | A | 09PROBE-1809-01 | SPICH221-09 | GU683915 | 58.6300 | -93.8190 |
| *Sisicottus montanus* | A | 09PROBE-1812-01 | SPICH224-09 | GU683988 | 58.6300 | -93.8190 |
| *Sisicottus montanus* | A | 09PROBE-1814-01 | SPICH226-09 | GU683989 | 58.6300 | -93.8190 |
| *Sisicottus montanus* | A | 09PROBE-1818-01 | SPICH230-09 | GU683990 | 58.6300 | -93.8190 |
| *Sisicottus montanus* | A | 09PROBE-1819-01 | SPICH231-09 | GU683992 | 58.6300 | -93.8190 |
| *Sisicottus montanus* | A | 09PROBE-1822-01 | SPICH234-09 | GU683991 | 58.6300 | -93.8190 |
| *Sisicottus montanus* | A | 09PROBE-1839-01 | SPICH008-09 | GU683894 | 58.6300 | -93.8190 |
| *Sisicottus montanus* | A | 09PROBE-1840-01 | SPICH009-09 | GU683891 | 58.6300 | -93.8190 |
| *Sisicottus montanus* | A | 09PROBE-1844-01 | SPICH013-09 | GU683890 | 58.6300 | -93.8190 |
| *Sisicottus montanus* | A | 09PROBE-1850-01 | SPICH019-09 | GU683892 | 58.6300 | -93.8190 |
| *Sisicottus montanus* | A | 09PROBE-1866-01 | SPICH035-09 | GU683893 | 58.6300 | -93.8190 |
| *Sisicottus montanus* | I | 09PROBE-1978-146 | SPICH389-09 | GU684185 | 58.6300 | -93.7980 |
| *Sisicottus montanus* | I | 09PROBE-1978-150 | SPICH393-09 | GU684186 | 58.7310 | -93.7800 |
| *Sisicottus montanus* | A | 09PROBE-1978-193 | SPICH436-09 | GU684067 | 58.6300 | -93.7980 |
| *Sisicottus montanus* | A | 09PROBE-1978-198 | SPICH441-09 | GU684059 | 58.6300 | -93.7980 |
| *Sisicottus montanus* | A | 09PROBE-1978-200 | SPICH443-09 | GU684057 | 58.6300 | -93.7980 |
| *Sisicottus montanus* | A | 09PROBE-1978-204 | SPICH447-09 | GU684060 | 58.6300 | -93.7980 |
| *Sisicottus montanus* | A | 09PROBE-1978-208 | SPICH451-09 | GU684069 | 58.6300 | -93.7980 |
| *Sisicottus montanus* | A | 09PROBE-1978-210 | SPICH453-09 | GU684056 | 58.6300 | -93.7980 |
| *Sisicottus montanus* | A | 09PROBE-1978-216 | SPICH459-09 | GU684066 | 58.6300 | -93.7980 |
| *Sisicottus montanus* | A | 09PROBE-1978-219 | SPICH462-09 | GU684065 | 58.6300 | -93.7980 |
| *Sisicottus montanus* | A | 09PROBE-1978-222 | SPICH465-09 | GU684063 | 58.6300 | -93.7980 |
| *Sisicottus montanus* | A | 09PROBE-1978-223 | SPICH466-09 | GU684062 | 58.6300 | -93.7980 |
| *Sisicottus montanus* | A | 09PROBE-1978-224 | SPICH467-09 | GU684061 | 58.6300 | -93.7980 |
| *Sisicottus montanus* | A | 09PROBE-1978-228 | SPICH471-09 | GU684058 | 58.6300 | -93.7980 |
| *Sisicottus montanus* | A | 09PROBE-1978-229 | SPICH472-09 | GU684068 | 58.6300 | -93.7980 |
| *Sisicottus montanus* | A | 09PROBE-1978-232 | SPICH475-09 | GU684071 | 58.6300 | -93.7980 |
| *Sisicottus montanus* | A | 09PROBE-1978-233 | SPICH476-09 | GU684064 | 58.6300 | -93.7980 |
| *Sisicottus montanus* | A | 09PROBE-1978-395 | SPICH638-09 | GU684535 | 58.6250 | -93.8160 |
| *Sisicottus montanus* | A | 09PROBE-645 | SPICH888-09 | GU684452 | 58.6300 | -93.7980 |
| *Sisicottus montanus* | A | 09PROBE-646 | SPICH889-09 | GU684413 | 58.6300 | -93.7980 |
| *Sisicottus montanus* | A | 09PROBE-751 | SPICH994-09 | GU684474 | 58.6260 | -94.2300 |
| *Sisis rotundus* | A | BIOUG00630-H07 | SPIRU1348-11 | KF368782 | 58.6300 | -93.8190 |
| *Sitticus ammophilus* | A | BIOUG00628-A11 | SPIRU1078-11 | KF368783 | 58.7544 | -93.9980 |
| *Sitticus finschi* | A | 09PROBE-1659-01 | SPISH015-09 | HM432635 | 58.6180 | -93.8290 |
| *Sitticus floricola palustris* | A | 09PROBE-581 | SPICH824-09 | GU684425 | 58.7600 | -94.0860 |
| *Sitticus floricola palustris* | I | BIOUG00627-D03 | SPIRU1011-11 | KF368789 | 58.7544 | -93.9980 |
| *Sitticus floricola palustris* | I | BIOUG00627-D04 | SPIRU1012-11 | KF368788 | 58.7544 | -93.9980 |
| *Sitticus floricola palustris* | A | BIOUG00627-E08 | SPIRU1028-11 | KF368790 | 58.7055 | -94.0536 |
| *Sitticus floricola palustris* | I | BIOUG00627-F12 | SPIRU1044-11 | KF368786 | 58.7717 | -94.1828 |
| *Sitticus floricola palustris* | A | BIOUG00628-B11 | SPIRU1090-11 | KF368787 | 58.7340 | -94.1120 |
| *Sitticus floricola palustris* | A | BIOUG00629-C09 | SPIRU1195-11 | KF368784 | 58.7179 | -94.1221 |
| *Sitticus floricola palustris* | A | BIOUG00629-E01 | SPIRU1211-11 | KF368791 | 58.6264 | -94.2303 |
| *Sitticus floricola palustris* | A | BIOUG00630-F05 | SPIRU1322-11 | KF368785 | 58.7305 | -93.7805 |
| *Sitticus ranieri* | A | 07CHU-AR-0033 | KKCHE945-09 | GU683651 | 58.6780 | -94.1460 |
| *Sitticus ranieri* | I | 07CHU-AR-0046 | KKCHE958-09 | GU683659 | 58.6620 | -93.1890 |
| *Sitticus ranieri* | I | 07PROBE-02847 | TWSC233-08 | KF368801 | 58.7300 | -93.7840 |
| *Sitticus ranieri* | I | 07PROBE-04619 | ERSCH074-07 | KF368798 | 58.7330 | -93.8200 |
| *Sitticus ranieri* | I | 09-PROBE-08115 | JDTGS019-09 | GU679841 | 58.6180 | -93.8100 |
| *Sitticus ranieri* | I | 09-PROBE-08116 | JDTGS020-09 | GU679899 | 58.7300 | -93.7840 |
| *Sitticus ranieri* | A | 09PROBE-573 | SPICH816-09 | GU684426 | 58.6300 | -93.7980 |
| *Sitticus ranieri* | I | 09PROBE-574 | SPICH817-09 | GU684427 | 58.6300 | -93.7980 |
| *Sitticus ranieri* | A | 09PROBE-946 | SPICH1167-09 | GU684588 | 58.7310 | -93.7800 |
| *Sitticus ranieri* | A | 09PROBE-947 | SPICH1168-09 | GU684589 | 58.7310 | -93.7800 |
| *Sitticus ranieri* | A | BIOUG00628-D06 | SPIRU1109-11 | KF368803 | 58.7605 | -93.9523 |
| *Sitticus ranieri* | I | BIOUG00628-E09 | SPIRU1124-11 | KF368802 | 58.7574 | -93.9009 |
| *Sitticus ranieri* | A | BIOUG00628-F03 | SPIRU1130-11 | KF368795 | 58.7544 | -93.9980 |
| *Sitticus ranieri* | A | BIOUG00629-D08 | SPIRU1206-11 | KF368794 | 58.7306 | -93.7804 |
| *Sitticus ranieri* | A | BIOUG00629-E04 | SPIRU1214-11 | KF368799 | 58.6296 | -93.7975 |
| *Sitticus ranieri* | A | BIOUG00629-G03 | SPIRU1237-11 | KF368796 | 58.7720 | -93.8431 |
| *Sitticus ranieri* | A | BIOUG00630-E09 | SPIRU1314-11 | KF368800 | 58.7720 | -93.8431 |
| *Sitticus ranieri* | A | BIOUG00630-F03 | SPIRU1320-11 | KF368792 | 58.7305 | -93.7805 |
| *Sitticus ranieri* | I | BIOUG00630-F04 | SPIRU1321-11 | KF368793 | 58.7305 | -93.7805 |
| *Sitticus ranieri* | I | JBWM0234213 | KKCHE278-07 | KF368797 | 58.7460 | -94.1130 |
| *Sitticus striatus* | A | BIOUG00628-H08 | SPIRU1159-11 | KF368804 | 58.6329 | -93.7869 |
| *Sitticus striatus* | A | BIOUG00628-H09 | SPIRU1160-11 | KF368805 | 58.6329 | -93.7869 |
| *Souessa spinifera* | A | 09PROBE-1978-103 | SPICH346-09 | GU684215 | 58.6690 | -94.1600 |
| *Souessa spinifera* | A | 09PROBE-1978-111 | SPICH354-09 | GU684214 | 58.6690 | -94.1600 |
| *Souessa spinifera* | A | 09PROBE-1978-112 | SPICH355-09 | GU684218 | 58.6690 | -94.1600 |
| *Souessa spinifera* | A | 09PROBE-1978-114 | SPICH357-09 | GU684216 | 58.6690 | -94.1600 |
| *Souessa spinifera* | A | 09PROBE-1978-125 | SPICH368-09 | GU684213 | 58.6690 | -94.1600 |
| *Souessa spinifera* | A | 09PROBE-1978-23 | SPICH266-09 | GU684290 | 58.6690 | -94.1600 |
| *Souessa spinifera* | A | 09PROBE-1978-293 | SPICH536-09 | GU684164 | 58.6690 | -94.1600 |
| *Souessa spinifera* | I | 09PROBE-1978-329 | SPICH572-09 | GU684162 | 58.6690 | -94.1600 |
| *Souessa spinifera* | A | 09PROBE-1978-347 | SPICH590-09 | GU684165 | 58.7610 | -93.9540 |
| *Souessa spinifera* | A | 09PROBE-1978-80 | SPICH323-09 | GU684291 | 58.6690 | -94.1600 |
| *Souessa spinifera* | I | 09PROBE-870 | SPICH1091-09 | GU684611 | 58.6630 | -94.1670 |
| *Souessa spinifera* | A | BIOUG00627-C02 | SPIRU998-11 | KF368806 | 58.7920 | -93.7510 |
| *Styloctetor purpurescens* | A | BIOUG00627-C08 | SPIRU1004-11 | KF368808 | 58.7544 | -93.9980 |
| *Styloctetor purpurescens* | A | BIOUG00627-D12 | SPIRU1020-11 | KF368807 | 58.7544 | -93.9980 |
| *Tapinocyba bicarinata* | A | 09PROBE-1862-01 | SPICH031-09 | GU683883 | 58.6300 | -93.8190 |
| *Tapinocyba bicarinata* | A | 09PROBE-1906-01 | SPICH063-09 | GU683884 | 58.6180 | -93.8290 |
| *Tapinocyba bicarinata* | I | 09PROBE-1978-173 | SPICH416-09 | GU684197 | 58.6180 | -93.8290 |
| *Tapinocyba bicarinata* | A | 09PROBE-1978-211 | SPICH454-09 | GU684072 | 58.6300 | -93.7980 |
| *Tapinocyba minuta* | I | 09PROBE-1765-01 | SPICH189-09 | HM432626 | 58.6300 | -93.8190 |
| *Tapinocyba* sp. 1GAB | I | 09PROBE-1978-379 | SPICH622-09 | HQ975034 | 58.7310 | -93.7800 |
| *Tetragnatha extensa* | I | 07PROBE-04578 | ERSCH033-07 | KF368866 | 58.7310 | -93.7800 |
| *Tetragnatha extensa* | I | 07PROBE-04579 | ERSCH034-07 | KF368815 | 58.7310 | -93.7800 |
| *Tetragnatha extensa* | I | 07PROBE-04586 | ERSCH041-07 | KF368833 | 58.7310 | -93.7800 |
| *Tetragnatha extensa* | I | 07PROBE-04592 | ERSCH047-07 | KF368848 | 58.7310 | -93.7800 |
| *Tetragnatha extensa* | I | 07PROBE-04610 | ERSCH065-07 | KF368879 | 58.7330 | -93.8200 |
| *Tetragnatha extensa* | I | 07PROBE-04625 | ERSCH080-07 | KF368812 | 58.6300 | -93.7980 |
| *Tetragnatha extensa* | I | 07PROBE-04630 | ERSCH085-07 | KF368884 | 58.6300 | -93.7980 |
| *Tetragnatha extensa* | I | 07PROBE-04730 | ERSCH095-07 | KF368878 | 58.7350 | -94.1090 |
| *Tetragnatha extensa* | I | 07PROBE-04732 | ERSCH097-07 | KF368864 | 58.7350 | -94.1090 |
| *Tetragnatha extensa* | I | 07PROBE-04751 | ERSCH116-07 | KF368859 | 58.7350 | -94.1090 |
| *Tetragnatha extensa* | I | 07PROBE-04753 | ERSCH118-07 | KF368836 | 58.7350 | -94.1090 |
| *Tetragnatha extensa* | I | 07PROBE-04756 | ERSCH121-07 | KF368819 | 58.7350 | -94.1090 |
| *Tetragnatha extensa* | I | 07PROBE-04760 | ERSCH125-07 | KF368852 | 58.6300 | -93.7980 |
| *Tetragnatha extensa* | I | 07PROBE-04767 | ERSCH132-07 | KF368814 | 58.6300 | -93.7980 |
| *Tetragnatha extensa* | I | 07PROBE-04771 | ERSCH136-07 | KF368871 | 58.6300 | -93.7980 |
| *Tetragnatha extensa* | I | 09PROBE-01571 | SWSWE001-09 | KF368888 | 58.6900 | -93.8500 |
| *Tetragnatha extensa* | I | 09PROBE-01576 | SWSWE006-09 | GU681066 | 58.6900 | -93.8500 |
| *Tetragnatha extensa* | I | 09PROBE-01598 | SWSWE028-09 | KF368868 | 58.7560 | -93.9130 |
| *Tetragnatha extensa* | I | 09PROBE-01600 | SWSWE030-09 | KF368850 | 58.7540 | -93.9140 |
| *Tetragnatha extensa* | I | 09PROBE-01610 | SWSWE040-09 | KF368843 | 58.6620 | -94.1650 |
| *Tetragnatha extensa* | I | 09PROBE-01611 | SWSWE041-09 | KF368876 | 58.6620 | -94.1650 |
| *Tetragnatha extensa* | I | 09PROBE-1713-01 | SPICH167-09 | GU684025 | 58.6690 | -94.1600 |
| *Tetragnatha extensa* | I | 09PROBE-1713-02 | SPICH168-09 | GU684028 | 58.6690 | -94.1600 |
| *Tetragnatha extensa* | A | 09PROBE-1967-01 | SPICH099-09 | GU684026 | 58.6690 | -94.1600 |
| *Tetragnatha extensa* | A | 09PROBE-1978-399 | SPICH642-09 | KF368863 | 58.7380 | -93.8190 |
| *Tetragnatha extensa* | A | 09PROBE-1978-486 | SPICH729-09 | KF368867 | 58.6300 | -94.2300 |
| *Tetragnatha extensa* | I | 09PROBE-1978-548 | SPICH791-09 | KF368823 | 58.7870 | -93.7140 |
| *Tetragnatha extensa* | A | 09PROBE-597 | SPICH840-09 | KF368873 | 58.7900 | -94.2270 |
| *Tetragnatha extensa* | A | 09PROBE-598 | SPICH841-09 | KF368857 | 58.7900 | -94.2270 |
| *Tetragnatha extensa* | I | 09PROBE-796 | SPICH1017-09 | KF368856 | 58.7380 | -93.8190 |
| *Tetragnatha extensa* | I | 10PROBE-21121 | GBADC001-10 | HQ956665 | 58.7340 | -94.1120 |
| *Tetragnatha extensa* | I | 10PROBE-21124 | GBADC004-10 | HQ956668 | 58.7340 | -94.1120 |
| *Tetragnatha extensa* | I | 10PROBE-21125 | GBADC005-10 | HQ956669 | 58.7340 | -94.1120 |
| *Tetragnatha extensa* | I | 10PROBE-21139 | GBADC019-10 | HQ956682 | 58.7340 | -94.1120 |
| *Tetragnatha extensa* | I | 10PROBE-21144 | GBADC024-10 | HQ956687 | 58.7340 | -94.1120 |
| *Tetragnatha extensa* | I | 10PROBE-21147 | GBADC027-10 | HQ956690 | 58.7340 | -94.1120 |
| *Tetragnatha extensa* | I | 10PROBE-21149 | GBADC029-10 | HQ956692 | 58.7340 | -94.1120 |
| *Tetragnatha extensa* | I | 10PROBE-21150 | GBADC030-10 | HQ956693 | 58.7340 | -94.1120 |
| *Tetragnatha extensa* | I | 10PROBE-21153 | GBADC033-10 | HQ956696 | 58.7340 | -94.1120 |
| *Tetragnatha extensa* | I | 10PROBE-21156 | GBADC036-10 | HQ956699 | 58.7340 | -94.1120 |
| *Tetragnatha extensa* | I | 10PROBE-21158 | GBADC038-10 | HQ956700 | 58.7340 | -94.1120 |
| *Tetragnatha extensa* | I | 10PROBE-21159 | GBADC039-10 | HQ956701 | 58.7340 | -94.1120 |
| *Tetragnatha extensa* | I | 10PROBE-21161 | GBADC041-10 | HQ956703 | 58.7340 | -94.1120 |
| *Tetragnatha extensa* | I | 10PROBE-21162 | GBADC042-10 | HQ956704 | 58.7340 | -94.1120 |
| *Tetragnatha extensa* | I | 10PROBE-21174 | GBADC054-10 | HQ956715 | 58.6340 | -93.7860 |
| *Tetragnatha extensa* | I | 10PROBE-21179 | GBADC059-10 | HQ956720 | 58.6340 | -93.7860 |
| *Tetragnatha extensa* | I | 10PROBE-21185 | GBADC065-10 | HQ956725 | 58.6340 | -93.7860 |
| *Tetragnatha extensa* | I | 10PROBE-21189 | GBADC069-10 | HQ956729 | 58.6610 | -93.8320 |
| *Tetragnatha extensa* | I | 10PROBE-21191 | GBADC071-10 | HQ956731 | 58.6610 | -93.8320 |
| *Tetragnatha extensa* | I | 10PROBE-21192 | GBADC072-10 | HQ956732 | 58.6610 | -93.8320 |
| *Tetragnatha extensa* | I | 10PROBE-21199 | GBADC079-10 | HQ956739 | 58.6610 | -93.8320 |
| *Tetragnatha extensa* | I | 10PROBE-21203 | GBADC083-10 | HQ956742 | 58.7180 | -94.1240 |
| *Tetragnatha extensa* | I | 10PROBE-21211 | GBADC091-10 | HQ956749 | 58.7180 | -94.1240 |
| *Tetragnatha extensa* | I | 10PROBE-21213 | GBADC093-10 | HQ956751 | 58.7180 | -94.1240 |
| *Tetragnatha extensa* | I | BIOUG00627-B10 | SPIRU994-11 | KF368822 | 58.7177 | -94.1224 |
| *Tetragnatha extensa* | A | BIOUG00627-B11 | SPIRU995-11 | KF368877 | 58.7177 | -94.1224 |
| *Tetragnatha extensa* | A | BIOUG00627-B12 | SPIRU996-11 | KF368837 | 58.7177 | -94.1224 |
| *Tetragnatha extensa* | A | BIOUG00627-E04 | SPIRU1024-11 | KF368887 | 58.7045 | -94.1742 |
| *Tetragnatha extensa* | I | BIOUG00628-D01 | SPIRU1104-11 | KF368855 | 58.7304 | -93.7805 |
| *Tetragnatha extensa* | A | BIOUG00628-D02 | SPIRU1105-11 | KF368849 | 58.7304 | -93.7805 |
| *Tetragnatha extensa* | A | BIOUG00630-F12 | SPIRU1329-11 | KF368869 | 58.7305 | -93.7805 |
| *Tetragnatha extensa* | A | CHU-SPI-005 | KKCHE114-06 | KF368825 | 58.6180 | -93.8230 |
| *Tetragnatha extensa* | I | CHU-SPI-013 | KKCHE122-06 | KF368830 | 58.6220 | -93.8100 |
| *Tetragnatha extensa* | A | CHU-SPI-022 | KKCHE131-06 | KF368835 | 58.6820 | -94.1520 |
| *Tetragnatha extensa* | I | CHU-SPI-025 | KKCHE134-06 | KF368846 | 58.6180 | -93.8230 |
| *Tetragnatha extensa* | I | CHU-SPI-042 | KKCHE151-06 | KF368841 | 58.6180 | -93.8230 |
| *Tetragnatha extensa* | I | CHU-SPI-054 | KKCHE163-06 | KF368883 | 58.6220 | -93.8270 |
| *Tetragnatha extensa* | A | CHU-SPI-145 | KKCHE348-07 | KF368829 | 58.6170 | -93.8210 |
| *Tetragnatha extensa* | A | CHU-SPI-167 | KKCHE370-07 | KF368827 | 58.6170 | -93.8210 |
| *Tetragnatha extensa* | I | CHU-SPI-206 | KKCHE409-07 | KF368886 | 58.6220 | -93.8270 |
| *Tetragnatha extensa* | I | CHU-SPI-450 | KKCHE653-07 | KF368821 | 58.7460 | -94.1130 |
| *Tetragnatha extensa* | I | CHU-SPI-464 | KKCHE667-07 | KF368824 | 58.7400 | -93.8200 |
| *Tetragnatha extensa* | I | CHU-SPI-486 | KKCHE689-07 | KF368816 | 58.6200 | -93.8300 |
| *Tetragnatha extensa* | I | CHU-SPI-488 | KKCHE691-07 | KF368872 | 58.6290 | -93.7980 |
| *Tetragnatha extensa* | I | CHU-SPI-520 | KKCHE723-07 | KF368840 | 58.7470 | -94.1340 |
| *Tetragnatha extensa* | A | CHU-SPI-521 | KKCHE724-07 | KF368842 | 58.7470 | -94.1340 |
| *Tetragnatha extensa* | I | CHU-SPI-532 | KKCHE735-07 | KF368865 | 58.7470 | -94.1340 |
| *Tetragnatha extensa* | I | CHU-SPI-541 | KKCHE744-07 | KF368817 | 58.7470 | -94.1340 |
| *Tetragnatha extensa* | A | CHU-SPI-544 | KKCHE747-07 | KF368881 | 58.7470 | -94.1340 |
| *Tetragnatha extensa* | A | CHU-SPI-545 | KKCHE748-07 | KF368832 | 58.7470 | -94.1340 |
| *Tetragnatha extensa* | I | CHU-SPI-565 | KKCHE768-07 | KF368838 | 58.7470 | -94.1340 |
| *Tetragnatha extensa* | I | CHU-SPI-566 | KKCHE769-07 | KF368828 | 58.7470 | -94.1340 |
| *Tetragnatha extensa* | A | CHU-SPI-567 | KKCHE770-07 | KF368845 | 58.7470 | -94.1340 |
| *Tetragnatha extensa* | I | CHU-SPI-579 | KKCHE782-07 | KF368851 | 58.7470 | -94.1340 |
| *Tetragnatha extensa* | A | CHU-SPI-592 | KKCHE795-07 | KF368820 | 58.7470 | -94.1340 |
| *Tetragnatha extensa* | A | CHU-SPI-593 | KKCHE796-07 | KF368853 | 58.7470 | -94.1340 |
| *Tetragnatha extensa* | A | CHU-SPI-594 | KKCHE797-07 | KF368880 | 58.7470 | -94.1340 |
| *Tetragnatha extensa* | I | CHU-SPI-598 | KKCHE801-07 | KF368847 | 58.7470 | -94.1340 |
| *Tetragnatha extensa* | I | CHU-SPI-599 | KKCHE802-07 | KF368810 | 58.7470 | -94.1340 |
| *Tetragnatha extensa* | I | CHU-SPI-602 | KKCHE805-07 | KF368882 | 58.7470 | -94.1340 |
| *Tetragnatha extensa* | I | CHU-SPI-603 | KKCHE806-07 | KF368826 | 58.7470 | -94.1340 |
| *Tetragnatha extensa* | I | CHU-SPI-607 | KKCHE810-07 | KF368818 | 58.7470 | -94.1340 |
| *Tetragnatha extensa* | I | CHU-SPI-609 | KKCHE812-07 | KF368809 | 58.7470 | -94.1340 |
| *Tetragnatha extensa* | I | CHU-SPI-613 | KKCHE816-07 | KF368890 | 58.7470 | -94.1340 |
| *Tetragnatha extensa* | I | CHU-SPI-614 | KKCHE817-07 | KF368870 | 58.7470 | -94.1340 |
| *Tetragnatha extensa* | I | CHU-SPI-617 | KKCHE820-07 | KF368861 | 58.7470 | -94.1340 |
| *Tetragnatha extensa* | I | CHU-SPI-619 | KKCHE822-07 | KF368834 | 58.7470 | -94.1340 |
| *Tetragnatha extensa* | I | CHU-SPI-622 | KKCHE825-07 | KF368860 | 58.7470 | -94.1340 |
| *Tetragnatha extensa* | I | HLC13407 | KKCHE005-06 | KF368874 | 58.7640 | -93.8680 |
| *Tetragnatha extensa* | I | HLC13427 | KKCHE010-06 | KF368811 | 58.7640 | -93.8680 |
| *Tetragnatha extensa* | I | HLC13429 | KKCHE013-06 | KF368839 | 58.7640 | -93.8680 |
| *Tetragnatha extensa* | I | INV0070b | KKCHE099-06 | KF368885 | 58.7540 | -93.9140 |
| *Tetragnatha extensa* | I | INV0128 | KKCHE006-06 | KF368875 | 58.6320 | -93.7860 |
| *Tetragnatha extensa* | I | INV0140 | KKCHE003-06 | KF368813 | 58.7880 | -94.2030 |
| *Tetragnatha extensa* | I | INV0141 | KKCHE014-06 | KF368889 | 58.7880 | -94.2030 |
| *Tetragnatha extensa* | I | INV0143 | KKCHE012-06 | KF368831 | 58.7880 | -94.2030 |
| *Tetragnatha extensa* | I | INV0310 | KKCHE004-06 | KF368862 | 58.6320 | -93.7860 |
| *Tetragnatha extensa* | I | INV0325 | KKCHE011-06 | KF368854 | 58.6580 | -94.1670 |
| *Tetragnatha extensa* | I | JBWM0234242 | KKCHE282-07 | KF368858 | 58.6170 | -93.8170 |
| *Tetragnatha extensa* | I | JBWM0234245 | KKCHE283-07 | KF368844 | 58.7470 | -94.1340 |
| *Tetragnatha versicolor* | I | 07PROBE-04762 | ERSCH127-07 | KF368894 | 58.6300 | -93.7980 |
| *Tetragnatha versicolor* | I | 09PROBE-01578 | SWSWE008-09 | GU681067 | 58.6900 | -93.8500 |
| *Tetragnatha versicolor* | I | 09PROBE-01586 | SWSWE016-09 | GU681068 | 58.6190 | -93.8290 |
| *Tetragnatha versicolor* | I | 09PROBE-01607 | SWSWE037-09 | GU681054 | 58.6260 | -94.2290 |
| *Tetragnatha versicolor* | I | 09-PROBE-08117 | JDTGS021-09 | GU679838 | 58.6920 | -94.1320 |
| *Tetragnatha versicolor* | I | 09-PROBE-08167 | JDTGS071-09 | GU679833 | 58.6180 | -93.8290 |
| *Tetragnatha versicolor* | I | 09-PROBE-08176 | JDTGS080-09 | GU679834 | 58.6250 | -93.8190 |
| *Tetragnatha versicolor* | I | 09-PROBE-08177 | JDTGS081-09 | GU679831 | 58.6250 | -93.8190 |
| *Tetragnatha versicolor* | A | 09PROBE-1790-01 | SPICH204-09 | GU683911 | 58.6300 | -93.8190 |
| *Tetragnatha versicolor* | A | 09PROBE-596 | SPICH839-09 | GU684404 | 58.7310 | -93.7800 |
| *Tetragnatha versicolor* | I | BIOUG00628-C12 | SPIRU1103-11 | KF368895 | 58.7304 | -93.7805 |
| *Tetragnatha versicolor* | I | BIOUG00629-F08 | SPIRU1230-11 | KF368893 | 58.7720 | -93.8431 |
| *Tetragnatha versicolor* | I | CHU-SPI-081 | KKCHE190-06 | KF368892 | 58.6820 | -94.1520 |
| *Tetragnatha versicolor* | I | INV0417 | KKCHE015-06 | KF368891 | 58.6580 | -94.1670 |
| *Thanatus formicinus* | I | 07PROBE-02885 | TWSC271-08 | KF368899 | 58.7300 | -93.7840 |
| *Thanatus formicinus* | A | 09PROBE-1800-01 | SPICH209-09 | GU683936 | 58.6300 | -93.8190 |
| *Thanatus formicinus* | A | BIOUG00627-B03 | SPIRU987-11 | KF368898 | 58.7541 | -93.9974 |
| *Thanatus formicinus* | A | BIOUG00630-D01 | SPIRU1294-11 | KF368897 | 58.7770 | -94.1880 |
| *Thanatus formicinus* | I | HLC13393 | KKCHE068-06 | KF368896 | 58.7680 | -93.8680 |
| *Thanatus rubicellus* | I | 07CHU-AR-0256 | KKCHE1063-09 | GU683740 | 58.7700 | -93.8430 |
| *Thanatus rubicellus* | I | 07CHU-AR-0258 | KKCHE1065-09 | GU683743 | 58.1280 | -92.8560 |
| *Thanatus rubicellus* | A | 07CHU-AR-0260 | KKCHE1067-09 | GU683745 | 58.6620 | -93.1890 |
| *Thanatus rubicellus* | I | 07CHU-AR-0277 | KKCHE1084-09 | GU683597 | 58.7690 | -93.8620 |
| *Thanatus rubicellus* | I | 07PROBE-02828 | TWSC214-08 | KF368901 | 58.7310 | -93.7800 |
| *Thanatus rubicellus* | I | 07PROBE-02836 | TWSC222-08 | KF368908 | 58.7310 | -93.7800 |
| *Thanatus rubicellus* | I | 07PROBE-02842 | TWSC228-08 | KF368906 | 58.7310 | -93.7800 |
| *Thanatus rubicellus* | I | 07PROBE-02845 | TWSC231-08 | KF368902 | 58.7310 | -93.7800 |
| *Thanatus rubicellus* | I | 07PROBE-02872 | TWSC258-08 | KF368910 | 58.7310 | -93.7800 |
| *Thanatus rubicellus* | I | 07PROBE-02873 | TWSC259-08 | KF368905 | 58.7910 | -93.7540 |
| *Thanatus rubicellus* | I | 09-PROBE-08118 | JDTGS022-09 | GU679896 | 58.7850 | -93.7370 |
| *Thanatus rubicellus* | I | 09-PROBE-08119 | JDTGS023-09 | GU679839 | 58.7890 | -93.7100 |
| *Thanatus rubicellus* | A | 09PROBE-1670-01 | SPISH028-09 | GU683923 | 58.7640 | -93.8970 |
| *Thanatus rubicellus* | A | 09PROBE-1670-02 | SPISH029-09 | GU683924 | 58.7640 | -93.8970 |
| *Thanatus rubicellus* | A | 09PROBE-1796-01 | SPICH207-09 | GU683922 | 58.6300 | -93.8190 |
| *Thanatus rubicellus* | I | 09PROBE-1978-547 | SPICH790-09 | GU684392 | 58.7870 | -93.7140 |
| *Thanatus rubicellus* | A | BIOUG00629-G12 | SPIRU1246-11 | KF368907 | 58.7710 | -93.8510 |
| *Thanatus rubicellus* | A | BIOUG00632-A12 | SPIRU1364-11 | KF368911 | 58.7690 | -93.8620 |
| *Thanatus rubicellus* | I | JBWM0006188 | KKCHE276-07 | KF368904 | 58.7540 | -93.9130 |
| *Thanatus rubicellus* | I | JBWM0006189 | KKCHE277-07 | KF368903 | 58.7540 | -93.9130 |
| *Thanatus rubicellus* | I | SD2406SG105 | SAPIT116-08 | KF368900 | 58.7300 | -93.8000 |
| *Thanatus rubicellus* | I | SD2406SH501 | SAPIT159-08 | KF368909 | 58.7300 | -93.8000 |
| *Theridion pictum* | I | 09-PROBE-08114 | JDTGS018-09 | GU679898 | 58.6920 | -94.1320 |
| *Theridion pictum* | A | 09PROBE-1978-01 | SPICH244-09 | GU684344 | 58.6690 | -94.1600 |
| *Theridion pictum* | A | 09PROBE-1978-02 | SPICH245-09 | GU684345 | 58.6690 | -94.1600 |
| *Theridion pictum* | A | 09PROBE-1978-122 | SPICH365-09 | GU684271 | 58.6690 | -94.1600 |
| *Theridion pictum* | A | 09PROBE-799 | SPICH1020-09 | GU684698 | 58.6260 | -94.2300 |
| *Theridion pictum* | I | 10PROBE-21146 | GBADC026-10 | HQ956689 | 58.7340 | -94.1120 |
| *Theridion pictum* | I | BIOUG00628-F02 | SPIRU1129-11 | KF368915 | 58.7544 | -93.9980 |
| *Theridion pictum* | A | BIOUG00628-F05 | SPIRU1132-11 | KF368914 | 58.7055 | -94.0536 |
| *Theridion pictum* | I | BIOUG00629-A03 | SPIRU1165-11 | KF368913 | 58.7306 | -93.7804 |
| *Theridion pictum* | I | CHU-SPI-244 | KKCHE447-07 | KF368912 | 58.6910 | -94.1790 |
| *Thymoites oleatus* | I | 09PROBE-1978-134 | SPICH377-09 | GU684268 | 58.6690 | -94.1600 |
| *Thymoites oleatus* | I | 09PROBE-1978-95 | SPICH338-09 | GU684347 | 58.6690 | -94.1600 |
| *Tibellus maritimus* | A | 09PROBE-688 | SPICH931-09 | GU684489 | 58.7310 | -93.7800 |
| *Tibellus maritimus* | I | 10PROBE-21166 | GBADC046-10 | HQ956707 | 58.7340 | -94.1120 |
| *Tibellus maritimus* | I | 10PROBE-21176 | GBADC056-10 | HQ956717 | 58.6340 | -93.7860 |
| *Tibellus maritimus* | I | 10PROBE-21177 | GBADC057-10 | HQ956718 | 58.6340 | -93.7860 |
| *Tibellus maritimus* | I | 10PROBE-21194 | GBADC074-10 | HQ956734 | 58.6610 | -93.8320 |
| *Tibellus maritimus* | I | 10PROBE-21198 | GBADC078-10 | HQ956738 | 58.6610 | -93.8320 |
| *Tibellus maritimus* | I | BIOUG00627-A08 | SPIRU980-11 | KF368916 | 58.6340 | -93.7860 |
| *Tibellus maritimus* | A | BIOUG00628-B07 | SPIRU1086-11 | KF368917 | 58.7340 | -94.1120 |
| *Tibellus maritimus* | I | CHU-SPI-474 | KKCHE677-07 | KF368921 | 58.6200 | -93.8300 |
| *Tibellus maritimus* | I | JBWM0234318f | KKCHE302-07 | KF368918 | 58.6290 | -93.7980 |
| *Tibellus maritimus* | I | JBWM0234318m | KKCHE301-07 | KF368920 | 58.6290 | -93.7980 |
| *Tibellus maritimus* | I | SD0406SH104 | SAPIT189-08 | KF368919 | 58.7300 | -93.8000 |
| *Tiso aestivus* | A | 07CHU-AR-0250 | KKCHE1057-09 | GU683735 | 58.6300 | -93.7980 |
| *Tiso aestivus* | A | 09PROBE-1861-01 | SPICH030-09 | GU683851 | 58.6300 | -93.8190 |
| *Tiso aestivus* | A | 09PROBE-1865-01 | SPICH034-09 | GU683852 | 58.6300 | -93.8190 |
| *Tiso aestivus* | I | 09PROBE-1978-141 | SPICH384-09 | GU684223 | 58.7310 | -93.7800 |
| *Tiso aestivus* | I | 09PROBE-1978-142 | SPICH385-09 | GU684224 | 58.7310 | -93.7800 |
| *Tiso aestivus* | I | 09PROBE-1978-143 | SPICH386-09 | GU684221 | 58.7310 | -93.7800 |
| *Tiso aestivus* | A | 09PROBE-1978-156 | SPICH399-09 | GU684222 | 58.7310 | -93.7800 |
| *Tiso aestivus* | A | CHU-SPI-546 | KKCHE749-07 | KF368922 | 58.7380 | -93.8190 |
| *Tmeticus ornatus* | A | 09PROBE-1978-51 | SPICH294-09 | GU684294 | 58.6690 | -94.1600 |
| *Tmeticus ornatus* | A | BIOUG00627-E01 | SPIRU1021-11 | KF368927 | 58.7544 | -93.9980 |
| *Tmeticus ornatus* | A | BIOUG00628-E05 | SPIRU1120-11 | KF368926 | 58.7574 | -93.9009 |
| *Tmeticus ornatus* | I | BIOUG00628-H05 | SPIRU1156-11 | KF368923 | 58.7545 | -93.9136 |
| *Tmeticus ornatus* | A | BIOUG00629-B05 | SPIRU1179-11 | KF368924 | 58.7544 | -93.9980 |
| *Tmeticus ornatus* | I | CHU-SPI-568 | KKCHE771-07 | KF368925 | 58.7470 | -94.1340 |
| *Tunagyna debilis* | A | 07CHU-AR-0032 | KKCHE944-09 | GU683647 | 58.6780 | -94.1460 |
| *Tunagyna debilis* | A | 09PROBE-1978-178 | SPICH421-09 | GU684193 | 58.6260 | -94.2300 |
| *Tunagyna debilis* | A | 09PROBE-1978-334 | SPICH577-09 | GU684138 | 58.6260 | -94.2300 |
| *Tunagyna debilis* | I | 09PROBE-887 | SPICH1108-09 | GU684637 | 58.6260 | -94.2300 |
| *Tunagyna debilis* | A | BIOUG00630-G07 | SPIRU1336-11 | KF368928 | 58.6340 | -93.7860 |
| *Tunagyna debilis* | A | BIOUG00630-H04 | SPIRU1345-11 | KF368929 | 58.7920 | -93.7510 |
| *Typhochrestus pygmaeus* | I | 09PROBE-660 | SPICH903-09 | HM416917 | 58.7960 | -93.7540 |
| *Wabasso cacuminatus* | A | 09PROBE-1978-174 | SPICH417-09 | HM432630 | 58.6180 | -93.8290 |
| *Wabasso cacuminatus* | A | 09PROBE-1978-182 | SPICH425-09 | GU684196 | 58.6260 | -94.2300 |
| *Wabasso cacuminatus* | A | 09PROBE-1978-298 | SPICH541-09 | GU684183 | 58.6690 | -94.1600 |
| *Wabasso cacuminatus* | A | BIOUG00630-G06 | SPIRU1335-11 | KF368932 | 58.6340 | -93.7860 |
| *Wabasso cacuminatus* | I | SD2406SG305 | SAPIT131-08 | KF368933 | 58.7300 | -93.8000 |
| *Wabasso cacuminatus* | I | SD2406SG502 | SAPIT137-08 | KF368930 | 58.7300 | -93.8000 |
| *Wabasso cacuminatus* | A | SD2406SH105 | SAPIT146-08 | KF368931 | 58.7300 | -93.8000 |
| *Wabasso quaestio* | A | 09PROBE-1978-180 | SPICH423-09 | GU684195 | 58.6260 | -94.2300 |
| *Wabasso quaestio* | I | 09PROBE-1978-318 | SPICH561-09 | GU684140 | 58.6690 | -94.1600 |
| *Wabasso quaestio* | I | 09PROBE-1978-319 | SPICH562-09 | GU684181 | 58.6690 | -94.1600 |
| *Wabasso quaestio* | A | 09PROBE-1978-337 | SPICH580-09 | GU684137 | 58.6260 | -94.2300 |
| *Walckenaeria castanea* | A | 09PROBE-1978-393 | SPICH636-09 | GU684538 | 58.6250 | -93.8160 |
| *Walckenaeria castanea* | A | 09PROBE-582 | SPICH825-09 | GU684446 | 58.7310 | -93.7800 |
| *Walckenaeria castanea* | I | SD2707SH503 | SAPIT039-08 | KF368934 | 58.7300 | -93.8000 |
| *Walckenaeria communis* | I | 09PROBE-1826-01 | SPICH238-09 | GU683978 | 58.6300 | -93.8190 |
| *Walckenaeria communis* | A | 09PROBE-1868-01 | SPICH037-09 | GU683866 | 58.6300 | -93.8190 |
| *Walckenaeria communis* | A | 09PROBE-1871-01 | SPICH040-09 | GU683867 | 58.6300 | -93.8190 |
| *Walckenaeria communis* | A | 09PROBE-1978-225 | SPICH468-09 | GU684074 | 58.6300 | -93.7980 |
| *Walckenaeria communis* | A | 09PROBE-1978-231 | SPICH474-09 | GU684075 | 58.6300 | -93.7980 |
| *Walckenaeria communis* | A | 09PROBE-592 | SPICH835-09 | GU684450 | 58.7900 | -94.2270 |
| *Walckenaeria communis* | A | 09PROBE-593 | SPICH836-09 | GU684407 | 58.7900 | -94.2270 |
| *Walckenaeria communis* | A | 09PROBE-647 | SPICH890-09 | GU684449 | 58.6300 | -93.7980 |
| *Walckenaeria communis* | A | BIOUG00630-G09 | SPIRU1338-11 | KF368937 | 58.6173 | -93.8123 |
| *Walckenaeria communis* | I | CHU-SPI-328 | KKCHE555-07 | KF368936 | 58.6910 | -94.1790 |
| *Walckenaeria communis* | I | CHU-SPI-360 | KKCHE535-07 | KF368935 | 58.6320 | -93.7860 |
| *Walckenaeria exigua* | A | 09PROBE-1837-01 | SPICH006-09 | GU683863 | 58.6300 | -93.8190 |
| *Walckenaeria exigua* | A | 09PROBE-1851-01 | SPICH020-09 | GU683864 | 58.6300 | -93.8190 |
| *Walckenaeria karpinskii* | A | 09PROBE-1633-01 | SPISH006-09 | GU683980 | 58.7960 | -93.7540 |
| *Walckenaeria karpinskii* | A | 09PROBE-1946-01 | SPICH085-09 | GU683868 | 58.7640 | -93.8970 |
| *Walckenaeria karpinskii* | A | 09PROBE-1978-430 | SPICH673-09 | GU684539 | 58.7640 | -93.8970 |
| *Walckenaeria karpinskii* | A | BIOUG00629-G05 | SPIRU1239-11 | KF368938 | 58.7720 | -93.8431 |
| *Walckenaeria kochi* | A | 09PROBE-1919-01 | SPICH071-09 | GU683871 | 58.7630 | -93.8660 |
| *Walckenaeria kochi* | A | 09PROBE-1920-01 | SPICH072-09 | GU683870 | 58.7630 | -93.8660 |
| *Walckenaeria kochi* | A | 09PROBE-1947-01 | SPICH086-09 | GU683869 | 58.7640 | -93.8970 |
| *Walckenaeria kochi* | I | 09PROBE-1978-363 | SPICH606-09 | GU684155 | 58.7640 | -93.8970 |
| *Walckenaeria kochi* | A | 09PROBE-1978-364 | SPICH607-09 | GU684156 | 58.7640 | -93.8970 |
| *Walckenaeria kochi* | I | 09PROBE-1978-432 | SPICH675-09 | GU684541 | 58.7640 | -93.8970 |
| *Walckenaeria kochi* | A | 09PROBE-1978-442 | SPICH685-09 | GU684540 | 58.7640 | -93.8970 |
| *Walckenaeria kochi* | A | 09PROBE-1978-446 | SPICH689-09 | GU684542 | 58.7640 | -93.8970 |
| *Walckenaeria kochi* | I | 09PROBE-742 | SPICH985-09 | GU684470 | 58.6260 | -94.2300 |
| *Walckenaeria kochi* | A | BIOUG00627-D09 | SPIRU1017-11 | KF368939 | 58.7544 | -93.9980 |
| *Walckenaeria lepida* | A | 07CHU-AR-0005 | KKCHE917-09 | GU683625 | 58.6170 | -93.8120 |
| *Walckenaeria lepida* | A | 07CHU-AR-0006 | KKCHE918-09 | GU683624 | 58.6170 | -93.8120 |
| *Walckenaeria lepida* | A | BIOUG00627-A03 | SPIRU975-11 | KF368943 | 58.7180 | -94.1240 |
| *Walckenaeria lepida* | A | BIOUG00629-D10 | SPIRU1208-11 | KF368941 | 58.6173 | -93.8123 |
| *Walckenaeria lepida* | I | CHU-SPI-012 | KKCHE121-06 | KF368944 | 58.6220 | -93.8100 |
| *Walckenaeria lepida* | I | CHU-SPI-024 | KKCHE133-06 | KF368940 | 58.6180 | -93.8230 |
| *Walckenaeria lepida* | I | CHU-SPI-074 | KKCHE183-06 | KF368942 | 58.6220 | -93.8100 |
| *Walckenaeria palustris* | A | 09PROBE-1831-01 | SPICH243-09 | GU683979 | 58.6300 | -93.8190 |
| *Walckenaeria palustris* | A | 09PROBE-1838-01 | SPICH007-09 | GU683865 | 58.6300 | -93.8190 |
| *Walckenaeria palustris* | A | 09PROBE-1841-01 | SPICH010-09 | GU683830 | 58.6300 | -93.8190 |
| *Walckenaeria palustris* | A | 09PROBE-1978-213 | SPICH456-09 | GU684076 | 58.6300 | -93.7980 |
| *Walckenaeria palustris* | A | 09PROBE-1978-215 | SPICH458-09 | GU684077 | 58.6300 | -93.7980 |
| *Walckenaeria palustris* | A | 09PROBE-1978-221 | SPICH464-09 | GU684079 | 58.6300 | -93.7980 |
| *Walckenaeria spiralis* | A | 09PROBE-1869-01 | SPICH038-09 | HM432623 | 58.6300 | -93.8190 |
| *Walckenaeria spiralis* | A | BIOUG00627-C12 | SPIRU1008-11 | KF368945 | 58.7544 | -93.9980 |
| *Xysticus britcheri* | I | 07PROBE-02820 | TWSC206-08 | KF368974 | 58.7310 | -93.7800 |
| *Xysticus britcheri* | A | 07PROBE-02830 | TWSC216-08 | KF368985 | 58.7310 | -93.7800 |
| *Xysticus britcheri* | A | 07PROBE-02838 | TWSC224-08 | KF368980 | 58.7310 | -93.7800 |
| *Xysticus britcheri* | A | 07PROBE-02852 | TWSC238-08 | KF368965 | 58.7310 | -93.7800 |
| *Xysticus britcheri* | A | 07PROBE-06520 | TWSC034-07 | KF368960 | 58.7310 | -93.7800 |
| *Xysticus britcheri* | A | 07PROBE-06521 | TWSC035-07 | KF368958 | 58.7600 | -94.0860 |
| *Xysticus britcheri* | A | 07PROBE-06523 | TWSC037-07 | KF368969 | 58.7310 | -93.7800 |
| *Xysticus britcheri* | A | 07PROBE-06524 | TWSC038-07 | KF368983 | 58.7310 | -93.7800 |
| *Xysticus britcheri* | A | 07PROBE-06525 | TWSC039-07 | KF368956 | 58.7050 | -94.0520 |
| *Xysticus britcheri* | A | 07PROBE-06526 | TWSC040-07 | KF368962 | 58.7370 | -93.8230 |
| *Xysticus britcheri* | A | 07PROBE-06527 | TWSC041-07 | KF368954 | 58.7600 | -94.0860 |
| *Xysticus britcheri* | A | 07PROBE-06528 | TWSC042-07 | KF368990 | 58.7050 | -94.0520 |
| *Xysticus britcheri* | A | 07PROBE-06537 | TWSC051-07 | KF368976 | 58.7310 | -93.7800 |
| *Xysticus britcheri* | I | 09-PROBE-08110 | JDTGS014-09 | GU679900 | 58.7850 | -93.7370 |
| *Xysticus britcheri* | A | BIOUG00627-C03 | SPIRU999-11 | KF368975 | 58.7544 | -93.9980 |
| *Xysticus britcheri* | I | BIOUG00627-D05 | SPIRU1013-11 | KF368959 | 58.7544 | -93.9980 |
| *Xysticus britcheri* | I | BIOUG00627-D06 | SPIRU1014-11 | KF368968 | 58.7544 | -93.9980 |
| *Xysticus britcheri* | I | BIOUG00627-G07 | SPIRU1051-11 | KF368963 | 58.7574 | -94.1509 |
| *Xysticus britcheri* | I | BIOUG00627-G08 | SPIRU1052-11 | KF368946 | 58.7574 | -94.1509 |
| *Xysticus britcheri* | A | BIOUG00628-D03 | SPIRU1106-11 | KF368971 | 58.7304 | -93.7805 |
| *Xysticus britcheri* | I | BIOUG00628-E02 | SPIRU1117-11 | KF368978 | 58.7544 | -93.9980 |
| *Xysticus britcheri* | I | BIOUG00628-E03 | SPIRU1118-11 | KF368989 | 58.7544 | -93.9980 |
| *Xysticus britcheri* | A | BIOUG00628-E04 | SPIRU1119-11 | KF368953 | 58.7544 | -93.9980 |
| *Xysticus britcheri* | A | BIOUG00628-E10 | SPIRU1125-11 | KF368993 | 58.7304 | -93.7805 |
| *Xysticus britcheri* | A | BIOUG00628-E11 | SPIRU1126-11 | KF368970 | 58.7304 | -93.7805 |
| *Xysticus britcheri* | A | BIOUG00628-E12 | SPIRU1127-11 | KF368981 | 58.7304 | -93.7805 |
| *Xysticus britcheri* | A | BIOUG00628-F01 | SPIRU1128-11 | KF368979 | 58.7544 | -93.9980 |
| *Xysticus britcheri* | I | BIOUG00628-H07 | SPIRU1158-11 | KF368961 | 58.7304 | -93.7805 |
| *Xysticus britcheri* | I | BIOUG00629-F01 | SPIRU1223-11 | KF368982 | 58.7710 | -93.8510 |
| *Xysticus britcheri* | A | BIOUG00629-F03 | SPIRU1225-11 | KF368986 | 58.7710 | -93.8510 |
| *Xysticus britcheri* | A | BIOUG00629-F04 | SPIRU1226-11 | KF368949 | 58.7710 | -93.8510 |
| *Xysticus britcheri* | A | BIOUG00629-F05 | SPIRU1227-11 | KF368973 | 58.7710 | -93.8510 |
| *Xysticus britcheri* | A | BIOUG00629-G09 | SPIRU1243-11 | KF368957 | 58.7710 | -93.8510 |
| *Xysticus britcheri* | A | BIOUG00630-B09 | SPIRU1278-11 | KF368948 | 58.7690 | -93.8620 |
| *Xysticus britcheri* | A | BIOUG00630-C07 | SPIRU1288-11 | KF368992 | 58.7710 | -93.8510 |
| *Xysticus britcheri* | A | BIOUG00630-D03 | SPIRU1296-11 | KF368951 | 58.7770 | -94.1880 |
| *Xysticus britcheri* | A | BIOUG00630-E12 | SPIRU1317-11 | KF368977 | 58.7720 | -93.8431 |
| *Xysticus britcheri* | I | CHU-SPI-363 | KKCHE544-07 | KF368987 | 58.6320 | -93.7860 |
| *Xysticus britcheri* | I | CHU-SPI-466 | KKCHE669-07 | KF368950 | 58.7380 | -93.8190 |
| *Xysticus britcheri* | I | CHU-SPI-467 | KKCHE670-07 | KF368984 | 58.7380 | -93.8190 |
| *Xysticus britcheri* | I | CHU-SPI-468 | KKCHE671-07 | KF368967 | 58.7380 | -93.8190 |
| *Xysticus britcheri* | I | CHU-SPI-490 | KKCHE693-07 | KF368991 | 58.7470 | -94.1340 |
| *Xysticus britcheri* | I | CHU-SPI-502 | KKCHE705-07 | KF368994 | 58.7470 | -94.1340 |
| *Xysticus britcheri* | I | CHU-SPI-505 | KKCHE708-07 | KF368964 | 58.7470 | -94.1340 |
| *Xysticus britcheri* | I | CHU-SPI-506 | KKCHE709-07 | KF368952 | 58.7470 | -94.1340 |
| *Xysticus britcheri* | I | CHU-SPI-540 | KKCHE743-07 | KF368972 | 58.7470 | -94.1340 |
| *Xysticus britcheri* | I | HLC-26868 | KKCHE899-09 | KF368995 | 58.7650 | -93.9970 |
| *Xysticus britcheri* | A | HLC-26881 | KKCHE912-09 | KF368966 | 58.7310 | -93.7800 |
| *Xysticus britcheri* | I | JBWM0234259 | KKCHE284-07 | KF368955 | 58.7540 | -93.9130 |
| *Xysticus britcheri* | I | JBWM0234266 | KKCHE285-07 | KF368988 | 58.7470 | -94.1340 |
| *Xysticus britcheri* | I | SD0406HE101 | SAPIT161-08 | KF368947 | 58.7300 | -93.8000 |
| *Xysticus canadensis* | I | CHU-SPI-014 | KKCHE123-06 | KF368996 | 58.6180 | -93.8230 |
| *Xysticus deichmanni* | I | 07CHU-AR-0269 | KKCHE1076-09 | GU683753 | 58.1280 | -92.8560 |
| *Xysticus deichmanni* | A | 07CHU-AR-0271 | KKCHE1078-09 | GU683755 | 58.1280 | -92.8560 |
| *Xysticus deichmanni* | I | 09PROBE-1978-546 | SPICH789-09 | GU684397 | 58.7870 | -93.7140 |
| *Xysticus deichmanni* | A | BIOUG00630-C08 | SPIRU1289-11 | KF368997 | 58.7710 | -93.8510 |
| *Xysticus deichmanni* | A | CCDB-04289-G11 | SPACH008-09 | GU683612 | 58.7600 | -94.0860 |
| *Xysticus deichmanni* | A | CCDB-04289-H02 | SPACH011-09 | GU683613 | 58.7600 | -94.0860 |
| *Xysticus deichmanni* | A | CCDB-04289-H05 | SPACH014-09 | GU683616 | 58.7600 | -94.0860 |
| *Xysticus deichmanni* | A | CCDB-04359-H05 | SPACH001-09 | GU683604 | 58.7640 | -94.0870 |
| *Xysticus deichmanni* | A | CCDB-04359-H06 | SPACH002-09 | GU683606 | 58.7640 | -94.0870 |
| *Xysticus deichmanni* | A | CCDB-04359-H11 | SPACH007-09 | GU683609 | 58.7600 | -94.0860 |
| *Xysticus deichmanni* | I | HLC-26798 | KKCHE829-09 | KF369002 | 58.7800 | -94.1860 |
| *Xysticus deichmanni* | I | HLC-26813 | KKCHE844-09 | KF369001 | 58.7800 | -94.1860 |
| *Xysticus deichmanni* | I | HLC-26833 | KKCHE864-09 | KF368999 | 58.7800 | -94.1860 |
| *Xysticus deichmanni* | I | HLC-26844 | KKCHE875-09 | KF368998 | 58.1300 | -92.9000 |
| *Xysticus deichmanni* | I | HLC-26846 | KKCHE877-09 | KF369000 | 58.1300 | -92.9000 |
| *Xysticus durus* | I | 07CHU-AR-0231 | KKCHE1038-09 | GU683723 | 58.6260 | -94.2300 |
| *Xysticus durus* | I | 07CHU-AR-0232 | KKCHE1039-09 | GU683722 | 58.6260 | -94.2300 |
| *Xysticus durus* | A | 07CHU-AR-0266 | KKCHE1073-09 | GU683750 | 58.7380 | -93.8190 |
| *Xysticus durus* | I | 07CHU-AR-0267 | KKCHE1074-09 | GU683749 | 58.7380 | -93.8190 |
| *Xysticus durus* | I | 07CHU-AR-0272 | KKCHE1079-09 | GU683594 | 58.7600 | -93.9630 |
| *Xysticus durus* | A | 07PROBE-02829 | TWSC215-08 | KF369024 | 58.7310 | -93.7800 |
| *Xysticus durus* | A | 07PROBE-02840 | TWSC226-08 | KF369046 | 58.7310 | -93.7800 |
| *Xysticus durus* | A | 07PROBE-02854 | TWSC240-08 | KF369041 | 58.7600 | -94.0860 |
| *Xysticus durus* | I | 07PROBE-02858 | TWSC244-08 | KF369013 | 58.7600 | -94.0860 |
| *Xysticus durus* | I | 07PROBE-02880 | TWSC266-08 | KF369027 | 58.7310 | -93.7800 |
| *Xysticus durus* | I | 07PROBE-06496 | TWSC010-07 | KF369019 | 58.7570 | -93.9840 |
| *Xysticus durus* | A | 07PROBE-06529 | TWSC043-07 | KF369021 | 58.7570 | -93.9840 |
| *Xysticus durus* | A | 07PROBE-06530 | TWSC044-07 | KF369022 | 58.7310 | -93.7800 |
| *Xysticus durus* | I | 07PROBE-06531 | TWSC045-07 | KF369042 | 58.7600 | -94.0860 |
| *Xysticus durus* | A | 07PROBE-06532 | TWSC046-07 | KF369023 | 58.7370 | -93.8230 |
| *Xysticus durus* | A | 07PROBE-06533 | TWSC047-07 | KF369032 | 58.7310 | -93.8150 |
| *Xysticus durus* | A | 07PROBE-06534 | TWSC048-07 | KF369043 | 58.7370 | -93.8230 |
| *Xysticus durus* | A | 07PROBE-06535 | TWSC049-07 | KF369004 | 58.7600 | -94.0860 |
| *Xysticus durus* | A | 07PROBE-06536 | TWSC050-07 | KF369005 | 58.7310 | -93.7800 |
| *Xysticus durus* | A | 07PROBE-06538 | TWSC052-07 | KF369026 | 58.7570 | -93.9840 |
| *Xysticus durus* | A | 09PROBE-1791-01 | SPICH205-09 | GU683926 | 58.6300 | -93.8190 |
| *Xysticus durus* | I | 09PROBE-1893-01 | SPICH058-09 | GU683896 | 58.7700 | -93.8430 |
| *Xysticus durus* | A | 09PROBE-1978-488 | SPICH731-09 | GU684350 | 58.6300 | -94.2300 |
| *Xysticus durus* | I | BIOUG00627-G02 | SPIRU1046-11 | KF369030 | 58.6329 | -93.7869 |
| *Xysticus durus* | A | BIOUG00628-A09 | SPIRU1076-11 | KF369011 | 58.7616 | -93.9295 |
| *Xysticus durus* | I | BIOUG00629-E07 | SPIRU1217-11 | KF369034 | 58.7660 | -93.8680 |
| *Xysticus durus* | A | BIOUG00629-E08 | SPIRU1218-11 | KF369016 | 58.7660 | -93.8680 |
| *Xysticus durus* | A | BIOUG00629-F02 | SPIRU1224-11 | KF369044 | 58.7710 | -93.8510 |
| *Xysticus durus* | A | BIOUG00629-G08 | SPIRU1242-11 | KF369038 | 58.7710 | -93.8510 |
| *Xysticus durus* | A | BIOUG00630-B10 | SPIRU1279-11 | KF369040 | 58.7690 | -93.8620 |
| *Xysticus durus* | A | BIOUG00630-D02 | SPIRU1295-11 | KF369036 | 58.7770 | -94.1880 |
| *Xysticus durus* | A | BIOUG00630-D06 | SPIRU1299-11 | KF369003 | 58.7305 | -93.7805 |
| *Xysticus durus* | I | BIOUG00630-F01 | SPIRU1318-11 | KF369029 | 58.7720 | -93.8431 |
| *Xysticus durus* | I | BIOUG00633-C02 | SPIRU1414-11 | KF369045 | 58.7710 | -93.8510 |
| *Xysticus durus* | I | CCDB-04289-G12 | SPACH009-09 | GU683611 | 58.7600 | -94.0860 |
| *Xysticus durus* | I | CHU-SPI-408 | KKCHE611-07 | KF369037 | 58.7380 | -93.8190 |
| *Xysticus durus* | I | CHU-SPI-420 | KKCHE623-07 | KF369017 | 58.7380 | -93.8190 |
| *Xysticus durus* | I | CHU-SPI-431 | KKCHE634-07 | KF369039 | 58.7380 | -93.8190 |
| *Xysticus durus* | I | CHU-SPI-456 | KKCHE659-07 | KF369031 | 58.7380 | -93.8190 |
| *Xysticus durus* | A | CHU-SPI-457 | KKCHE660-07 | KF369020 | 58.7380 | -93.8190 |
| *Xysticus durus* | I | CHU-SPI-465 | KKCHE668-07 | KF369014 | 58.7380 | -93.8190 |
| *Xysticus durus* | I | CHU-SPI-480 | KKCHE683-07 | KF369009 | 58.7380 | -93.8190 |
| *Xysticus durus* | I | CHU-SPI-498 | KKCHE701-07 | KF369010 | 58.7380 | -93.8190 |
| *Xysticus durus* | I | CHU-SPI-527 | KKCHE730-07 | KF369028 | 58.7470 | -94.1340 |
| *Xysticus durus* | I | CHU-SPI-531 | KKCHE734-07 | KF369012 | 58.7470 | -94.1340 |
| *Xysticus durus* | I | CHU-SPI-547 | KKCHE750-07 | KF369018 | 58.7380 | -93.8190 |
| *Xysticus durus* | A | CHU-SPI-573 | KKCHE776-07 | KF369006 | 58.7470 | -94.1340 |
| *Xysticus durus* | I | CHU-SPI-583 | KKCHE786-07 | KF369015 | 58.7380 | -93.8190 |
| *Xysticus durus* | I | HLC-26807 | KKCHE838-09 | KF369035 | 58.7800 | -94.1860 |
| *Xysticus durus* | I | HLC-26834 | KKCHE865-09 | KF369007 | 58.7800 | -94.1860 |
| *Xysticus durus* | I | HLC-26869 | KKCHE900-09 | KF369025 | 58.7650 | -93.9970 |
| *Xysticus durus* | I | JBWM0234272 | KKCHE287-07 | KF369033 | 58.7710 | -93.8420 |
| *Xysticus durus* | I | JBWM0234278 | KKCHE288-07 | KF369008 | 58.7380 | -93.8190 |
| *Xysticus ellipticus* | A | 09PROBE-1656-01 | SPISH011-09 | GU683928 | 58.6180 | -93.8290 |
| *Xysticus ellipticus* | A | 09PROBE-1682-01 | SPICH116-09 | GU683801 | 58.6300 | -93.7980 |
| *Xysticus ellipticus* | A | 09PROBE-1682-02 | SPICH117-09 | GU683798 | 58.6300 | -93.7980 |
| *Xysticus labradorensis* | I | 07CHU-AR-0268 | KKCHE1075-09 | GU683754 | 58.1280 | -92.8560 |
| *Xysticus labradorensis* | A | 07CHU-AR-0270 | KKCHE1077-09 | GU683752 | 58.1280 | -92.8560 |
| *Xysticus labradorensis* | A | 07PROBE-02889 | TWSC275-08 | KF369058 | 58.7600 | -94.0860 |
| *Xysticus labradorensis* | A | 07PROBE-06508 | TWSC022-07 | KF369061 | 58.7600 | -94.0860 |
| *Xysticus labradorensis* | A | 07PROBE-06509 | TWSC023-07 | KF369047 | 58.7600 | -94.0860 |
| *Xysticus labradorensis* | A | BIOUG00629-G11 | SPIRU1245-11 | KF369057 | 58.7710 | -93.8510 |
| *Xysticus labradorensis* | I | CCDB-04289-H01 | SPACH010-09 | GU683614 | 58.7600 | -94.0860 |
| *Xysticus labradorensis* | I | CCDB-04289-H03 | SPACH012-09 | GU683618 | 58.7600 | -94.0860 |
| *Xysticus labradorensis* | I | CCDB-04289-H04 | SPACH013-09 | GU683617 | 58.7600 | -94.0860 |
| *Xysticus labradorensis* | I | CCDB-04289-H06 | SPACH015-09 | GU683615 | 58.7600 | -94.0860 |
| *Xysticus labradorensis* | I | CCDB-04289-H07 | SPACH016-09 | GU683622 | 58.7600 | -94.0860 |
| *Xysticus labradorensis* | I | CCDB-04289-H08 | SPACH017-09 | GU683621 | 58.7600 | -94.0860 |
| *Xysticus labradorensis* | I | CCDB-04289-H09 | SPACH018-09 | GU683620 | 58.7640 | -94.0870 |
| *Xysticus labradorensis* | I | CCDB-04289-H10 | SPACH019-09 | GU683619 | 58.7640 | -94.0870 |
| *Xysticus labradorensis* | I | CCDB-04289-H11 | SPACH020-09 | GU683623 | 58.7640 | -94.0870 |
| *Xysticus labradorensis* | I | CCDB-04359-H07 | SPACH003-09 | GU683605 | 58.7640 | -94.0870 |
| *Xysticus labradorensis* | I | CCDB-04359-H08 | SPACH004-09 | GU683607 | 58.7640 | -94.0870 |
| *Xysticus labradorensis* | I | CCDB-04359-H09 | SPACH005-09 | GU683608 | 58.7640 | -94.0870 |
| *Xysticus labradorensis* | I | CCDB-04359-H10 | SPACH006-09 | GU683610 | 58.7600 | -94.0860 |
| *Xysticus labradorensis* | I | HLC-26800 | KKCHE831-09 | KF369062 | 58.7800 | -94.1860 |
| *Xysticus labradorensis* | I | HLC-26801 | KKCHE832-09 | KF369054 | 58.7800 | -94.1860 |
| *Xysticus labradorensis* | I | HLC-26803 | KKCHE834-09 | KF369052 | 58.7800 | -94.1860 |
| *Xysticus labradorensis* | A | HLC-26805 | KKCHE836-09 | KF369056 | 58.7800 | -94.1860 |
| *Xysticus labradorensis* | I | HLC-26815 | KKCHE846-09 | KF369055 | 58.7800 | -94.1860 |
| *Xysticus labradorensis* | I | HLC-26818 | KKCHE849-09 | KF369060 | 58.7800 | -94.1860 |
| *Xysticus labradorensis* | I | HLC-26825 | KKCHE856-09 | KF369049 | 58.7800 | -94.1860 |
| *Xysticus labradorensis* | I | HLC-26831 | KKCHE862-09 | KF369048 | 58.7800 | -94.1860 |
| *Xysticus labradorensis* | I | HLC-26841 | KKCHE872-09 | KF369051 | 58.7800 | -94.1860 |
| *Xysticus labradorensis* | I | HLC-26843 | KKCHE874-09 | KF369050 | 58.1300 | -92.9000 |
| *Xysticus labradorensis* | I | HLC-26845 | KKCHE876-09 | KF369059 | 58.1300 | -92.9000 |
| *Xysticus labradorensis* | I | JBWM0234306 | KKCHE290-07 | KF369053 | 58.7650 | -93.9840 |
| *Xysticus luctuosus* | A | 09PROBE-1683-01 | SPICH118-09 | GU683803 | 58.6180 | -93.8290 |
| *Xysticus luctuosus* | I | 09PROBE-1696-01 | SPICH141-09 | GU683800 | 58.7310 | -93.7800 |
| *Xysticus luctuosus* | A | 09PROBE-1798-01 | SPICH208-09 | GU683927 | 58.6300 | -93.8190 |
| *Xysticus luctuosus* | A | BIOUG00628-E07 | SPIRU1122-11 | KF369064 | 58.7574 | -93.9009 |
| *Xysticus luctuosus* | I | BIOUG00628-E08 | SPIRU1123-11 | KF369063 | 58.7574 | -93.9009 |
| *Xysticus nigromaculatus* | A | 07CHU-AR-0261 | KKCHE1068-09 | GU683744 | 58.6300 | -93.7980 |
| *Xysticus nigromaculatus* | A | 07PROBE-06522 | TWSC036-07 | KF369066 | 58.7370 | -93.8230 |
| *Xysticus nigromaculatus* | I | 09PROBE-1887-01 | SPICH053-09 | GU683895 | 58.7500 | -94.0840 |
| *Xysticus nigromaculatus* | I | JBWM0234271 | KKCHE286-07 | KF369065 | 58.7650 | -93.9840 |
| *Xysticus obscurus* | I | 07CHU-AR-0274 | KKCHE1081-09 | GU683596 | 58.6780 | -94.1460 |
| *Xysticus obscurus* | I | 09PROBE-1978-380 | SPICH623-09 | GU684175 | 58.6250 | -93.8160 |
| *Xysticus obscurus* | A | 09PROBE-788 | SPICH1009-09 | GU684734 | 58.6300 | -93.7980 |
| *Xysticus obscurus* | A | 09PROBE-944 | SPICH1165-09 | GU684592 | 58.7310 | -93.7800 |
| *Xysticus obscurus* | A | BIOUG00628-G02 | SPIRU1141-11 | KF369067 | 58.6350 | -93.7999 |
| *Xysticus triangulosus* | A | 07CHU-AR-0279 | KKCHE1086-09 | GU683600 | 58.6170 | -93.8120 |
| *Xysticus triangulosus* | I | 08BBARAC-0165 | ARSO289-08 | KF408104 | 51.1480 | -115.4200 |
| *Xysticus triangulosus* | A | 09PROBE-786 | SPICH1007-09 | GU684731 | 58.6300 | -93.7980 |
| *Xysticus triangulosus* | A | 09PROBE-787 | SPICH1008-09 | GU684732 | 58.6300 | -93.7980 |
| *Xysticus triangulosus* | A | BIOUG00628-F12 | SPIRU1139-11 | KF369069 | 58.6350 | -93.7999 |
| *Xysticus triangulosus* | A | BIOUG00629-C02 | SPIRU1188-11 | KF369068 | 58.6921 | -94.1328 |
| *Xysticus triangulosus* | I | YUKIN-0188 | JSYKA388-10 | HQ569153 | 69.1693 | -140.1590 |
| *Xysticus triangulosus* | I | YUKIN-0343 | JSYKA543-10 | HQ569240 | 69.1693 | -140.1590 |
| *Xysticus triangulosus* | I | YUKIN-0356 | JSYKA556-10 | HQ569251 | 69.1693 | -140.1590 |
| *Xysticus triguttatus* | A | 09PROBE-1689-01 | SPICH128-09 | GU683802 | 58.6180 | -93.8290 |
| *Zelotes fratris* | I | CHU-SPI-423 | KKCHE626-07 | KF369070 | 58.7380 | -93.8190 |
| *Zelotes sula* | I | 07CHU-AR-0230 | KKCHE1037-09 | GU683719 | 58.6260 | -94.2300 |
| *Zelotes sula* | A | 07PROBE-06504 | TWSC018-07 | KF369082 | 58.7640 | -94.0870 |
| *Zelotes sula* | A | 07PROBE-06505 | TWSC019-07 | KF369094 | 58.7570 | -93.9840 |
| *Zelotes sula* | A | 07PROBE-06507 | TWSC021-07 | KF369072 | 58.6300 | -93.7750 |
| *Zelotes sula* | A | 09PROBE-1679-01 | SPICH108-09 | GU683774 | 58.7610 | -93.9540 |
| *Zelotes sula* | I | 09PROBE-1710-01 | SPICH160-09 | GU683775 | 58.6610 | -93.8320 |
| *Zelotes sula* | A | 09PROBE-1801-01 | SPICH210-09 | GU683957 | 58.6300 | -93.8190 |
| *Zelotes sula* | A | 09PROBE-1801-02 | SPICH211-09 | GU683914 | 58.6300 | -93.8190 |
| *Zelotes sula* | A | 09PROBE-1875-01 | SPICH041-09 | GU683907 | 58.7500 | -94.0840 |
| *Zelotes sula* | A | 09PROBE-1876-01 | SPICH042-09 | GU683908 | 58.7500 | -94.0840 |
| *Zelotes sula* | A | 09PROBE-1877-01 | SPICH043-09 | GU683909 | 58.7500 | -94.0840 |
| *Zelotes sula* | I | 09PROBE-1912-01 | SPICH064-09 | GU683910 | 58.7630 | -93.8660 |
| *Zelotes sula* | A | 09PROBE-1978-478 | SPICH721-09 | GU684401 | 58.7640 | -93.8970 |
| *Zelotes sula* | A | 09PROBE-1978-496 | SPICH739-09 | GU684402 | 58.7890 | -93.7090 |
| *Zelotes sula* | A | 09PROBE-591 | SPICH834-09 | GU684406 | 58.7900 | -94.2270 |
| *Zelotes sula* | A | BIOUG00630-D07 | SPIRU1300-11 | KF369078 | 58.7707 | -94.1808 |
| *Zelotes sula* | A | BIOUG00630-E04 | SPIRU1309-11 | KF369087 | 58.7305 | -93.7805 |
| *Zelotes sula* | A | BIOUG00630-E05 | SPIRU1310-11 | KF369086 | 58.7305 | -93.7805 |
| *Zelotes sula* | A | BIOUG00630-E06 | SPIRU1311-11 | KF369083 | 58.7305 | -93.7805 |
| *Zelotes sula* | A | BIOUG00630-E10 | SPIRU1315-11 | KF369080 | 58.7720 | -93.8431 |
| *Zelotes sula* | A | BIOUG00630-G05 | SPIRU1334-11 | KF369077 | 58.7690 | -93.8620 |
| *Zelotes sula* | I | BIOUG00632-B02 | SPIRU1366-11 | KF369088 | 58.7710 | -93.8510 |
| *Zelotes sula* | A | BIOUG00632-B03 | SPIRU1367-11 | KF369089 | 58.7710 | -93.8510 |
| *Zelotes sula* | I | CHU-SPI-353 | KKCHE522-07 | KF369091 | 58.6320 | -93.7860 |
| *Zelotes sula* | I | CHU-SPI-399 | KKCHE602-07 | KF369075 | 58.7380 | -93.8190 |
| *Zelotes sula* | I | CHU-SPI-438 | KKCHE641-07 | KF369092 | 58.7380 | -93.8190 |
| *Zelotes sula* | I | CHU-SPI-447 | KKCHE650-07 | KF369073 | 58.7380 | -93.8190 |
| *Zelotes sula* | I | CHU-SPI-483 | KKCHE686-07 | KF369084 | 58.7380 | -93.8190 |
| *Zelotes sula* | I | CHU-SPI-536 | KKCHE739-07 | KF369076 | 58.7380 | -93.8190 |
| *Zelotes sula* | I | CHU-SPI-542 | KKCHE745-07 | KF369085 | 58.7470 | -94.1340 |
| *Zelotes sula* | I | CHU-SPI-543 | KKCHE746-07 | KF369071 | 58.7470 | -94.1340 |
| *Zelotes sula* | A | CHU-SPI-571 | KKCHE774-07 | KF369079 | 58.7380 | -93.8190 |
| *Zelotes sula* | I | CHU-SPI-601 | KKCHE804-07 | KF369074 | 58.7400 | -93.8200 |
| *Zelotes sula* | I | HLC-26867 | KKCHE898-09 | KF369081 | 58.7760 | -94.1880 |
| *Zelotes sula* | A | INV0276 | KKCHE082-06 | KF369090 | 58.6620 | -93.8350 |
| *Zelotes sula* | I | JBWM0006002f | KKCHE237-07 | KF369093 | 58.6290 | -93.7980 |
| *Zornella armata* | A | 09PROBE-1777-01 | SPICH199-09 | HM432627 | 58.6300 | -93.8190 |
| *Zornella armata* | I | BIOUG00632-A01 | SPIRU1353-11 | KF369095 | 58.6300 | -93.8190 |
| *Zygiella nearctica* | I | 07PROBE-04556 | ERSCH011-07 | KF369097 | 58.7310 | -93.7800 |
| *Zygiella nearctica* | I | 07PROBE-04559 | ERSCH014-07 | KF369101 | 58.7310 | -93.7800 |
| *Zygiella nearctica* | I | 07PROBE-04577 | ERSCH032-07 | KF369099 | 58.7310 | -93.7800 |
| *Zygiella nearctica* | I | 09PROBE-01585 | SWSWE015-09 | GU681069 | 58.6320 | -93.7860 |
| *Zygiella nearctica* | I | 09PROBE-01589 | SWSWE019-09 | GU681070 | 58.6320 | -93.7860 |
| *Zygiella nearctica* | I | 09PROBE-01592 | SWSWE022-09 | GU681064 | 58.6320 | -93.7860 |
| *Zygiella nearctica* | I | 09PROBE-01593 | SWSWE023-09 | GU681065 | 58.7300 | -93.7800 |
| *Zygiella nearctica* | I | 09PROBE-01595 | SWSWE025-09 | GU681063 | 58.7300 | -93.7800 |
| *Zygiella nearctica* | I | 09PROBE-01596 | SWSWE026-09 | GU681060 | 58.7300 | -93.7800 |
| *Zygiella nearctica* | I | 09PROBE-01601 | SWSWE031-09 | GU681059 | 58.7300 | -93.7800 |
| *Zygiella nearctica* | I | 09PROBE-01602 | SWSWE032-09 | GU681057 | 58.7300 | -93.7800 |
| *Zygiella nearctica* | I | 09PROBE-01625 | SWSWE055-09 | GU681040 | 58.6320 | -93.7860 |
| *Zygiella nearctica* | I | 09PROBE-01626 | SWSWE056-09 | GU681041 | 58.6320 | -93.7860 |
| *Zygiella nearctica* | I | 09PROBE-01628 | SWSWE058-09 | GU681039 | 58.6320 | -93.7860 |
| *Zygiella nearctica* | I | 09PROBE-01630 | SWSWE060-09 | GU681037 | 58.6320 | -93.7860 |
| *Zygiella nearctica* | I | 09PROBE-01631 | SWSWE061-09 | GU681018 | 58.6320 | -93.7860 |
| *Zygiella nearctica* | I | 09PROBE-01633 | SWSWE063-09 | GU681035 | 58.6320 | -93.7860 |
| *Zygiella nearctica* | I | 09PROBE-01634 | SWSWE064-09 | GU681032 | 58.6320 | -93.7860 |
| *Zygiella nearctica* | I | 09PROBE-01638 | SWSWE068-09 | GU681031 | 58.6320 | -93.7860 |
| *Zygiella nearctica* | I | 09PROBE-01639 | SWSWE069-09 | GU681028 | 58.6320 | -93.7860 |
| *Zygiella nearctica* | I | 09PROBE-01640 | SWSWE070-09 | GU681029 | 58.6320 | -93.7860 |
| *Zygiella nearctica* | I | 09PROBE-01641 | SWSWE071-09 | GU681026 | 58.6320 | -93.7860 |
| *Zygiella nearctica* | I | 09PROBE-01642 | SWSWE072-09 | GU681027 | 58.6320 | -93.7860 |
| *Zygiella nearctica* | I | 09-PROBE-08191 | JDTGS095-09 | GU679829 | 58.6300 | -93.8190 |
| *Zygiella nearctica* | A | 09PROBE-1687-01 | SPICH123-09 | GU684001 | 58.7310 | -93.7800 |
| *Zygiella nearctica* | I | 09PROBE-1700-01 | SPICH146-09 | GU683823 | 58.7300 | -93.7800 |
| *Zygiella nearctica* | A | 09PROBE-764 | SPISH046-09 | GU684739 | 58.7310 | -93.7800 |
| *Zygiella nearctica* | A | 09PROBE-905 | SPICH1126-09 | GU684584 | 58.6300 | -93.7980 |
| *Zygiella nearctica* | A | 09PROBE-909 | SPICH1130-09 | GU684585 | 58.6300 | -93.7980 |
| *Zygiella nearctica* | A | 09PROBE-912 | SPICH1133-09 | GU684586 | 58.6300 | -93.7980 |
| *Zygiella nearctica* | A | BIOUG00629-A05 | SPIRU1167-11 | KF369100 | 58.7305 | -93.7804 |
| *Zygiella nearctica* | A | BIOUG00629-A11 | SPIRU1173-11 | KF369102 | 58.7306 | -93.7804 |
| *Zygiella nearctica* | A | BIOUG00629-A12 | SPIRU1174-11 | KF369096 | 58.7306 | -93.7804 |
| *Zygiella nearctica* | A | BIOUG00629-B02 | SPIRU1176-11 | KF369098 | 58.7306 | -93.7804 |
