# Supplementary material for: Spiders (Araneae) of Churchill, Manitoba: DNA barcodes and morphology reveal high species diversity and new Canadian records
Source: BMC Ecol. 2013 Nov 26;13:44. doi: 10.1186/1472-6785-13-44 (PMC4222278; doi:10.1186/1472-6785-13-44)
Supplement: Additional file 3 — Ultrametricized neighbour-joining tree (K2P) for all 2704 COI sequences >500 bp from spiders collected at Churchill. Red branches indicate the cryptic species, and blue colouring is used to highlight the new species records for Canada. [file 1472-6785-13-44-S3.pdf]

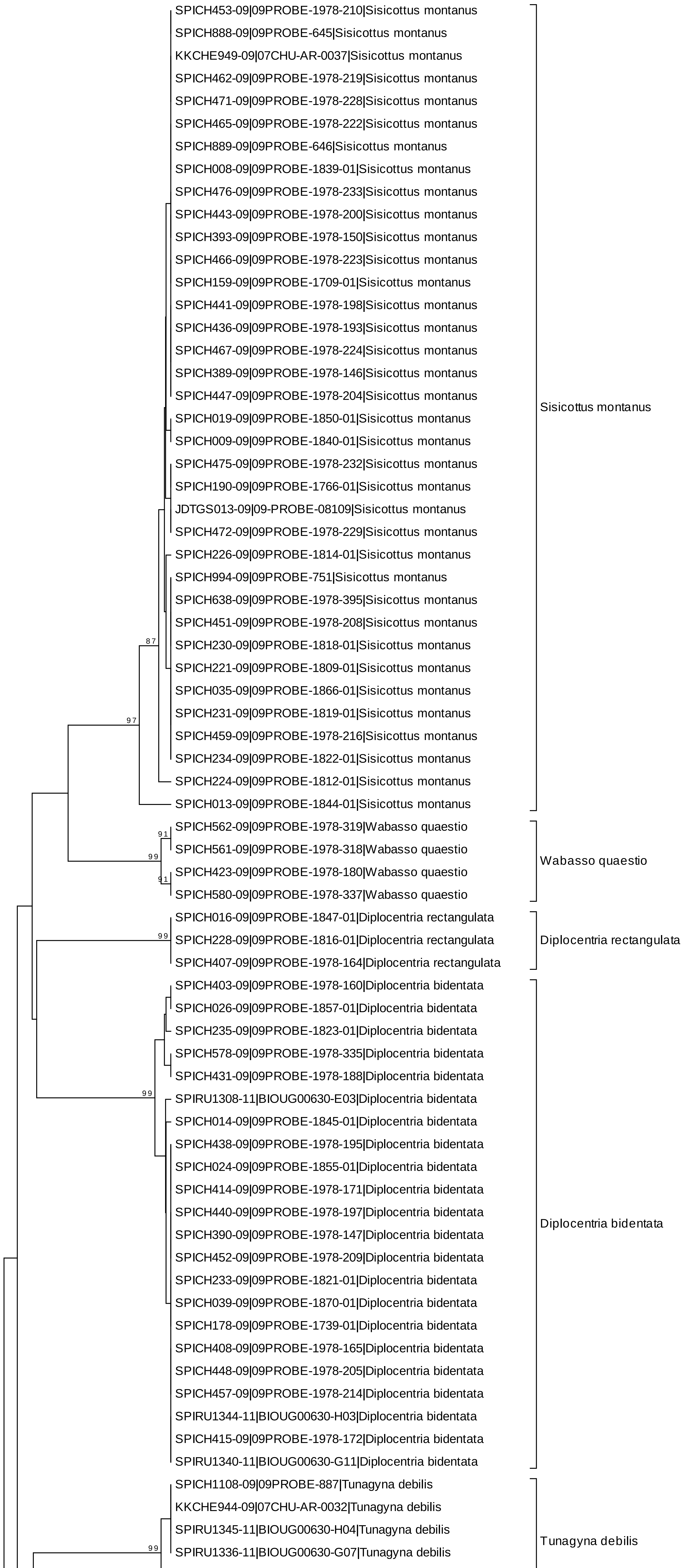
SPICH453-09|09PROBE-1978-210|Sisicottus montanus
SPICH888-09|09PROBE-645|Sisicottus montanus
KKCHE949-09|07CHU-AR-0037|Sisicottus montanus
SPICH462-09|09PROBE-1978-219|Sisicottus montanus
SPICH471-09|09PROBE-1978-228|Sisicottus montanus
SPICH465-09|09PROBE-1978-222|Sisicottus montanus
SPICH889-09|09PROBE-646|Sisicottus montanus
SPICH008-09|09PROBE-1839-01|Sisicottus montanus
SPICH476-09|09PROBE-1978-233|Sisicottus montanus
SPICH443-09|09PROBE-1978-200|Sisicottus montanus
SPICH393-09|09PROBE-1978-150|Sisicottus montanus
SPICH466-09|09PROBE-1978-223|Sisicottus montanus
SPICH159-09|09PROBE-1709-01|Sisicottus montanus
SPICH441-09|09PROBE-1978-198|Sisicottus montanus
SPICH436-09|09PROBE-1978-193|Sisicottus montanus
SPICH467-09|09PROBE-1978-224|Sisicottus montanus
SPICH389-09|09PROBE-1978-146|Sisicottus montanus
SPICH447-09|09PROBE-1978-204|Sisicottus montanus
SPICH019-09|09PROBE-1850-01|Sisicottus montanus
SPICH009-09|09PROBE-1840-01|Sisicottus montanus
SPICH475-09|09PROBE-1978-232|Sisicottus montanus
SPICH190-09|09PROBE-1766-01|Sisicottus montanus
JDTGS013-09|09-PROBE-08109|Sisicottus montanus
SPICH472-09|09PROBE-1978-229|Sisicottus montanus
SPICH226-09|09PROBE-1814-01|Sisicottus montanus
SPICH994-09|09PROBE-751|Sisicottus montanus
SPICH638-09|09PROBE-1978-395|Sisicottus montanus
SPICH451-09|09PROBE-1978-208|Sisicottus montanus
87
SPICH230-09|09PROBE-1818-01|Sisicottus montanus
SPICH221-09|09PROBE-1809-01|Sisicottus montanus
SPICH035-09|09PROBE-1866-01|Sisicottus montanus
97
SPICH231-09|09PROBE-1819-01|Sisicottus montanus
SPICH459-09|09PROBE-1978-216|Sisicottus montanus
SPICH234-09|09PROBE-1822-01|Sisicottus montanus
SPICH224-09|09PROBE-1812-01|Sisicottus montanus
SPICH013-09|09PROBE-1844-01|Sisicottus montanus
Sisicottus montanus
91
SPICH562-09|09PROBE-1978-319|Wabasso quaestio
99
SPICH561-09|09PROBE-1978-318|Wabasso quaestio
SPICH423-09|09PROBE-1978-180|Wabasso quaestio
91
SPICH580-09|09PROBE-1978-337|Wabasso quaestio
Wabasso quaestio
SPICH016-09|09PROBE-1847-01|Diplocentria rectangulata
99
SPICH228-09|09PROBE-1816-01|Diplocentria rectangulata
SPICH407-09|09PROBE-1978-164|Diplocentria rectangulata
Diplocentria rectangulata
SPICH403-09|09PROBE-1978-160|Diplocentria bidentata
SPICH026-09|09PROBE-1857-01|Diplocentria bidentata
SPICH235-09|09PROBE-1823-01|Diplocentria bidentata
SPICH578-09|09PROBE-1978-335|Diplocentria bidentata
SPICH431-09|09PROBE-1978-188|Diplocentria bidentata
99
SPIRU1308-11|BIOUG00630-E03|Diplocentria bidentata
SPICH014-09|09PROBE-1845-01|Diplocentria bidentata
SPICH438-09|09PROBE-1978-195|Diplocentria bidentata
SPICH024-09|09PROBE-1855-01|Diplocentria bidentata
SPICH414-09|09PROBE-1978-171|Diplocentria bidentata
SPICH440-09|09PROBE-1978-197|Diplocentria bidentata
SPICH390-09|09PROBE-1978-147|Diplocentria bidentata
SPICH452-09|09PROBE-1978-209|Diplocentria bidentata
SPICH233-09|09PROBE-1821-01|Diplocentria bidentata
SPICH039-09|09PROBE-1870-01|Diplocentria bidentata
SPICH178-09|09PROBE-1739-01|Diplocentria bidentata
SPICH408-09|09PROBE-1978-165|Diplocentria bidentata
SPICH448-09|09PROBE-1978-205|Diplocentria bidentata
SPICH457-09|09PROBE-1978-214|Diplocentria bidentata
SPIRU1344-11|BIOUG00630-H03|Diplocentria bidentata
SPICH415-09|09PROBE-1978-172|Diplocentria bidentata
SPIRU1340-11|BIOUG00630-G11|Diplocentria bidentata
Diplocentria bidentata
SPICH1108-09|09PROBE-887|Tunagyna debilis
KKCHE944-09|07CHU-AR-0032|Tunagyna debilis
SPIRU1345-11|BIOUG00630-H04|Tunagyna debilis
99
SPIRU1336-11|BIOUG00630-G07|Tunagyna debilis
Tunagyna debilis

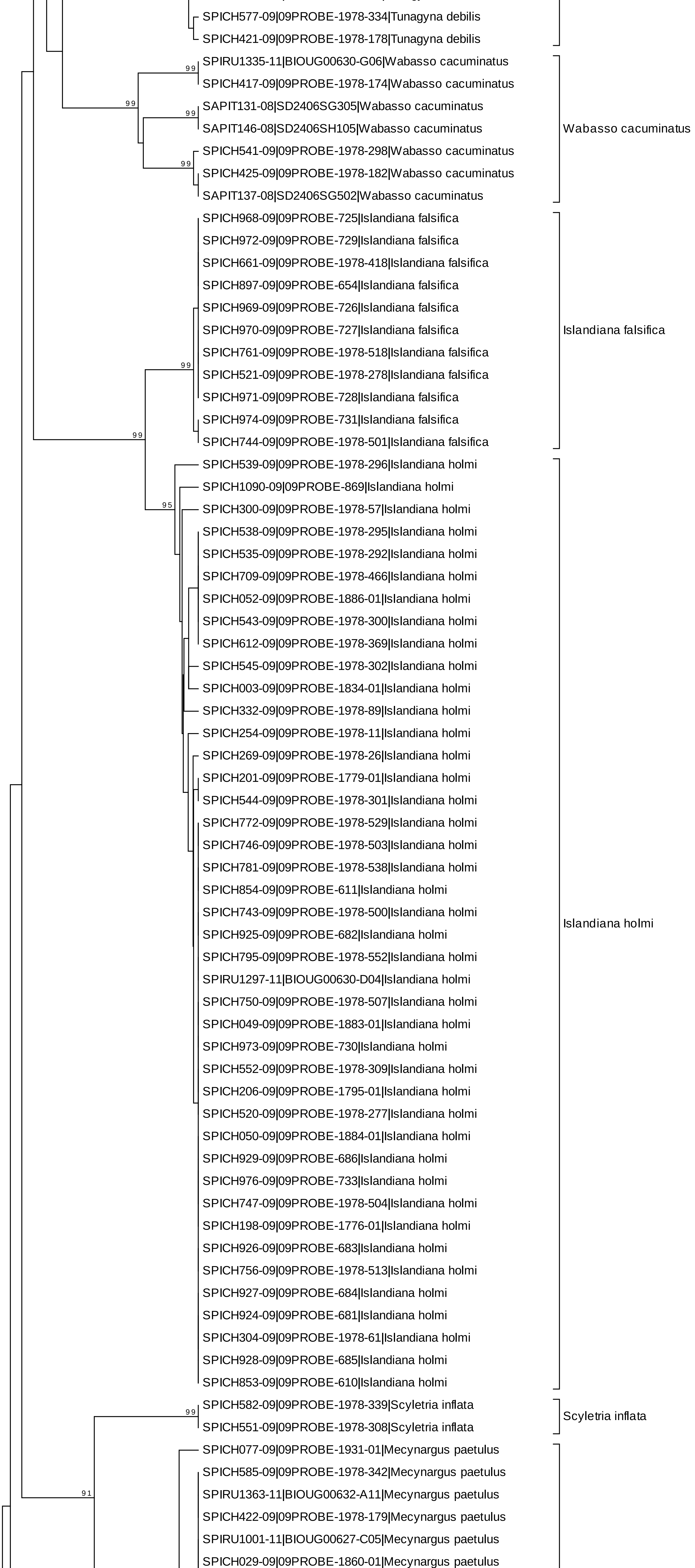
SPICH577-09|09PROBE-1978-334|Tunagyna debilis
SPICH421-09|09PROBE-1978-178|Tunagyna debilis
99
SPIRU1335-11|BIOUG00630-G06|Wabasso cacuminatus
SPICH417-09|09PROBE-1978-174|Wabasso cacuminatus
99
99
SAPIT131-08|SD2406SG305|Wabasso cacuminatus
SAPIT146-08|SD2406SH105|Wabasso cacuminatus
99
SPICH541-09|09PROBE-1978-298|Wabasso cacuminatus
SPICH425-09|09PROBE-1978-182|Wabasso cacuminatus
SAPIT137-08|SD2406SG502|Wabasso cacuminatus
Wabasso cacuminatus
SPICH968-09|09PROBE-725|Islandiana falsifica
SPICH972-09|09PROBE-729|Islandiana falsifica
SPICH661-09|09PROBE-1978-418|Islandiana falsifica
SPICH897-09|09PROBE-654|Islandiana falsifica
SPICH969-09|09PROBE-726|Islandiana falsifica
SPICH970-09|09PROBE-727|Islandiana falsifica
SPICH761-09|09PROBE-1978-518|Islandiana falsifica
99
SPICH521-09|09PROBE-1978-278|Islandiana falsifica
SPICH971-09|09PROBE-728|Islandiana falsifica
SPICH974-09|09PROBE-731|Islandiana falsifica
99
SPICH744-09|09PROBE-1978-501|Islandiana falsifica
Islandiana falsifica
SPICH539-09|09PROBE-1978-296|Islandiana holmi
SPICH1090-09|09PROBE-869|Islandiana holmi
95
SPICH300-09|09PROBE-1978-57|Islandiana holmi
SPICH538-09|09PROBE-1978-295|Islandiana holmi
SPICH535-09|09PROBE-1978-292|Islandiana holmi
SPICH709-09|09PROBE-1978-466|Islandiana holmi
SPICH052-09|09PROBE-1886-01|Islandiana holmi
SPICH543-09|09PROBE-1978-300|Islandiana holmi
SPICH612-09|09PROBE-1978-369|Islandiana holmi
SPICH545-09|09PROBE-1978-302|Islandiana holmi
SPICH003-09|09PROBE-1834-01|Islandiana holmi
SPICH332-09|09PROBE-1978-89|Islandiana holmi
SPICH254-09|09PROBE-1978-11|Islandiana holmi
SPICH269-09|09PROBE-1978-26|Islandiana holmi
SPICH201-09|09PROBE-1779-01|Islandiana holmi
SPICH544-09|09PROBE-1978-301|Islandiana holmi
SPICH772-09|09PROBE-1978-529|Islandiana holmi
SPICH746-09|09PROBE-1978-503|Islandiana holmi
SPICH781-09|09PROBE-1978-538|Islandiana holmi
SPICH854-09|09PROBE-611|Islandiana holmi
SPICH743-09|09PROBE-1978-500|Islandiana holmi
SPICH925-09|09PROBE-682|Islandiana holmi
SPICH795-09|09PROBE-1978-552|Islandiana holmi
SPIRU1297-11|BIOUG00630-D04|Islandiana holmi
SPICH750-09|09PROBE-1978-507|Islandiana holmi
SPICH049-09|09PROBE-1883-01|Islandiana holmi
SPICH973-09|09PROBE-730|Islandiana holmi
SPICH552-09|09PROBE-1978-309|Islandiana holmi
SPICH206-09|09PROBE-1795-01|Islandiana holmi
SPICH520-09|09PROBE-1978-277|Islandiana holmi
SPICH050-09|09PROBE-1884-01|Islandiana holmi
SPICH929-09|09PROBE-686|Islandiana holmi
SPICH976-09|09PROBE-733|Islandiana holmi
SPICH747-09|09PROBE-1978-504|Islandiana holmi
SPICH198-09|09PROBE-1776-01|Islandiana holmi
SPICH926-09|09PROBE-683|Islandiana holmi
SPICH756-09|09PROBE-1978-513|Islandiana holmi
SPICH927-09|09PROBE-684|Islandiana holmi
SPICH924-09|09PROBE-681|Islandiana holmi
SPICH304-09|09PROBE-1978-61|Islandiana holmi
SPICH928-09|09PROBE-685|Islandiana holmi
SPICH853-09|09PROBE-610|Islandiana holmi
Islandiana holmi
99
SPICH582-09|09PROBE-1978-339|Scyletria inflata
SPICH551-09|09PROBE-1978-308|Scyletria inflata
Scyletria inflata
SPICH077-09|09PROBE-1931-01|Mecynargus paetulus
SPICH585-09|09PROBE-1978-342|Mecynargus paetulus
91
SPIRU1363-11|BIOUG00632-A11|Mecynargus paetulus
SPICH422-09|09PROBE-1978-179|Mecynargus paetulus
SPIRU1001-11|BIOUG00627-C05|Mecynargus paetulus
SPICH029-09|09PROBE-1860-01|Mecynargus paetulus

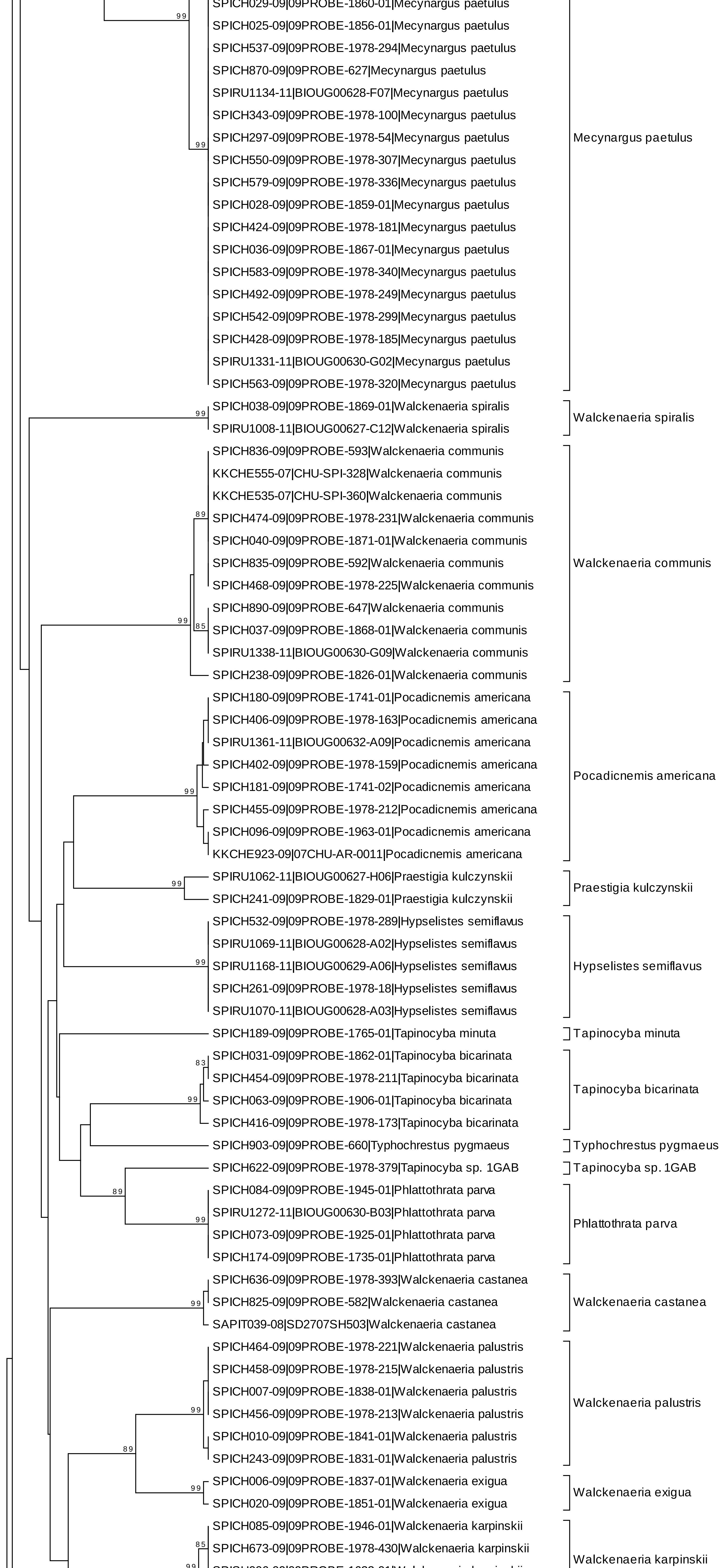
SPICH029-09|09PROBE-1860-01|Mecynargus paetulus
SPICH025-09|09PROBE-1856-01|Mecynargus paetulus
SPICH537-09|09PROBE-1978-294|Mecynargus paetulus
SPICH870-09|09PROBE-627|Mecynargus paetulus
SPIRU1134-11|BIOUG00628-F07|Mecynargus paetulus
SPICH343-09|09PROBE-1978-100|Mecynargus paetulus
SPICH297-09|09PROBE-1978-54|Mecynargus paetulus
SPICH550-09|09PROBE-1978-307|Mecynargus paetulus
SPICH579-09|09PROBE-1978-336|Mecynargus paetulus
SPICH028-09|09PROBE-1859-01|Mecynargus paetulus
SPICH424-09|09PROBE-1978-181|Mecynargus paetulus
SPICH036-09|09PROBE-1867-01|Mecynargus paetulus
SPICH583-09|09PROBE-1978-340|Mecynargus paetulus
SPICH492-09|09PROBE-1978-249|Mecynargus paetulus
SPICH542-09|09PROBE-1978-299|Mecynargus paetulus
SPICH428-09|09PROBE-1978-185|Mecynargus paetulus
SPIRU1331-11|BIOUG00630-G02|Mecynargus paetulus
SPICH563-09|09PROBE-1978-320|Mecynargus paetulus
Mecynargus paetulus
SPICH038-09|09PROBE-1869-01|Walckenaeria spiralis
SPIRU1008-11|BIOUG00627-C12|Walckenaeria spiralis
Walckenaeria spiralis
SPICH836-09|09PROBE-593|Walckenaeria communis
KKCHE555-07|CHU-SPI-328|Walckenaeria communis
KKCHE535-07|CHU-SPI-360|Walckenaeria communis
SPICH474-09|09PROBE-1978-231|Walckenaeria communis
SPICH040-09|09PROBE-1871-01|Walckenaeria communis
SPICH835-09|09PROBE-592|Walckenaeria communis
SPICH468-09|09PROBE-1978-225|Walckenaeria communis
SPICH890-09|09PROBE-647|Walckenaeria communis
SPICH037-09|09PROBE-1868-01|Walckenaeria communis
SPIRU1338-11|BIOUG00630-G09|Walckenaeria communis
SPICH238-09|09PROBE-1826-01|Walckenaeria communis
Walckenaeria communis
SPICH180-09|09PROBE-1741-01|Pocadicnemis americana
SPICH406-09|09PROBE-1978-163|Pocadicnemis americana
SPIRU1361-11|BIOUG00632-A09|Pocadicnemis americana
SPICH402-09|09PROBE-1978-159|Pocadicnemis americana
SPICH181-09|09PROBE-1741-02|Pocadicnemis americana
SPICH455-09|09PROBE-1978-212|Pocadicnemis americana
SPICH096-09|09PROBE-1963-01|Pocadicnemis americana
KKCHE923-09|07CHU-AR-0011|Pocadicnemis americana
Pocadicnemis americana
SPIRU1062-11|BIOUG00627-H06|Praestigia kulczynskii
SPICH241-09|09PROBE-1829-01|Praestigia kulczynskii
Praestigia kulczynskii
SPICH532-09|09PROBE-1978-289|Hypselistes semiflavus
SPIRU1069-11|BIOUG00628-A02|Hypselistes semiflavus
SPIRU1168-11|BIOUG00629-A06|Hypselistes semiflavus
SPICH261-09|09PROBE-1978-18|Hypselistes semiflavus
SPIRU1070-11|BIOUG00628-A03|Hypselistes semiflavus
Hypselistes semiflavus
SPICH189-09|09PROBE-1765-01|Tapinocyba minuta
Tapinocyba minuta
SPICH031-09|09PROBE-1862-01|Tapinocyba bicarinata
SPICH454-09|09PROBE-1978-211|Tapinocyba bicarinata
SPICH063-09|09PROBE-1906-01|Tapinocyba bicarinata
SPICH416-09|09PROBE-1978-173|Tapinocyba bicarinata
Tapinocyba bicarinata
SPICH903-09|09PROBE-660|Typhochrestus pygmaeus
Typhochrestus pygmaeus
SPICH622-09|09PROBE-1978-379|Tapinocyba sp. 1GAB
Tapinocyba sp. 1GAB
SPICH084-09|09PROBE-1945-01|Phlattothrata parva
SPIRU1272-11|BIOUG00630-B03|Phlattothrata parva
SPICH073-09|09PROBE-1925-01|Phlattothrata parva
SPICH174-09|09PROBE-1735-01|Phlattothrata parva
Phlattothrata parva
SPICH636-09|09PROBE-1978-393|Walckenaeria castanea
SPICH825-09|09PROBE-582|Walckenaeria castanea
SAPIT039-08|SD2707SH503|Walckenaeria castanea
Walckenaeria castanea
SPICH464-09|09PROBE-1978-221|Walckenaeria palustris
SPICH458-09|09PROBE-1978-215|Walckenaeria palustris
SPICH007-09|09PROBE-1838-01|Walckenaeria palustris
SPICH456-09|09PROBE-1978-213|Walckenaeria palustris
SPICH010-09|09PROBE-1841-01|Walckenaeria palustris
SPICH243-09|09PROBE-1831-01|Walckenaeria palustris
Walckenaeria palustris
SPICH006-09|09PROBE-1837-01|Walckenaeria exigua
SPICH020-09|09PROBE-1851-01|Walckenaeria exigua
Walckenaeria exigua
SPICH085-09|09PROBE-1946-01|Walckenaeria karpinskii
SPICH673-09|09PROBE-1978-430|Walckenaeria karpinskii
Walckenaeria karpinskii

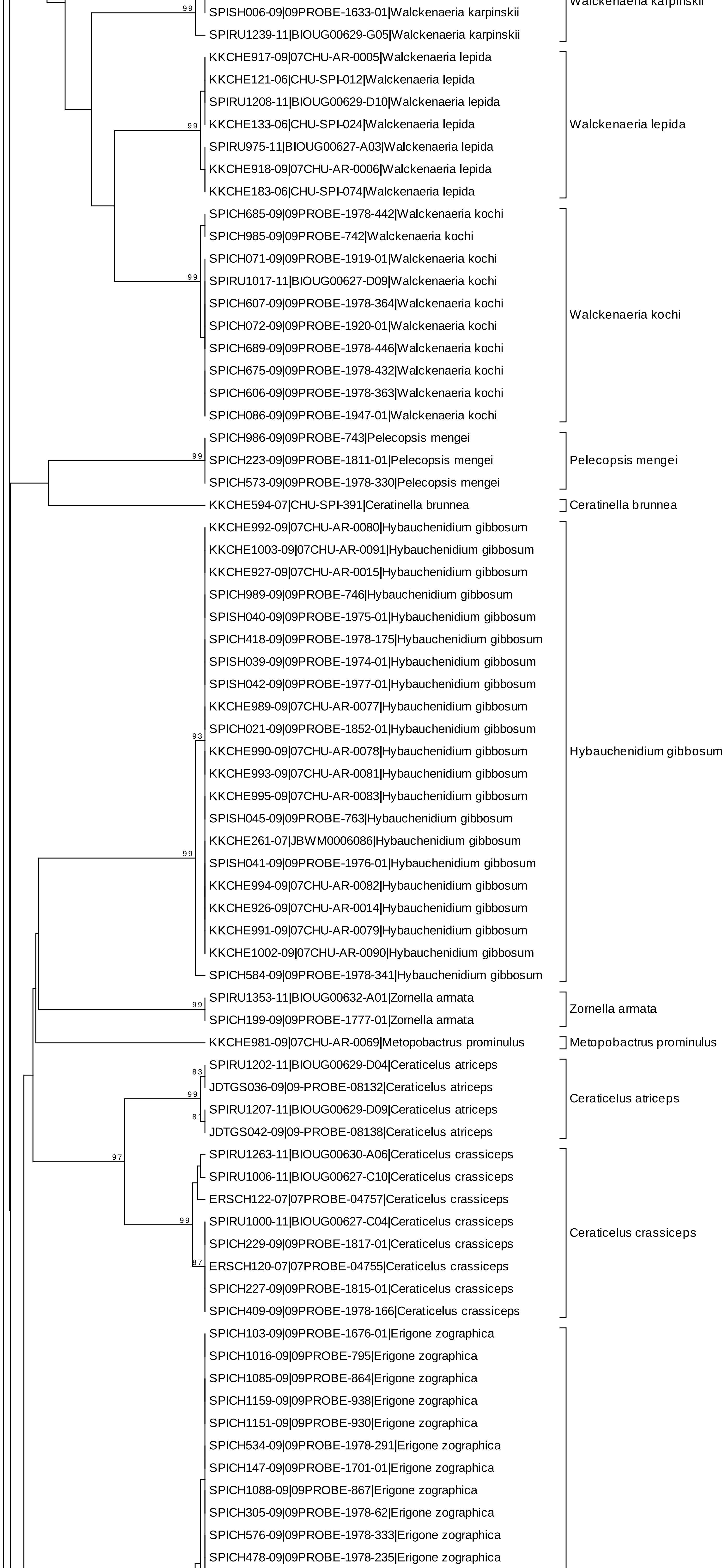

SPISH006-09|09PROBE-1633-01|Walckenaeria karpinskii
SPIRU1239-11|BIOUG00629-G05|Walckenaeria karpinskii
Walckenaeria karpinskii
KKCHE917-09|07CHU-AR-0005|Walckenaeria lepida
KKCHE121-06|CHU-SPI-012|Walckenaeria lepida
SPIRU1208-11|BIOUG00629-D10|Walckenaeria lepida
KKCHE133-06|CHU-SPI-024|Walckenaeria lepida
SPIRU975-11|BIOUG00627-A03|Walckenaeria lepida
KKCHE918-09|07CHU-AR-0006|Walckenaeria lepida
KKCHE183-06|CHU-SPI-074|Walckenaeria lepida
Walckenaeria lepida
SPICH685-09|09PROBE-1978-442|Walckenaeria kochi
SPICH985-09|09PROBE-742|Walckenaeria kochi
SPICH071-09|09PROBE-1919-01|Walckenaeria kochi
SPIRU1017-11|BIOUG00627-D09|Walckenaeria kochi
SPICH607-09|09PROBE-1978-364|Walckenaeria kochi
SPICH072-09|09PROBE-1920-01|Walckenaeria kochi
SPICH689-09|09PROBE-1978-446|Walckenaeria kochi
SPICH675-09|09PROBE-1978-432|Walckenaeria kochi
SPICH606-09|09PROBE-1978-363|Walckenaeria kochi
SPICH086-09|09PROBE-1947-01|Walckenaeria kochi
Walckenaeria kochi
SPICH986-09|09PROBE-743|Pelecopsis mengei
SPICH223-09|09PROBE-1811-01|Pelecopsis mengei
SPICH573-09|09PROBE-1978-330|Pelecopsis mengei
Pelecopsis mengei
KKCHE594-07|CHU-SPI-391|Ceratinella brunnea
Ceratinella brunnea
KKCHE992-09|07CHU-AR-0080|Hybauchenidium gibbosum
KKCHE1003-09|07CHU-AR-0091|Hybauchenidium gibbosum
KKCHE927-09|07CHU-AR-0015|Hybauchenidium gibbosum
SPICH989-09|09PROBE-746|Hybauchenidium gibbosum
SPISH040-09|09PROBE-1975-01|Hybauchenidium gibbosum
SPICH418-09|09PROBE-1978-175|Hybauchenidium gibbosum
SPISH039-09|09PROBE-1974-01|Hybauchenidium gibbosum
SPISH042-09|09PROBE-1977-01|Hybauchenidium gibbosum
KKCHE989-09|07CHU-AR-0077|Hybauchenidium gibbosum
SPICH021-09|09PROBE-1852-01|Hybauchenidium gibbosum
KKCHE990-09|07CHU-AR-0078|Hybauchenidium gibbosum
KKCHE993-09|07CHU-AR-0081|Hybauchenidium gibbosum
KKCHE995-09|07CHU-AR-0083|Hybauchenidium gibbosum
SPISH045-09|09PROBE-763|Hybauchenidium gibbosum
KKCHE261-07|JBWM0006086|Hybauchenidium gibbosum
SPISH041-09|09PROBE-1976-01|Hybauchenidium gibbosum
KKCHE994-09|07CHU-AR-0082|Hybauchenidium gibbosum
KKCHE926-09|07CHU-AR-0014|Hybauchenidium gibbosum
KKCHE991-09|07CHU-AR-0079|Hybauchenidium gibbosum
KKCHE1002-09|07CHU-AR-0090|Hybauchenidium gibbosum
SPICH584-09|09PROBE-1978-341|Hybauchenidium gibbosum
Hybauchenidium gibbosum
SPIRU1353-11|BIOUG00632-A01|Zornella armata
SPICH199-09|09PROBE-1777-01|Zornella armata
Zornella armata
KKCHE981-09|07CHU-AR-0069|Metopobactrus prominulus
Metopobactrus prominulus
SPIRU1202-11|BIOUG00629-D04|Ceraticelus atriceps
JDTGS036-09|09-PROBE-08132|Ceraticelus atriceps
SPIRU1207-11|BIOUG00629-D09|Ceraticelus atriceps
JDTGS042-09|09-PROBE-08138|Ceraticelus atriceps
Ceraticelus atriceps
SPIRU1263-11|BIOUG00630-A06|Ceraticelus crassiceps
SPIRU1006-11|BIOUG00627-C10|Ceraticelus crassiceps
ERSCH122-07|07PROBE-04757|Ceraticelus crassiceps
SPIRU1000-11|BIOUG00627-C04|Ceraticelus crassiceps
SPICH229-09|09PROBE-1817-01|Ceraticelus crassiceps
ERSCH120-07|07PROBE-04755|Ceraticelus crassiceps
SPICH227-09|09PROBE-1815-01|Ceraticelus crassiceps
SPICH409-09|09PROBE-1978-166|Ceraticelus crassiceps
Ceraticelus crassiceps
SPICH103-09|09PROBE-1676-01|Erigone zographica
SPICH1016-09|09PROBE-795|Erigone zographica
SPICH1085-09|09PROBE-864|Erigone zographica
SPICH1159-09|09PROBE-938|Erigone zographica
SPICH1151-09|09PROBE-930|Erigone zographica
SPICH534-09|09PROBE-1978-291|Erigone zographica
SPICH147-09|09PROBE-1701-01|Erigone zographica
SPICH1088-09|09PROBE-867|Erigone zographica
SPICH305-09|09PROBE-1978-62|Erigone zographica
SPICH576-09|09PROBE-1978-333|Erigone zographica
SPICH478-09|09PROBE-1978-235|Erigone zographica
99
99
99
99
93
99
99
83
99
81
97
99
87

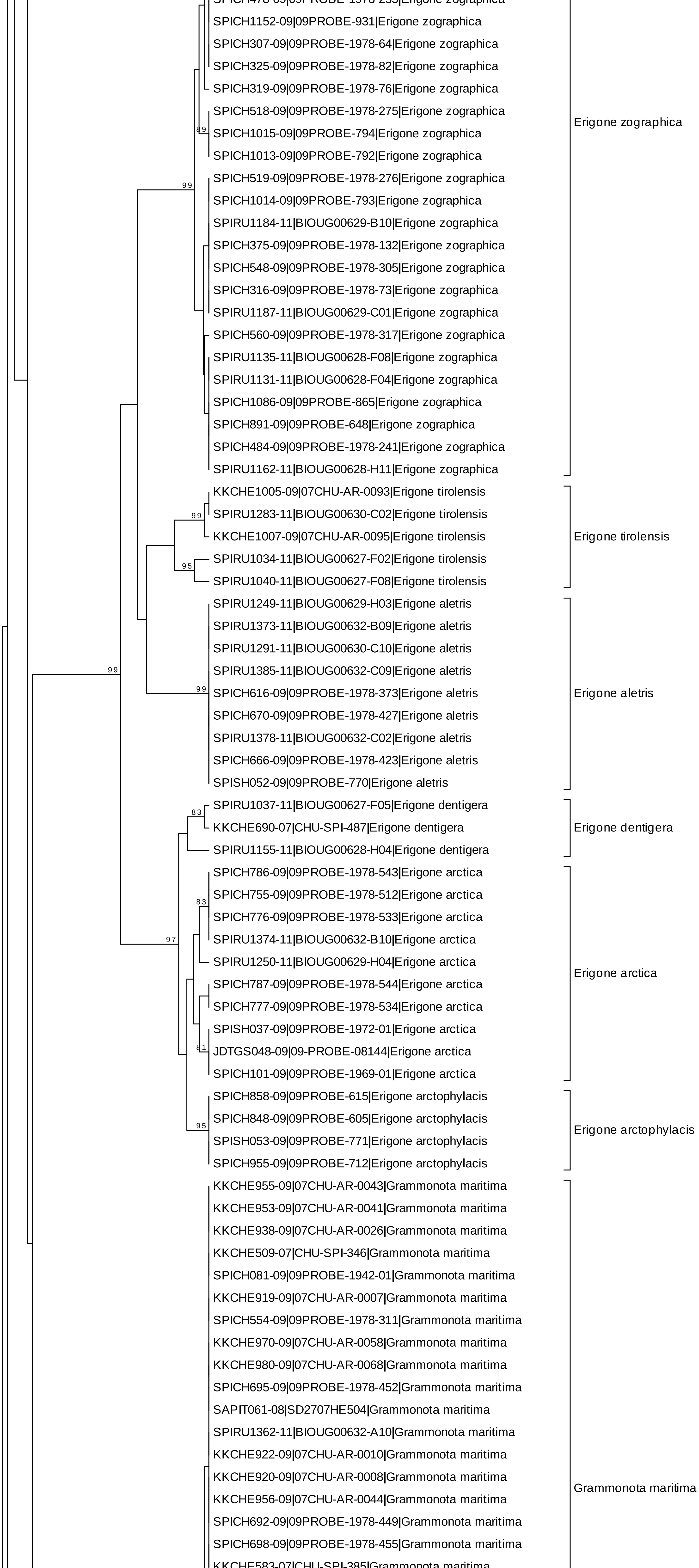
SPICH1152-09|09PROBE-931|Erigone zographica
SPICH307-09|09PROBE-1978-64|Erigone zographica
SPICH325-09|09PROBE-1978-82|Erigone zographica
SPICH319-09|09PROBE-1978-76|Erigone zographica
SPICH518-09|09PROBE-1978-275|Erigone zographica
89
SPICH1015-09|09PROBE-794|Erigone zographica
SPICH1013-09|09PROBE-792|Erigone zographica
99
SPICH519-09|09PROBE-1978-276|Erigone zographica
SPICH1014-09|09PROBE-793|Erigone zographica
SPIRU1184-11|BIOUG00629-B10|Erigone zographica
SPICH375-09|09PROBE-1978-132|Erigone zographica
SPICH548-09|09PROBE-1978-305|Erigone zographica
SPICH316-09|09PROBE-1978-73|Erigone zographica
SPIRU1187-11|BIOUG00629-C01|Erigone zographica
SPICH560-09|09PROBE-1978-317|Erigone zographica
SPIRU1135-11|BIOUG00628-F08|Erigone zographica
SPIRU1131-11|BIOUG00628-F04|Erigone zographica
SPICH1086-09|09PROBE-865|Erigone zographica
SPICH891-09|09PROBE-648|Erigone zographica
SPICH484-09|09PROBE-1978-241|Erigone zographica
SPIRU1162-11|BIOUG00628-H11|Erigone zographica
Erigone zographica
KKCHE1005-09|07CHU-AR-0093|Erigone tirolensis
99
SPIRU1283-11|BIOUG00630-C02|Erigone tirolensis
KKCHE1007-09|07CHU-AR-0095|Erigone tirolensis
95
SPIRU1034-11|BIOUG00627-F02|Erigone tirolensis
SPIRU1040-11|BIOUG00627-F08|Erigone tirolensis
Erigone tirolensis
SPIRU1249-11|BIOUG00629-H03|Erigone aletris
SPIRU1373-11|BIOUG00632-B09|Erigone aletris
SPIRU1291-11|BIOUG00630-C10|Erigone aletris
99
SPIRU1385-11|BIOUG00632-C09|Erigone aletris
99
SPICH616-09|09PROBE-1978-373|Erigone aletris
SPICH670-09|09PROBE-1978-427|Erigone aletris
SPIRU1378-11|BIOUG00632-C02|Erigone aletris
SPICH666-09|09PROBE-1978-423|Erigone aletris
SPISH052-09|09PROBE-770|Erigone aletris
Erigone aletris
83
SPIRU1037-11|BIOUG00627-F05|Erigone dentigera
KKCHE690-07|CHU-SPI-487|Erigone dentigera
SPIRU1155-11|BIOUG00628-H04|Erigone dentigera
Erigone dentigera
SPICH786-09|09PROBE-1978-543|Erigone arctica
SPICH755-09|09PROBE-1978-512|Erigone arctica
83
SPICH776-09|09PROBE-1978-533|Erigone arctica
97
SPIRU1374-11|BIOUG00632-B10|Erigone arctica
SPIRU1250-11|BIOUG00629-H04|Erigone arctica
SPICH787-09|09PROBE-1978-544|Erigone arctica
SPICH777-09|09PROBE-1978-534|Erigone arctica
SPISH037-09|09PROBE-1972-01|Erigone arctica
81
JDTGS048-09|09-PROBE-08144|Erigone arctica
SPICH101-09|09PROBE-1969-01|Erigone arctica
Erigone arctica
SPICH858-09|09PROBE-615|Erigone arctophylacis
95
SPICH848-09|09PROBE-605|Erigone arctophylacis
SPISH053-09|09PROBE-771|Erigone arctophylacis
SPICH955-09|09PROBE-712|Erigone arctophylacis
Erigone arctophylacis
KKCHE955-09|07CHU-AR-0043|Grammonota maritima
KKCHE953-09|07CHU-AR-0041|Grammonota maritima
KKCHE938-09|07CHU-AR-0026|Grammonota maritima
KKCHE509-07|CHU-SPI-346|Grammonota maritima
SPICH081-09|09PROBE-1942-01|Grammonota maritima
KKCHE919-09|07CHU-AR-0007|Grammonota maritima
SPICH554-09|09PROBE-1978-311|Grammonota maritima
KKCHE970-09|07CHU-AR-0058|Grammonota maritima
KKCHE980-09|07CHU-AR-0068|Grammonota maritima
SPICH695-09|09PROBE-1978-452|Grammonota maritima
SAPIT061-08|SD2707HE504|Grammonota maritima
SPIRU1362-11|BIOUG00632-A10|Grammonota maritima
KKCHE922-09|07CHU-AR-0010|Grammonota maritima
KKCHE920-09|07CHU-AR-0008|Grammonota maritima
KKCHE956-09|07CHU-AR-0044|Grammonota maritima
SPICH692-09|09PROBE-1978-449|Grammonota maritima
SPICH698-09|09PROBE-1978-455|Grammonota maritima
Grammonota maritima

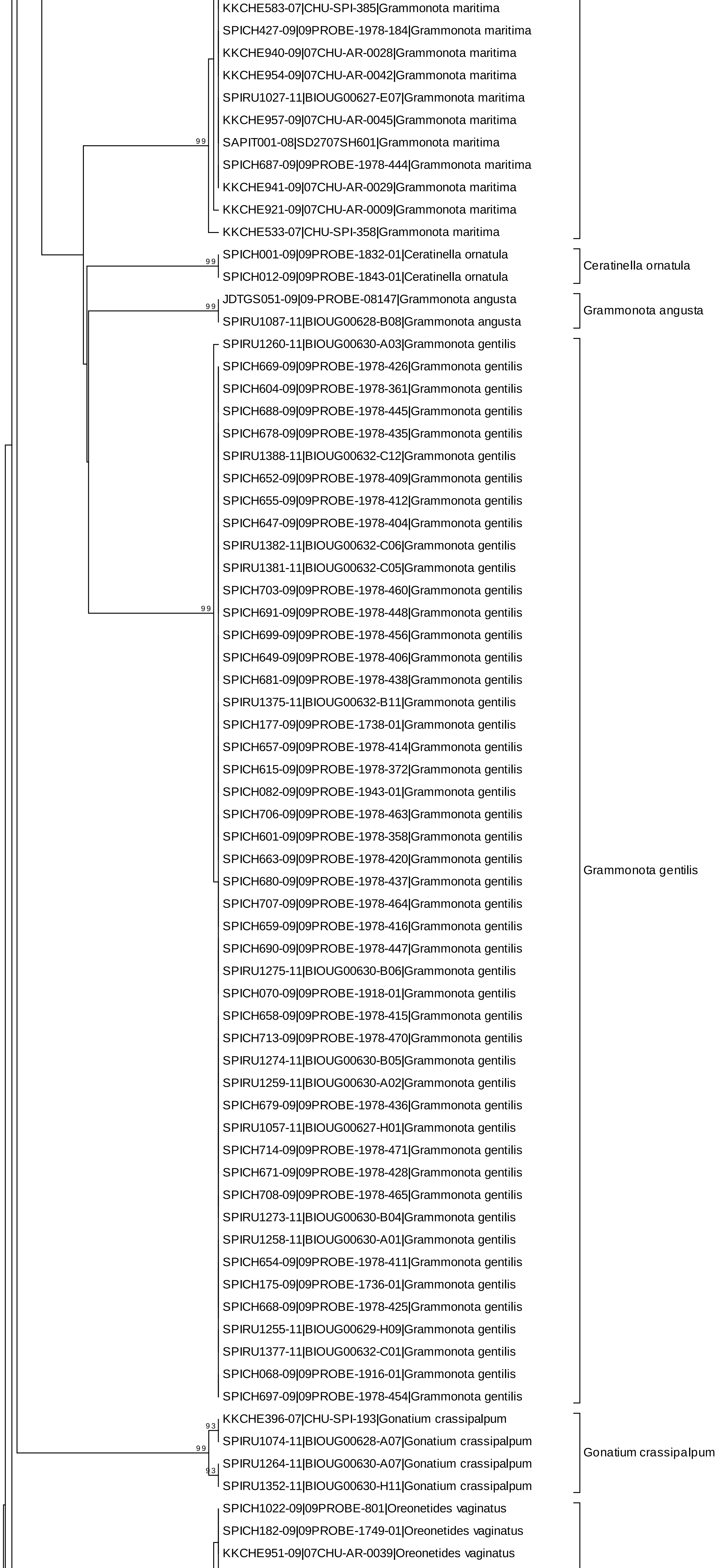

KKCHE583-07|CHU-SPI-385|Grammonota maritima
SPICH427-09|09PROBE-1978-184|Grammonota maritima
KKCHE940-09|07CHU-AR-0028|Grammonota maritima
KKCHE954-09|07CHU-AR-0042|Grammonota maritima
SPIRU1027-11|BIOUG00627-E07|Grammonota maritima
KKCHE957-09|07CHU-AR-0045|Grammonota maritima
SAPIT001-08|SD2707SH601|Grammonota maritima
SPICH687-09|09PROBE-1978-444|Grammonota maritima
KKCHE941-09|07CHU-AR-0029|Grammonota maritima
KKCHE921-09|07CHU-AR-0009|Grammonota maritima
KKCHE533-07|CHU-SPI-358|Grammonota maritima
99
SPICH001-09|09PROBE-1832-01|Ceratinella ornatula
SPICH012-09|09PROBE-1843-01|Ceratinella ornatula
99
Ceratinella ornatula
JDTGS051-09|09-PROBE-08147|Grammonota angusta
SPIRU1087-11|BIOUG00628-B08|Grammonota angusta
99
Grammonota angusta
SPIRU1260-11|BIOUG00630-A03|Grammonota gentilis
SPICH669-09|09PROBE-1978-426|Grammonota gentilis
SPICH604-09|09PROBE-1978-361|Grammonota gentilis
SPICH688-09|09PROBE-1978-445|Grammonota gentilis
SPICH678-09|09PROBE-1978-435|Grammonota gentilis
SPIRU1388-11|BIOUG00632-C12|Grammonota gentilis
SPICH652-09|09PROBE-1978-409|Grammonota gentilis
SPICH655-09|09PROBE-1978-412|Grammonota gentilis
SPICH647-09|09PROBE-1978-404|Grammonota gentilis
SPIRU1382-11|BIOUG00632-C06|Grammonota gentilis
SPIRU1381-11|BIOUG00632-C05|Grammonota gentilis
SPICH703-09|09PROBE-1978-460|Grammonota gentilis
SPICH691-09|09PROBE-1978-448|Grammonota gentilis
SPICH699-09|09PROBE-1978-456|Grammonota gentilis
SPICH649-09|09PROBE-1978-406|Grammonota gentilis
SPICH681-09|09PROBE-1978-438|Grammonota gentilis
SPIRU1375-11|BIOUG00632-B11|Grammonota gentilis
SPICH177-09|09PROBE-1738-01|Grammonota gentilis
SPICH657-09|09PROBE-1978-414|Grammonota gentilis
SPICH615-09|09PROBE-1978-372|Grammonota gentilis
SPICH082-09|09PROBE-1943-01|Grammonota gentilis
SPICH706-09|09PROBE-1978-463|Grammonota gentilis
SPICH601-09|09PROBE-1978-358|Grammonota gentilis
SPICH663-09|09PROBE-1978-420|Grammonota gentilis
SPICH680-09|09PROBE-1978-437|Grammonota gentilis
SPICH707-09|09PROBE-1978-464|Grammonota gentilis
SPICH659-09|09PROBE-1978-416|Grammonota gentilis
SPICH690-09|09PROBE-1978-447|Grammonota gentilis
SPIRU1275-11|BIOUG00630-B06|Grammonota gentilis
SPICH070-09|09PROBE-1918-01|Grammonota gentilis
SPICH658-09|09PROBE-1978-415|Grammonota gentilis
SPICH713-09|09PROBE-1978-470|Grammonota gentilis
SPIRU1274-11|BIOUG00630-B05|Grammonota gentilis
SPIRU1259-11|BIOUG00630-A02|Grammonota gentilis
SPICH679-09|09PROBE-1978-436|Grammonota gentilis
SPIRU1057-11|BIOUG00627-H01|Grammonota gentilis
SPICH714-09|09PROBE-1978-471|Grammonota gentilis
SPICH671-09|09PROBE-1978-428|Grammonota gentilis
SPICH708-09|09PROBE-1978-465|Grammonota gentilis
SPIRU1273-11|BIOUG00630-B04|Grammonota gentilis
SPIRU1258-11|BIOUG00630-A01|Grammonota gentilis
SPICH654-09|09PROBE-1978-411|Grammonota gentilis
SPICH175-09|09PROBE-1736-01|Grammonota gentilis
SPICH668-09|09PROBE-1978-425|Grammonota gentilis
SPIRU1255-11|BIOUG00629-H09|Grammonota gentilis
SPIRU1377-11|BIOUG00632-C01|Grammonota gentilis
SPICH068-09|09PROBE-1916-01|Grammonota gentilis
SPICH697-09|09PROBE-1978-454|Grammonota gentilis
99
Grammonota gentilis
KKCHE396-07|CHU-SPI-193|Gonatium crassipalpum
SPIRU1074-11|BIOUG00628-A07|Gonatium crassipalpum
SPIRU1264-11|BIOUG00630-A07|Gonatium crassipalpum
SPIRU1352-11|BIOUG00630-H11|Gonatium crassipalpum
93
99
93
Gonatium crassipalpum
SPICH1022-09|09PROBE-801|Oreonetides vaginatus
SPICH182-09|09PROBE-1749-01|Oreonetides vaginatus
KKCHE951-09|07CHU-AR-0039|Oreonetides vaginatus

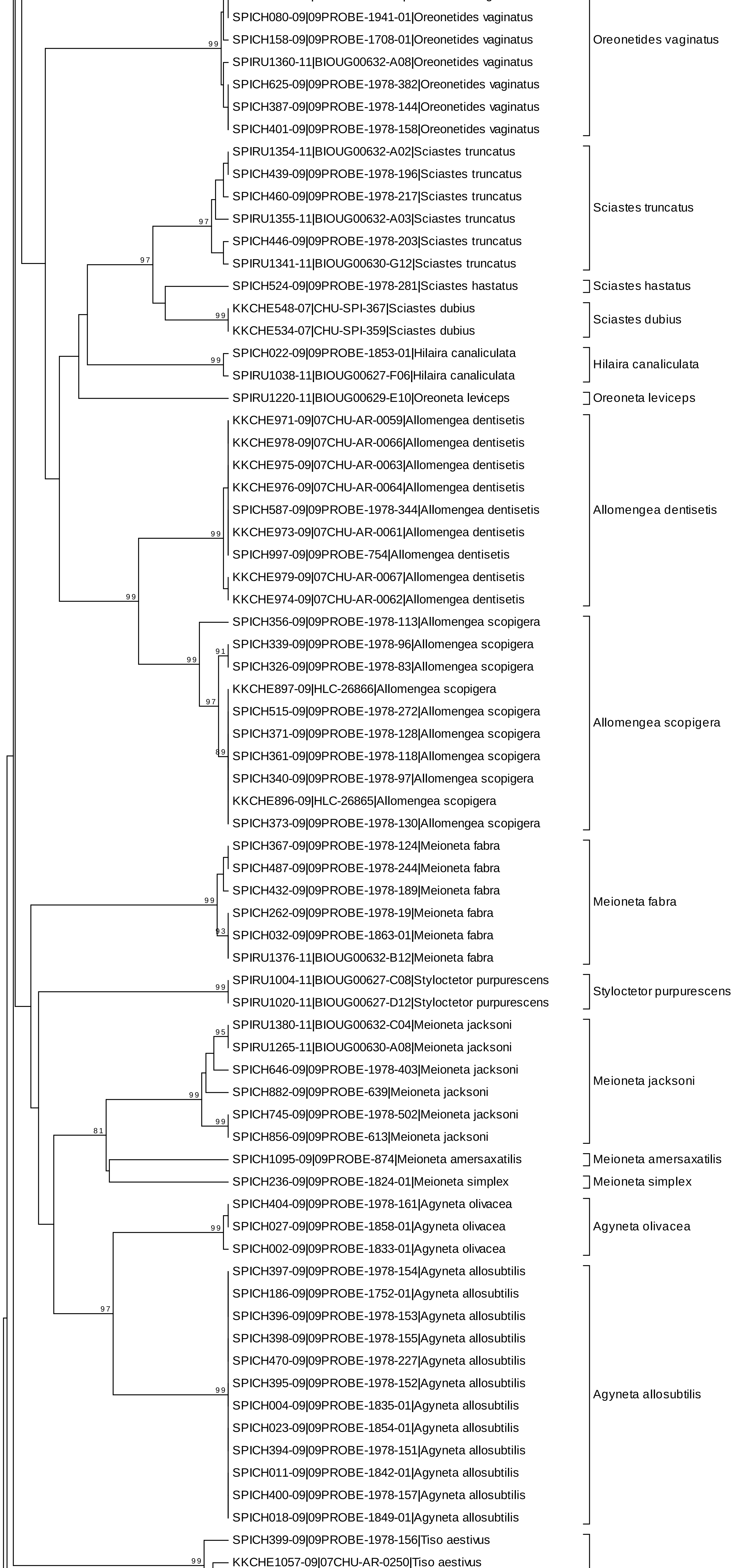
SPICH080-09|09PROBE-1941-01|Oreonetides vaginatus
SPICH158-09|09PROBE-1708-01|Oreonetides vaginatus
SPIRU1360-11|BIOUG00632-A08|Oreonetides vaginatus
SPICH625-09|09PROBE-1978-382|Oreonetides vaginatus
SPICH387-09|09PROBE-1978-144|Oreonetides vaginatus
SPICH401-09|09PROBE-1978-158|Oreonetides vaginatus
Oreonetides vaginatus
SPIRU1354-11|BIOUG00632-A02|Sciastes truncatus
SPICH439-09|09PROBE-1978-196|Sciastes truncatus
SPICH460-09|09PROBE-1978-217|Sciastes truncatus
SPIRU1355-11|BIOUG00632-A03|Sciastes truncatus
SPICH446-09|09PROBE-1978-203|Sciastes truncatus
SPIRU1341-11|BIOUG00630-G12|Sciastes truncatus
Sciastes truncatus
SPICH524-09|09PROBE-1978-281|Sciastes hastatus
Sciastes hastatus
KKCHE548-07|CHU-SPI-367|Sciastes dubius
KKCHE534-07|CHU-SPI-359|Sciastes dubius
Sciastes dubius
SPICH022-09|09PROBE-1853-01|Hilaira canaliculata
SPIRU1038-11|BIOUG00627-F06|Hilaira canaliculata
Hilaira canaliculata
SPIRU1220-11|BIOUG00629-E10|Oreoneta leviceps
Oreoneta leviceps
KKCHE971-09|07CHU-AR-0059|Allomengea dentisetis
KKCHE978-09|07CHU-AR-0066|Allomengea dentisetis
KKCHE975-09|07CHU-AR-0063|Allomengea dentisetis
KKCHE976-09|07CHU-AR-0064|Allomengea dentisetis
SPICH587-09|09PROBE-1978-344|Allomengea dentisetis
KKCHE973-09|07CHU-AR-0061|Allomengea dentisetis
SPICH997-09|09PROBE-754|Allomengea dentisetis
KKCHE979-09|07CHU-AR-0067|Allomengea dentisetis
KKCHE974-09|07CHU-AR-0062|Allomengea dentisetis
Allomengea dentisetis
SPICH356-09|09PROBE-1978-113|Allomengea scopigera
SPICH339-09|09PROBE-1978-96|Allomengea scopigera
SPICH326-09|09PROBE-1978-83|Allomengea scopigera
KKCHE897-09|HLC-26866|Allomengea scopigera
SPICH515-09|09PROBE-1978-272|Allomengea scopigera
SPICH371-09|09PROBE-1978-128|Allomengea scopigera
SPICH361-09|09PROBE-1978-118|Allomengea scopigera
SPICH340-09|09PROBE-1978-97|Allomengea scopigera
KKCHE896-09|HLC-26865|Allomengea scopigera
SPICH373-09|09PROBE-1978-130|Allomengea scopigera
Allomengea scopigera
SPICH367-09|09PROBE-1978-124|Meioneta fabra
SPICH487-09|09PROBE-1978-244|Meioneta fabra
SPICH432-09|09PROBE-1978-189|Meioneta fabra
SPICH262-09|09PROBE-1978-19|Meioneta fabra
SPICH032-09|09PROBE-1863-01|Meioneta fabra
SPIRU1376-11|BIOUG00632-B12|Meioneta fabra
Meioneta fabra
SPIRU1004-11|BIOUG00627-C08|Styloctetor purpurescens
SPIRU1020-11|BIOUG00627-D12|Styloctetor purpurescens
Styloctetor purpurescens
SPIRU1380-11|BIOUG00632-C04|Meioneta jacksoni
SPIRU1265-11|BIOUG00630-A08|Meioneta jacksoni
SPICH646-09|09PROBE-1978-403|Meioneta jacksoni
SPICH882-09|09PROBE-639|Meioneta jacksoni
SPICH745-09|09PROBE-1978-502|Meioneta jacksoni
SPICH856-09|09PROBE-613|Meioneta jacksoni
Meioneta jacksoni
SPICH1095-09|09PROBE-874|Meioneta amersaxatilis
Meioneta amersaxatilis
SPICH236-09|09PROBE-1824-01|Meioneta simplex
Meioneta simplex
SPICH404-09|09PROBE-1978-161|Agyneta olivacea
SPICH027-09|09PROBE-1858-01|Agyneta olivacea
SPICH002-09|09PROBE-1833-01|Agyneta olivacea
Agyneta olivacea
SPICH397-09|09PROBE-1978-154|Agyneta allosubtilis
SPICH186-09|09PROBE-1752-01|Agyneta allosubtilis
SPICH396-09|09PROBE-1978-153|Agyneta allosubtilis
SPICH398-09|09PROBE-1978-155|Agyneta allosubtilis
SPICH470-09|09PROBE-1978-227|Agyneta allosubtilis
SPICH395-09|09PROBE-1978-152|Agyneta allosubtilis
SPICH004-09|09PROBE-1835-01|Agyneta allosubtilis
SPICH023-09|09PROBE-1854-01|Agyneta allosubtilis
SPICH394-09|09PROBE-1978-151|Agyneta allosubtilis
SPICH011-09|09PROBE-1842-01|Agyneta allosubtilis
SPICH400-09|09PROBE-1978-157|Agyneta allosubtilis
SPICH018-09|09PROBE-1849-01|Agyneta allosubtilis
Agyneta allosubtilis
SPICH399-09|09PROBE-1978-156|Tiso aestivus
KKCHE1057-09|07CHU-AR-0250|Tiso aestivus

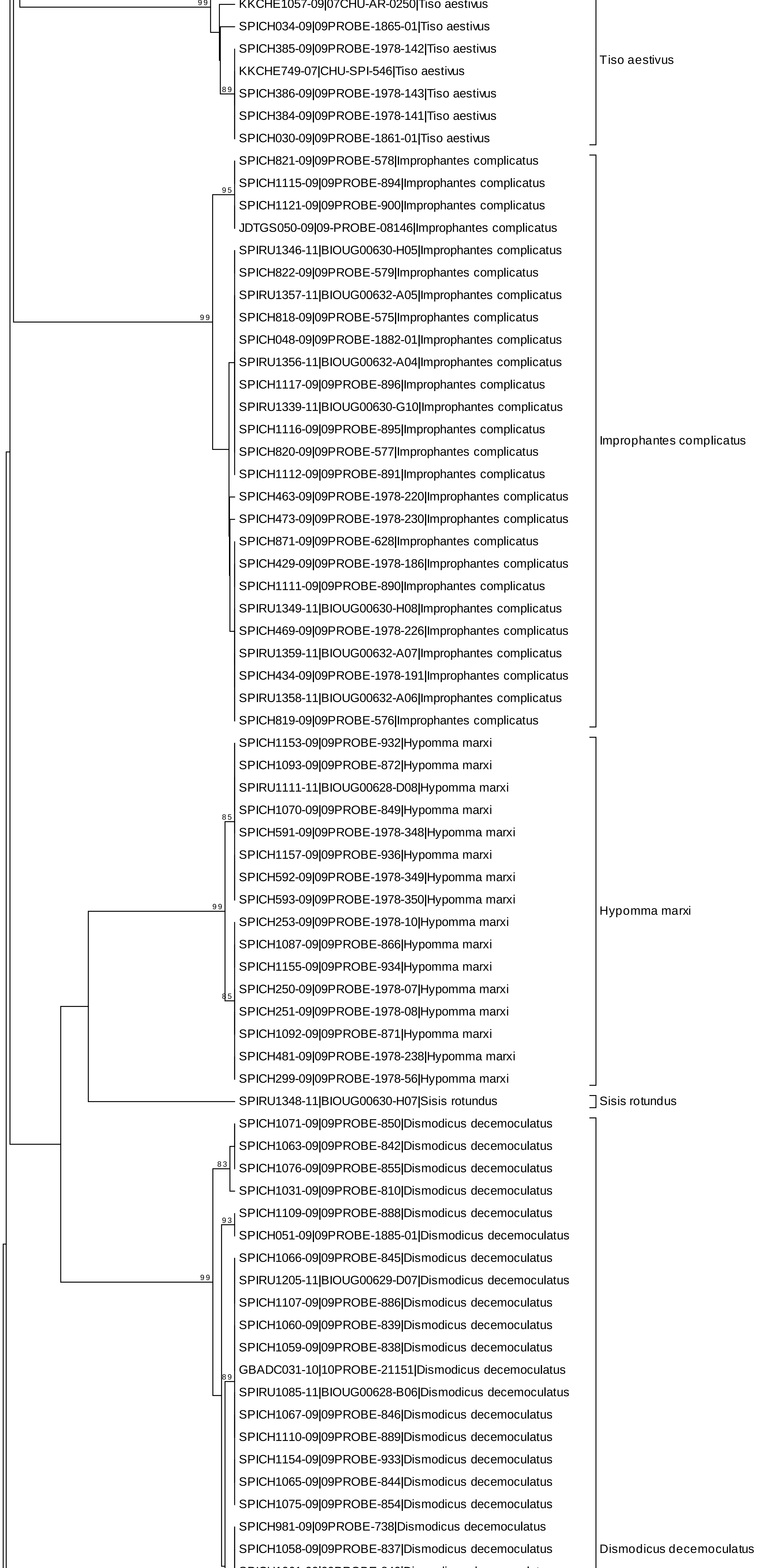
KKCHE1057-09|07CHU-AR-0250|Tiso aestivus
SPICH034-09|09PROBE-1865-01|Tiso aestivus
SPICH385-09|09PROBE-1978-142|Tiso aestivus
KKCHE749-07|CHU-SPI-546|Tiso aestivus
SPICH386-09|09PROBE-1978-143|Tiso aestivus
SPICH384-09|09PROBE-1978-141|Tiso aestivus
SPICH030-09|09PROBE-1861-01|Tiso aestivus
Tiso aestivus
SPICH821-09|09PROBE-578|Improphantes complicatus
SPICH1115-09|09PROBE-894|Improphantes complicatus
SPICH1121-09|09PROBE-900|Improphantes complicatus
JDTGS050-09|09-PROBE-08146|Improphantes complicatus
SPIRU1346-11|BIOUG00630-H05|Improphantes complicatus
SPICH822-09|09PROBE-579|Improphantes complicatus
SPIRU1357-11|BIOUG00632-A05|Improphantes complicatus
SPICH818-09|09PROBE-575|Improphantes complicatus
SPICH048-09|09PROBE-1882-01|Improphantes complicatus
SPIRU1356-11|BIOUG00632-A04|Improphantes complicatus
SPICH1117-09|09PROBE-896|Improphantes complicatus
SPIRU1339-11|BIOUG00630-G10|Improphantes complicatus
SPICH1116-09|09PROBE-895|Improphantes complicatus
SPICH820-09|09PROBE-577|Improphantes complicatus
SPICH1112-09|09PROBE-891|Improphantes complicatus
SPICH463-09|09PROBE-1978-220|Improphantes complicatus
SPICH473-09|09PROBE-1978-230|Improphantes complicatus
SPICH871-09|09PROBE-628|Improphantes complicatus
SPICH429-09|09PROBE-1978-186|Improphantes complicatus
SPICH1111-09|09PROBE-890|Improphantes complicatus
SPIRU1349-11|BIOUG00630-H08|Improphantes complicatus
SPICH469-09|09PROBE-1978-226|Improphantes complicatus
SPIRU1359-11|BIOUG00632-A07|Improphantes complicatus
SPICH434-09|09PROBE-1978-191|Improphantes complicatus
SPIRU1358-11|BIOUG00632-A06|Improphantes complicatus
SPICH819-09|09PROBE-576|Improphantes complicatus
Improphantes complicatus
SPICH1153-09|09PROBE-932|Hypomma marxi
SPICH1093-09|09PROBE-872|Hypomma marxi
SPIRU1111-11|BIOUG00628-D08|Hypomma marxi
SPICH1070-09|09PROBE-849|Hypomma marxi
SPICH591-09|09PROBE-1978-348|Hypomma marxi
SPICH1157-09|09PROBE-936|Hypomma marxi
SPICH592-09|09PROBE-1978-349|Hypomma marxi
SPICH593-09|09PROBE-1978-350|Hypomma marxi
SPICH253-09|09PROBE-1978-10|Hypomma marxi
SPICH1087-09|09PROBE-866|Hypomma marxi
SPICH1155-09|09PROBE-934|Hypomma marxi
SPICH250-09|09PROBE-1978-07|Hypomma marxi
SPICH251-09|09PROBE-1978-08|Hypomma marxi
SPICH1092-09|09PROBE-871|Hypomma marxi
SPICH481-09|09PROBE-1978-238|Hypomma marxi
SPICH299-09|09PROBE-1978-56|Hypomma marxi
Hypomma marxi
SPIRU1348-11|BIOUG00630-H07|Sisis rotundus
Sisis rotundus
SPICH1071-09|09PROBE-850|Dismodicus decemoculatus
SPICH1063-09|09PROBE-842|Dismodicus decemoculatus
SPICH1076-09|09PROBE-855|Dismodicus decemoculatus
SPICH1031-09|09PROBE-810|Dismodicus decemoculatus
SPICH1109-09|09PROBE-888|Dismodicus decemoculatus
SPICH051-09|09PROBE-1885-01|Dismodicus decemoculatus
SPICH1066-09|09PROBE-845|Dismodicus decemoculatus
SPIRU1205-11|BIOUG00629-D07|Dismodicus decemoculatus
SPICH1107-09|09PROBE-886|Dismodicus decemoculatus
SPICH1060-09|09PROBE-839|Dismodicus decemoculatus
SPICH1059-09|09PROBE-838|Dismodicus decemoculatus
GBADC031-10|10PROBE-21151|Dismodicus decemoculatus
SPIRU1085-11|BIOUG00628-B06|Dismodicus decemoculatus
SPICH1067-09|09PROBE-846|Dismodicus decemoculatus
SPICH1110-09|09PROBE-889|Dismodicus decemoculatus
SPICH1154-09|09PROBE-933|Dismodicus decemoculatus
SPICH1065-09|09PROBE-844|Dismodicus decemoculatus
SPICH1075-09|09PROBE-854|Dismodicus decemoculatus
SPICH981-09|09PROBE-738|Dismodicus decemoculatus
SPICH1058-09|09PROBE-837|Dismodicus decemoculatus
Dismodicus decemoculatus

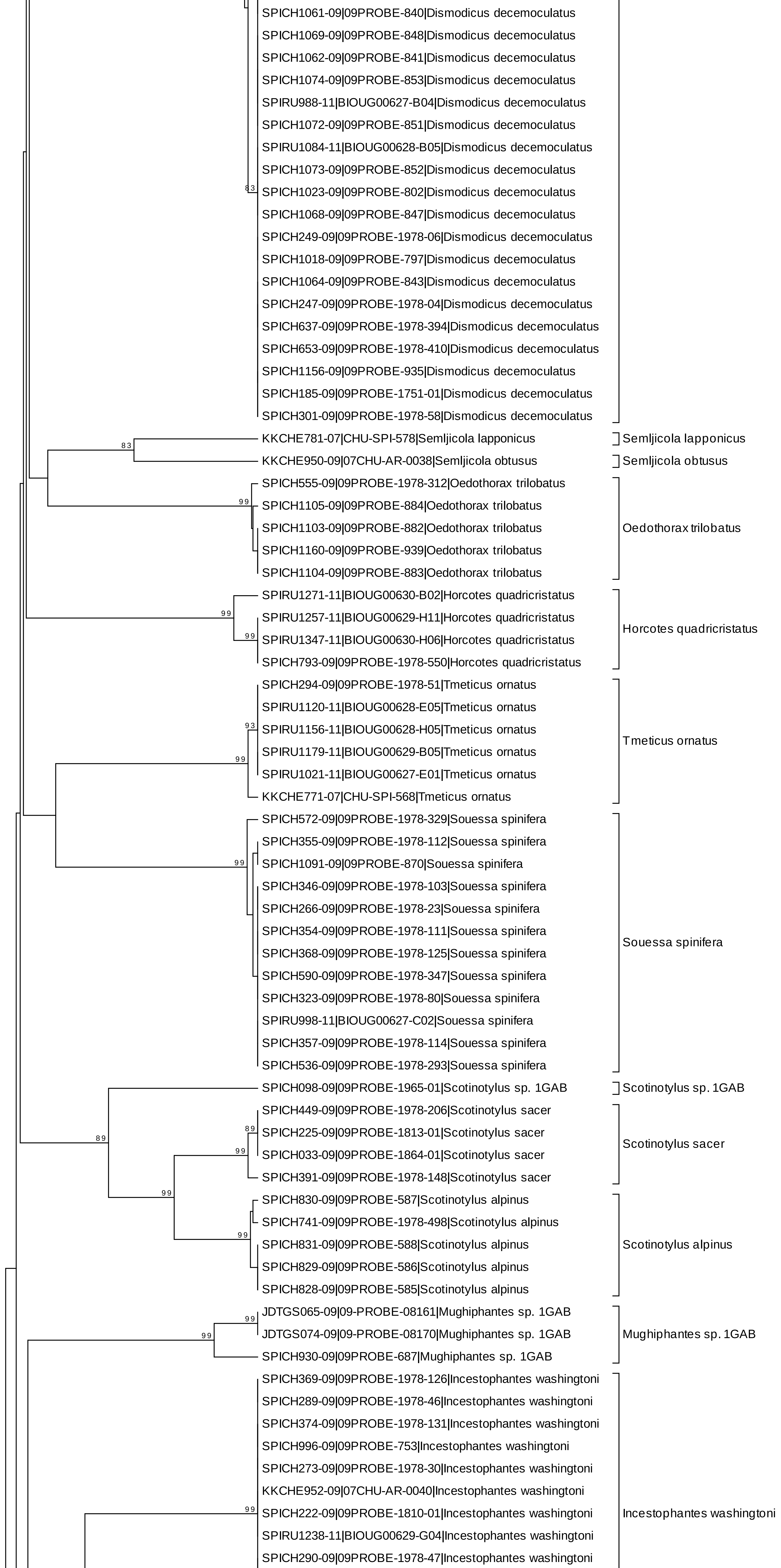
SPICH1061-09|09PROBE-840|Dismodicus decemoculatus
SPICH1069-09|09PROBE-848|Dismodicus decemoculatus
SPICH1062-09|09PROBE-841|Dismodicus decemoculatus
SPICH1074-09|09PROBE-853|Dismodicus decemoculatus
SPIRU988-11|BIOUG00627-B04|Dismodicus decemoculatus
SPICH1072-09|09PROBE-851|Dismodicus decemoculatus
SPIRU1084-11|BIOUG00628-B05|Dismodicus decemoculatus
SPICH1073-09|09PROBE-852|Dismodicus decemoculatus
83
SPICH1023-09|09PROBE-802|Dismodicus decemoculatus
SPICH1068-09|09PROBE-847|Dismodicus decemoculatus
SPICH249-09|09PROBE-1978-06|Dismodicus decemoculatus
SPICH1018-09|09PROBE-797|Dismodicus decemoculatus
SPICH1064-09|09PROBE-843|Dismodicus decemoculatus
SPICH247-09|09PROBE-1978-04|Dismodicus decemoculatus
SPICH637-09|09PROBE-1978-394|Dismodicus decemoculatus
SPICH653-09|09PROBE-1978-410|Dismodicus decemoculatus
SPICH1156-09|09PROBE-935|Dismodicus decemoculatus
SPICH185-09|09PROBE-1751-01|Dismodicus decemoculatus
SPICH301-09|09PROBE-1978-58|Dismodicus decemoculatus
83
KKCHE781-07|CHU-SPI-578|Semljicola lapponicus
Semljicola lapponicus
KKCHE950-09|07CHU-AR-0038|Semljicola obtusus
Semljicola obtusus
SPICH555-09|09PROBE-1978-312|Oedothorax trilobatus
99
SPICH1105-09|09PROBE-884|Oedothorax trilobatus
SPICH1103-09|09PROBE-882|Oedothorax trilobatus
SPICH1160-09|09PROBE-939|Oedothorax trilobatus
SPICH1104-09|09PROBE-883|Oedothorax trilobatus
Oedothorax trilobatus
SPIRU1271-11|BIOUG00630-B02|Horcotes quadricristatus
99
SPIRU1257-11|BIOUG00629-H11|Horcotes quadricristatus
99
SPIRU1347-11|BIOUG00630-H06|Horcotes quadricristatus
SPICH793-09|09PROBE-1978-550|Horcotes quadricristatus
Horcotes quadricristatus
SPICH294-09|09PROBE-1978-51|Tmeticus ornatus
SPIRU1120-11|BIOUG00628-E05|Tmeticus ornatus
93
SPIRU1156-11|BIOUG00628-H05|Tmeticus ornatus
SPIRU1179-11|BIOUG00629-B05|Tmeticus ornatus
99
SPIRU1021-11|BIOUG00627-E01|Tmeticus ornatus
KKCHE771-07|CHU-SPI-568|Tmeticus ornatus
Tmeticus ornatus
SPICH572-09|09PROBE-1978-329|Souessa spinifera
SPICH355-09|09PROBE-1978-112|Souessa spinifera
99
SPICH1091-09|09PROBE-870|Souessa spinifera
SPICH346-09|09PROBE-1978-103|Souessa spinifera
SPICH266-09|09PROBE-1978-23|Souessa spinifera
SPICH354-09|09PROBE-1978-111|Souessa spinifera
SPICH368-09|09PROBE-1978-125|Souessa spinifera
SPICH590-09|09PROBE-1978-347|Souessa spinifera
SPICH323-09|09PROBE-1978-80|Souessa spinifera
SPIRU998-11|BIOUG00627-C02|Souessa spinifera
SPICH357-09|09PROBE-1978-114|Souessa spinifera
SPICH536-09|09PROBE-1978-293|Souessa spinifera
Souessa spinifera
SPICH098-09|09PROBE-1965-01|Scotinotylus sp. 1GAB
Scotinotylus sp. 1GAB
SPICH449-09|09PROBE-1978-206|Scotinotylus sacer
89
SPICH225-09|09PROBE-1813-01|Scotinotylus sacer
89
99
SPICH033-09|09PROBE-1864-01|Scotinotylus sacer
SPICH391-09|09PROBE-1978-148|Scotinotylus sacer
Scotinotylus sacer
99
SPICH830-09|09PROBE-587|Scotinotylus alpinus
SPICH741-09|09PROBE-1978-498|Scotinotylus alpinus
99
SPICH831-09|09PROBE-588|Scotinotylus alpinus
SPICH829-09|09PROBE-586|Scotinotylus alpinus
SPICH828-09|09PROBE-585|Scotinotylus alpinus
Scotinotylus alpinus
JDTGS065-09|09-PROBE-08161|Mughiphantes sp. 1GAB
99
99
JDTGS074-09|09-PROBE-08170|Mughiphantes sp. 1GAB
SPICH930-09|09PROBE-687|Mughiphantes sp. 1GAB
Mughiphantes sp. 1GAB
SPICH369-09|09PROBE-1978-126|Incestophantes washingtoni
SPICH289-09|09PROBE-1978-46|Incestophantes washingtoni
SPICH374-09|09PROBE-1978-131|Incestophantes washingtoni
SPICH996-09|09PROBE-753|Incestophantes washingtoni
SPICH273-09|09PROBE-1978-30|Incestophantes washingtoni
KKCHE952-09|07CHU-AR-0040|Incestophantes washingtoni
99
SPICH222-09|09PROBE-1810-01|Incestophantes washingtoni
SPIRU1238-11|BIOUG00629-G04|Incestophantes washingtoni
SPICH290-09|09PROBE-1978-47|Incestophantes washingtoni
Incestophantes washingtoni

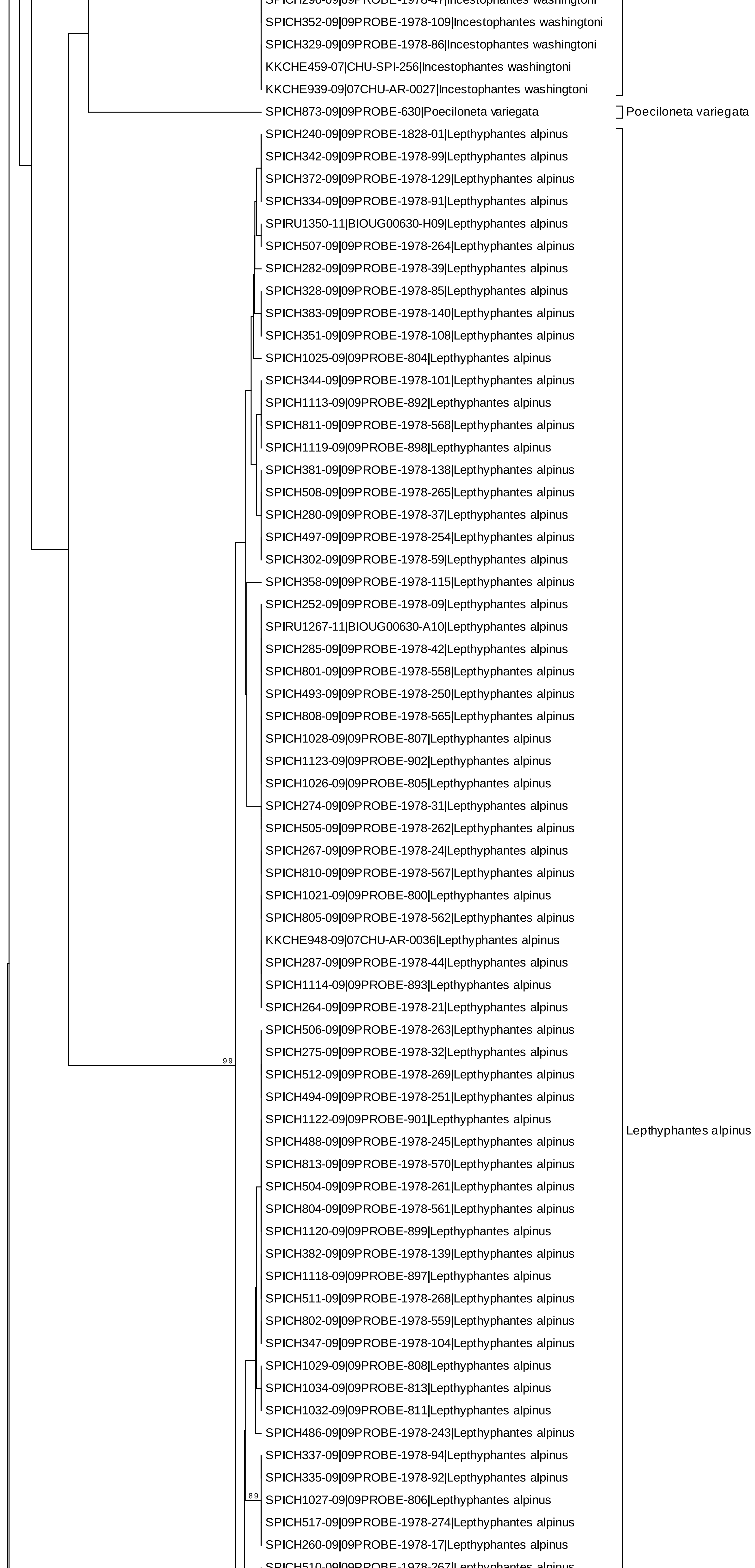
SPICH352-09|09PROBE-1978-109|Incestophantes washingtoni
SPICH329-09|09PROBE-1978-86|Incestophantes washingtoni
KKCHE459-07|CHU-SPI-256|Incestophantes washingtoni
KKCHE939-09|07CHU-AR-0027|Incestophantes washingtoni
SPICH873-09|09PROBE-630|Poeciloneta variegata
Poeciloneta variegata
SPICH240-09|09PROBE-1828-01|Lepthyphantes alpinus
SPICH342-09|09PROBE-1978-99|Lepthyphantes alpinus
SPICH372-09|09PROBE-1978-129|Lepthyphantes alpinus
SPICH334-09|09PROBE-1978-91|Lepthyphantes alpinus
SPIRU1350-11|BIOUG00630-H09|Lepthyphantes alpinus
SPICH507-09|09PROBE-1978-264|Lepthyphantes alpinus
SPICH282-09|09PROBE-1978-39|Lepthyphantes alpinus
SPICH328-09|09PROBE-1978-85|Lepthyphantes alpinus
SPICH383-09|09PROBE-1978-140|Lepthyphantes alpinus
SPICH351-09|09PROBE-1978-108|Lepthyphantes alpinus
SPICH1025-09|09PROBE-804|Lepthyphantes alpinus
SPICH344-09|09PROBE-1978-101|Lepthyphantes alpinus
SPICH1113-09|09PROBE-892|Lepthyphantes alpinus
SPICH811-09|09PROBE-1978-568|Lepthyphantes alpinus
SPICH1119-09|09PROBE-898|Lepthyphantes alpinus
SPICH381-09|09PROBE-1978-138|Lepthyphantes alpinus
SPICH508-09|09PROBE-1978-265|Lepthyphantes alpinus
SPICH280-09|09PROBE-1978-37|Lepthyphantes alpinus
SPICH497-09|09PROBE-1978-254|Lepthyphantes alpinus
SPICH302-09|09PROBE-1978-59|Lepthyphantes alpinus
SPICH358-09|09PROBE-1978-115|Lepthyphantes alpinus
SPICH252-09|09PROBE-1978-09|Lepthyphantes alpinus
SPIRU1267-11|BIOUG00630-A10|Lepthyphantes alpinus
SPICH285-09|09PROBE-1978-42|Lepthyphantes alpinus
SPICH801-09|09PROBE-1978-558|Lepthyphantes alpinus
SPICH493-09|09PROBE-1978-250|Lepthyphantes alpinus
SPICH808-09|09PROBE-1978-565|Lepthyphantes alpinus
SPICH1028-09|09PROBE-807|Lepthyphantes alpinus
SPICH1123-09|09PROBE-902|Lepthyphantes alpinus
SPICH1026-09|09PROBE-805|Lepthyphantes alpinus
SPICH274-09|09PROBE-1978-31|Lepthyphantes alpinus
SPICH505-09|09PROBE-1978-262|Lepthyphantes alpinus
SPICH267-09|09PROBE-1978-24|Lepthyphantes alpinus
SPICH810-09|09PROBE-1978-567|Lepthyphantes alpinus
SPICH1021-09|09PROBE-800|Lepthyphantes alpinus
SPICH805-09|09PROBE-1978-562|Lepthyphantes alpinus
KKCHE948-09|07CHU-AR-0036|Lepthyphantes alpinus
SPICH287-09|09PROBE-1978-44|Lepthyphantes alpinus
SPICH1114-09|09PROBE-893|Lepthyphantes alpinus
SPICH264-09|09PROBE-1978-21|Lepthyphantes alpinus
SPICH506-09|09PROBE-1978-263|Lepthyphantes alpinus
99
SPICH275-09|09PROBE-1978-32|Lepthyphantes alpinus
SPICH512-09|09PROBE-1978-269|Lepthyphantes alpinus
SPICH494-09|09PROBE-1978-251|Lepthyphantes alpinus
SPICH1122-09|09PROBE-901|Lepthyphantes alpinus
SPICH488-09|09PROBE-1978-245|Lepthyphantes alpinus
Lepthyphantes alpinus
SPICH813-09|09PROBE-1978-570|Lepthyphantes alpinus
SPICH504-09|09PROBE-1978-261|Lepthyphantes alpinus
SPICH804-09|09PROBE-1978-561|Lepthyphantes alpinus
SPICH1120-09|09PROBE-899|Lepthyphantes alpinus
SPICH382-09|09PROBE-1978-139|Lepthyphantes alpinus
SPICH1118-09|09PROBE-897|Lepthyphantes alpinus
SPICH511-09|09PROBE-1978-268|Lepthyphantes alpinus
SPICH802-09|09PROBE-1978-559|Lepthyphantes alpinus
SPICH347-09|09PROBE-1978-104|Lepthyphantes alpinus
SPICH1029-09|09PROBE-808|Lepthyphantes alpinus
SPICH1034-09|09PROBE-813|Lepthyphantes alpinus
SPICH1032-09|09PROBE-811|Lepthyphantes alpinus
SPICH486-09|09PROBE-1978-243|Lepthyphantes alpinus
SPICH337-09|09PROBE-1978-94|Lepthyphantes alpinus
SPICH335-09|09PROBE-1978-92|Lepthyphantes alpinus
89
SPICH1027-09|09PROBE-806|Lepthyphantes alpinus
SPICH517-09|09PROBE-1978-274|Lepthyphantes alpinus
SPICH260-09|09PROBE-1978-17|Lepthyphantes alpinus

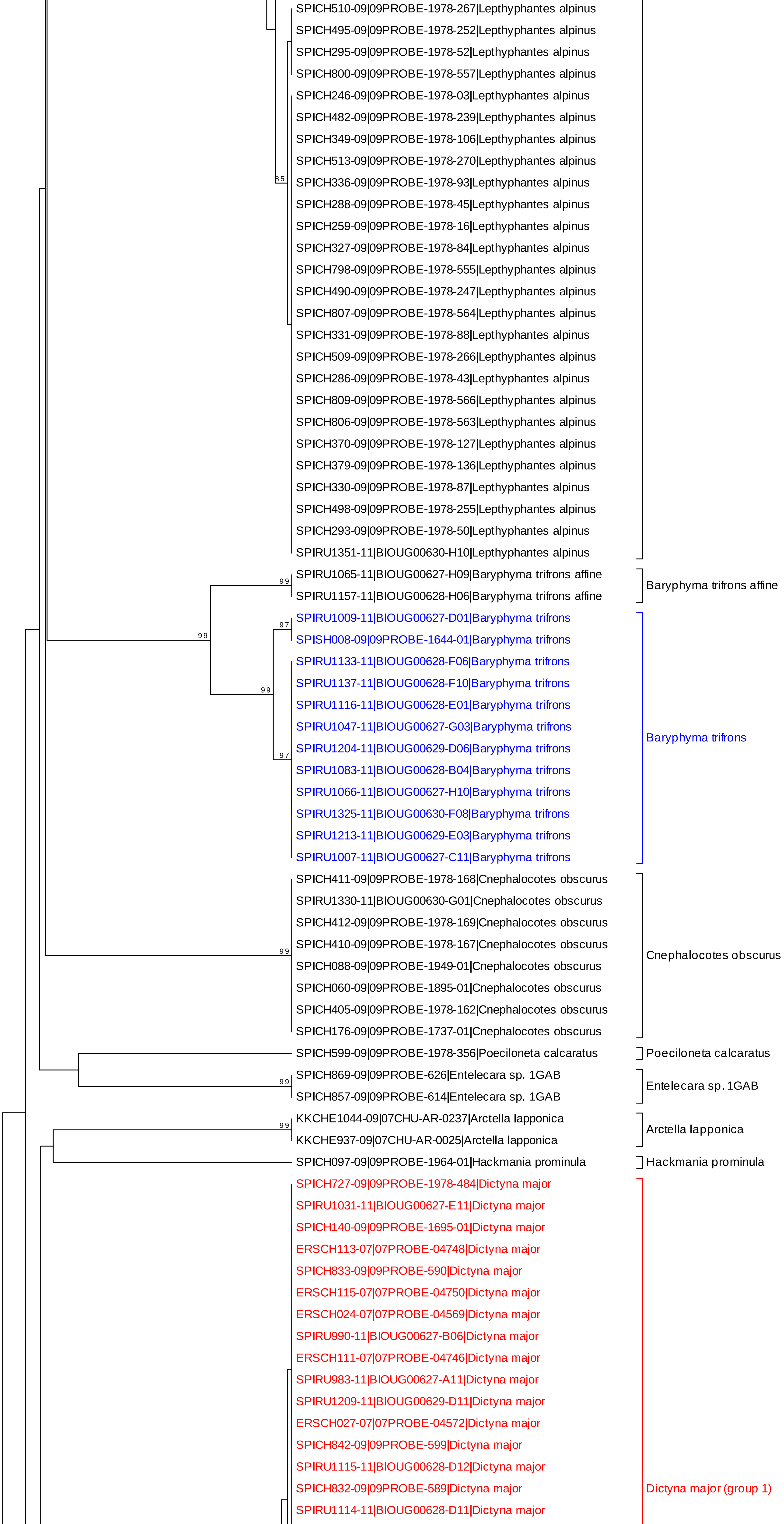

SPICH510-09|09PROBE-1978-267|Lepthyphantes alpinus
SPICH495-09|09PROBE-1978-252|Lepthyphantes alpinus
SPICH295-09|09PROBE-1978-52|Lepthyphantes alpinus
SPICH800-09|09PROBE-1978-557|Lepthyphantes alpinus
SPICH246-09|09PROBE-1978-03|Lepthyphantes alpinus
SPICH482-09|09PROBE-1978-239|Lepthyphantes alpinus
SPICH349-09|09PROBE-1978-106|Lepthyphantes alpinus
SPICH513-09|09PROBE-1978-270|Lepthyphantes alpinus
SPICH336-09|09PROBE-1978-93|Lepthyphantes alpinus
SPICH288-09|09PROBE-1978-45|Lepthyphantes alpinus
SPICH259-09|09PROBE-1978-16|Lepthyphantes alpinus
SPICH327-09|09PROBE-1978-84|Lepthyphantes alpinus
SPICH798-09|09PROBE-1978-555|Lepthyphantes alpinus
SPICH490-09|09PROBE-1978-247|Lepthyphantes alpinus
SPICH807-09|09PROBE-1978-564|Lepthyphantes alpinus
SPICH331-09|09PROBE-1978-88|Lepthyphantes alpinus
SPICH509-09|09PROBE-1978-266|Lepthyphantes alpinus
SPICH286-09|09PROBE-1978-43|Lepthyphantes alpinus
SPICH809-09|09PROBE-1978-566|Lepthyphantes alpinus
SPICH806-09|09PROBE-1978-563|Lepthyphantes alpinus
SPICH370-09|09PROBE-1978-127|Lepthyphantes alpinus
SPICH379-09|09PROBE-1978-136|Lepthyphantes alpinus
SPICH330-09|09PROBE-1978-87|Lepthyphantes alpinus
SPICH498-09|09PROBE-1978-255|Lepthyphantes alpinus
SPICH293-09|09PROBE-1978-50|Lepthyphantes alpinus
SPIRU1351-11|BIOUG00630-H10|Lepthyphantes alpinus
SPIRU1065-11|BIOUG00627-H09|Baryphyma trifrons affine
SPIRU1157-11|BIOUG00628-H06|Baryphyma trifrons affine
Baryphyma trifrons affine
SPIRU1009-11|BIOUG00627-D01|Baryphyma trifrons
SPISH008-09|09PROBE-1644-01|Baryphyma trifrons
SPIRU1133-11|BIOUG00628-F06|Baryphyma trifrons
SPIRU1137-11|BIOUG00628-F10|Baryphyma trifrons
SPIRU1116-11|BIOUG00628-E01|Baryphyma trifrons
SPIRU1047-11|BIOUG00627-G03|Baryphyma trifrons
SPIRU1204-11|BIOUG00629-D06|Baryphyma trifrons
SPIRU1083-11|BIOUG00628-B04|Baryphyma trifrons
SPIRU1066-11|BIOUG00627-H10|Baryphyma trifrons
SPIRU1325-11|BIOUG00630-F08|Baryphyma trifrons
SPIRU1213-11|BIOUG00629-E03|Baryphyma trifrons
SPIRU1007-11|BIOUG00627-C11|Baryphyma trifrons
Baryphyma trifrons
SPICH411-09|09PROBE-1978-168|Cnephalocotes obscurus
SPIRU1330-11|BIOUG00630-G01|Cnephalocotes obscurus
SPICH412-09|09PROBE-1978-169|Cnephalocotes obscurus
SPICH410-09|09PROBE-1978-167|Cnephalocotes obscurus
SPICH088-09|09PROBE-1949-01|Cnephalocotes obscurus
SPICH060-09|09PROBE-1895-01|Cnephalocotes obscurus
SPICH405-09|09PROBE-1978-162|Cnephalocotes obscurus
SPICH176-09|09PROBE-1737-01|Cnephalocotes obscurus
Cnephalocotes obscurus
SPICH599-09|09PROBE-1978-356|Poeciloneta calcaratus
Poeciloneta calcaratus
SPICH869-09|09PROBE-626|Entelecara sp. 1GAB
SPICH857-09|09PROBE-614|Entelecara sp. 1GAB
Entelecara sp. 1GAB
KKCHE1044-09|07CHU-AR-0237|Arctella lapponica
KKCHE937-09|07CHU-AR-0025|Arctella lapponica
Arctella lapponica
SPICH097-09|09PROBE-1964-01|Hackmania prominula
Hackmania prominula
SPICH727-09|09PROBE-1978-484|Dictyna major
SPIRU1031-11|BIOUG00627-E11|Dictyna major
SPICH140-09|09PROBE-1695-01|Dictyna major
ERSCH113-07|07PROBE-04748|Dictyna major
SPICH833-09|09PROBE-590|Dictyna major
ERSCH115-07|07PROBE-04750|Dictyna major
ERSCH024-07|07PROBE-04569|Dictyna major
SPIRU990-11|BIOUG00627-B06|Dictyna major
ERSCH111-07|07PROBE-04746|Dictyna major
SPIRU983-11|BIOUG00627-A11|Dictyna major
SPIRU1209-11|BIOUG00629-D11|Dictyna major
ERSCH027-07|07PROBE-04572|Dictyna major
SPICH842-09|09PROBE-599|Dictyna major
SPIRU1115-11|BIOUG00628-D12|Dictyna major
SPICH832-09|09PROBE-589|Dictyna major
SPIRU1114-11|BIOUG00628-D11|Dictyna major
Dictyna major (group 1)

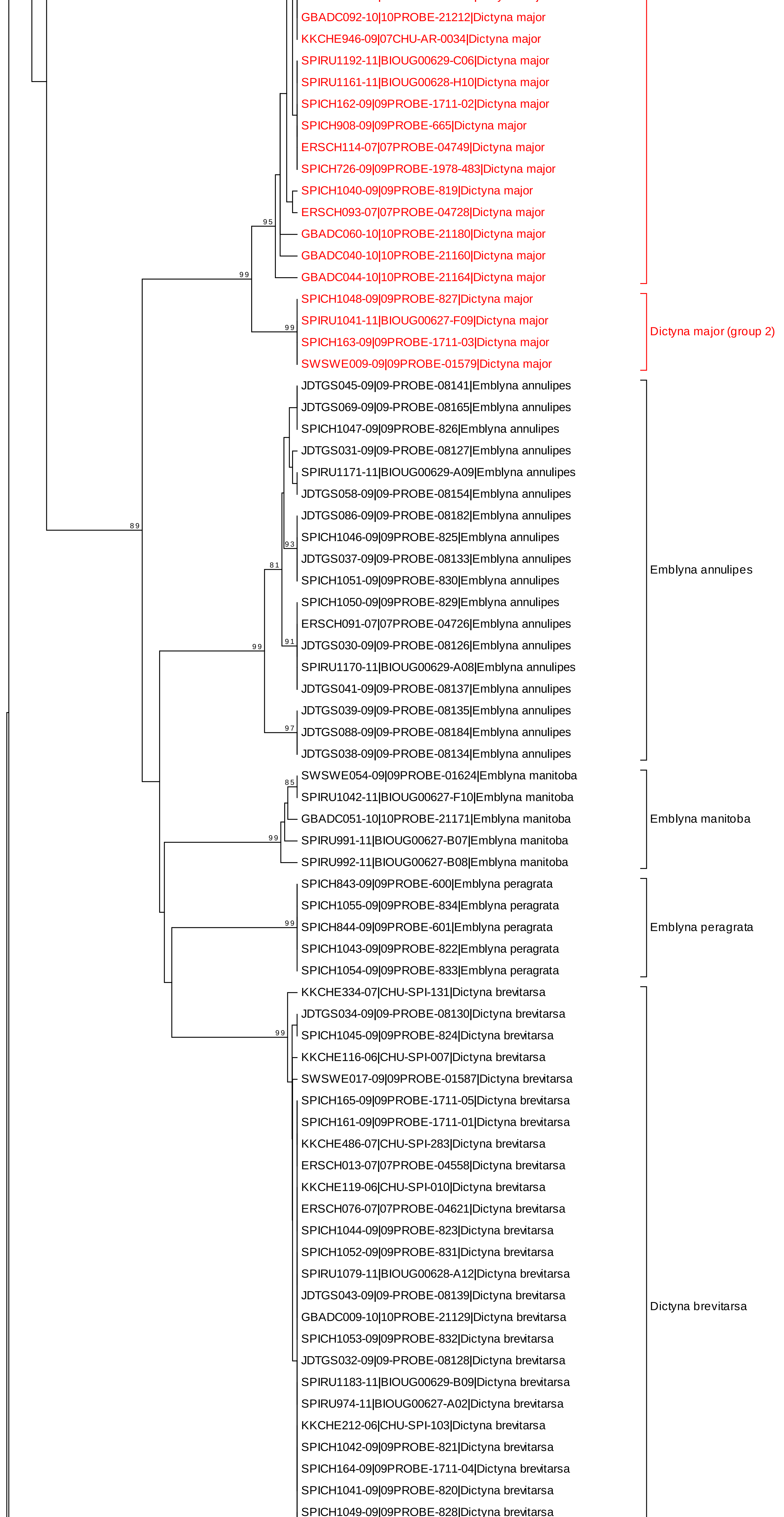
GBADC092-10|10PROBE-21212|Dictyna major
KKCHE946-09|07CHU-AR-0034|Dictyna major
SPIRU1192-11|BIOUG00629-C06|Dictyna major
SPIRU1161-11|BIOUG00628-H10|Dictyna major
SPICH162-09|09PROBE-1711-02|Dictyna major
SPICH908-09|09PROBE-665|Dictyna major
ERSCH114-07|07PROBE-04749|Dictyna major
SPICH726-09|09PROBE-1978-483|Dictyna major
SPICH1040-09|09PROBE-819|Dictyna major
ERSCH093-07|07PROBE-04728|Dictyna major
95
GBADC060-10|10PROBE-21180|Dictyna major
GBADC040-10|10PROBE-21160|Dictyna major
99
GBADC044-10|10PROBE-21164|Dictyna major
SPICH1048-09|09PROBE-827|Dictyna major
SPIRU1041-11|BIOUG00627-F09|Dictyna major
99
SPICH163-09|09PROBE-1711-03|Dictyna major
SWSWE009-09|09PROBE-01579|Dictyna major
Dictyna major (group 2)
JDTGS045-09|09-PROBE-08141|Emblyna annulipes
JDTGS069-09|09-PROBE-08165|Emblyna annulipes
SPICH1047-09|09PROBE-826|Emblyna annulipes
JDTGS031-09|09-PROBE-08127|Emblyna annulipes
SPIRU1171-11|BIOUG00629-A09|Emblyna annulipes
JDTGS058-09|09-PROBE-08154|Emblyna annulipes
JDTGS086-09|09-PROBE-08182|Emblyna annulipes
89
SPICH1046-09|09PROBE-825|Emblyna annulipes
93
JDTGS037-09|09-PROBE-08133|Emblyna annulipes
81
SPICH1051-09|09PROBE-830|Emblyna annulipes
SPICH1050-09|09PROBE-829|Emblyna annulipes
ERSCH091-07|07PROBE-04726|Emblyna annulipes
91
JDTGS030-09|09-PROBE-08126|Emblyna annulipes
99
SPIRU1170-11|BIOUG00629-A08|Emblyna annulipes
JDTGS041-09|09-PROBE-08137|Emblyna annulipes
JDTGS039-09|09-PROBE-08135|Emblyna annulipes
97
JDTGS088-09|09-PROBE-08184|Emblyna annulipes
JDTGS038-09|09-PROBE-08134|Emblyna annulipes
Emblyna annulipes
85
SWSWE054-09|09PROBE-01624|Emblyna manitoba
SPIRU1042-11|BIOUG00627-F10|Emblyna manitoba
GBADC051-10|10PROBE-21171|Emblyna manitoba
99
SPIRU991-11|BIOUG00627-B07|Emblyna manitoba
SPIRU992-11|BIOUG00627-B08|Emblyna manitoba
Emblyna manitoba
SPICH843-09|09PROBE-600|Emblyna peragrata
SPICH1055-09|09PROBE-834|Emblyna peragrata
99
SPICH844-09|09PROBE-601|Emblyna peragrata
SPICH1043-09|09PROBE-822|Emblyna peragrata
SPICH1054-09|09PROBE-833|Emblyna peragrata
Emblyna peragrata
KKCHE334-07|CHU-SPI-131|Dictyna brevitarsa
JDTGS034-09|09-PROBE-08130|Dictyna brevitarsa
99
SPICH1045-09|09PROBE-824|Dictyna brevitarsa
KKCHE116-06|CHU-SPI-007|Dictyna brevitarsa
SWSWE017-09|09PROBE-01587|Dictyna brevitarsa
SPICH165-09|09PROBE-1711-05|Dictyna brevitarsa
SPICH161-09|09PROBE-1711-01|Dictyna brevitarsa
KKCHE486-07|CHU-SPI-283|Dictyna brevitarsa
ERSCH013-07|07PROBE-04558|Dictyna brevitarsa
KKCHE119-06|CHU-SPI-010|Dictyna brevitarsa
ERSCH076-07|07PROBE-04621|Dictyna brevitarsa
SPICH1044-09|09PROBE-823|Dictyna brevitarsa
SPICH1052-09|09PROBE-831|Dictyna brevitarsa
SPIRU1079-11|BIOUG00628-A12|Dictyna brevitarsa
JDTGS043-09|09-PROBE-08139|Dictyna brevitarsa
GBADC009-10|10PROBE-21129|Dictyna brevitarsa
SPICH1053-09|09PROBE-832|Dictyna brevitarsa
JDTGS032-09|09-PROBE-08128|Dictyna brevitarsa
SPIRU1183-11|BIOUG00629-B09|Dictyna brevitarsa
SPIRU974-11|BIOUG00627-A02|Dictyna brevitarsa
KKCHE212-06|CHU-SPI-103|Dictyna brevitarsa
SPICH1042-09|09PROBE-821|Dictyna brevitarsa
SPICH164-09|09PROBE-1711-04|Dictyna brevitarsa
SPICH1041-09|09PROBE-820|Dictyna brevitarsa
SPICH1049-09|09PROBE-828|Dictyna brevitarsa
Dictyna brevitarsa

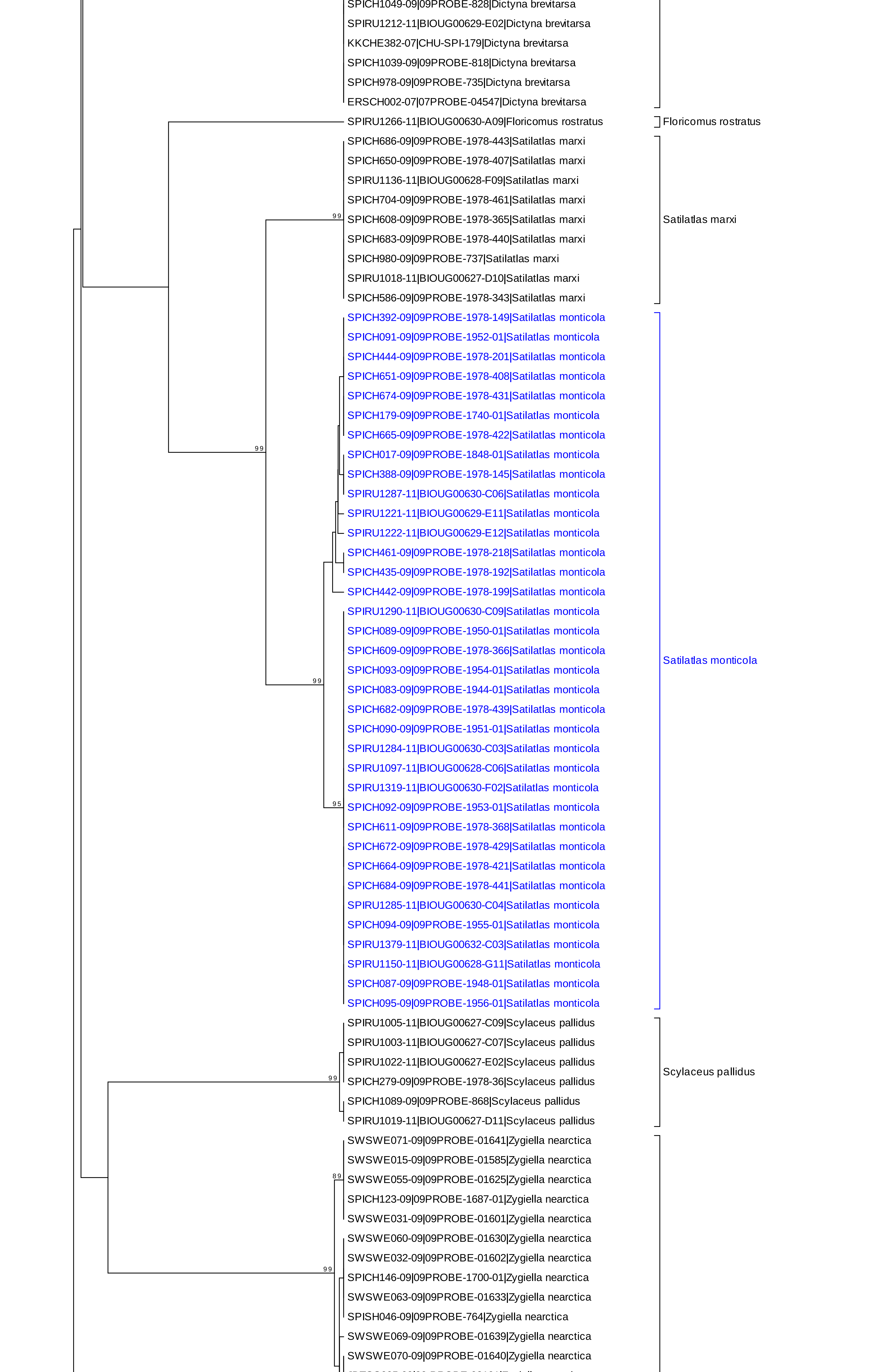
SPICH1049-09|09PROBE-828|Dictyna brevitarsa
SPIRU1212-11|BIOUG00629-E02|Dictyna brevitarsa
KKCHE382-07|CHU-SPI-179|Dictyna brevitarsa
SPICH1039-09|09PROBE-818|Dictyna brevitarsa
SPICH978-09|09PROBE-735|Dictyna brevitarsa
ERSCH002-07|07PROBE-04547|Dictyna brevitarsa
SPIRU1266-11|BIOUG00630-A09|Floricomus rostratus
Floricomus rostratus
SPICH686-09|09PROBE-1978-443|Satilatlas marxi
SPICH650-09|09PROBE-1978-407|Satilatlas marxi
SPIRU1136-11|BIOUG00628-F09|Satilatlas marxi
SPICH704-09|09PROBE-1978-461|Satilatlas marxi
99
SPICH608-09|09PROBE-1978-365|Satilatlas marxi
Satilatlas marxi
SPICH683-09|09PROBE-1978-440|Satilatlas marxi
SPICH980-09|09PROBE-737|Satilatlas marxi
SPIRU1018-11|BIOUG00627-D10|Satilatlas marxi
SPICH586-09|09PROBE-1978-343|Satilatlas marxi
SPICH392-09|09PROBE-1978-149|Satilatlas monticola
SPICH091-09|09PROBE-1952-01|Satilatlas monticola
SPICH444-09|09PROBE-1978-201|Satilatlas monticola
SPICH651-09|09PROBE-1978-408|Satilatlas monticola
SPICH674-09|09PROBE-1978-431|Satilatlas monticola
SPICH179-09|09PROBE-1740-01|Satilatlas monticola
SPICH665-09|09PROBE-1978-422|Satilatlas monticola
99
SPICH017-09|09PROBE-1848-01|Satilatlas monticola
SPICH388-09|09PROBE-1978-145|Satilatlas monticola
SPIRU1287-11|BIOUG00630-C06|Satilatlas monticola
SPIRU1221-11|BIOUG00629-E11|Satilatlas monticola
SPIRU1222-11|BIOUG00629-E12|Satilatlas monticola
SPICH461-09|09PROBE-1978-218|Satilatlas monticola
SPICH435-09|09PROBE-1978-192|Satilatlas monticola
SPICH442-09|09PROBE-1978-199|Satilatlas monticola
SPIRU1290-11|BIOUG00630-C09|Satilatlas monticola
SPICH089-09|09PROBE-1950-01|Satilatlas monticola
SPICH609-09|09PROBE-1978-366|Satilatlas monticola
Satilatlas monticola
SPICH093-09|09PROBE-1954-01|Satilatlas monticola
99
SPICH083-09|09PROBE-1944-01|Satilatlas monticola
SPICH682-09|09PROBE-1978-439|Satilatlas monticola
SPICH090-09|09PROBE-1951-01|Satilatlas monticola
SPIRU1284-11|BIOUG00630-C03|Satilatlas monticola
SPIRU1097-11|BIOUG00628-C06|Satilatlas monticola
SPIRU1319-11|BIOUG00630-F02|Satilatlas monticola
95
SPICH092-09|09PROBE-1953-01|Satilatlas monticola
SPICH611-09|09PROBE-1978-368|Satilatlas monticola
SPICH672-09|09PROBE-1978-429|Satilatlas monticola
SPICH664-09|09PROBE-1978-421|Satilatlas monticola
SPICH684-09|09PROBE-1978-441|Satilatlas monticola
SPIRU1285-11|BIOUG00630-C04|Satilatlas monticola
SPICH094-09|09PROBE-1955-01|Satilatlas monticola
SPIRU1379-11|BIOUG00632-C03|Satilatlas monticola
SPIRU1150-11|BIOUG00628-G11|Satilatlas monticola
SPICH087-09|09PROBE-1948-01|Satilatlas monticola
SPICH095-09|09PROBE-1956-01|Satilatlas monticola
SPIRU1005-11|BIOUG00627-C09|Scylaceus pallidus
SPIRU1003-11|BIOUG00627-C07|Scylaceus pallidus
SPIRU1022-11|BIOUG00627-E02|Scylaceus pallidus
Scylaceus pallidus
99
SPICH279-09|09PROBE-1978-36|Scylaceus pallidus
SPICH1089-09|09PROBE-868|Scylaceus pallidus
SPIRU1019-11|BIOUG00627-D11|Scylaceus pallidus
SWSWE071-09|09PROBE-01641|Zygiella nearctica
SWSWE015-09|09PROBE-01585|Zygiella nearctica
89
SWSWE055-09|09PROBE-01625|Zygiella nearctica
SPICH123-09|09PROBE-1687-01|Zygiella nearctica
SWSWE031-09|09PROBE-01601|Zygiella nearctica
SWSWE060-09|09PROBE-01630|Zygiella nearctica
SWSWE032-09|09PROBE-01602|Zygiella nearctica
99
SPICH146-09|09PROBE-1700-01|Zygiella nearctica
SWSWE063-09|09PROBE-01633|Zygiella nearctica
SPISH046-09|09PROBE-764|Zygiella nearctica
SWSWE069-09|09PROBE-01639|Zygiella nearctica
SWSWE070-09|09PROBE-01640|Zygiella nearctica

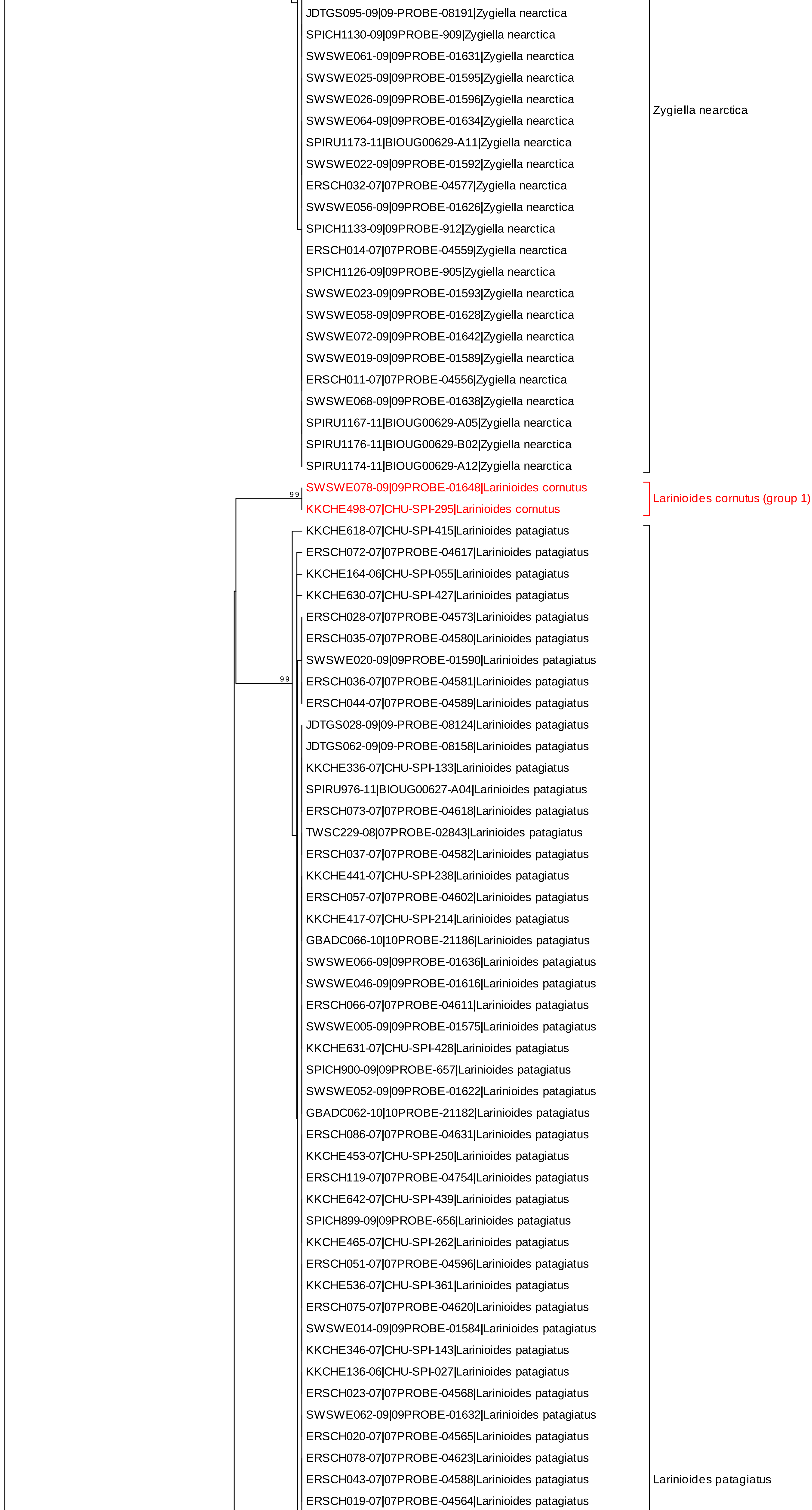
JDTGS095-09|09-PROBE-08191|Zygiella nearctica
SPICH1130-09|09PROBE-909|Zygiella nearctica
SWSWE061-09|09PROBE-01631|Zygiella nearctica
SWSWE025-09|09PROBE-01595|Zygiella nearctica
SWSWE026-09|09PROBE-01596|Zygiella nearctica
SWSWE064-09|09PROBE-01634|Zygiella nearctica
SPIRU1173-11|BIOUG00629-A11|Zygiella nearctica
SWSWE022-09|09PROBE-01592|Zygiella nearctica
ERSCH032-07|07PROBE-04577|Zygiella nearctica
SWSWE056-09|09PROBE-01626|Zygiella nearctica
SPICH1133-09|09PROBE-912|Zygiella nearctica
ERSCH014-07|07PROBE-04559|Zygiella nearctica
SPICH1126-09|09PROBE-905|Zygiella nearctica
SWSWE023-09|09PROBE-01593|Zygiella nearctica
SWSWE058-09|09PROBE-01628|Zygiella nearctica
SWSWE072-09|09PROBE-01642|Zygiella nearctica
SWSWE019-09|09PROBE-01589|Zygiella nearctica
ERSCH011-07|07PROBE-04556|Zygiella nearctica
SWSWE068-09|09PROBE-01638|Zygiella nearctica
SPIRU1167-11|BIOUG00629-A05|Zygiella nearctica
SPIRU1176-11|BIOUG00629-B02|Zygiella nearctica
SPIRU1174-11|BIOUG00629-A12|Zygiella nearctica
Zygiella nearctica
99
SWSWE078-09|09PROBE-01648|Larinioides cornutus
KKCHE498-07|CHU-SPI-295|Larinioides cornutus
Larinioides cornutus (group 1)
99
KKCHE618-07|CHU-SPI-415|Larinioides patagiatus
ERSCH072-07|07PROBE-04617|Larinioides patagiatus
KKCHE164-06|CHU-SPI-055|Larinioides patagiatus
KKCHE630-07|CHU-SPI-427|Larinioides patagiatus
ERSCH028-07|07PROBE-04573|Larinioides patagiatus
ERSCH035-07|07PROBE-04580|Larinioides patagiatus
SWSWE020-09|09PROBE-01590|Larinioides patagiatus
ERSCH036-07|07PROBE-04581|Larinioides patagiatus
ERSCH044-07|07PROBE-04589|Larinioides patagiatus
JDTGS028-09|09-PROBE-08124|Larinioides patagiatus
JDTGS062-09|09-PROBE-08158|Larinioides patagiatus
KKCHE336-07|CHU-SPI-133|Larinioides patagiatus
SPIRU976-11|BIOUG00627-A04|Larinioides patagiatus
ERSCH073-07|07PROBE-04618|Larinioides patagiatus
TWSC229-08|07PROBE-02843|Larinioides patagiatus
ERSCH037-07|07PROBE-04582|Larinioides patagiatus
KKCHE441-07|CHU-SPI-238|Larinioides patagiatus
ERSCH057-07|07PROBE-04602|Larinioides patagiatus
KKCHE417-07|CHU-SPI-214|Larinioides patagiatus
GBADC066-10|10PROBE-21186|Larinioides patagiatus
SWSWE066-09|09PROBE-01636|Larinioides patagiatus
SWSWE046-09|09PROBE-01616|Larinioides patagiatus
ERSCH066-07|07PROBE-04611|Larinioides patagiatus
SWSWE005-09|09PROBE-01575|Larinioides patagiatus
KKCHE631-07|CHU-SPI-428|Larinioides patagiatus
SPICH900-09|09PROBE-657|Larinioides patagiatus
SWSWE052-09|09PROBE-01622|Larinioides patagiatus
GBADC062-10|10PROBE-21182|Larinioides patagiatus
ERSCH086-07|07PROBE-04631|Larinioides patagiatus
KKCHE453-07|CHU-SPI-250|Larinioides patagiatus
ERSCH119-07|07PROBE-04754|Larinioides patagiatus
KKCHE642-07|CHU-SPI-439|Larinioides patagiatus
SPICH899-09|09PROBE-656|Larinioides patagiatus
KKCHE465-07|CHU-SPI-262|Larinioides patagiatus
ERSCH051-07|07PROBE-04596|Larinioides patagiatus
KKCHE536-07|CHU-SPI-361|Larinioides patagiatus
ERSCH075-07|07PROBE-04620|Larinioides patagiatus
SWSWE014-09|09PROBE-01584|Larinioides patagiatus
KKCHE346-07|CHU-SPI-143|Larinioides patagiatus
KKCHE136-06|CHU-SPI-027|Larinioides patagiatus
ERSCH023-07|07PROBE-04568|Larinioides patagiatus
SWSWE062-09|09PROBE-01632|Larinioides patagiatus
ERSCH020-07|07PROBE-04565|Larinioides patagiatus
ERSCH078-07|07PROBE-04623|Larinioides patagiatus
ERSCH043-07|07PROBE-04588|Larinioides patagiatus
ERSCH019-07|07PROBE-04564|Larinioides patagiatus
Larinioides patagiatus

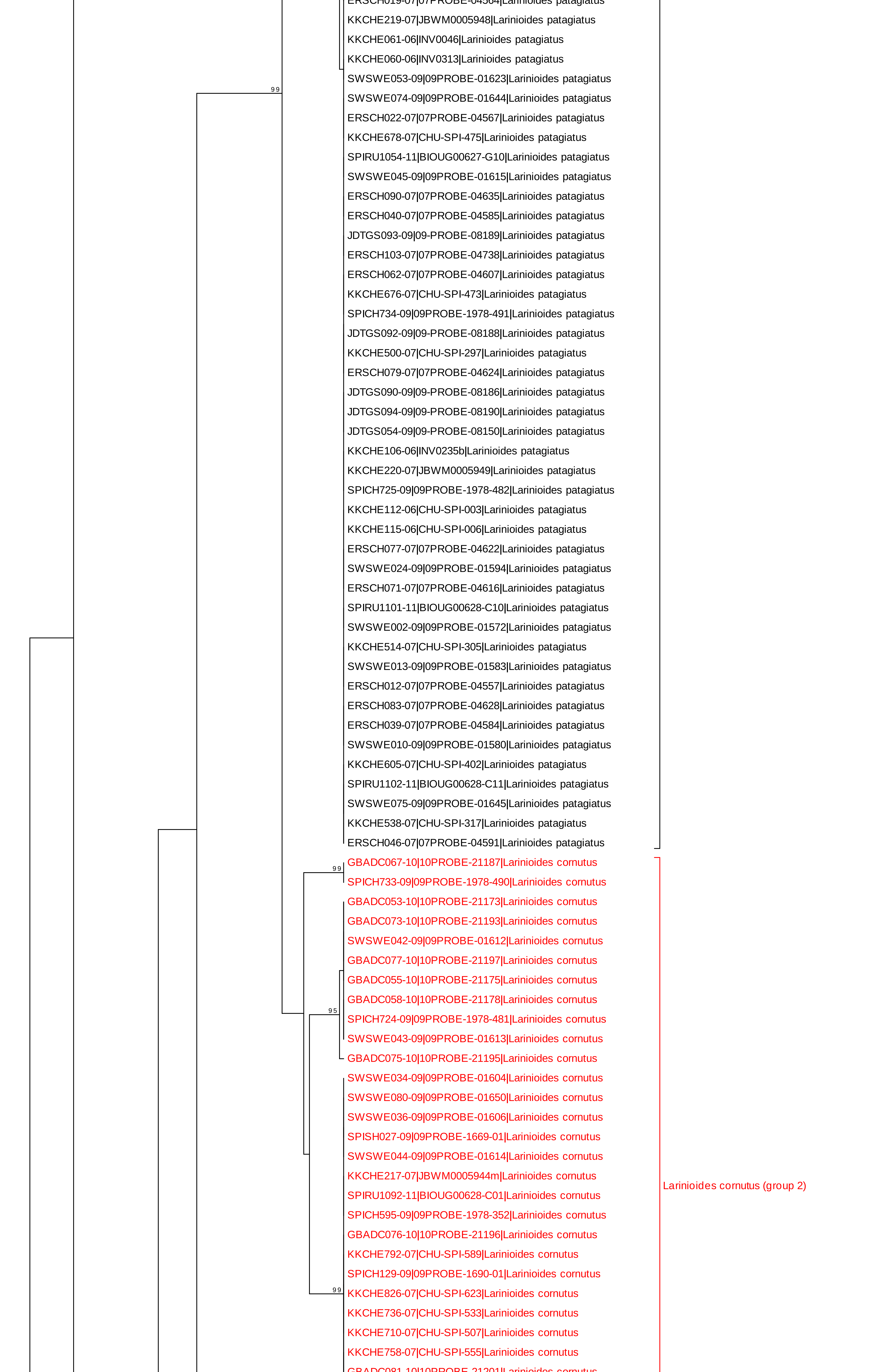
ERSCH019-07|07PROBE-04564|Larinioides patagiatus
KKCHE219-07|JBWM0005948|Larinioides patagiatus
KKCHE061-06|INV0046|Larinioides patagiatus
KKCHE060-06|INV0313|Larinioides patagiatus
99
SWSWE053-09|09PROBE-01623|Larinioides patagiatus
SWSWE074-09|09PROBE-01644|Larinioides patagiatus
ERSCH022-07|07PROBE-04567|Larinioides patagiatus
KKCHE678-07|CHU-SPI-475|Larinioides patagiatus
SPIRU1054-11|BIOUG00627-G10|Larinioides patagiatus
SWSWE045-09|09PROBE-01615|Larinioides patagiatus
ERSCH090-07|07PROBE-04635|Larinioides patagiatus
ERSCH040-07|07PROBE-04585|Larinioides patagiatus
JDTGS093-09|09-PROBE-08189|Larinioides patagiatus
ERSCH103-07|07PROBE-04738|Larinioides patagiatus
ERSCH062-07|07PROBE-04607|Larinioides patagiatus
KKCHE676-07|CHU-SPI-473|Larinioides patagiatus
SPICH734-09|09PROBE-1978-491|Larinioides patagiatus
JDTGS092-09|09-PROBE-08188|Larinioides patagiatus
KKCHE500-07|CHU-SPI-297|Larinioides patagiatus
ERSCH079-07|07PROBE-04624|Larinioides patagiatus
JDTGS090-09|09-PROBE-08186|Larinioides patagiatus
JDTGS094-09|09-PROBE-08190|Larinioides patagiatus
JDTGS054-09|09-PROBE-08150|Larinioides patagiatus
KKCHE106-06|INV0235b|Larinioides patagiatus
KKCHE220-07|JBWM0005949|Larinioides patagiatus
SPICH725-09|09PROBE-1978-482|Larinioides patagiatus
KKCHE112-06|CHU-SPI-003|Larinioides patagiatus
KKCHE115-06|CHU-SPI-006|Larinioides patagiatus
ERSCH077-07|07PROBE-04622|Larinioides patagiatus
SWSWE024-09|09PROBE-01594|Larinioides patagiatus
ERSCH071-07|07PROBE-04616|Larinioides patagiatus
SPIRU1101-11|BIOUG00628-C10|Larinioides patagiatus
SWSWE002-09|09PROBE-01572|Larinioides patagiatus
KKCHE514-07|CHU-SPI-305|Larinioides patagiatus
SWSWE013-09|09PROBE-01583|Larinioides patagiatus
ERSCH012-07|07PROBE-04557|Larinioides patagiatus
ERSCH083-07|07PROBE-04628|Larinioides patagiatus
ERSCH039-07|07PROBE-04584|Larinioides patagiatus
SWSWE010-09|09PROBE-01580|Larinioides patagiatus
KKCHE605-07|CHU-SPI-402|Larinioides patagiatus
SPIRU1102-11|BIOUG00628-C11|Larinioides patagiatus
SWSWE075-09|09PROBE-01645|Larinioides patagiatus
KKCHE538-07|CHU-SPI-317|Larinioides patagiatus
ERSCH046-07|07PROBE-04591|Larinioides patagiatus
99
GBADC067-10|10PROBE-21187|Larinioides cornutus
SPICH733-09|09PROBE-1978-490|Larinioides cornutus
GBADC053-10|10PROBE-21173|Larinioides cornutus
GBADC073-10|10PROBE-21193|Larinioides cornutus
SWSWE042-09|09PROBE-01612|Larinioides cornutus
GBADC077-10|10PROBE-21197|Larinioides cornutus
GBADC055-10|10PROBE-21175|Larinioides cornutus
GBADC058-10|10PROBE-21178|Larinioides cornutus
95
SPICH724-09|09PROBE-1978-481|Larinioides cornutus
SWSWE043-09|09PROBE-01613|Larinioides cornutus
GBADC075-10|10PROBE-21195|Larinioides cornutus
SWSWE034-09|09PROBE-01604|Larinioides cornutus
SWSWE080-09|09PROBE-01650|Larinioides cornutus
SWSWE036-09|09PROBE-01606|Larinioides cornutus
SPISH027-09|09PROBE-1669-01|Larinioides cornutus
SWSWE044-09|09PROBE-01614|Larinioides cornutus
KKCHE217-07|JBWM0005944m|Larinioides cornutus
Larinioides cornutus (group 2)
SPIRU1092-11|BIOUG00628-C01|Larinioides cornutus
SPICH595-09|09PROBE-1978-352|Larinioides cornutus
GBADC076-10|10PROBE-21196|Larinioides cornutus
KKCHE792-07|CHU-SPI-589|Larinioides cornutus
SPICH129-09|09PROBE-1690-01|Larinioides cornutus
99
KKCHE826-07|CHU-SPI-623|Larinioides cornutus
KKCHE736-07|CHU-SPI-533|Larinioides cornutus
KKCHE710-07|CHU-SPI-507|Larinioides cornutus
KKCHE758-07|CHU-SPI-555|Larinioides cornutus

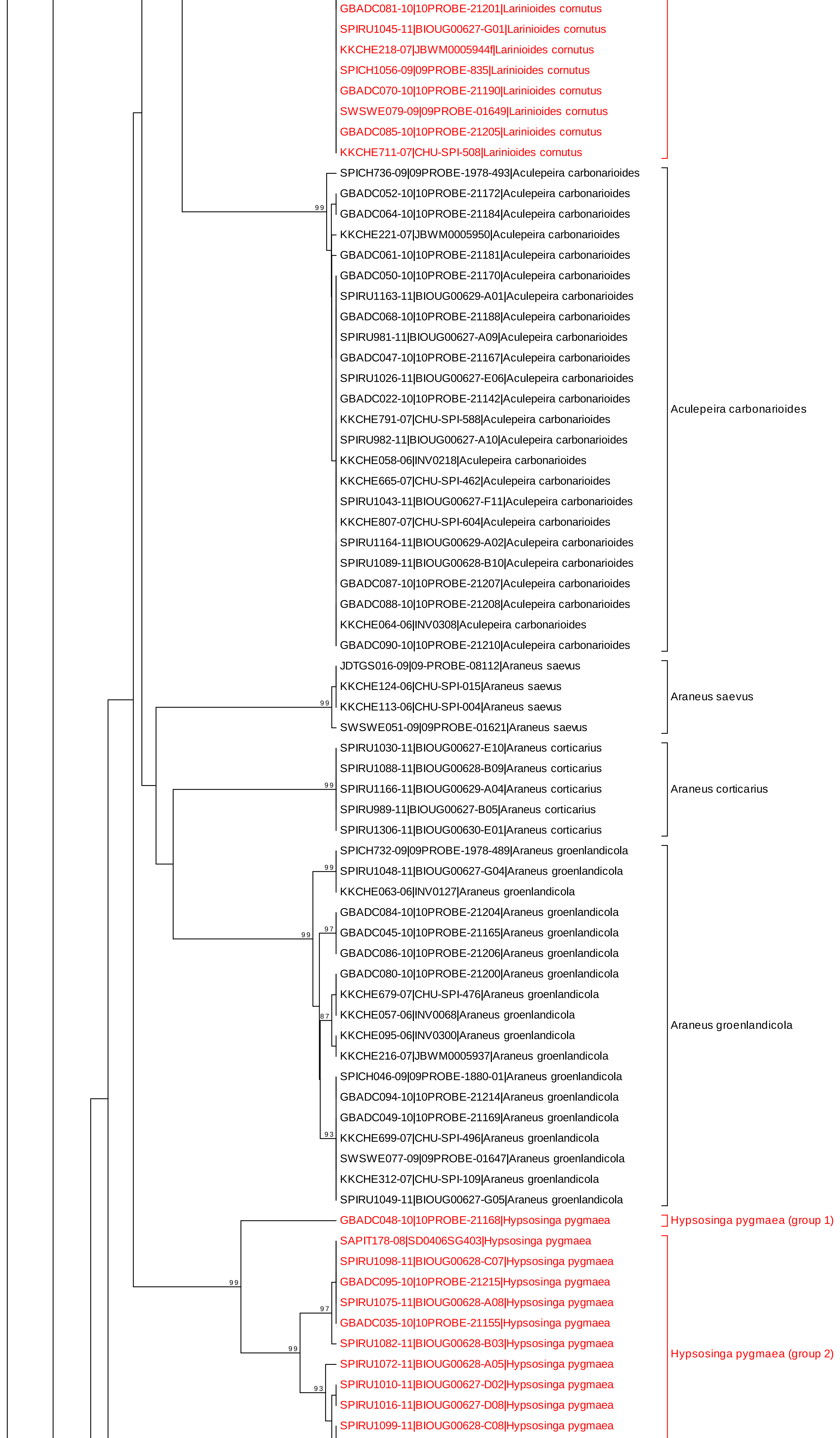
GBADC081-10|10PROBE-21201|Larinioides cornutus
SPIRU1045-11|BIOUG00627-G01|Larinioides cornutus
KKCHE218-07|JBWM0005944f|Larinioides cornutus
SPICH1056-09|09PROBE-835|Larinioides cornutus
GBADC070-10|10PROBE-21190|Larinioides cornutus
SWSWE079-09|09PROBE-01649|Larinioides cornutus
GBADC085-10|10PROBE-21205|Larinioides cornutus
KKCHE711-07|CHU-SPI-508|Larinioides cornutus
SPICH736-09|09PROBE-1978-493|Aculepeira carbonarioides
99
GBADC052-10|10PROBE-21172|Aculepeira carbonarioides
GBADC064-10|10PROBE-21184|Aculepeira carbonarioides
KKCHE221-07|JBWM0005950|Aculepeira carbonarioides
GBADC061-10|10PROBE-21181|Aculepeira carbonarioides
GBADC050-10|10PROBE-21170|Aculepeira carbonarioides
SPIRU1163-11|BIOUG00629-A01|Aculepeira carbonarioides
GBADC068-10|10PROBE-21188|Aculepeira carbonarioides
SPIRU981-11|BIOUG00627-A09|Aculepeira carbonarioides
GBADC047-10|10PROBE-21167|Aculepeira carbonarioides
SPIRU1026-11|BIOUG00627-E06|Aculepeira carbonarioides
GBADC022-10|10PROBE-21142|Aculepeira carbonarioides
KKCHE791-07|CHU-SPI-588|Aculepeira carbonarioides
SPIRU982-11|BIOUG00627-A10|Aculepeira carbonarioides
KKCHE058-06|INV0218|Aculepeira carbonarioides
KKCHE665-07|CHU-SPI-462|Aculepeira carbonarioides
SPIRU1043-11|BIOUG00627-F11|Aculepeira carbonarioides
KKCHE807-07|CHU-SPI-604|Aculepeira carbonarioides
SPIRU1164-11|BIOUG00629-A02|Aculepeira carbonarioides
SPIRU1089-11|BIOUG00628-B10|Aculepeira carbonarioides
GBADC087-10|10PROBE-21207|Aculepeira carbonarioides
GBADC088-10|10PROBE-21208|Aculepeira carbonarioides
KKCHE064-06|INV0308|Aculepeira carbonarioides
GBADC090-10|10PROBE-21210|Aculepeira carbonarioides
Aculepeira carbonarioides
JDTGS016-09|09-PROBE-08112|Araneus saevus
KKCHE124-06|CHU-SPI-015|Araneus saevus
99
KKCHE113-06|CHU-SPI-004|Araneus saevus
SWSWE051-09|09PROBE-01621|Araneus saevus
Araneus saevus
SPIRU1030-11|BIOUG00627-E10|Araneus corticarius
SPIRU1088-11|BIOUG00628-B09|Araneus corticarius
99
SPIRU1166-11|BIOUG00629-A04|Araneus corticarius
SPIRU989-11|BIOUG00627-B05|Araneus corticarius
SPIRU1306-11|BIOUG00630-E01|Araneus corticarius
Araneus corticarius
SPICH732-09|09PROBE-1978-489|Araneus groenlandicola
99
SPIRU1048-11|BIOUG00627-G04|Araneus groenlandicola
KKCHE063-06|INV0127|Araneus groenlandicola
GBADC084-10|10PROBE-21204|Araneus groenlandicola
97
GBADC045-10|10PROBE-21165|Araneus groenlandicola
99
GBADC086-10|10PROBE-21206|Araneus groenlandicola
GBADC080-10|10PROBE-21200|Araneus groenlandicola
KKCHE679-07|CHU-SPI-476|Araneus groenlandicola
87
KKCHE057-06|INV0068|Araneus groenlandicola
KKCHE095-06|INV0300|Araneus groenlandicola
KKCHE216-07|JBWM0005937|Araneus groenlandicola
SPICH046-09|09PROBE-1880-01|Araneus groenlandicola
GBADC094-10|10PROBE-21214|Araneus groenlandicola
GBADC049-10|10PROBE-21169|Araneus groenlandicola
93
KKCHE699-07|CHU-SPI-496|Araneus groenlandicola
SWSWE077-09|09PROBE-01647|Araneus groenlandicola
KKCHE312-07|CHU-SPI-109|Araneus groenlandicola
SPIRU1049-11|BIOUG00627-G05|Araneus groenlandicola
Araneus groenlandicola
GBADC048-10|10PROBE-21168|Hypsosinga pygmaea
Hypsosinga pygmaea (group 1)
SAPIT178-08|SD0406SG403|Hypsosinga pygmaea
SPIRU1098-11|BIOUG00628-C07|Hypsosinga pygmaea
99
GBADC095-10|10PROBE-21215|Hypsosinga pygmaea
97
SPIRU1075-11|BIOUG00628-A08|Hypsosinga pygmaea
GBADC035-10|10PROBE-21155|Hypsosinga pygmaea
99
SPIRU1082-11|BIOUG00628-B03|Hypsosinga pygmaea
SPIRU1072-11|BIOUG00628-A05|Hypsosinga pygmaea
93
SPIRU1010-11|BIOUG00627-D02|Hypsosinga pygmaea
SPIRU1016-11|BIOUG00627-D08|Hypsosinga pygmaea
SPIRU1099-11|BIOUG00628-C08|Hypsosinga pygmaea
Hypsosinga pygmaea (group 2)

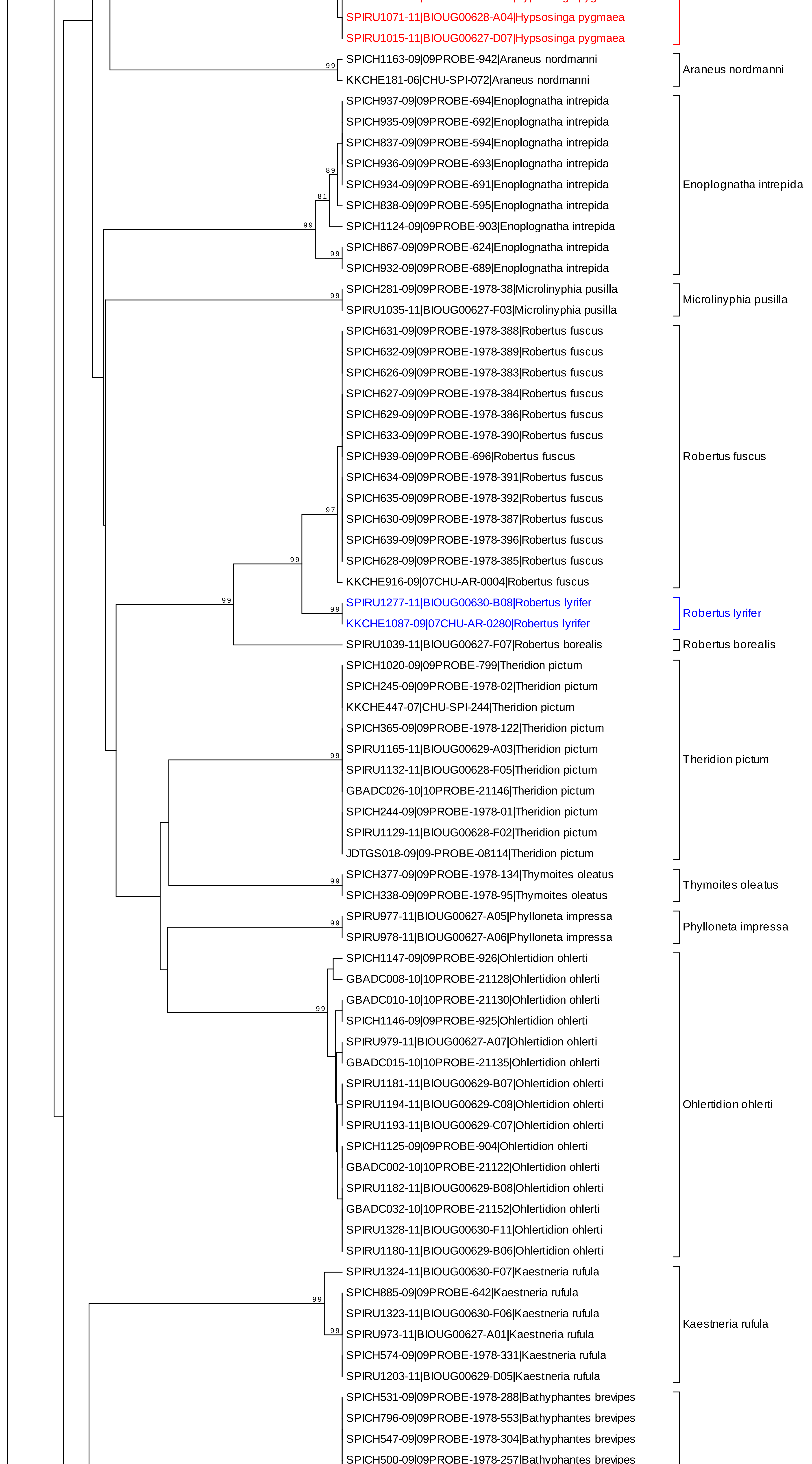

SPIRU1071-11|BIOUG00628-A04|Hypsosinga pygmaea
SPIRU1015-11|BIOUG00627-D07|Hypsosinga pygmaea
SPICH1163-09|09PROBE-942|Araneus nordmanni
KKCHE181-06|CHU-SPI-072|Araneus nordmanni
Araneus nordmanni
SPICH937-09|09PROBE-694|Enoplognatha intrepida
SPICH935-09|09PROBE-692|Enoplognatha intrepida
SPICH837-09|09PROBE-594|Enoplognatha intrepida
SPICH936-09|09PROBE-693|Enoplognatha intrepida
SPICH934-09|09PROBE-691|Enoplognatha intrepida
SPICH838-09|09PROBE-595|Enoplognatha intrepida
SPICH1124-09|09PROBE-903|Enoplognatha intrepida
SPICH867-09|09PROBE-624|Enoplognatha intrepida
SPICH932-09|09PROBE-689|Enoplognatha intrepida
Enoplognatha intrepida
SPICH281-09|09PROBE-1978-38|Microlinyphia pusilla
SPIRU1035-11|BIOUG00627-F03|Microlinyphia pusilla
Microlinyphia pusilla
SPICH631-09|09PROBE-1978-388|Robertus fuscus
SPICH632-09|09PROBE-1978-389|Robertus fuscus
SPICH626-09|09PROBE-1978-383|Robertus fuscus
SPICH627-09|09PROBE-1978-384|Robertus fuscus
SPICH629-09|09PROBE-1978-386|Robertus fuscus
SPICH633-09|09PROBE-1978-390|Robertus fuscus
SPICH939-09|09PROBE-696|Robertus fuscus
SPICH634-09|09PROBE-1978-391|Robertus fuscus
SPICH635-09|09PROBE-1978-392|Robertus fuscus
SPICH630-09|09PROBE-1978-387|Robertus fuscus
SPICH639-09|09PROBE-1978-396|Robertus fuscus
SPICH628-09|09PROBE-1978-385|Robertus fuscus
KKCHE916-09|07CHU-AR-0004|Robertus fuscus
Robertus fuscus
SPIRU1277-11|BIOUG00630-B08|Robertus lyrifer
KKCHE1087-09|07CHU-AR-0280|Robertus lyrifer
Robertus lyrifer
SPIRU1039-11|BIOUG00627-F07|Robertus borealis
Robertus borealis
SPICH1020-09|09PROBE-799|Theridion pictum
SPICH245-09|09PROBE-1978-02|Theridion pictum
KKCHE447-07|CHU-SPI-244|Theridion pictum
SPICH365-09|09PROBE-1978-122|Theridion pictum
SPIRU1165-11|BIOUG00629-A03|Theridion pictum
SPIRU1132-11|BIOUG00628-F05|Theridion pictum
GBADC026-10|10PROBE-21146|Theridion pictum
SPICH244-09|09PROBE-1978-01|Theridion pictum
SPIRU1129-11|BIOUG00628-F02|Theridion pictum
JDTGS018-09|09-PROBE-08114|Theridion pictum
Theridion pictum
SPICH377-09|09PROBE-1978-134|Thymoites oleatus
SPICH338-09|09PROBE-1978-95|Thymoites oleatus
Thymoites oleatus
SPIRU977-11|BIOUG00627-A05|Phylloneta impressa
SPIRU978-11|BIOUG00627-A06|Phylloneta impressa
Phylloneta impressa
SPICH1147-09|09PROBE-926|Ohlertidion ohlerti
GBADC008-10|10PROBE-21128|Ohlertidion ohlerti
GBADC010-10|10PROBE-21130|Ohlertidion ohlerti
SPICH1146-09|09PROBE-925|Ohlertidion ohlerti
SPIRU979-11|BIOUG00627-A07|Ohlertidion ohlerti
GBADC015-10|10PROBE-21135|Ohlertidion ohlerti
SPIRU1181-11|BIOUG00629-B07|Ohlertidion ohlerti
SPIRU1194-11|BIOUG00629-C08|Ohlertidion ohlerti
SPIRU1193-11|BIOUG00629-C07|Ohlertidion ohlerti
SPICH1125-09|09PROBE-904|Ohlertidion ohlerti
GBADC002-10|10PROBE-21122|Ohlertidion ohlerti
SPIRU1182-11|BIOUG00629-B08|Ohlertidion ohlerti
GBADC032-10|10PROBE-21152|Ohlertidion ohlerti
SPIRU1328-11|BIOUG00630-F11|Ohlertidion ohlerti
SPIRU1180-11|BIOUG00629-B06|Ohlertidion ohlerti
Ohlertidion ohlerti
SPIRU1324-11|BIOUG00630-F07|Kaestneria rufula
SPICH885-09|09PROBE-642|Kaestneria rufula
SPIRU1323-11|BIOUG00630-F06|Kaestneria rufula
SPIRU973-11|BIOUG00627-A01|Kaestneria rufula
SPICH574-09|09PROBE-1978-331|Kaestneria rufula
SPIRU1203-11|BIOUG00629-D05|Kaestneria rufula
Kaestneria rufula
SPICH531-09|09PROBE-1978-288|Bathyphantes brevipes
SPICH796-09|09PROBE-1978-553|Bathyphantes brevipes
SPICH547-09|09PROBE-1978-304|Bathyphantes brevipes
SPICH500-09|09PROBE-1978-257|Bathyphantes brevipes

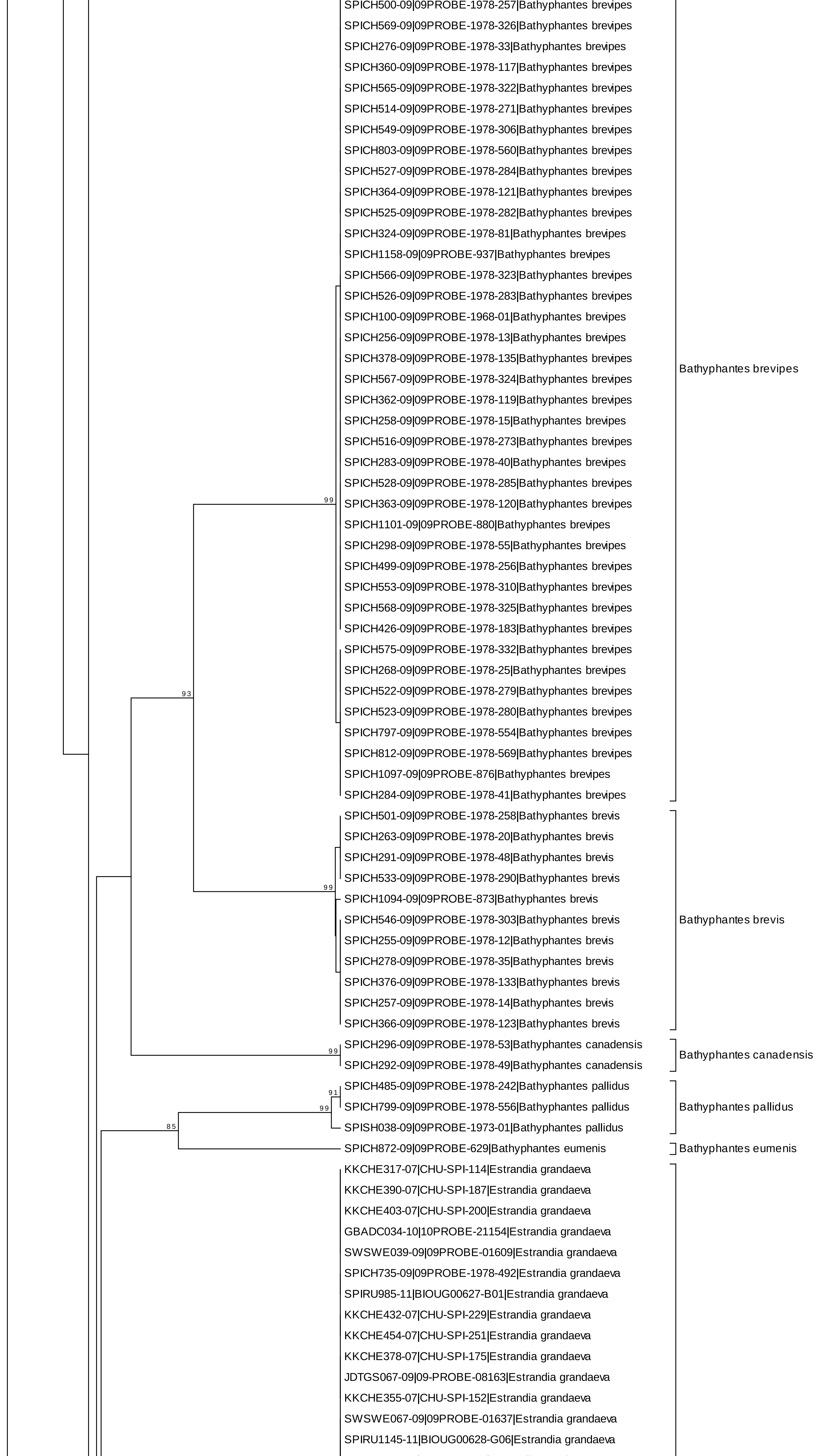
SPICH500-09|09PROBE-1978-257|Bathyphantes brevipes
SPICH569-09|09PROBE-1978-326|Bathyphantes brevipes
SPICH276-09|09PROBE-1978-33|Bathyphantes brevipes
SPICH360-09|09PROBE-1978-117|Bathyphantes brevipes
SPICH565-09|09PROBE-1978-322|Bathyphantes brevipes
SPICH514-09|09PROBE-1978-271|Bathyphantes brevipes
SPICH549-09|09PROBE-1978-306|Bathyphantes brevipes
SPICH803-09|09PROBE-1978-560|Bathyphantes brevipes
SPICH527-09|09PROBE-1978-284|Bathyphantes brevipes
SPICH364-09|09PROBE-1978-121|Bathyphantes brevipes
SPICH525-09|09PROBE-1978-282|Bathyphantes brevipes
SPICH324-09|09PROBE-1978-81|Bathyphantes brevipes
SPICH1158-09|09PROBE-937|Bathyphantes brevipes
SPICH566-09|09PROBE-1978-323|Bathyphantes brevipes
SPICH526-09|09PROBE-1978-283|Bathyphantes brevipes
SPICH100-09|09PROBE-1968-01|Bathyphantes brevipes
SPICH256-09|09PROBE-1978-13|Bathyphantes brevipes
SPICH378-09|09PROBE-1978-135|Bathyphantes brevipes
SPICH567-09|09PROBE-1978-324|Bathyphantes brevipes
SPICH362-09|09PROBE-1978-119|Bathyphantes brevipes
SPICH258-09|09PROBE-1978-15|Bathyphantes brevipes
SPICH516-09|09PROBE-1978-273|Bathyphantes brevipes
SPICH283-09|09PROBE-1978-40|Bathyphantes brevipes
SPICH528-09|09PROBE-1978-285|Bathyphantes brevipes
99
SPICH363-09|09PROBE-1978-120|Bathyphantes brevipes
SPICH1101-09|09PROBE-880|Bathyphantes brevipes
SPICH298-09|09PROBE-1978-55|Bathyphantes brevipes
SPICH499-09|09PROBE-1978-256|Bathyphantes brevipes
SPICH553-09|09PROBE-1978-310|Bathyphantes brevipes
SPICH568-09|09PROBE-1978-325|Bathyphantes brevipes
SPICH426-09|09PROBE-1978-183|Bathyphantes brevipes
SPICH575-09|09PROBE-1978-332|Bathyphantes brevipes
SPICH268-09|09PROBE-1978-25|Bathyphantes brevipes
93
SPICH522-09|09PROBE-1978-279|Bathyphantes brevipes
SPICH523-09|09PROBE-1978-280|Bathyphantes brevipes
SPICH797-09|09PROBE-1978-554|Bathyphantes brevipes
SPICH812-09|09PROBE-1978-569|Bathyphantes brevipes
SPICH1097-09|09PROBE-876|Bathyphantes brevipes
SPICH284-09|09PROBE-1978-41|Bathyphantes brevipes
Bathyphantes brevipes
SPICH501-09|09PROBE-1978-258|Bathyphantes brevis
SPICH263-09|09PROBE-1978-20|Bathyphantes brevis
SPICH291-09|09PROBE-1978-48|Bathyphantes brevis
SPICH533-09|09PROBE-1978-290|Bathyphantes brevis
99
SPICH1094-09|09PROBE-873|Bathyphantes brevis
SPICH546-09|09PROBE-1978-303|Bathyphantes brevis
SPICH255-09|09PROBE-1978-12|Bathyphantes brevis
SPICH278-09|09PROBE-1978-35|Bathyphantes brevis
SPICH376-09|09PROBE-1978-133|Bathyphantes brevis
SPICH257-09|09PROBE-1978-14|Bathyphantes brevis
SPICH366-09|09PROBE-1978-123|Bathyphantes brevis
Bathyphantes brevis
SPICH296-09|09PROBE-1978-53|Bathyphantes canadensis
99
SPICH292-09|09PROBE-1978-49|Bathyphantes canadensis
Bathyphantes canadensis
SPICH485-09|09PROBE-1978-242|Bathyphantes pallidus
91
99
SPICH799-09|09PROBE-1978-556|Bathyphantes pallidus
85
SPISH038-09|09PROBE-1973-01|Bathyphantes pallidus
Bathyphantes pallidus
SPICH872-09|09PROBE-629|Bathyphantes eumenis
Bathyphantes eumenis
KKCHE317-07|CHU-SPI-114|Estrandia grandaeva
KKCHE390-07|CHU-SPI-187|Estrandia grandaeva
KKCHE403-07|CHU-SPI-200|Estrandia grandaeva
GBADC034-10|10PROBE-21154|Estrandia grandaeva
SWSWE039-09|09PROBE-01609|Estrandia grandaeva
SPICH735-09|09PROBE-1978-492|Estrandia grandaeva
SPIRU985-11|BIOUG00627-B01|Estrandia grandaeva
KKCHE432-07|CHU-SPI-229|Estrandia grandaeva
KKCHE454-07|CHU-SPI-251|Estrandia grandaeva
KKCHE378-07|CHU-SPI-175|Estrandia grandaeva
JDTGS067-09|09-PROBE-08163|Estrandia grandaeva
KKCHE355-07|CHU-SPI-152|Estrandia grandaeva
SWSWE067-09|09PROBE-01637|Estrandia grandaeva
SPIRU1145-11|BIOUG00628-G06|Estrandia grandaeva

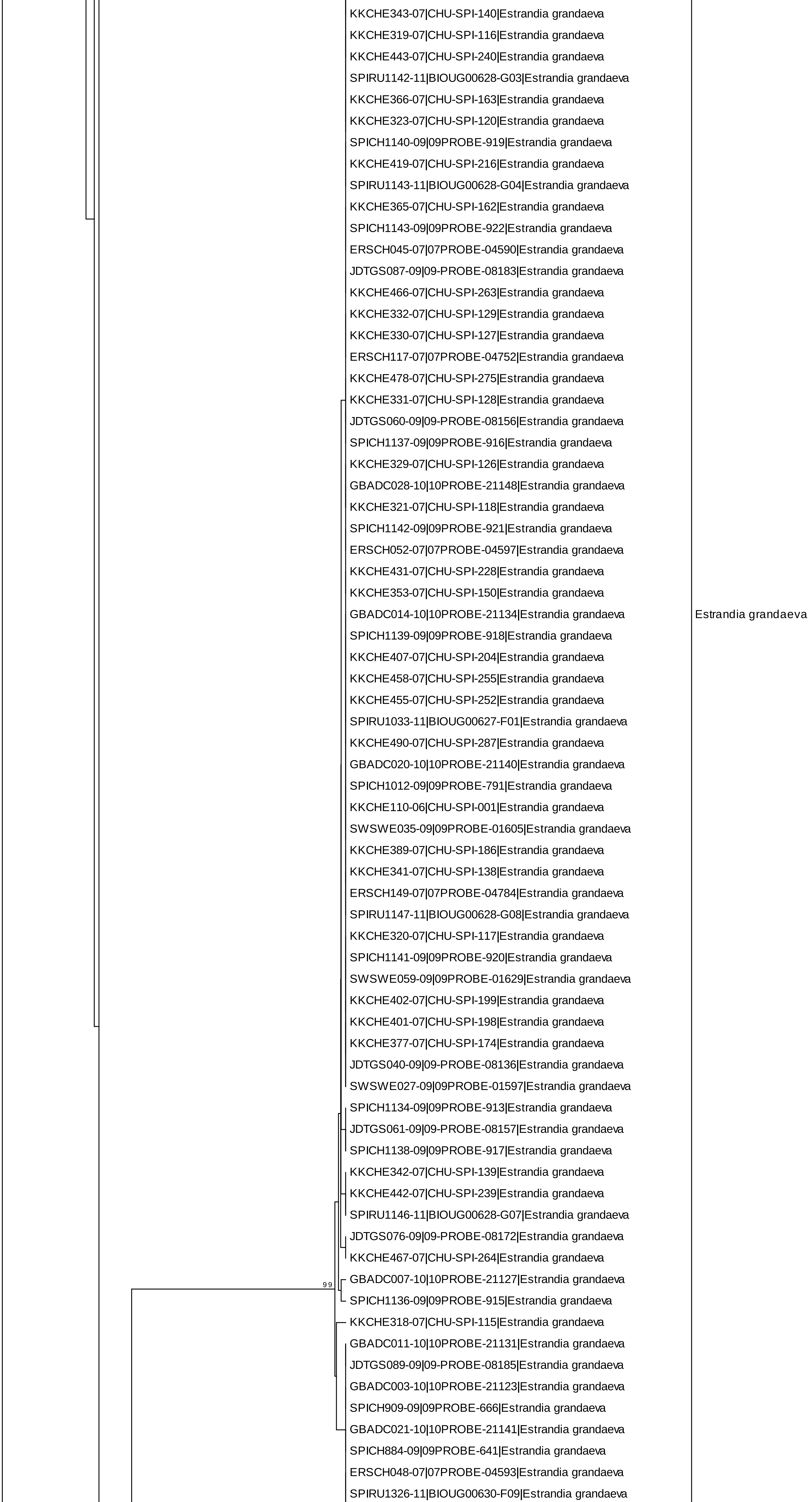
KKCHE343-07|CHU-SPI-140|Estrandia grandaeva
KKCHE319-07|CHU-SPI-116|Estrandia grandaeva
KKCHE443-07|CHU-SPI-240|Estrandia grandaeva
SPIRU1142-11|BIOUG00628-G03|Estrandia grandaeva
KKCHE366-07|CHU-SPI-163|Estrandia grandaeva
KKCHE323-07|CHU-SPI-120|Estrandia grandaeva
SPICH1140-09|09PROBE-919|Estrandia grandaeva
KKCHE419-07|CHU-SPI-216|Estrandia grandaeva
SPIRU1143-11|BIOUG00628-G04|Estrandia grandaeva
KKCHE365-07|CHU-SPI-162|Estrandia grandaeva
SPICH1143-09|09PROBE-922|Estrandia grandaeva
ERSCH045-07|07PROBE-04590|Estrandia grandaeva
JDTGS087-09|09-PROBE-08183|Estrandia grandaeva
KKCHE466-07|CHU-SPI-263|Estrandia grandaeva
KKCHE332-07|CHU-SPI-129|Estrandia grandaeva
KKCHE330-07|CHU-SPI-127|Estrandia grandaeva
ERSCH117-07|07PROBE-04752|Estrandia grandaeva
KKCHE478-07|CHU-SPI-275|Estrandia grandaeva
KKCHE331-07|CHU-SPI-128|Estrandia grandaeva
JDTGS060-09|09-PROBE-08156|Estrandia grandaeva
SPICH1137-09|09PROBE-916|Estrandia grandaeva
KKCHE329-07|CHU-SPI-126|Estrandia grandaeva
GBADC028-10|10PROBE-21148|Estrandia grandaeva
KKCHE321-07|CHU-SPI-118|Estrandia grandaeva
SPICH1142-09|09PROBE-921|Estrandia grandaeva
ERSCH052-07|07PROBE-04597|Estrandia grandaeva
KKCHE431-07|CHU-SPI-228|Estrandia grandaeva
KKCHE353-07|CHU-SPI-150|Estrandia grandaeva
GBADC014-10|10PROBE-21134|Estrandia grandaeva
Estrandia grandaeva
SPICH1139-09|09PROBE-918|Estrandia grandaeva
KKCHE407-07|CHU-SPI-204|Estrandia grandaeva
KKCHE458-07|CHU-SPI-255|Estrandia grandaeva
KKCHE455-07|CHU-SPI-252|Estrandia grandaeva
SPIRU1033-11|BIOUG00627-F01|Estrandia grandaeva
KKCHE490-07|CHU-SPI-287|Estrandia grandaeva
GBADC020-10|10PROBE-21140|Estrandia grandaeva
SPICH1012-09|09PROBE-791|Estrandia grandaeva
KKCHE110-06|CHU-SPI-001|Estrandia grandaeva
SWSWE035-09|09PROBE-01605|Estrandia grandaeva
KKCHE389-07|CHU-SPI-186|Estrandia grandaeva
KKCHE341-07|CHU-SPI-138|Estrandia grandaeva
ERSCH149-07|07PROBE-04784|Estrandia grandaeva
SPIRU1147-11|BIOUG00628-G08|Estrandia grandaeva
KKCHE320-07|CHU-SPI-117|Estrandia grandaeva
SPICH1141-09|09PROBE-920|Estrandia grandaeva
SWSWE059-09|09PROBE-01629|Estrandia grandaeva
KKCHE402-07|CHU-SPI-199|Estrandia grandaeva
KKCHE401-07|CHU-SPI-198|Estrandia grandaeva
KKCHE377-07|CHU-SPI-174|Estrandia grandaeva
JDTGS040-09|09-PROBE-08136|Estrandia grandaeva
SWSWE027-09|09PROBE-01597|Estrandia grandaeva
SPICH1134-09|09PROBE-913|Estrandia grandaeva
JDTGS061-09|09-PROBE-08157|Estrandia grandaeva
SPICH1138-09|09PROBE-917|Estrandia grandaeva
KKCHE342-07|CHU-SPI-139|Estrandia grandaeva
KKCHE442-07|CHU-SPI-239|Estrandia grandaeva
SPIRU1146-11|BIOUG00628-G07|Estrandia grandaeva
JDTGS076-09|09-PROBE-08172|Estrandia grandaeva
KKCHE467-07|CHU-SPI-264|Estrandia grandaeva
99
GBADC007-10|10PROBE-21127|Estrandia grandaeva
SPICH1136-09|09PROBE-915|Estrandia grandaeva
KKCHE318-07|CHU-SPI-115|Estrandia grandaeva
GBADC011-10|10PROBE-21131|Estrandia grandaeva
JDTGS089-09|09-PROBE-08185|Estrandia grandaeva
GBADC003-10|10PROBE-21123|Estrandia grandaeva
SPICH909-09|09PROBE-666|Estrandia grandaeva
GBADC021-10|10PROBE-21141|Estrandia grandaeva
SPICH884-09|09PROBE-641|Estrandia grandaeva
ERSCH048-07|07PROBE-04593|Estrandia grandaeva
SPIRU1326-11|BIOUG00630-F09|Estrandia grandaeva

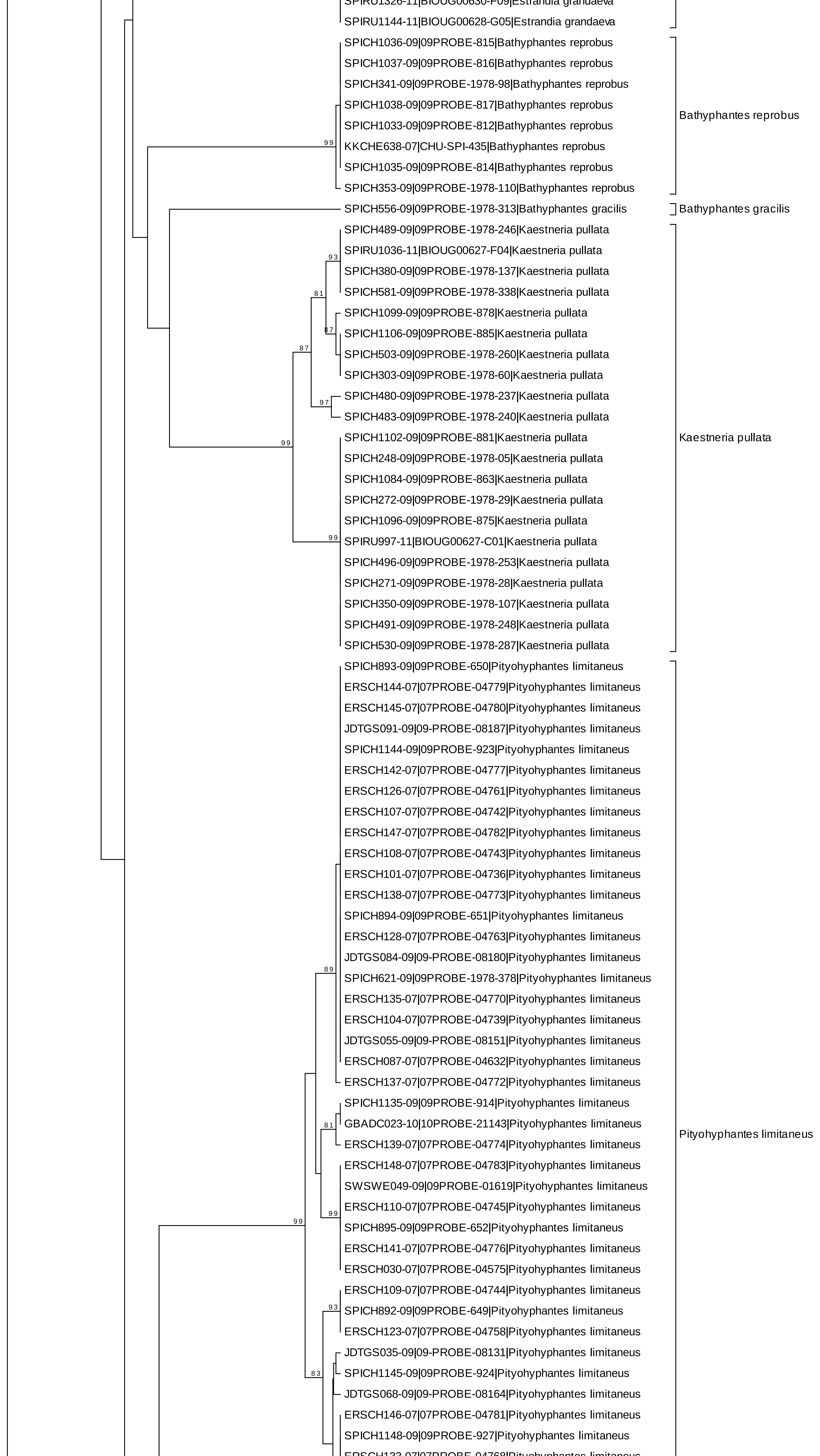
SPIRU1326-11|BIOUG00630-F09|Estrandia grandaeva
SPIRU1144-11|BIOUG00628-G05|Estrandia grandaeva
SPICH1036-09|09PROBE-815|Bathyphantes reprobus
SPICH1037-09|09PROBE-816|Bathyphantes reprobus
SPICH341-09|09PROBE-1978-98|Bathyphantes reprobus
SPICH1038-09|09PROBE-817|Bathyphantes reprobus
SPICH1033-09|09PROBE-812|Bathyphantes reprobus
KKCHE638-07|CHU-SPI-435|Bathyphantes reprobus
SPICH1035-09|09PROBE-814|Bathyphantes reprobus
SPICH353-09|09PROBE-1978-110|Bathyphantes reprobus
99
Bathyphantes reprobus
SPICH556-09|09PROBE-1978-313|Bathyphantes gracilis
Bathyphantes gracilis
SPICH489-09|09PROBE-1978-246|Kaestneria pullata
SPIRU1036-11|BIOUG00627-F04|Kaestneria pullata
SPICH380-09|09PROBE-1978-137|Kaestneria pullata
SPICH581-09|09PROBE-1978-338|Kaestneria pullata
SPICH1099-09|09PROBE-878|Kaestneria pullata
SPICH1106-09|09PROBE-885|Kaestneria pullata
SPICH503-09|09PROBE-1978-260|Kaestneria pullata
SPICH303-09|09PROBE-1978-60|Kaestneria pullata
SPICH480-09|09PROBE-1978-237|Kaestneria pullata
SPICH483-09|09PROBE-1978-240|Kaestneria pullata
SPICH1102-09|09PROBE-881|Kaestneria pullata
SPICH248-09|09PROBE-1978-05|Kaestneria pullata
SPICH1084-09|09PROBE-863|Kaestneria pullata
SPICH272-09|09PROBE-1978-29|Kaestneria pullata
SPICH1096-09|09PROBE-875|Kaestneria pullata
SPIRU997-11|BIOUG00627-C01|Kaestneria pullata
SPICH496-09|09PROBE-1978-253|Kaestneria pullata
SPICH271-09|09PROBE-1978-28|Kaestneria pullata
SPICH350-09|09PROBE-1978-107|Kaestneria pullata
SPICH491-09|09PROBE-1978-248|Kaestneria pullata
SPICH530-09|09PROBE-1978-287|Kaestneria pullata
93
81
87
87
97
99
99
Kaestneria pullata
SPICH893-09|09PROBE-650|Pityohyphantes limitaneus
ERSCH144-07|07PROBE-04779|Pityohyphantes limitaneus
ERSCH145-07|07PROBE-04780|Pityohyphantes limitaneus
JDTGS091-09|09-PROBE-08187|Pityohyphantes limitaneus
SPICH1144-09|09PROBE-923|Pityohyphantes limitaneus
ERSCH142-07|07PROBE-04777|Pityohyphantes limitaneus
ERSCH126-07|07PROBE-04761|Pityohyphantes limitaneus
ERSCH107-07|07PROBE-04742|Pityohyphantes limitaneus
ERSCH147-07|07PROBE-04782|Pityohyphantes limitaneus
ERSCH108-07|07PROBE-04743|Pityohyphantes limitaneus
ERSCH101-07|07PROBE-04736|Pityohyphantes limitaneus
ERSCH138-07|07PROBE-04773|Pityohyphantes limitaneus
SPICH894-09|09PROBE-651|Pityohyphantes limitaneus
ERSCH128-07|07PROBE-04763|Pityohyphantes limitaneus
JDTGS084-09|09-PROBE-08180|Pityohyphantes limitaneus
SPICH621-09|09PROBE-1978-378|Pityohyphantes limitaneus
ERSCH135-07|07PROBE-04770|Pityohyphantes limitaneus
ERSCH104-07|07PROBE-04739|Pityohyphantes limitaneus
JDTGS055-09|09-PROBE-08151|Pityohyphantes limitaneus
ERSCH087-07|07PROBE-04632|Pityohyphantes limitaneus
ERSCH137-07|07PROBE-04772|Pityohyphantes limitaneus
SPICH1135-09|09PROBE-914|Pityohyphantes limitaneus
GBADC023-10|10PROBE-21143|Pityohyphantes limitaneus
ERSCH139-07|07PROBE-04774|Pityohyphantes limitaneus
ERSCH148-07|07PROBE-04783|Pityohyphantes limitaneus
SWSWE049-09|09PROBE-01619|Pityohyphantes limitaneus
ERSCH110-07|07PROBE-04745|Pityohyphantes limitaneus
SPICH895-09|09PROBE-652|Pityohyphantes limitaneus
ERSCH141-07|07PROBE-04776|Pityohyphantes limitaneus
ERSCH030-07|07PROBE-04575|Pityohyphantes limitaneus
ERSCH109-07|07PROBE-04744|Pityohyphantes limitaneus
SPICH892-09|09PROBE-649|Pityohyphantes limitaneus
ERSCH123-07|07PROBE-04758|Pityohyphantes limitaneus
JDTGS035-09|09-PROBE-08131|Pityohyphantes limitaneus
SPICH1145-09|09PROBE-924|Pityohyphantes limitaneus
JDTGS068-09|09-PROBE-08164|Pityohyphantes limitaneus
ERSCH146-07|07PROBE-04781|Pityohyphantes limitaneus
SPICH1148-09|09PROBE-927|Pityohyphantes limitaneus
89
81
99
99
93
83
Pityohyphantes limitaneus

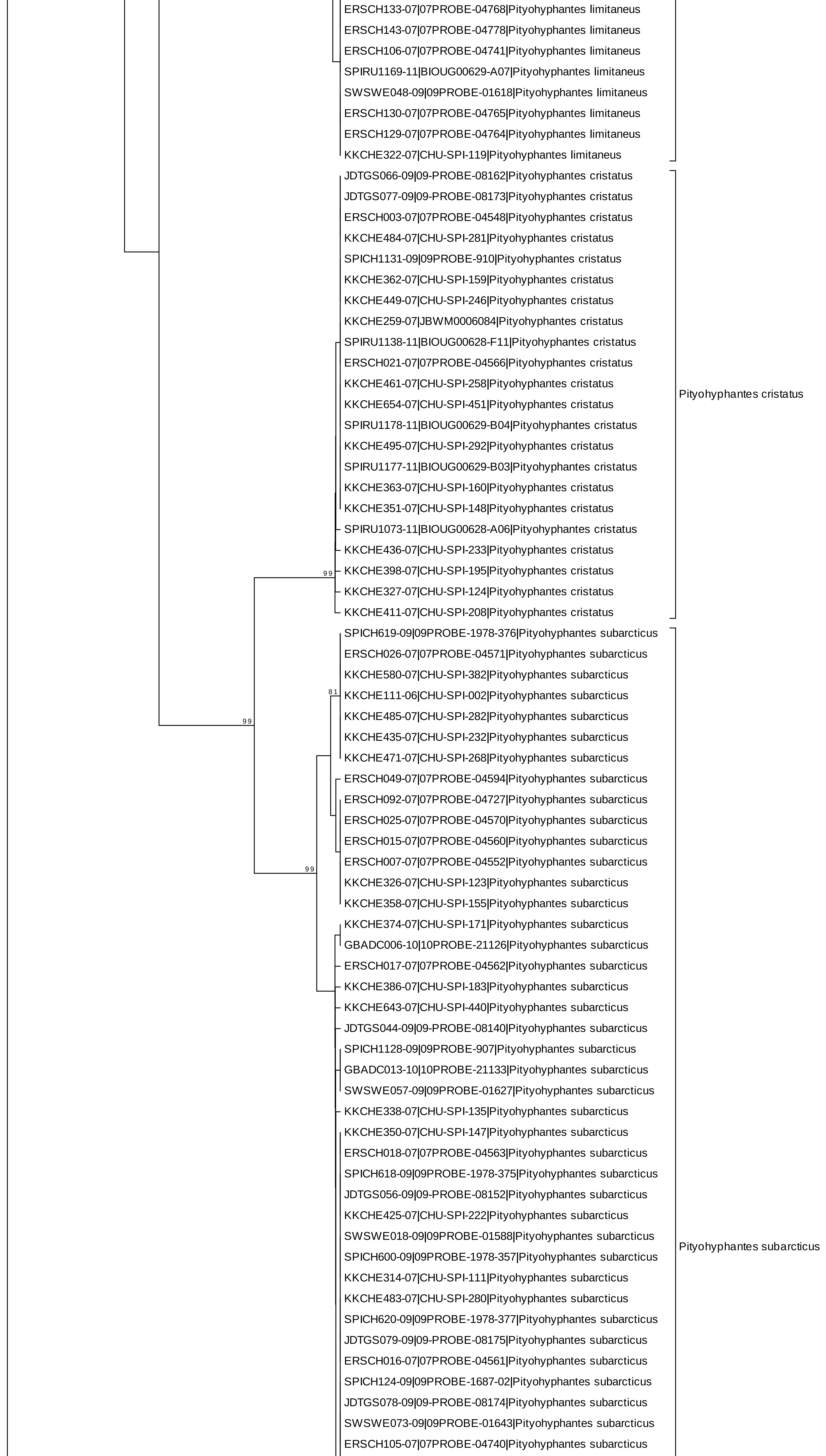
ERSCH133-07|07PROBE-04768|Pityohyphantes limitaneus
ERSCH143-07|07PROBE-04778|Pityohyphantes limitaneus
ERSCH106-07|07PROBE-04741|Pityohyphantes limitaneus
SPIRU1169-11|BIOUG00629-A07|Pityohyphantes limitaneus
SWSWE048-09|09PROBE-01618|Pityohyphantes limitaneus
ERSCH130-07|07PROBE-04765|Pityohyphantes limitaneus
ERSCH129-07|07PROBE-04764|Pityohyphantes limitaneus
KKCHE322-07|CHU-SPI-119|Pityohyphantes limitaneus
JDTGS066-09|09-PROBE-08162|Pityohyphantes cristatus
JDTGS077-09|09-PROBE-08173|Pityohyphantes cristatus
ERSCH003-07|07PROBE-04548|Pityohyphantes cristatus
KKCHE484-07|CHU-SPI-281|Pityohyphantes cristatus
SPICH1131-09|09PROBE-910|Pityohyphantes cristatus
KKCHE362-07|CHU-SPI-159|Pityohyphantes cristatus
KKCHE449-07|CHU-SPI-246|Pityohyphantes cristatus
KKCHE259-07|JBWM0006084|Pityohyphantes cristatus
SPIRU1138-11|BIOUG00628-F11|Pityohyphantes cristatus
ERSCH021-07|07PROBE-04566|Pityohyphantes cristatus
KKCHE461-07|CHU-SPI-258|Pityohyphantes cristatus
KKCHE654-07|CHU-SPI-451|Pityohyphantes cristatus
SPIRU1178-11|BIOUG00629-B04|Pityohyphantes cristatus
KKCHE495-07|CHU-SPI-292|Pityohyphantes cristatus
SPIRU1177-11|BIOUG00629-B03|Pityohyphantes cristatus
KKCHE363-07|CHU-SPI-160|Pityohyphantes cristatus
KKCHE351-07|CHU-SPI-148|Pityohyphantes cristatus
SPIRU1073-11|BIOUG00628-A06|Pityohyphantes cristatus
KKCHE436-07|CHU-SPI-233|Pityohyphantes cristatus
99
KKCHE398-07|CHU-SPI-195|Pityohyphantes cristatus
KKCHE327-07|CHU-SPI-124|Pityohyphantes cristatus
KKCHE411-07|CHU-SPI-208|Pityohyphantes cristatus
Pityohyphantes cristatus
SPICH619-09|09PROBE-1978-376|Pityohyphantes subarcticus
ERSCH026-07|07PROBE-04571|Pityohyphantes subarcticus
KKCHE580-07|CHU-SPI-382|Pityohyphantes subarcticus
81
KKCHE111-06|CHU-SPI-002|Pityohyphantes subarcticus
KKCHE485-07|CHU-SPI-282|Pityohyphantes subarcticus
99
KKCHE435-07|CHU-SPI-232|Pityohyphantes subarcticus
KKCHE471-07|CHU-SPI-268|Pityohyphantes subarcticus
ERSCH049-07|07PROBE-04594|Pityohyphantes subarcticus
ERSCH092-07|07PROBE-04727|Pityohyphantes subarcticus
ERSCH025-07|07PROBE-04570|Pityohyphantes subarcticus
ERSCH015-07|07PROBE-04560|Pityohyphantes subarcticus
99
ERSCH007-07|07PROBE-04552|Pityohyphantes subarcticus
KKCHE326-07|CHU-SPI-123|Pityohyphantes subarcticus
KKCHE358-07|CHU-SPI-155|Pityohyphantes subarcticus
KKCHE374-07|CHU-SPI-171|Pityohyphantes subarcticus
GBADC006-10|10PROBE-21126|Pityohyphantes subarcticus
ERSCH017-07|07PROBE-04562|Pityohyphantes subarcticus
KKCHE386-07|CHU-SPI-183|Pityohyphantes subarcticus
KKCHE643-07|CHU-SPI-440|Pityohyphantes subarcticus
JDTGS044-09|09-PROBE-08140|Pityohyphantes subarcticus
SPICH1128-09|09PROBE-907|Pityohyphantes subarcticus
GBADC013-10|10PROBE-21133|Pityohyphantes subarcticus
SWSWE057-09|09PROBE-01627|Pityohyphantes subarcticus
KKCHE338-07|CHU-SPI-135|Pityohyphantes subarcticus
KKCHE350-07|CHU-SPI-147|Pityohyphantes subarcticus
ERSCH018-07|07PROBE-04563|Pityohyphantes subarcticus
SPICH618-09|09PROBE-1978-375|Pityohyphantes subarcticus
JDTGS056-09|09-PROBE-08152|Pityohyphantes subarcticus
KKCHE425-07|CHU-SPI-222|Pityohyphantes subarcticus
SWSWE018-09|09PROBE-01588|Pityohyphantes subarcticus
Pityohyphantes subarcticus
SPICH600-09|09PROBE-1978-357|Pityohyphantes subarcticus
KKCHE314-07|CHU-SPI-111|Pityohyphantes subarcticus
KKCHE483-07|CHU-SPI-280|Pityohyphantes subarcticus
SPICH620-09|09PROBE-1978-377|Pityohyphantes subarcticus
JDTGS079-09|09-PROBE-08175|Pityohyphantes subarcticus
ERSCH016-07|07PROBE-04561|Pityohyphantes subarcticus
SPICH124-09|09PROBE-1687-02|Pityohyphantes subarcticus
JDTGS078-09|09-PROBE-08174|Pityohyphantes subarcticus
SWSWE073-09|09PROBE-01643|Pityohyphantes subarcticus
ERSCH105-07|07PROBE-04740|Pityohyphantes subarcticus

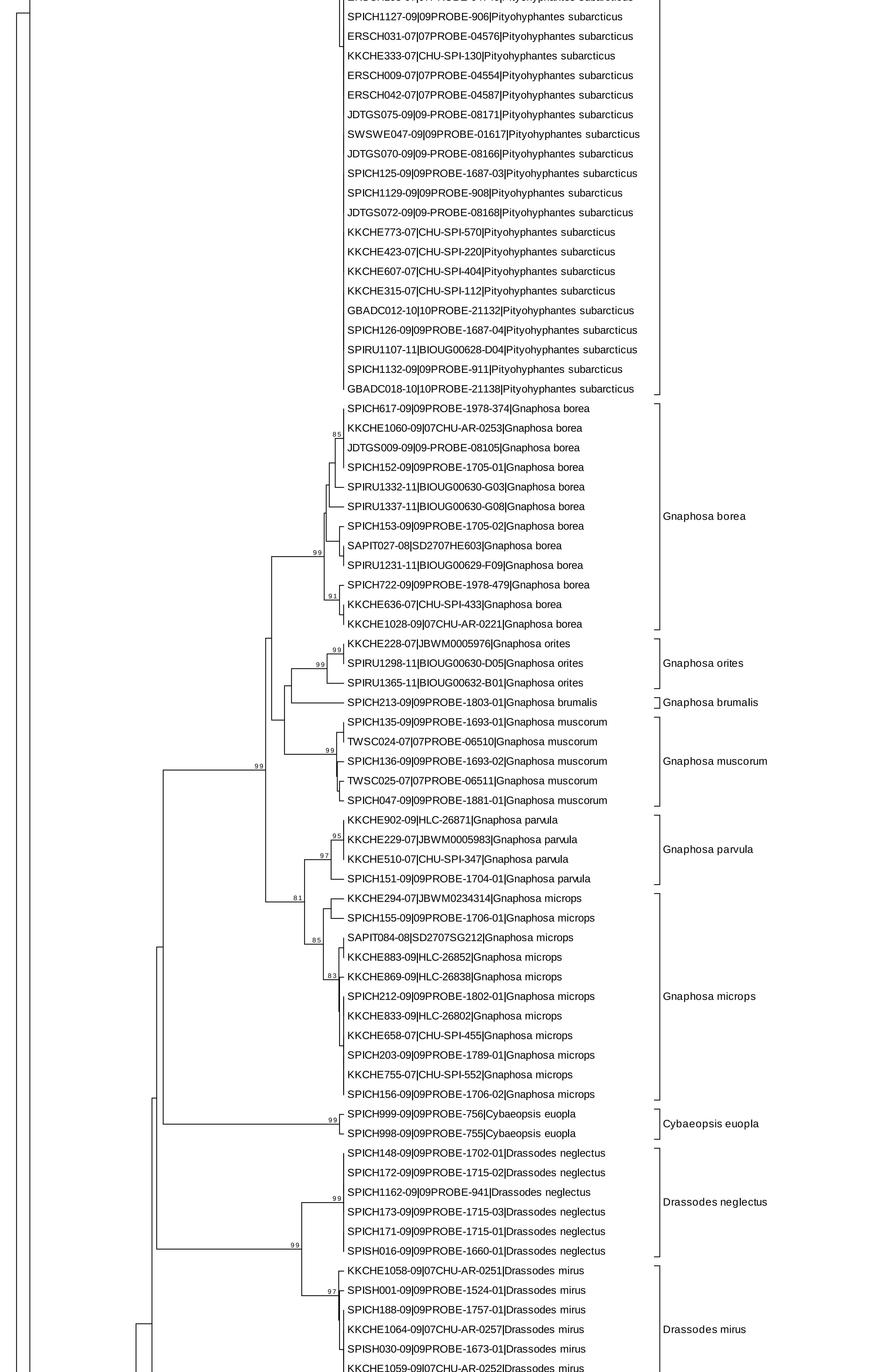
SPICH1127-09|09PROBE-906|Pityohyphantes subarcticus
ERSCH031-07|07PROBE-04576|Pityohyphantes subarcticus
KKCHE333-07|CHU-SPI-130|Pityohyphantes subarcticus
ERSCH009-07|07PROBE-04554|Pityohyphantes subarcticus
ERSCH042-07|07PROBE-04587|Pityohyphantes subarcticus
JDTGS075-09|09-PROBE-08171|Pityohyphantes subarcticus
SWSWE047-09|09PROBE-01617|Pityohyphantes subarcticus
JDTGS070-09|09-PROBE-08166|Pityohyphantes subarcticus
SPICH125-09|09PROBE-1687-03|Pityohyphantes subarcticus
SPICH1129-09|09PROBE-908|Pityohyphantes subarcticus
JDTGS072-09|09-PROBE-08168|Pityohyphantes subarcticus
KKCHE773-07|CHU-SPI-570|Pityohyphantes subarcticus
KKCHE423-07|CHU-SPI-220|Pityohyphantes subarcticus
KKCHE607-07|CHU-SPI-404|Pityohyphantes subarcticus
KKCHE315-07|CHU-SPI-112|Pityohyphantes subarcticus
GBADC012-10|10PROBE-21132|Pityohyphantes subarcticus
SPICH126-09|09PROBE-1687-04|Pityohyphantes subarcticus
SPIRU1107-11|BIOUG00628-D04|Pityohyphantes subarcticus
SPICH1132-09|09PROBE-911|Pityohyphantes subarcticus
GBADC018-10|10PROBE-21138|Pityohyphantes subarcticus
SPICH617-09|09PROBE-1978-374|Gnaphosa borea
KKCHE1060-09|07CHU-AR-0253|Gnaphosa borea
JDTGS009-09|09-PROBE-08105|Gnaphosa borea
SPICH152-09|09PROBE-1705-01|Gnaphosa borea
SPIRU1332-11|BIOUG00630-G03|Gnaphosa borea
SPIRU1337-11|BIOUG00630-G08|Gnaphosa borea
SPICH153-09|09PROBE-1705-02|Gnaphosa borea
SAPIT027-08|SD2707HE603|Gnaphosa borea
SPIRU1231-11|BIOUG00629-F09|Gnaphosa borea
SPICH722-09|09PROBE-1978-479|Gnaphosa borea
KKCHE636-07|CHU-SPI-433|Gnaphosa borea
KKCHE1028-09|07CHU-AR-0221|Gnaphosa borea
Gnaphosa borea
KKCHE228-07|JBWM0005976|Gnaphosa orites
SPIRU1298-11|BIOUG00630-D05|Gnaphosa orites
SPIRU1365-11|BIOUG00632-B01|Gnaphosa orites
Gnaphosa orites
SPICH213-09|09PROBE-1803-01|Gnaphosa brumalis
Gnaphosa brumalis
SPICH135-09|09PROBE-1693-01|Gnaphosa muscorum
TWSC024-07|07PROBE-06510|Gnaphosa muscorum
SPICH136-09|09PROBE-1693-02|Gnaphosa muscorum
TWSC025-07|07PROBE-06511|Gnaphosa muscorum
SPICH047-09|09PROBE-1881-01|Gnaphosa muscorum
Gnaphosa muscorum
KKCHE902-09|HLC-26871|Gnaphosa parvula
KKCHE229-07|JBWM0005983|Gnaphosa parvula
KKCHE510-07|CHU-SPI-347|Gnaphosa parvula
SPICH151-09|09PROBE-1704-01|Gnaphosa parvula
Gnaphosa parvula
KKCHE294-07|JBWM0234314|Gnaphosa microps
SPICH155-09|09PROBE-1706-01|Gnaphosa microps
SAPIT084-08|SD2707SG212|Gnaphosa microps
KKCHE883-09|HLC-26852|Gnaphosa microps
KKCHE869-09|HLC-26838|Gnaphosa microps
SPICH212-09|09PROBE-1802-01|Gnaphosa microps
KKCHE833-09|HLC-26802|Gnaphosa microps
KKCHE658-07|CHU-SPI-455|Gnaphosa microps
SPICH203-09|09PROBE-1789-01|Gnaphosa microps
KKCHE755-07|CHU-SPI-552|Gnaphosa microps
SPICH156-09|09PROBE-1706-02|Gnaphosa microps
Gnaphosa microps
SPICH999-09|09PROBE-756|Cybaeopsis euopla
SPICH998-09|09PROBE-755|Cybaeopsis euopla
Cybaeopsis euopla
SPICH148-09|09PROBE-1702-01|Drassodes neglectus
SPICH172-09|09PROBE-1715-02|Drassodes neglectus
SPICH1162-09|09PROBE-941|Drassodes neglectus
SPICH173-09|09PROBE-1715-03|Drassodes neglectus
SPICH171-09|09PROBE-1715-01|Drassodes neglectus
SPISH016-09|09PROBE-1660-01|Drassodes neglectus
Drassodes neglectus
KKCHE1058-09|07CHU-AR-0251|Drassodes mirus
SPISH001-09|09PROBE-1524-01|Drassodes mirus
SPICH188-09|09PROBE-1757-01|Drassodes mirus
KKCHE1064-09|07CHU-AR-0257|Drassodes mirus
SPISH030-09|09PROBE-1673-01|Drassodes mirus
KKCHE1059-09|07CHU-AR-0252|Drassodes mirus
Drassodes mirus

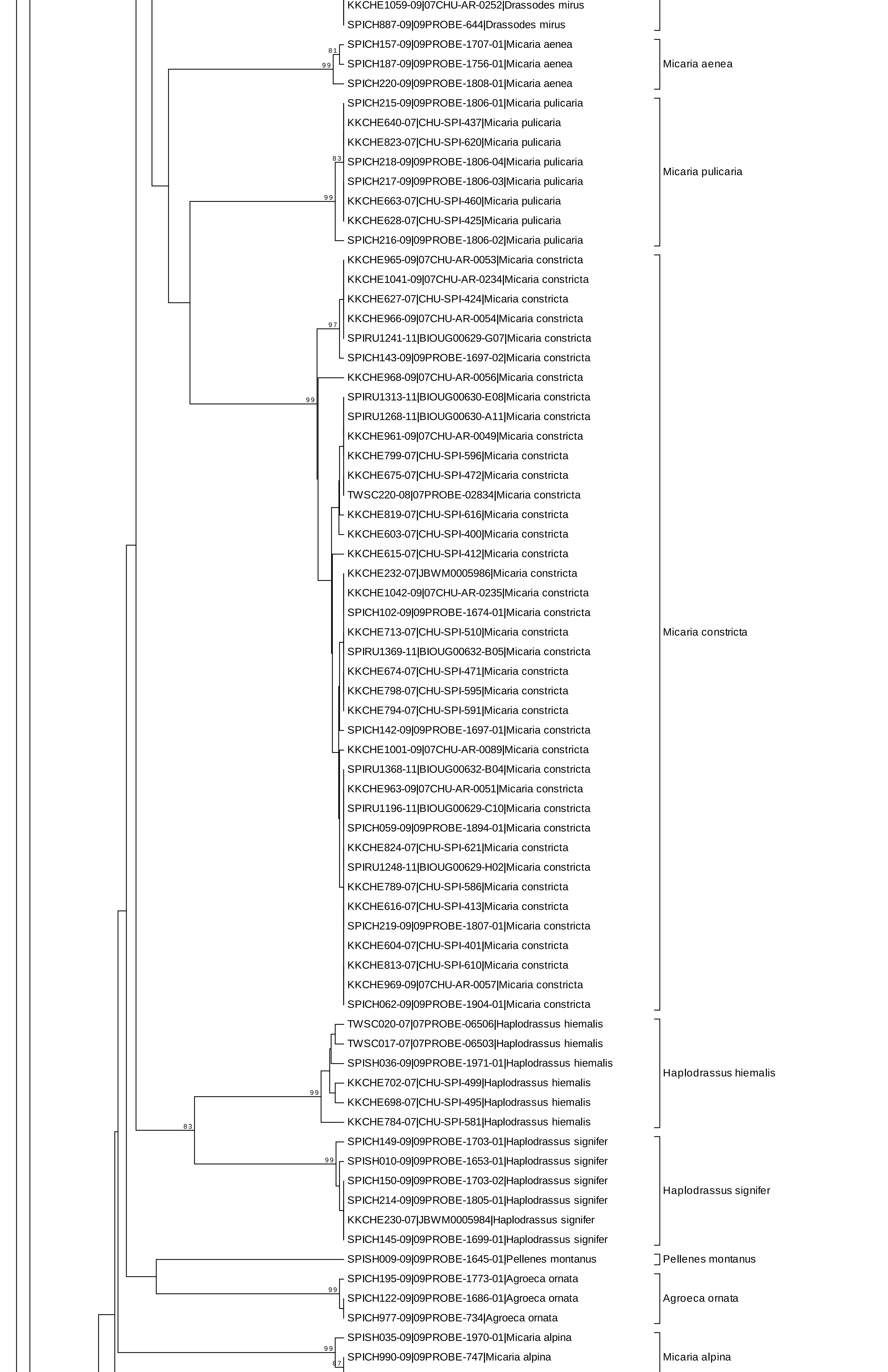
KKCHE1059-09|07CHU-AR-0252|Drassodes mirus
SPICH887-09|09PROBE-644|Drassodes mirus
SPICH157-09|09PROBE-1707-01|Micaria aenea
SPICH187-09|09PROBE-1756-01|Micaria aenea
SPICH220-09|09PROBE-1808-01|Micaria aenea
Micaria aenea
SPICH215-09|09PROBE-1806-01|Micaria pulicaria
KKCHE640-07|CHU-SPI-437|Micaria pulicaria
KKCHE823-07|CHU-SPI-620|Micaria pulicaria
SPICH218-09|09PROBE-1806-04|Micaria pulicaria
SPICH217-09|09PROBE-1806-03|Micaria pulicaria
KKCHE663-07|CHU-SPI-460|Micaria pulicaria
KKCHE628-07|CHU-SPI-425|Micaria pulicaria
SPICH216-09|09PROBE-1806-02|Micaria pulicaria
Micaria pulicaria
KKCHE965-09|07CHU-AR-0053|Micaria constricta
KKCHE1041-09|07CHU-AR-0234|Micaria constricta
KKCHE627-07|CHU-SPI-424|Micaria constricta
KKCHE966-09|07CHU-AR-0054|Micaria constricta
SPIRU1241-11|BIOUG00629-G07|Micaria constricta
SPICH143-09|09PROBE-1697-02|Micaria constricta
KKCHE968-09|07CHU-AR-0056|Micaria constricta
SPIRU1313-11|BIOUG00630-E08|Micaria constricta
SPIRU1268-11|BIOUG00630-A11|Micaria constricta
KKCHE961-09|07CHU-AR-0049|Micaria constricta
KKCHE799-07|CHU-SPI-596|Micaria constricta
KKCHE675-07|CHU-SPI-472|Micaria constricta
TWSC220-08|07PROBE-02834|Micaria constricta
KKCHE819-07|CHU-SPI-616|Micaria constricta
KKCHE603-07|CHU-SPI-400|Micaria constricta
KKCHE615-07|CHU-SPI-412|Micaria constricta
KKCHE232-07|JBWM0005986|Micaria constricta
KKCHE1042-09|07CHU-AR-0235|Micaria constricta
SPICH102-09|09PROBE-1674-01|Micaria constricta
KKCHE713-07|CHU-SPI-510|Micaria constricta
SPIRU1369-11|BIOUG00632-B05|Micaria constricta
KKCHE674-07|CHU-SPI-471|Micaria constricta
KKCHE798-07|CHU-SPI-595|Micaria constricta
KKCHE794-07|CHU-SPI-591|Micaria constricta
SPICH142-09|09PROBE-1697-01|Micaria constricta
KKCHE1001-09|07CHU-AR-0089|Micaria constricta
SPIRU1368-11|BIOUG00632-B04|Micaria constricta
KKCHE963-09|07CHU-AR-0051|Micaria constricta
SPIRU1196-11|BIOUG00629-C10|Micaria constricta
SPICH059-09|09PROBE-1894-01|Micaria constricta
KKCHE824-07|CHU-SPI-621|Micaria constricta
SPIRU1248-11|BIOUG00629-H02|Micaria constricta
KKCHE789-07|CHU-SPI-586|Micaria constricta
KKCHE616-07|CHU-SPI-413|Micaria constricta
SPICH219-09|09PROBE-1807-01|Micaria constricta
KKCHE604-07|CHU-SPI-401|Micaria constricta
KKCHE813-07|CHU-SPI-610|Micaria constricta
KKCHE969-09|07CHU-AR-0057|Micaria constricta
SPICH062-09|09PROBE-1904-01|Micaria constricta
Micaria constricta
TWSC020-07|07PROBE-06506|Haplodrassus hiemalis
TWSC017-07|07PROBE-06503|Haplodrassus hiemalis
SPISH036-09|09PROBE-1971-01|Haplodrassus hiemalis
KKCHE702-07|CHU-SPI-499|Haplodrassus hiemalis
KKCHE698-07|CHU-SPI-495|Haplodrassus hiemalis
KKCHE784-07|CHU-SPI-581|Haplodrassus hiemalis
Haplodrassus hiemalis
SPICH149-09|09PROBE-1703-01|Haplodrassus signifer
SPISH010-09|09PROBE-1653-01|Haplodrassus signifer
SPICH150-09|09PROBE-1703-02|Haplodrassus signifer
SPICH214-09|09PROBE-1805-01|Haplodrassus signifer
KKCHE230-07|JBWM0005984|Haplodrassus signifer
SPICH145-09|09PROBE-1699-01|Haplodrassus signifer
Haplodrassus signifer
SPISH009-09|09PROBE-1645-01|Pellenes montanus
Pellenes montanus
SPICH195-09|09PROBE-1773-01|Agroeca ornata
SPICH122-09|09PROBE-1686-01|Agroeca ornata
SPICH977-09|09PROBE-734|Agroeca ornata
Agroeca ornata
SPISH035-09|09PROBE-1970-01|Micaria alpina
SPICH990-09|09PROBE-747|Micaria alpina
Micaria alpina

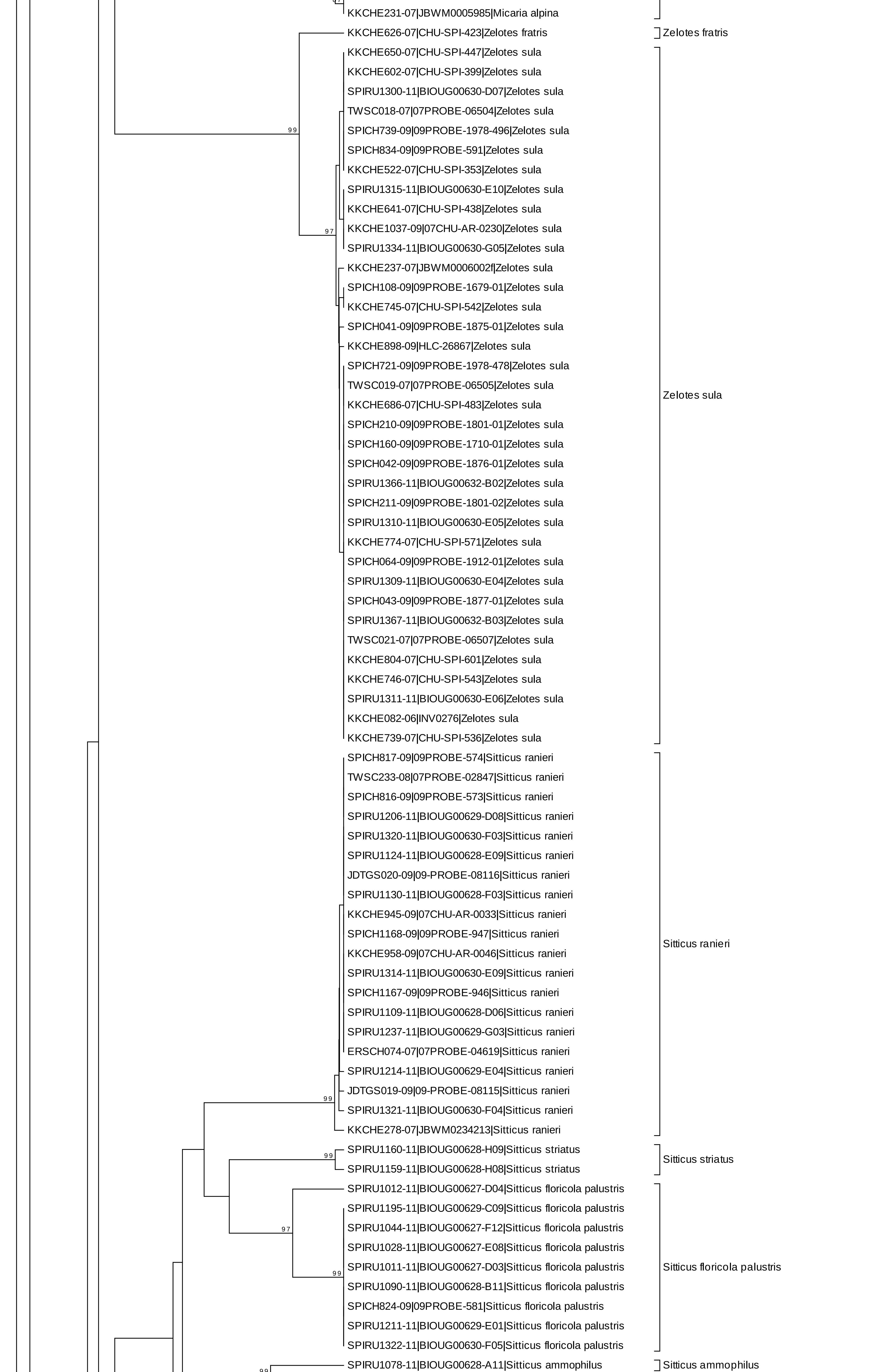
KKCHE231-07|JBWM0005985|Micaria alpina
KKCHE626-07|CHU-SPI-423|Zelotes fratris
Zelotes fratris
KKCHE650-07|CHU-SPI-447|Zelotes sula
KKCHE602-07|CHU-SPI-399|Zelotes sula
SPIRU1300-11|BIOUG00630-D07|Zelotes sula
TWSC018-07|07PROBE-06504|Zelotes sula
SPICH739-09|09PROBE-1978-496|Zelotes sula
SPICH834-09|09PROBE-591|Zelotes sula
KKCHE522-07|CHU-SPI-353|Zelotes sula
SPIRU1315-11|BIOUG00630-E10|Zelotes sula
KKCHE641-07|CHU-SPI-438|Zelotes sula
KKCHE1037-09|07CHU-AR-0230|Zelotes sula
SPIRU1334-11|BIOUG00630-G05|Zelotes sula
KKCHE237-07|JBWM0006002f|Zelotes sula
SPICH108-09|09PROBE-1679-01|Zelotes sula
KKCHE745-07|CHU-SPI-542|Zelotes sula
SPICH041-09|09PROBE-1875-01|Zelotes sula
KKCHE898-09|HLC-26867|Zelotes sula
SPICH721-09|09PROBE-1978-478|Zelotes sula
TWSC019-07|07PROBE-06505|Zelotes sula
KKCHE686-07|CHU-SPI-483|Zelotes sula
SPICH210-09|09PROBE-1801-01|Zelotes sula
SPICH160-09|09PROBE-1710-01|Zelotes sula
SPICH042-09|09PROBE-1876-01|Zelotes sula
SPIRU1366-11|BIOUG00632-B02|Zelotes sula
SPICH211-09|09PROBE-1801-02|Zelotes sula
SPIRU1310-11|BIOUG00630-E05|Zelotes sula
KKCHE774-07|CHU-SPI-571|Zelotes sula
SPICH064-09|09PROBE-1912-01|Zelotes sula
SPIRU1309-11|BIOUG00630-E04|Zelotes sula
SPICH043-09|09PROBE-1877-01|Zelotes sula
SPIRU1367-11|BIOUG00632-B03|Zelotes sula
TWSC021-07|07PROBE-06507|Zelotes sula
KKCHE804-07|CHU-SPI-601|Zelotes sula
KKCHE746-07|CHU-SPI-543|Zelotes sula
SPIRU1311-11|BIOUG00630-E06|Zelotes sula
KKCHE082-06|INV0276|Zelotes sula
KKCHE739-07|CHU-SPI-536|Zelotes sula
Zelotes sula
99
97
SPICH817-09|09PROBE-574|Sitticus ranieri
TWSC233-08|07PROBE-02847|Sitticus ranieri
SPICH816-09|09PROBE-573|Sitticus ranieri
SPIRU1206-11|BIOUG00629-D08|Sitticus ranieri
SPIRU1320-11|BIOUG00630-F03|Sitticus ranieri
SPIRU1124-11|BIOUG00628-E09|Sitticus ranieri
JDTGS020-09|09-PROBE-08116|Sitticus ranieri
SPIRU1130-11|BIOUG00628-F03|Sitticus ranieri
KKCHE945-09|07CHU-AR-0033|Sitticus ranieri
SPICH1168-09|09PROBE-947|Sitticus ranieri
KKCHE958-09|07CHU-AR-0046|Sitticus ranieri
SPIRU1314-11|BIOUG00630-E09|Sitticus ranieri
SPICH1167-09|09PROBE-946|Sitticus ranieri
SPIRU1109-11|BIOUG00628-D06|Sitticus ranieri
SPIRU1237-11|BIOUG00629-G03|Sitticus ranieri
ERSCH074-07|07PROBE-04619|Sitticus ranieri
SPIRU1214-11|BIOUG00629-E04|Sitticus ranieri
JDTGS019-09|09-PROBE-08115|Sitticus ranieri
SPIRU1321-11|BIOUG00630-F04|Sitticus ranieri
KKCHE278-07|JBWM0234213|Sitticus ranieri
Sitticus ranieri
99
SPIRU1160-11|BIOUG00628-H09|Sitticus striatus
SPIRU1159-11|BIOUG00628-H08|Sitticus striatus
Sitticus striatus
99
SPIRU1012-11|BIOUG00627-D04|Sitticus floricola palustris
SPIRU1195-11|BIOUG00629-C09|Sitticus floricola palustris
SPIRU1044-11|BIOUG00627-F12|Sitticus floricola palustris
SPIRU1028-11|BIOUG00627-E08|Sitticus floricola palustris
SPIRU1011-11|BIOUG00627-D03|Sitticus floricola palustris
SPIRU1090-11|BIOUG00628-B11|Sitticus floricola palustris
SPICH824-09|09PROBE-581|Sitticus floricola palustris
SPIRU1211-11|BIOUG00629-E01|Sitticus floricola palustris
SPIRU1322-11|BIOUG00630-F05|Sitticus floricola palustris
Sitticus floricola palustris
97
99
SPIRU1078-11|BIOUG00628-A11|Sitticus ammophilus
Sitticus ammophilus
99

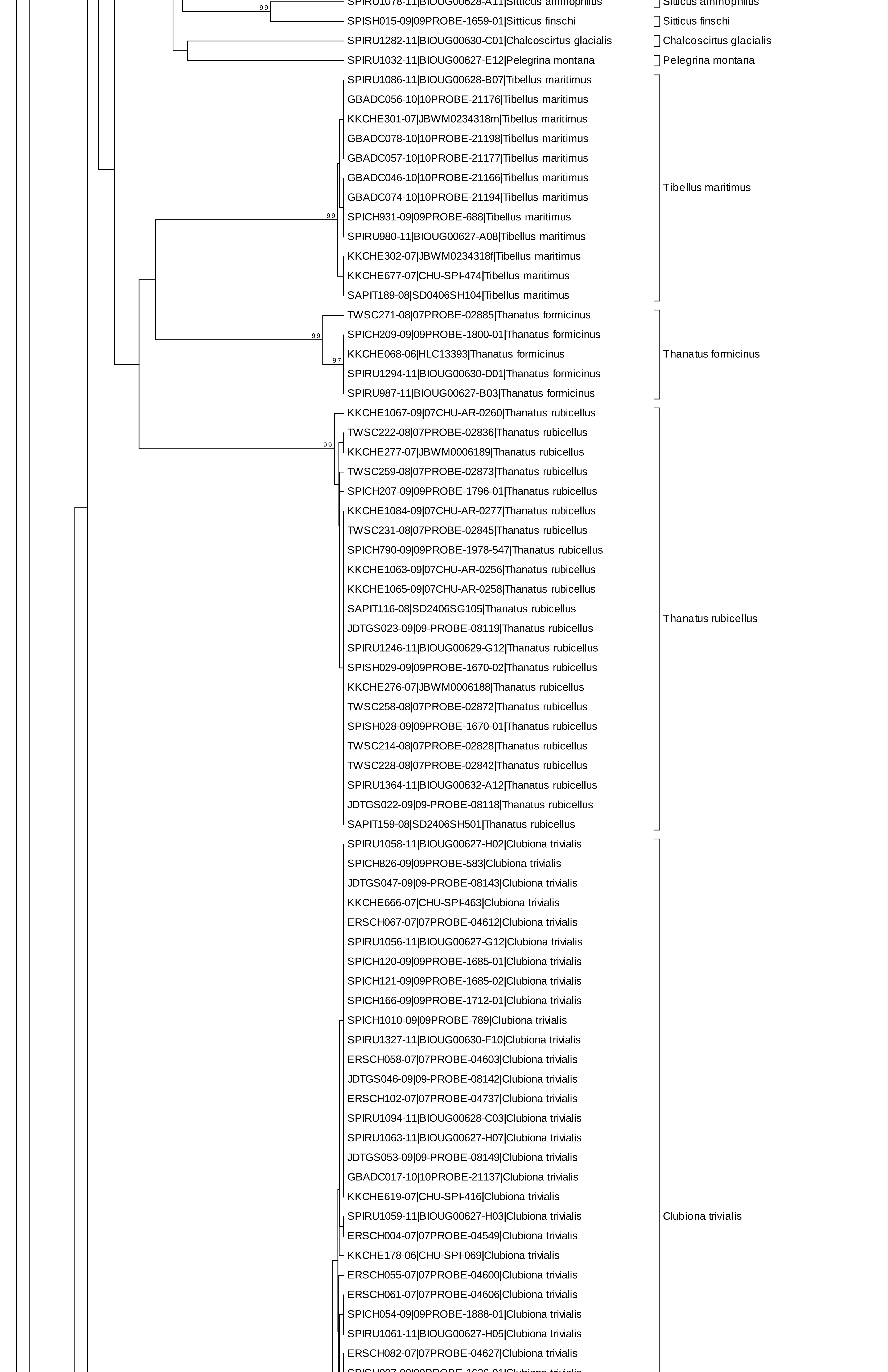
SPIRU1078-11|BIOUG00628-A11|Sitticus ammophilus
Sitticus ammophilus
99
SPISH015-09|09PROBE-1659-01|Sitticus finschi
Sitticus finschi
SPIRU1282-11|BIOUG00630-C01|Chalcoscirtus glacialis
Chalcoscirtus glacialis
SPIRU1032-11|BIOUG00627-E12|Pelegrina montana
Pelegrina montana
SPIRU1086-11|BIOUG00628-B07|Tibellus maritimus
GBADC056-10|10PROBE-21176|Tibellus maritimus
KKCHE301-07|JBWM0234318m|Tibellus maritimus
GBADC078-10|10PROBE-21198|Tibellus maritimus
GBADC057-10|10PROBE-21177|Tibellus maritimus
GBADC046-10|10PROBE-21166|Tibellus maritimus
GBADC074-10|10PROBE-21194|Tibellus maritimus
99
SPICH931-09|09PROBE-688|Tibellus maritimus
SPIRU980-11|BIOUG00627-A08|Tibellus maritimus
KKCHE302-07|JBWM0234318f|Tibellus maritimus
KKCHE677-07|CHU-SPI-474|Tibellus maritimus
SAPIT189-08|SD0406SH104|Tibellus maritimus
Tibellus maritimus
TWSC271-08|07PROBE-02885|Thanatus formicinus
99
SPICH209-09|09PROBE-1800-01|Thanatus formicinus
KKCHE068-06|HLC13393|Thanatus formicinus
97
SPIRU1294-11|BIOUG00630-D01|Thanatus formicinus
SPIRU987-11|BIOUG00627-B03|Thanatus formicinus
Thanatus formicinus
KKCHE1067-09|07CHU-AR-0260|Thanatus rubicellus
TWSC222-08|07PROBE-02836|Thanatus rubicellus
99
KKCHE277-07|JBWM0006189|Thanatus rubicellus
TWSC259-08|07PROBE-02873|Thanatus rubicellus
SPICH207-09|09PROBE-1796-01|Thanatus rubicellus
KKCHE1084-09|07CHU-AR-0277|Thanatus rubicellus
TWSC231-08|07PROBE-02845|Thanatus rubicellus
SPICH790-09|09PROBE-1978-547|Thanatus rubicellus
KKCHE1063-09|07CHU-AR-0256|Thanatus rubicellus
KKCHE1065-09|07CHU-AR-0258|Thanatus rubicellus
SAPIT116-08|SD2406SG105|Thanatus rubicellus
JDTGS023-09|09-PROBE-08119|Thanatus rubicellus
SPIRU1246-11|BIOUG00629-G12|Thanatus rubicellus
SPISH029-09|09PROBE-1670-02|Thanatus rubicellus
KKCHE276-07|JBWM0006188|Thanatus rubicellus
TWSC258-08|07PROBE-02872|Thanatus rubicellus
SPISH028-09|09PROBE-1670-01|Thanatus rubicellus
TWSC214-08|07PROBE-02828|Thanatus rubicellus
TWSC228-08|07PROBE-02842|Thanatus rubicellus
SPIRU1364-11|BIOUG00632-A12|Thanatus rubicellus
JDTGS022-09|09-PROBE-08118|Thanatus rubicellus
SAPIT159-08|SD2406SH501|Thanatus rubicellus
Thanatus rubicellus
SPIRU1058-11|BIOUG00627-H02|Clubiona trivialis
SPICH826-09|09PROBE-583|Clubiona trivialis
JDTGS047-09|09-PROBE-08143|Clubiona trivialis
KKCHE666-07|CHU-SPI-463|Clubiona trivialis
ERSCH067-07|07PROBE-04612|Clubiona trivialis
SPIRU1056-11|BIOUG00627-G12|Clubiona trivialis
SPICH120-09|09PROBE-1685-01|Clubiona trivialis
SPICH121-09|09PROBE-1685-02|Clubiona trivialis
SPICH166-09|09PROBE-1712-01|Clubiona trivialis
SPICH1010-09|09PROBE-789|Clubiona trivialis
SPIRU1327-11|BIOUG00630-F10|Clubiona trivialis
ERSCH058-07|07PROBE-04603|Clubiona trivialis
JDTGS046-09|09-PROBE-08142|Clubiona trivialis
ERSCH102-07|07PROBE-04737|Clubiona trivialis
SPIRU1094-11|BIOUG00628-C03|Clubiona trivialis
SPIRU1063-11|BIOUG00627-H07|Clubiona trivialis
JDTGS053-09|09-PROBE-08149|Clubiona trivialis
GBADC017-10|10PROBE-21137|Clubiona trivialis
KKCHE619-07|CHU-SPI-416|Clubiona trivialis
SPIRU1059-11|BIOUG00627-H03|Clubiona trivialis
ERSCH004-07|07PROBE-04549|Clubiona trivialis
KKCHE178-06|CHU-SPI-069|Clubiona trivialis
ERSCH055-07|07PROBE-04600|Clubiona trivialis
ERSCH061-07|07PROBE-04606|Clubiona trivialis
SPICH054-09|09PROBE-1888-01|Clubiona trivialis
SPIRU1061-11|BIOUG00627-H05|Clubiona trivialis
ERSCH082-07|07PROBE-04627|Clubiona trivialis
Clubiona trivialis

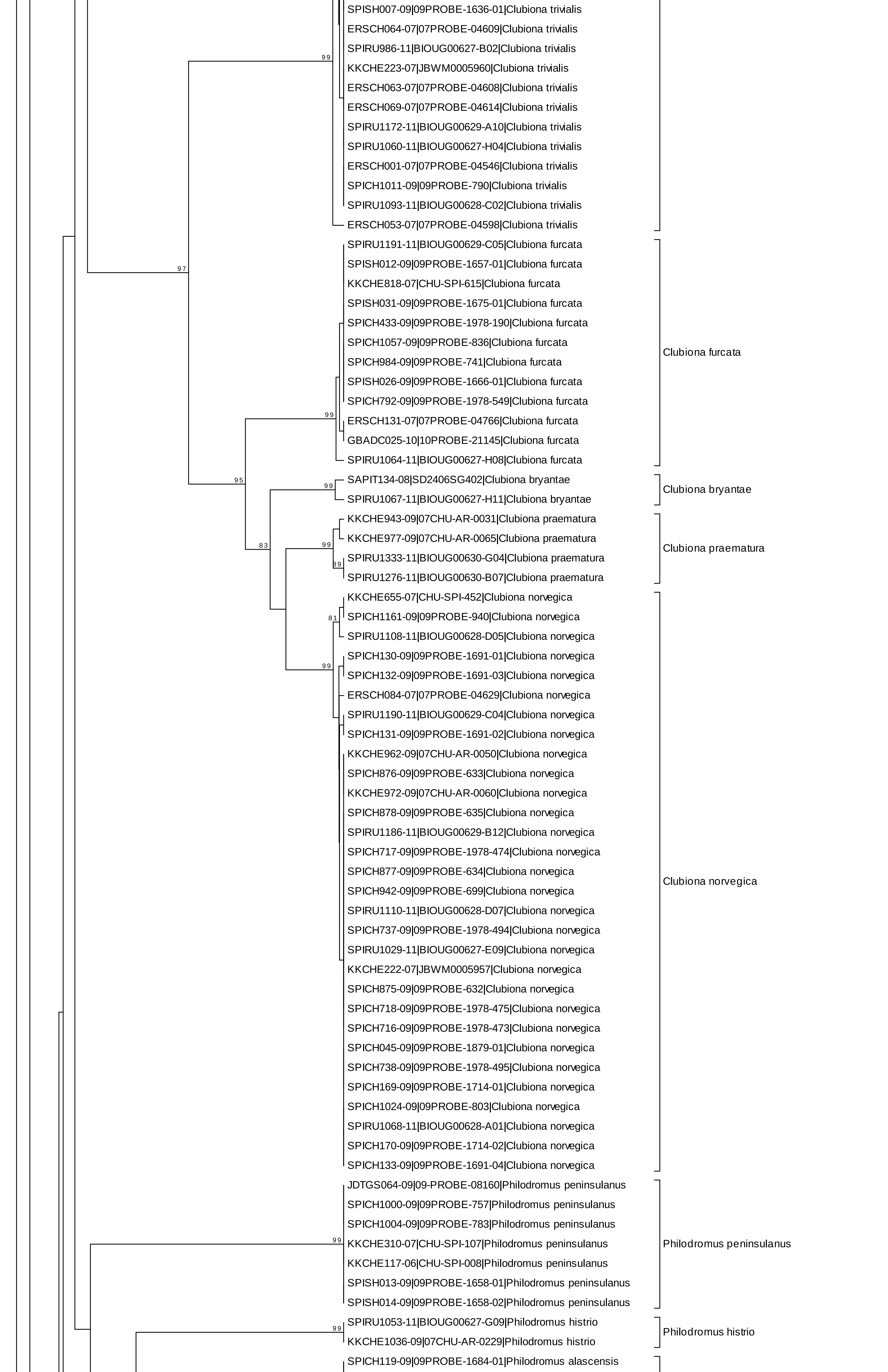
SPISH007-09|09PROBE-1636-01|Clubiona trivialis
ERSCH064-07|07PROBE-04609|Clubiona trivialis
SPIRU986-11|BIOUG00627-B02|Clubiona trivialis
KKCHE223-07|JBWM0005960|Clubiona trivialis
ERSCH063-07|07PROBE-04608|Clubiona trivialis
ERSCH069-07|07PROBE-04614|Clubiona trivialis
SPIRU1172-11|BIOUG00629-A10|Clubiona trivialis
SPIRU1060-11|BIOUG00627-H04|Clubiona trivialis
ERSCH001-07|07PROBE-04546|Clubiona trivialis
SPICH1011-09|09PROBE-790|Clubiona trivialis
SPIRU1093-11|BIOUG00628-C02|Clubiona trivialis
ERSCH053-07|07PROBE-04598|Clubiona trivialis
99
97
SPIRU1191-11|BIOUG00629-C05|Clubiona furcata
SPISH012-09|09PROBE-1657-01|Clubiona furcata
KKCHE818-07|CHU-SPI-615|Clubiona furcata
SPISH031-09|09PROBE-1675-01|Clubiona furcata
SPICH433-09|09PROBE-1978-190|Clubiona furcata
SPICH1057-09|09PROBE-836|Clubiona furcata
SPICH984-09|09PROBE-741|Clubiona furcata
SPISH026-09|09PROBE-1666-01|Clubiona furcata
SPICH792-09|09PROBE-1978-549|Clubiona furcata
ERSCH131-07|07PROBE-04766|Clubiona furcata
GBADC025-10|10PROBE-21145|Clubiona furcata
SPIRU1064-11|BIOUG00627-H08|Clubiona furcata
99
Clubiona furcata
95
SAPIT134-08|SD2406SG402|Clubiona bryantae
SPIRU1067-11|BIOUG00627-H11|Clubiona bryantae
99
Clubiona bryantae
83
KKCHE943-09|07CHU-AR-0031|Clubiona praematura
KKCHE977-09|07CHU-AR-0065|Clubiona praematura
SPIRU1333-11|BIOUG00630-G04|Clubiona praematura
SPIRU1276-11|BIOUG00630-B07|Clubiona praematura
99
Clubiona praematura
KKCHE655-07|CHU-SPI-452|Clubiona norvegica
SPICH1161-09|09PROBE-940|Clubiona norvegica
SPIRU1108-11|BIOUG00628-D05|Clubiona norvegica
81
SPICH130-09|09PROBE-1691-01|Clubiona norvegica
SPICH132-09|09PROBE-1691-03|Clubiona norvegica
ERSCH084-07|07PROBE-04629|Clubiona norvegica
SPIRU1190-11|BIOUG00629-C04|Clubiona norvegica
SPICH131-09|09PROBE-1691-02|Clubiona norvegica
99
KKCHE962-09|07CHU-AR-0050|Clubiona norvegica
SPICH876-09|09PROBE-633|Clubiona norvegica
KKCHE972-09|07CHU-AR-0060|Clubiona norvegica
SPICH878-09|09PROBE-635|Clubiona norvegica
SPIRU1186-11|BIOUG00629-B12|Clubiona norvegica
SPICH717-09|09PROBE-1978-474|Clubiona norvegica
SPICH877-09|09PROBE-634|Clubiona norvegica
SPICH942-09|09PROBE-699|Clubiona norvegica
SPIRU1110-11|BIOUG00628-D07|Clubiona norvegica
SPICH737-09|09PROBE-1978-494|Clubiona norvegica
SPIRU1029-11|BIOUG00627-E09|Clubiona norvegica
KKCHE222-07|JBWM0005957|Clubiona norvegica
SPICH875-09|09PROBE-632|Clubiona norvegica
SPICH718-09|09PROBE-1978-475|Clubiona norvegica
SPICH716-09|09PROBE-1978-473|Clubiona norvegica
SPICH045-09|09PROBE-1879-01|Clubiona norvegica
SPICH738-09|09PROBE-1978-495|Clubiona norvegica
SPICH169-09|09PROBE-1714-01|Clubiona norvegica
SPICH1024-09|09PROBE-803|Clubiona norvegica
SPIRU1068-11|BIOUG00628-A01|Clubiona norvegica
SPICH170-09|09PROBE-1714-02|Clubiona norvegica
SPICH133-09|09PROBE-1691-04|Clubiona norvegica
Clubiona norvegica
JDTGS064-09|09-PROBE-08160|Philodromus peninsulanus
SPICH1000-09|09PROBE-757|Philodromus peninsulanus
SPICH1004-09|09PROBE-783|Philodromus peninsulanus
KKCHE310-07|CHU-SPI-107|Philodromus peninsulanus
KKCHE117-06|CHU-SPI-008|Philodromus peninsulanus
SPISH013-09|09PROBE-1658-01|Philodromus peninsulanus
SPISH014-09|09PROBE-1658-02|Philodromus peninsulanus
99
Philodromus peninsulanus
SPIRU1053-11|BIOUG00627-G09|Philodromus histrio
KKCHE1036-09|07CHU-AR-0229|Philodromus histrio
99
Philodromus histrio
SPICH119-09|09PROBE-1684-01|Philodromus alascensis

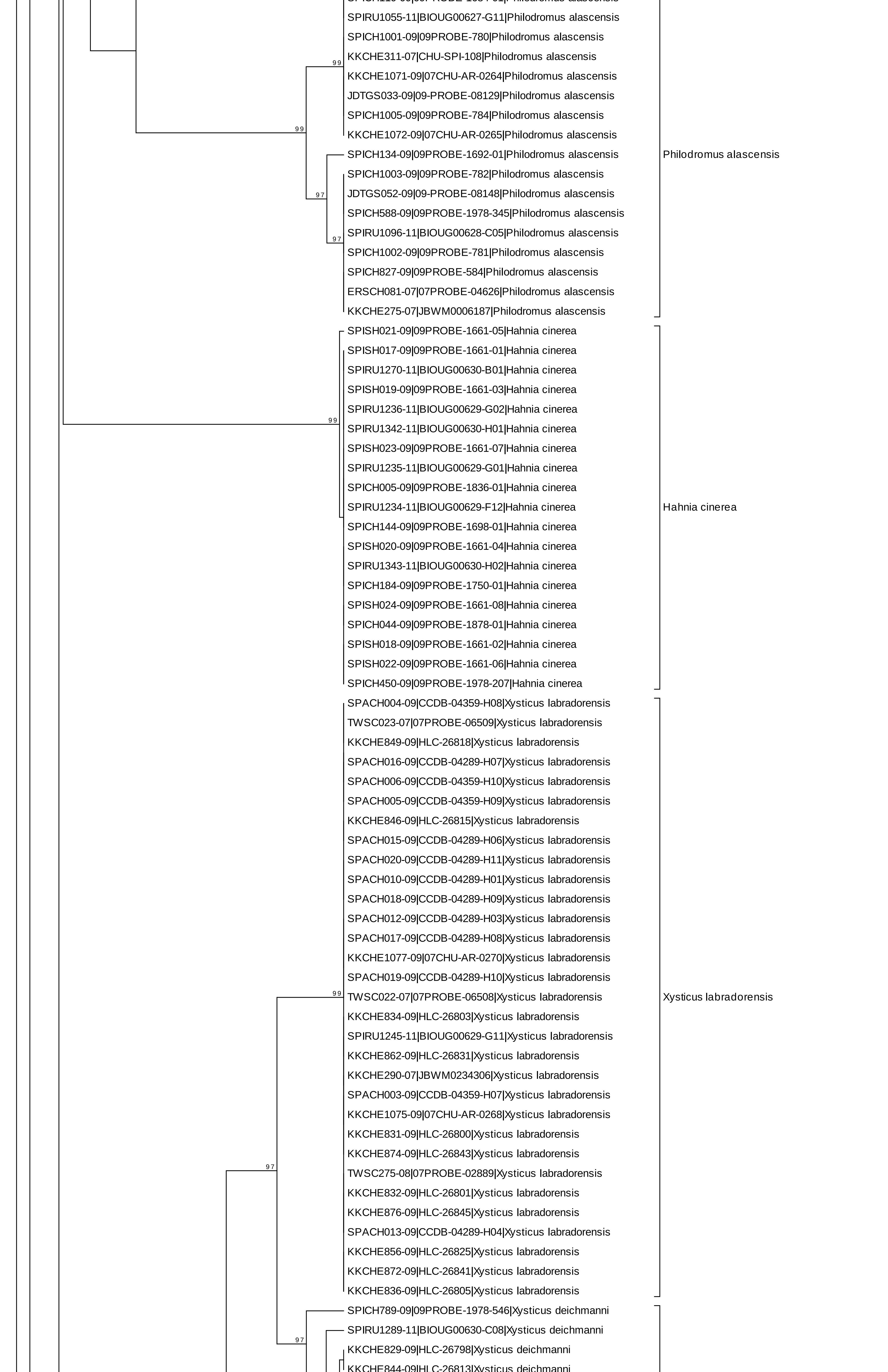
SPIRU1055-11|BIOUG00627-G11|Philodromus alascensis
SPICH1001-09|09PROBE-780|Philodromus alascensis
KKCHE311-07|CHU-SPI-108|Philodromus alascensis
KKCHE1071-09|07CHU-AR-0264|Philodromus alascensis
JDTGS033-09|09-PROBE-08129|Philodromus alascensis
SPICH1005-09|09PROBE-784|Philodromus alascensis
KKCHE1072-09|07CHU-AR-0265|Philodromus alascensis
SPICH134-09|09PROBE-1692-01|Philodromus alascensis
SPICH1003-09|09PROBE-782|Philodromus alascensis
JDTGS052-09|09-PROBE-08148|Philodromus alascensis
SPICH588-09|09PROBE-1978-345|Philodromus alascensis
SPIRU1096-11|BIOUG00628-C05|Philodromus alascensis
SPICH1002-09|09PROBE-781|Philodromus alascensis
SPICH827-09|09PROBE-584|Philodromus alascensis
ERSCH081-07|07PROBE-04626|Philodromus alascensis
KKCHE275-07|JBWM0006187|Philodromus alascensis
Philodromus alascensis
SPISH021-09|09PROBE-1661-05|Hahnia cinerea
SPISH017-09|09PROBE-1661-01|Hahnia cinerea
SPIRU1270-11|BIOUG00630-B01|Hahnia cinerea
SPISH019-09|09PROBE-1661-03|Hahnia cinerea
SPIRU1236-11|BIOUG00629-G02|Hahnia cinerea
SPIRU1342-11|BIOUG00630-H01|Hahnia cinerea
SPISH023-09|09PROBE-1661-07|Hahnia cinerea
SPIRU1235-11|BIOUG00629-G01|Hahnia cinerea
SPICH005-09|09PROBE-1836-01|Hahnia cinerea
SPIRU1234-11|BIOUG00629-F12|Hahnia cinerea
SPICH144-09|09PROBE-1698-01|Hahnia cinerea
SPISH020-09|09PROBE-1661-04|Hahnia cinerea
SPIRU1343-11|BIOUG00630-H02|Hahnia cinerea
SPICH184-09|09PROBE-1750-01|Hahnia cinerea
SPISH024-09|09PROBE-1661-08|Hahnia cinerea
SPICH044-09|09PROBE-1878-01|Hahnia cinerea
SPISH018-09|09PROBE-1661-02|Hahnia cinerea
SPISH022-09|09PROBE-1661-06|Hahnia cinerea
SPICH450-09|09PROBE-1978-207|Hahnia cinerea
Hahnia cinerea
SPACH004-09|CCDB-04359-H08|Xysticus labradorensis
TWSC023-07|07PROBE-06509|Xysticus labradorensis
KKCHE849-09|HLC-26818|Xysticus labradorensis
SPACH016-09|CCDB-04289-H07|Xysticus labradorensis
SPACH006-09|CCDB-04359-H10|Xysticus labradorensis
SPACH005-09|CCDB-04359-H09|Xysticus labradorensis
KKCHE846-09|HLC-26815|Xysticus labradorensis
SPACH015-09|CCDB-04289-H06|Xysticus labradorensis
SPACH020-09|CCDB-04289-H11|Xysticus labradorensis
SPACH010-09|CCDB-04289-H01|Xysticus labradorensis
SPACH018-09|CCDB-04289-H09|Xysticus labradorensis
SPACH012-09|CCDB-04289-H03|Xysticus labradorensis
SPACH017-09|CCDB-04289-H08|Xysticus labradorensis
KKCHE1077-09|07CHU-AR-0270|Xysticus labradorensis
SPACH019-09|CCDB-04289-H10|Xysticus labradorensis
TWSC022-07|07PROBE-06508|Xysticus labradorensis
KKCHE834-09|HLC-26803|Xysticus labradorensis
SPIRU1245-11|BIOUG00629-G11|Xysticus labradorensis
KKCHE862-09|HLC-26831|Xysticus labradorensis
KKCHE290-07|JBWM0234306|Xysticus labradorensis
SPACH003-09|CCDB-04359-H07|Xysticus labradorensis
KKCHE1075-09|07CHU-AR-0268|Xysticus labradorensis
KKCHE831-09|HLC-26800|Xysticus labradorensis
KKCHE874-09|HLC-26843|Xysticus labradorensis
TWSC275-08|07PROBE-02889|Xysticus labradorensis
KKCHE832-09|HLC-26801|Xysticus labradorensis
KKCHE876-09|HLC-26845|Xysticus labradorensis
SPACH013-09|CCDB-04289-H04|Xysticus labradorensis
KKCHE856-09|HLC-26825|Xysticus labradorensis
KKCHE872-09|HLC-26841|Xysticus labradorensis
KKCHE836-09|HLC-26805|Xysticus labradorensis
Xysticus labradorensis
SPICH789-09|09PROBE-1978-546|Xysticus deichmanni
SPIRU1289-11|BIOUG00630-C08|Xysticus deichmanni
KKCHE829-09|HLC-26798|Xysticus deichmanni
KKCHE844-09|HLC-26813|Xysticus deichmanni

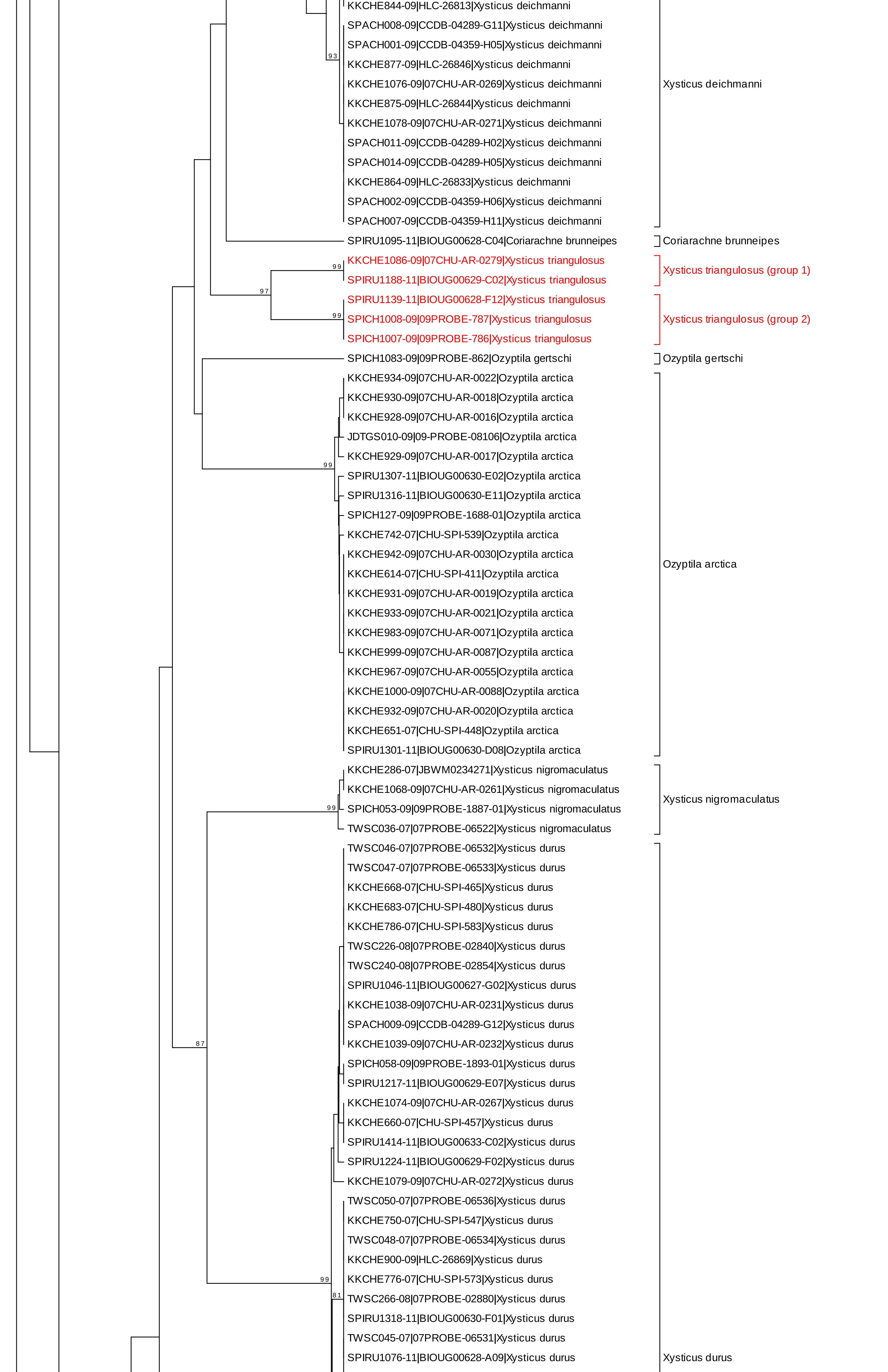
KKCHE844-09|HLC-26813|Xysticus deichmanni
SPACH008-09|CCDB-04289-G11|Xysticus deichmanni
SPACH001-09|CCDB-04359-H05|Xysticus deichmanni
KKCHE877-09|HLC-26846|Xysticus deichmanni
KKCHE1076-09|07CHU-AR-0269|Xysticus deichmanni
KKCHE875-09|HLC-26844|Xysticus deichmanni
KKCHE1078-09|07CHU-AR-0271|Xysticus deichmanni
SPACH011-09|CCDB-04289-H02|Xysticus deichmanni
SPACH014-09|CCDB-04289-H05|Xysticus deichmanni
KKCHE864-09|HLC-26833|Xysticus deichmanni
SPACH002-09|CCDB-04359-H06|Xysticus deichmanni
SPACH007-09|CCDB-04359-H11|Xysticus deichmanni
Xysticus deichmanni
SPIRU1095-11|BIOUG00628-C04|Coriarachne brunneipes
Coriarachne brunneipes
KKCHE1086-09|07CHU-AR-0279|Xysticus triangulosus
SPIRU1188-11|BIOUG00629-C02|Xysticus triangulosus
Xysticus triangulosus (group 1)
SPIRU1139-11|BIOUG00628-F12|Xysticus triangulosus
SPICH1008-09|09PROBE-787|Xysticus triangulosus
SPICH1007-09|09PROBE-786|Xysticus triangulosus
Xysticus triangulosus (group 2)
SPICH1083-09|09PROBE-862|Ozyptila gertschi
Ozyptila gertschi
KKCHE934-09|07CHU-AR-0022|Ozyptila arctica
KKCHE930-09|07CHU-AR-0018|Ozyptila arctica
KKCHE928-09|07CHU-AR-0016|Ozyptila arctica
JDTGS010-09|09-PROBE-08106|Ozyptila arctica
KKCHE929-09|07CHU-AR-0017|Ozyptila arctica
SPIRU1307-11|BIOUG00630-E02|Ozyptila arctica
SPIRU1316-11|BIOUG00630-E11|Ozyptila arctica
SPICH127-09|09PROBE-1688-01|Ozyptila arctica
KKCHE742-07|CHU-SPI-539|Ozyptila arctica
KKCHE942-09|07CHU-AR-0030|Ozyptila arctica
KKCHE614-07|CHU-SPI-411|Ozyptila arctica
KKCHE931-09|07CHU-AR-0019|Ozyptila arctica
KKCHE933-09|07CHU-AR-0021|Ozyptila arctica
KKCHE983-09|07CHU-AR-0071|Ozyptila arctica
KKCHE999-09|07CHU-AR-0087|Ozyptila arctica
KKCHE967-09|07CHU-AR-0055|Ozyptila arctica
KKCHE1000-09|07CHU-AR-0088|Ozyptila arctica
KKCHE932-09|07CHU-AR-0020|Ozyptila arctica
KKCHE651-07|CHU-SPI-448|Ozyptila arctica
SPIRU1301-11|BIOUG00630-D08|Ozyptila arctica
Ozyptila arctica
KKCHE286-07|JBWM0234271|Xysticus nigromaculatus
KKCHE1068-09|07CHU-AR-0261|Xysticus nigromaculatus
SPICH053-09|09PROBE-1887-01|Xysticus nigromaculatus
TWSC036-07|07PROBE-06522|Xysticus nigromaculatus
Xysticus nigromaculatus
TWSC046-07|07PROBE-06532|Xysticus durus
TWSC047-07|07PROBE-06533|Xysticus durus
KKCHE668-07|CHU-SPI-465|Xysticus durus
KKCHE683-07|CHU-SPI-480|Xysticus durus
KKCHE786-07|CHU-SPI-583|Xysticus durus
TWSC226-08|07PROBE-02840|Xysticus durus
TWSC240-08|07PROBE-02854|Xysticus durus
SPIRU1046-11|BIOUG00627-G02|Xysticus durus
KKCHE1038-09|07CHU-AR-0231|Xysticus durus
SPACH009-09|CCDB-04289-G12|Xysticus durus
KKCHE1039-09|07CHU-AR-0232|Xysticus durus
SPICH058-09|09PROBE-1893-01|Xysticus durus
SPIRU1217-11|BIOUG00629-E07|Xysticus durus
KKCHE1074-09|07CHU-AR-0267|Xysticus durus
KKCHE660-07|CHU-SPI-457|Xysticus durus
SPIRU1414-11|BIOUG00633-C02|Xysticus durus
SPIRU1224-11|BIOUG00629-F02|Xysticus durus
KKCHE1079-09|07CHU-AR-0272|Xysticus durus
TWSC050-07|07PROBE-06536|Xysticus durus
KKCHE750-07|CHU-SPI-547|Xysticus durus
TWSC048-07|07PROBE-06534|Xysticus durus
KKCHE900-09|HLC-26869|Xysticus durus
KKCHE776-07|CHU-SPI-573|Xysticus durus
TWSC266-08|07PROBE-02880|Xysticus durus
SPIRU1318-11|BIOUG00630-F01|Xysticus durus
TWSC045-07|07PROBE-06531|Xysticus durus
SPIRU1076-11|BIOUG00628-A09|Xysticus durus
Xysticus durus

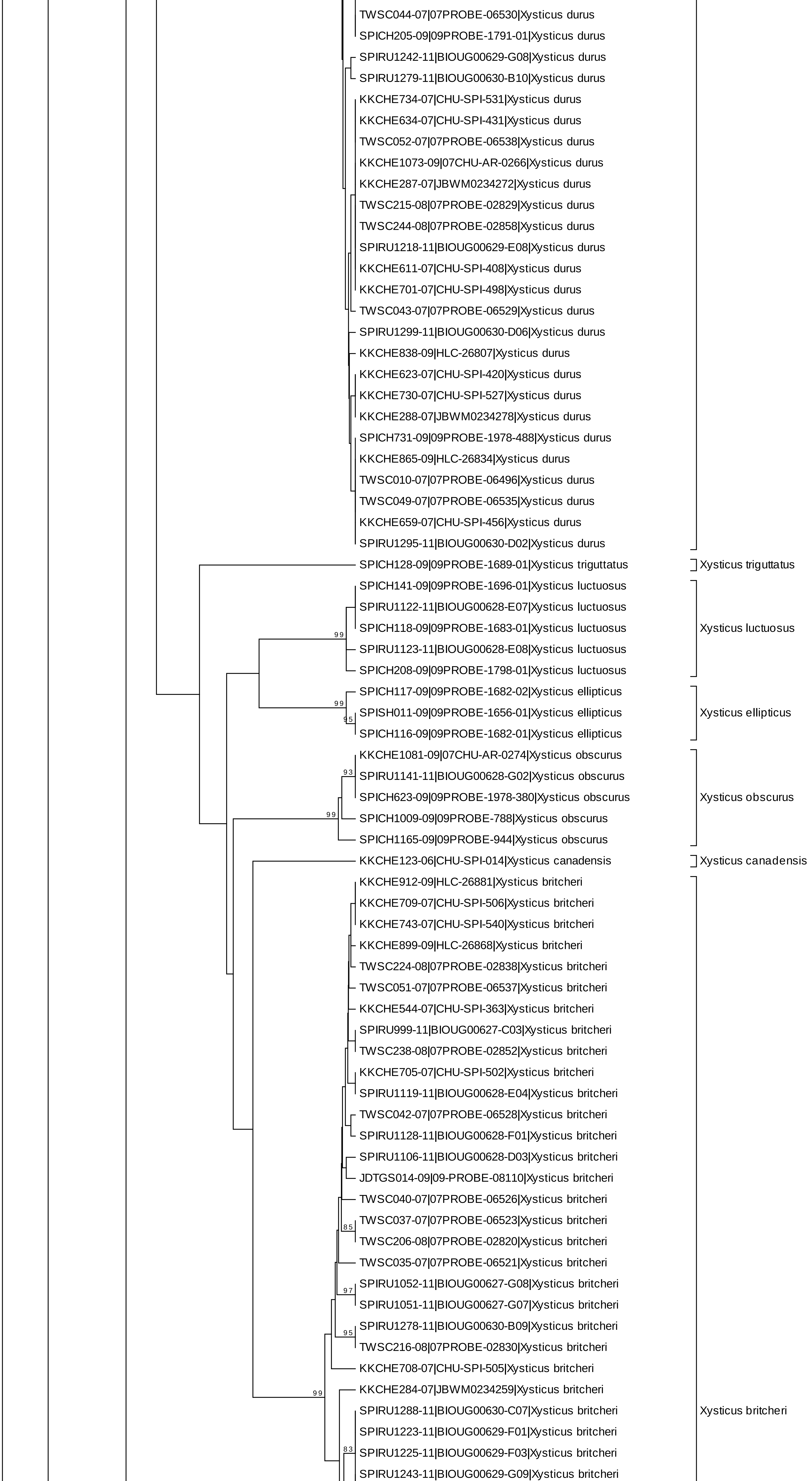
TWSC044-07|07PROBE-06530|Xysticus durus
SPICH205-09|09PROBE-1791-01|Xysticus durus
SPIRU1242-11|BIOUG00629-G08|Xysticus durus
SPIRU1279-11|BIOUG00630-B10|Xysticus durus
KKCHE734-07|CHU-SPI-531|Xysticus durus
KKCHE634-07|CHU-SPI-431|Xysticus durus
TWSC052-07|07PROBE-06538|Xysticus durus
KKCHE1073-09|07CHU-AR-0266|Xysticus durus
KKCHE287-07|JBWM0234272|Xysticus durus
TWSC215-08|07PROBE-02829|Xysticus durus
TWSC244-08|07PROBE-02858|Xysticus durus
SPIRU1218-11|BIOUG00629-E08|Xysticus durus
KKCHE611-07|CHU-SPI-408|Xysticus durus
KKCHE701-07|CHU-SPI-498|Xysticus durus
TWSC043-07|07PROBE-06529|Xysticus durus
SPIRU1299-11|BIOUG00630-D06|Xysticus durus
KKCHE838-09|HLC-26807|Xysticus durus
KKCHE623-07|CHU-SPI-420|Xysticus durus
KKCHE730-07|CHU-SPI-527|Xysticus durus
KKCHE288-07|JBWM0234278|Xysticus durus
SPICH731-09|09PROBE-1978-488|Xysticus durus
KKCHE865-09|HLC-26834|Xysticus durus
TWSC010-07|07PROBE-06496|Xysticus durus
TWSC049-07|07PROBE-06535|Xysticus durus
KKCHE659-07|CHU-SPI-456|Xysticus durus
SPIRU1295-11|BIOUG00630-D02|Xysticus durus
SPICH128-09|09PROBE-1689-01|Xysticus triguttatus
Xysticus triguttatus
SPICH141-09|09PROBE-1696-01|Xysticus luctuosus
SPIRU1122-11|BIOUG00628-E07|Xysticus luctuosus
99
SPICH118-09|09PROBE-1683-01|Xysticus luctuosus
SPIRU1123-11|BIOUG00628-E08|Xysticus luctuosus
SPICH208-09|09PROBE-1798-01|Xysticus luctuosus
Xysticus luctuosus
SPICH117-09|09PROBE-1682-02|Xysticus ellipticus
99
95
SPISH011-09|09PROBE-1656-01|Xysticus ellipticus
SPICH116-09|09PROBE-1682-01|Xysticus ellipticus
Xysticus ellipticus
KKCHE1081-09|07CHU-AR-0274|Xysticus obscurus
93
SPIRU1141-11|BIOUG00628-G02|Xysticus obscurus
SPICH623-09|09PROBE-1978-380|Xysticus obscurus
99
SPICH1009-09|09PROBE-788|Xysticus obscurus
SPICH1165-09|09PROBE-944|Xysticus obscurus
Xysticus obscurus
KKCHE123-06|CHU-SPI-014|Xysticus canadensis
Xysticus canadensis
KKCHE912-09|HLC-26881|Xysticus britcheri
KKCHE709-07|CHU-SPI-506|Xysticus britcheri
KKCHE743-07|CHU-SPI-540|Xysticus britcheri
KKCHE899-09|HLC-26868|Xysticus britcheri
TWSC224-08|07PROBE-02838|Xysticus britcheri
TWSC051-07|07PROBE-06537|Xysticus britcheri
KKCHE544-07|CHU-SPI-363|Xysticus britcheri
SPIRU999-11|BIOUG00627-C03|Xysticus britcheri
TWSC238-08|07PROBE-02852|Xysticus britcheri
KKCHE705-07|CHU-SPI-502|Xysticus britcheri
SPIRU1119-11|BIOUG00628-E04|Xysticus britcheri
TWSC042-07|07PROBE-06528|Xysticus britcheri
SPIRU1128-11|BIOUG00628-F01|Xysticus britcheri
SPIRU1106-11|BIOUG00628-D03|Xysticus britcheri
JDTGS014-09|09-PROBE-08110|Xysticus britcheri
TWSC040-07|07PROBE-06526|Xysticus britcheri
TWSC037-07|07PROBE-06523|Xysticus britcheri
85
TWSC206-08|07PROBE-02820|Xysticus britcheri
TWSC035-07|07PROBE-06521|Xysticus britcheri
SPIRU1052-11|BIOUG00627-G08|Xysticus britcheri
97
SPIRU1051-11|BIOUG00627-G07|Xysticus britcheri
SPIRU1278-11|BIOUG00630-B09|Xysticus britcheri
95
TWSC216-08|07PROBE-02830|Xysticus britcheri
KKCHE708-07|CHU-SPI-505|Xysticus britcheri
KKCHE284-07|JBWM0234259|Xysticus britcheri
99
SPIRU1288-11|BIOUG00630-C07|Xysticus britcheri
Xysticus britcheri
SPIRU1223-11|BIOUG00629-F01|Xysticus britcheri
83
SPIRU1225-11|BIOUG00629-F03|Xysticus britcheri
SPIRU1243-11|BIOUG00629-G09|Xysticus britcheri

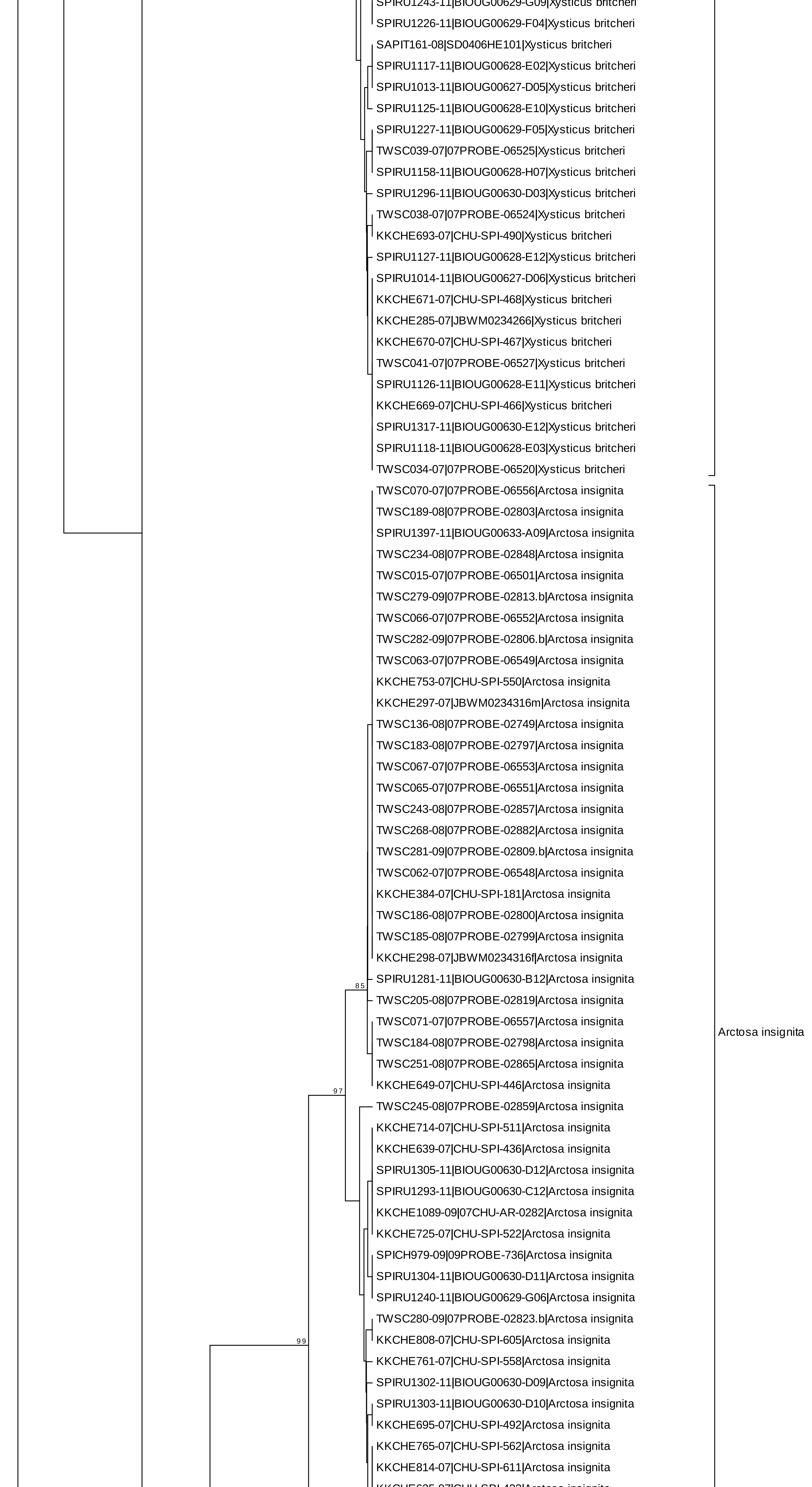
SPIRU1243-11|BIOUG00629-G09|Xysticus britcheri
SPIRU1226-11|BIOUG00629-F04|Xysticus britcheri
SAPIT161-08|SD0406HE101|Xysticus britcheri
SPIRU1117-11|BIOUG00628-E02|Xysticus britcheri
SPIRU1013-11|BIOUG00627-D05|Xysticus britcheri
SPIRU1125-11|BIOUG00628-E10|Xysticus britcheri
SPIRU1227-11|BIOUG00629-F05|Xysticus britcheri
TWSC039-07|07PROBE-06525|Xysticus britcheri
SPIRU1158-11|BIOUG00628-H07|Xysticus britcheri
SPIRU1296-11|BIOUG00630-D03|Xysticus britcheri
TWSC038-07|07PROBE-06524|Xysticus britcheri
KKCHE693-07|CHU-SPI-490|Xysticus britcheri
SPIRU1127-11|BIOUG00628-E12|Xysticus britcheri
SPIRU1014-11|BIOUG00627-D06|Xysticus britcheri
KKCHE671-07|CHU-SPI-468|Xysticus britcheri
KKCHE285-07|JBWM0234266|Xysticus britcheri
KKCHE670-07|CHU-SPI-467|Xysticus britcheri
TWSC041-07|07PROBE-06527|Xysticus britcheri
SPIRU1126-11|BIOUG00628-E11|Xysticus britcheri
KKCHE669-07|CHU-SPI-466|Xysticus britcheri
SPIRU1317-11|BIOUG00630-E12|Xysticus britcheri
SPIRU1118-11|BIOUG00628-E03|Xysticus britcheri
TWSC034-07|07PROBE-06520|Xysticus britcheri
TWSC070-07|07PROBE-06556|Arctosa insignita
TWSC189-08|07PROBE-02803|Arctosa insignita
SPIRU1397-11|BIOUG00633-A09|Arctosa insignita
TWSC234-08|07PROBE-02848|Arctosa insignita
TWSC015-07|07PROBE-06501|Arctosa insignita
TWSC279-09|07PROBE-02813.b|Arctosa insignita
TWSC066-07|07PROBE-06552|Arctosa insignita
TWSC282-09|07PROBE-02806.b|Arctosa insignita
TWSC063-07|07PROBE-06549|Arctosa insignita
KKCHE753-07|CHU-SPI-550|Arctosa insignita
KKCHE297-07|JBWM0234316m|Arctosa insignita
TWSC136-08|07PROBE-02749|Arctosa insignita
TWSC183-08|07PROBE-02797|Arctosa insignita
TWSC067-07|07PROBE-06553|Arctosa insignita
TWSC065-07|07PROBE-06551|Arctosa insignita
TWSC243-08|07PROBE-02857|Arctosa insignita
TWSC268-08|07PROBE-02882|Arctosa insignita
TWSC281-09|07PROBE-02809.b|Arctosa insignita
TWSC062-07|07PROBE-06548|Arctosa insignita
KKCHE384-07|CHU-SPI-181|Arctosa insignita
TWSC186-08|07PROBE-02800|Arctosa insignita
TWSC185-08|07PROBE-02799|Arctosa insignita
KKCHE298-07|JBWM0234316f|Arctosa insignita
SPIRU1281-11|BIOUG00630-B12|Arctosa insignita
85
TWSC205-08|07PROBE-02819|Arctosa insignita
TWSC071-07|07PROBE-06557|Arctosa insignita
TWSC184-08|07PROBE-02798|Arctosa insignita
Arctosa insignita
TWSC251-08|07PROBE-02865|Arctosa insignita
KKCHE649-07|CHU-SPI-446|Arctosa insignita
97
TWSC245-08|07PROBE-02859|Arctosa insignita
KKCHE714-07|CHU-SPI-511|Arctosa insignita
KKCHE639-07|CHU-SPI-436|Arctosa insignita
SPIRU1305-11|BIOUG00630-D12|Arctosa insignita
SPIRU1293-11|BIOUG00630-C12|Arctosa insignita
KKCHE1089-09|07CHU-AR-0282|Arctosa insignita
KKCHE725-07|CHU-SPI-522|Arctosa insignita
SPICH979-09|09PROBE-736|Arctosa insignita
SPIRU1304-11|BIOUG00630-D11|Arctosa insignita
SPIRU1240-11|BIOUG00629-G06|Arctosa insignita
TWSC280-09|07PROBE-02823.b|Arctosa insignita
99
KKCHE808-07|CHU-SPI-605|Arctosa insignita
KKCHE761-07|CHU-SPI-558|Arctosa insignita
SPIRU1302-11|BIOUG00630-D09|Arctosa insignita
SPIRU1303-11|BIOUG00630-D10|Arctosa insignita
KKCHE695-07|CHU-SPI-492|Arctosa insignita
KKCHE765-07|CHU-SPI-562|Arctosa insignita
KKCHE814-07|CHU-SPI-611|Arctosa insignita

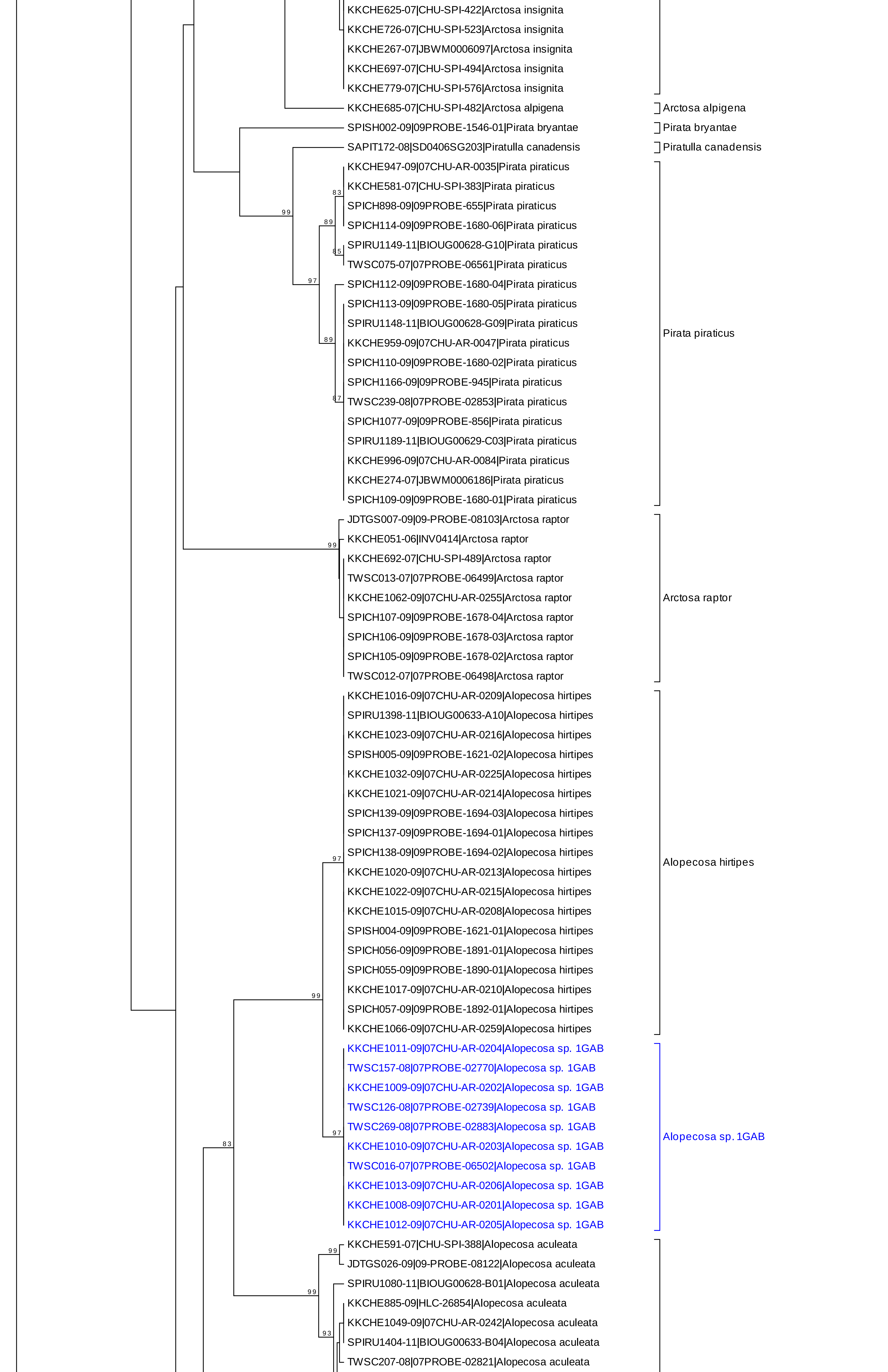
KKCHE625-07|CHU-SPI-422|Arctosa insignita
KKCHE726-07|CHU-SPI-523|Arctosa insignita
KKCHE267-07|JBWM0006097|Arctosa insignita
KKCHE697-07|CHU-SPI-494|Arctosa insignita
KKCHE779-07|CHU-SPI-576|Arctosa insignita
KKCHE685-07|CHU-SPI-482|Arctosa alpigena
Arctosa alpigena
SPISH002-09|09PROBE-1546-01|Pirata bryantae
Pirata bryantae
SAPIT172-08|SD0406SG203|Piratulla canadensis
Piratulla canadensis
KKCHE947-09|07CHU-AR-0035|Pirata piraticus
KKCHE581-07|CHU-SPI-383|Pirata piraticus
SPICH898-09|09PROBE-655|Pirata piraticus
SPICH114-09|09PROBE-1680-06|Pirata piraticus
SPIRU1149-11|BIOUG00628-G10|Pirata piraticus
TWSC075-07|07PROBE-06561|Pirata piraticus
SPICH112-09|09PROBE-1680-04|Pirata piraticus
SPICH113-09|09PROBE-1680-05|Pirata piraticus
SPIRU1148-11|BIOUG00628-G09|Pirata piraticus
KKCHE959-09|07CHU-AR-0047|Pirata piraticus
SPICH110-09|09PROBE-1680-02|Pirata piraticus
SPICH1166-09|09PROBE-945|Pirata piraticus
TWSC239-08|07PROBE-02853|Pirata piraticus
SPICH1077-09|09PROBE-856|Pirata piraticus
SPIRU1189-11|BIOUG00629-C03|Pirata piraticus
KKCHE996-09|07CHU-AR-0084|Pirata piraticus
KKCHE274-07|JBWM0006186|Pirata piraticus
SPICH109-09|09PROBE-1680-01|Pirata piraticus
Pirata piraticus
JDTGS007-09|09-PROBE-08103|Arctosa raptor
KKCHE051-06|INV0414|Arctosa raptor
KKCHE692-07|CHU-SPI-489|Arctosa raptor
TWSC013-07|07PROBE-06499|Arctosa raptor
KKCHE1062-09|07CHU-AR-0255|Arctosa raptor
SPICH107-09|09PROBE-1678-04|Arctosa raptor
SPICH106-09|09PROBE-1678-03|Arctosa raptor
SPICH105-09|09PROBE-1678-02|Arctosa raptor
TWSC012-07|07PROBE-06498|Arctosa raptor
Arctosa raptor
KKCHE1016-09|07CHU-AR-0209|Alopecosa hirtipes
SPIRU1398-11|BIOUG00633-A10|Alopecosa hirtipes
KKCHE1023-09|07CHU-AR-0216|Alopecosa hirtipes
SPISH005-09|09PROBE-1621-02|Alopecosa hirtipes
KKCHE1032-09|07CHU-AR-0225|Alopecosa hirtipes
KKCHE1021-09|07CHU-AR-0214|Alopecosa hirtipes
SPICH139-09|09PROBE-1694-03|Alopecosa hirtipes
SPICH137-09|09PROBE-1694-01|Alopecosa hirtipes
SPICH138-09|09PROBE-1694-02|Alopecosa hirtipes
KKCHE1020-09|07CHU-AR-0213|Alopecosa hirtipes
KKCHE1022-09|07CHU-AR-0215|Alopecosa hirtipes
KKCHE1015-09|07CHU-AR-0208|Alopecosa hirtipes
SPISH004-09|09PROBE-1621-01|Alopecosa hirtipes
SPICH056-09|09PROBE-1891-01|Alopecosa hirtipes
SPICH055-09|09PROBE-1890-01|Alopecosa hirtipes
KKCHE1017-09|07CHU-AR-0210|Alopecosa hirtipes
SPICH057-09|09PROBE-1892-01|Alopecosa hirtipes
KKCHE1066-09|07CHU-AR-0259|Alopecosa hirtipes
Alopecosa hirtipes
KKCHE1011-09|07CHU-AR-0204|Alopecosa sp. 1GAB
TWSC157-08|07PROBE-02770|Alopecosa sp. 1GAB
KKCHE1009-09|07CHU-AR-0202|Alopecosa sp. 1GAB
TWSC126-08|07PROBE-02739|Alopecosa sp. 1GAB
TWSC269-08|07PROBE-02883|Alopecosa sp. 1GAB
KKCHE1010-09|07CHU-AR-0203|Alopecosa sp. 1GAB
TWSC016-07|07PROBE-06502|Alopecosa sp. 1GAB
KKCHE1013-09|07CHU-AR-0206|Alopecosa sp. 1GAB
KKCHE1008-09|07CHU-AR-0201|Alopecosa sp. 1GAB
KKCHE1012-09|07CHU-AR-0205|Alopecosa sp. 1GAB
Alopecosa sp. 1GAB
KKCHE591-07|CHU-SPI-388|Alopecosa aculeata
JDTGS026-09|09-PROBE-08122|Alopecosa aculeata
SPIRU1080-11|BIOUG00628-B01|Alopecosa aculeata
KKCHE885-09|HLC-26854|Alopecosa aculeata
KKCHE1049-09|07CHU-AR-0242|Alopecosa aculeata
SPIRU1404-11|BIOUG00633-B04|Alopecosa aculeata
TWSC207-08|07PROBE-02821|Alopecosa aculeata

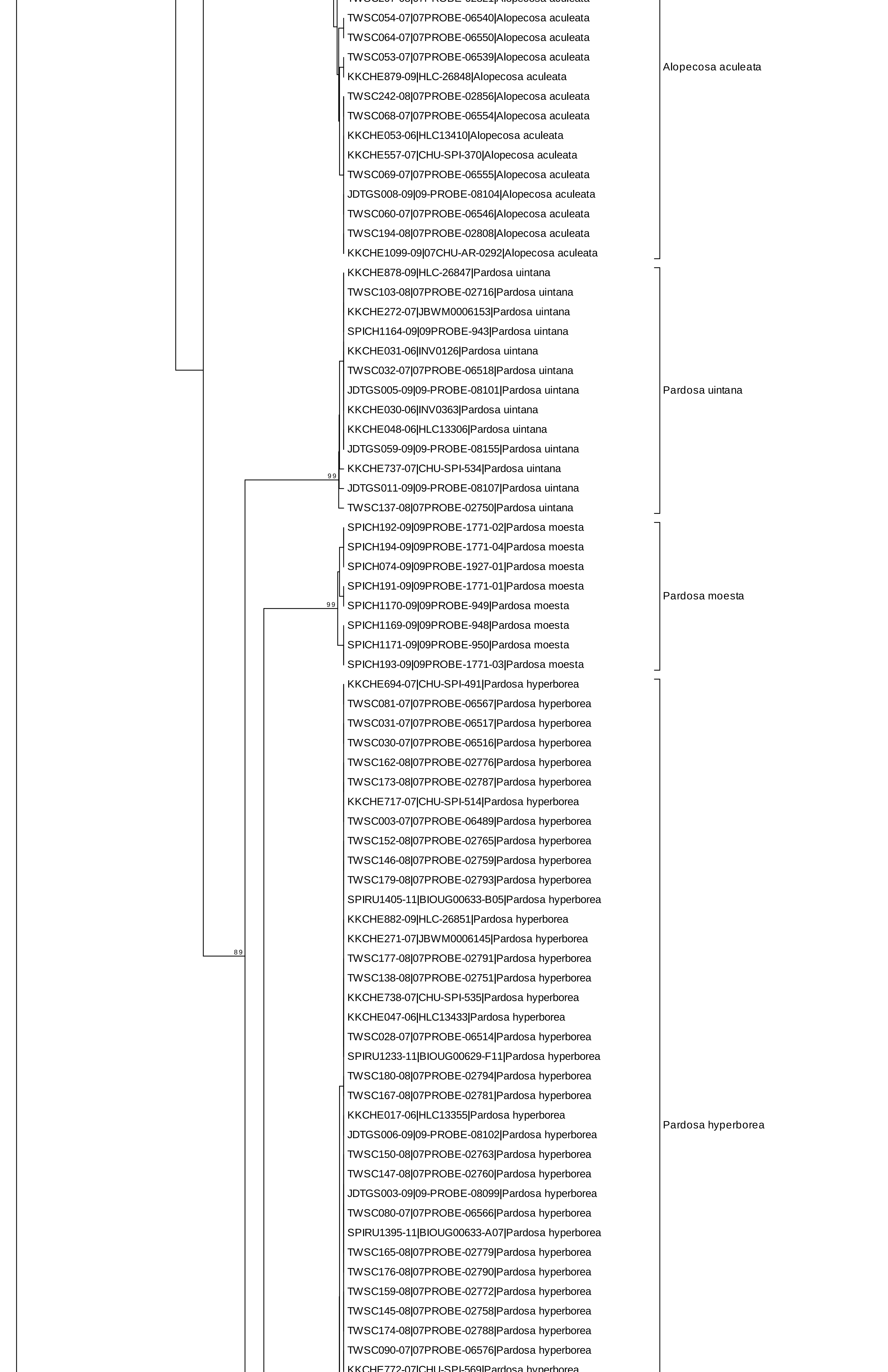
TWSC054-07|07PROBE-06540|Alopecosa aculeata
TWSC064-07|07PROBE-06550|Alopecosa aculeata
TWSC053-07|07PROBE-06539|Alopecosa aculeata
KKCHE879-09|HLC-26848|Alopecosa aculeata
TWSC242-08|07PROBE-02856|Alopecosa aculeata
TWSC068-07|07PROBE-06554|Alopecosa aculeata
KKCHE053-06|HLC13410|Alopecosa aculeata
KKCHE557-07|CHU-SPI-370|Alopecosa aculeata
TWSC069-07|07PROBE-06555|Alopecosa aculeata
JDTGS008-09|09-PROBE-08104|Alopecosa aculeata
TWSC060-07|07PROBE-06546|Alopecosa aculeata
TWSC194-08|07PROBE-02808|Alopecosa aculeata
KKCHE1099-09|07CHU-AR-0292|Alopecosa aculeata
Alopecosa aculeata
KKCHE878-09|HLC-26847|Pardosa uintana
TWSC103-08|07PROBE-02716|Pardosa uintana
KKCHE272-07|JBWM0006153|Pardosa uintana
SPICH1164-09|09PROBE-943|Pardosa uintana
KKCHE031-06|INV0126|Pardosa uintana
TWSC032-07|07PROBE-06518|Pardosa uintana
JDTGS005-09|09-PROBE-08101|Pardosa uintana
KKCHE030-06|INV0363|Pardosa uintana
KKCHE048-06|HLC13306|Pardosa uintana
JDTGS059-09|09-PROBE-08155|Pardosa uintana
KKCHE737-07|CHU-SPI-534|Pardosa uintana
99
JDTGS011-09|09-PROBE-08107|Pardosa uintana
TWSC137-08|07PROBE-02750|Pardosa uintana
Pardosa uintana
SPICH192-09|09PROBE-1771-02|Pardosa moesta
SPICH194-09|09PROBE-1771-04|Pardosa moesta
SPICH074-09|09PROBE-1927-01|Pardosa moesta
SPICH191-09|09PROBE-1771-01|Pardosa moesta
99
SPICH1170-09|09PROBE-949|Pardosa moesta
SPICH1169-09|09PROBE-948|Pardosa moesta
SPICH1171-09|09PROBE-950|Pardosa moesta
SPICH193-09|09PROBE-1771-03|Pardosa moesta
Pardosa moesta
KKCHE694-07|CHU-SPI-491|Pardosa hyperborea
TWSC081-07|07PROBE-06567|Pardosa hyperborea
TWSC031-07|07PROBE-06517|Pardosa hyperborea
TWSC030-07|07PROBE-06516|Pardosa hyperborea
TWSC162-08|07PROBE-02776|Pardosa hyperborea
TWSC173-08|07PROBE-02787|Pardosa hyperborea
KKCHE717-07|CHU-SPI-514|Pardosa hyperborea
TWSC003-07|07PROBE-06489|Pardosa hyperborea
TWSC152-08|07PROBE-02765|Pardosa hyperborea
TWSC146-08|07PROBE-02759|Pardosa hyperborea
TWSC179-08|07PROBE-02793|Pardosa hyperborea
SPIRU1405-11|BIOUG00633-B05|Pardosa hyperborea
KKCHE882-09|HLC-26851|Pardosa hyperborea
KKCHE271-07|JBWM0006145|Pardosa hyperborea
89
TWSC177-08|07PROBE-02791|Pardosa hyperborea
TWSC138-08|07PROBE-02751|Pardosa hyperborea
KKCHE738-07|CHU-SPI-535|Pardosa hyperborea
KKCHE047-06|HLC13433|Pardosa hyperborea
TWSC028-07|07PROBE-06514|Pardosa hyperborea
SPIRU1233-11|BIOUG00629-F11|Pardosa hyperborea
TWSC180-08|07PROBE-02794|Pardosa hyperborea
TWSC167-08|07PROBE-02781|Pardosa hyperborea
KKCHE017-06|HLC13355|Pardosa hyperborea
JDTGS006-09|09-PROBE-08102|Pardosa hyperborea
Pardosa hyperborea
TWSC150-08|07PROBE-02763|Pardosa hyperborea
TWSC147-08|07PROBE-02760|Pardosa hyperborea
JDTGS003-09|09-PROBE-08099|Pardosa hyperborea
TWSC080-07|07PROBE-06566|Pardosa hyperborea
SPIRU1395-11|BIOUG00633-A07|Pardosa hyperborea
TWSC165-08|07PROBE-02779|Pardosa hyperborea
TWSC176-08|07PROBE-02790|Pardosa hyperborea
TWSC159-08|07PROBE-02772|Pardosa hyperborea
TWSC145-08|07PROBE-02758|Pardosa hyperborea
TWSC174-08|07PROBE-02788|Pardosa hyperborea
TWSC090-07|07PROBE-06576|Pardosa hyperborea
KKCHE772-07|CHU-SPI-569|Pardosa hyperborea

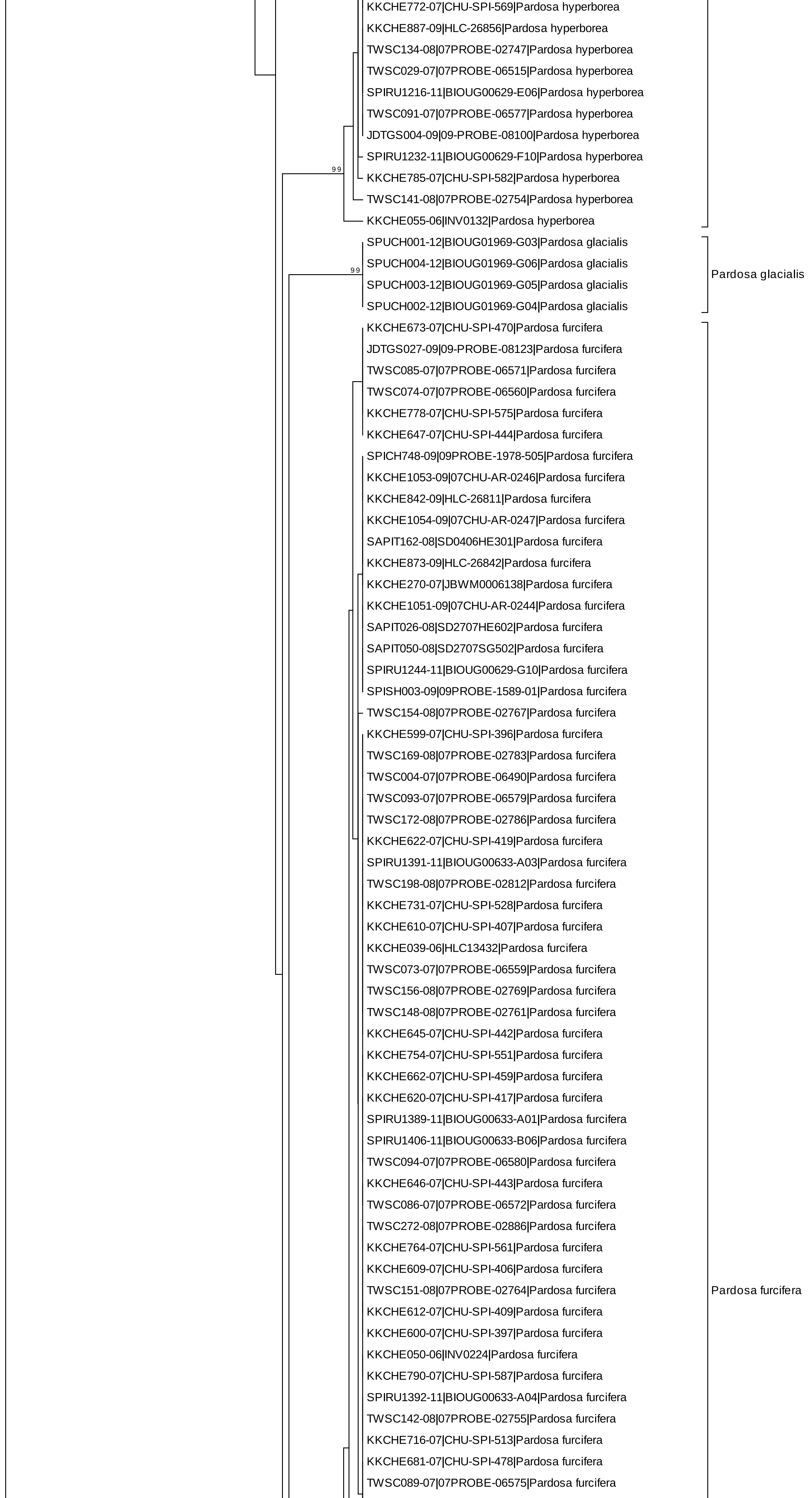
KKCHE772-07|CHU-SPI-569|Pardosa hyperborea
KKCHE887-09|HLC-26856|Pardosa hyperborea
TWSC134-08|07PROBE-02747|Pardosa hyperborea
TWSC029-07|07PROBE-06515|Pardosa hyperborea
SPIRU1216-11|BIOUG00629-E06|Pardosa hyperborea
TWSC091-07|07PROBE-06577|Pardosa hyperborea
JDTGS004-09|09-PROBE-08100|Pardosa hyperborea
SPIRU1232-11|BIOUG00629-F10|Pardosa hyperborea
99
KKCHE785-07|CHU-SPI-582|Pardosa hyperborea
TWSC141-08|07PROBE-02754|Pardosa hyperborea
KKCHE055-06|INV0132|Pardosa hyperborea
SPUCH001-12|BIOUG01969-G03|Pardosa glacialis
SPUCH004-12|BIOUG01969-G06|Pardosa glacialis
99
SPUCH003-12|BIOUG01969-G05|Pardosa glacialis
SPUCH002-12|BIOUG01969-G04|Pardosa glacialis
Pardosa glacialis
KKCHE673-07|CHU-SPI-470|Pardosa furcifera
JDTGS027-09|09-PROBE-08123|Pardosa furcifera
TWSC085-07|07PROBE-06571|Pardosa furcifera
TWSC074-07|07PROBE-06560|Pardosa furcifera
KKCHE778-07|CHU-SPI-575|Pardosa furcifera
KKCHE647-07|CHU-SPI-444|Pardosa furcifera
SPICH748-09|09PROBE-1978-505|Pardosa furcifera
KKCHE1053-09|07CHU-AR-0246|Pardosa furcifera
KKCHE842-09|HLC-26811|Pardosa furcifera
KKCHE1054-09|07CHU-AR-0247|Pardosa furcifera
SAPIT162-08|SD0406HE301|Pardosa furcifera
KKCHE873-09|HLC-26842|Pardosa furcifera
KKCHE270-07|JBWM0006138|Pardosa furcifera
KKCHE1051-09|07CHU-AR-0244|Pardosa furcifera
SAPIT026-08|SD2707HE602|Pardosa furcifera
SAPIT050-08|SD2707SG502|Pardosa furcifera
SPIRU1244-11|BIOUG00629-G10|Pardosa furcifera
SPISH003-09|09PROBE-1589-01|Pardosa furcifera
TWSC154-08|07PROBE-02767|Pardosa furcifera
KKCHE599-07|CHU-SPI-396|Pardosa furcifera
TWSC169-08|07PROBE-02783|Pardosa furcifera
TWSC004-07|07PROBE-06490|Pardosa furcifera
TWSC093-07|07PROBE-06579|Pardosa furcifera
TWSC172-08|07PROBE-02786|Pardosa furcifera
KKCHE622-07|CHU-SPI-419|Pardosa furcifera
SPIRU1391-11|BIOUG00633-A03|Pardosa furcifera
TWSC198-08|07PROBE-02812|Pardosa furcifera
KKCHE731-07|CHU-SPI-528|Pardosa furcifera
KKCHE610-07|CHU-SPI-407|Pardosa furcifera
KKCHE039-06|HLC13432|Pardosa furcifera
TWSC073-07|07PROBE-06559|Pardosa furcifera
TWSC156-08|07PROBE-02769|Pardosa furcifera
TWSC148-08|07PROBE-02761|Pardosa furcifera
KKCHE645-07|CHU-SPI-442|Pardosa furcifera
KKCHE754-07|CHU-SPI-551|Pardosa furcifera
KKCHE662-07|CHU-SPI-459|Pardosa furcifera
KKCHE620-07|CHU-SPI-417|Pardosa furcifera
SPIRU1389-11|BIOUG00633-A01|Pardosa furcifera
SPIRU1406-11|BIOUG00633-B06|Pardosa furcifera
TWSC094-07|07PROBE-06580|Pardosa furcifera
KKCHE646-07|CHU-SPI-443|Pardosa furcifera
TWSC086-07|07PROBE-06572|Pardosa furcifera
TWSC272-08|07PROBE-02886|Pardosa furcifera
KKCHE764-07|CHU-SPI-561|Pardosa furcifera
KKCHE609-07|CHU-SPI-406|Pardosa furcifera
TWSC151-08|07PROBE-02764|Pardosa furcifera
Pardosa furcifera
KKCHE612-07|CHU-SPI-409|Pardosa furcifera
KKCHE600-07|CHU-SPI-397|Pardosa furcifera
KKCHE050-06|INV0224|Pardosa furcifera
KKCHE790-07|CHU-SPI-587|Pardosa furcifera
SPIRU1392-11|BIOUG00633-A04|Pardosa furcifera
TWSC142-08|07PROBE-02755|Pardosa furcifera
KKCHE716-07|CHU-SPI-513|Pardosa furcifera
KKCHE681-07|CHU-SPI-478|Pardosa furcifera
TWSC089-07|07PROBE-06575|Pardosa furcifera

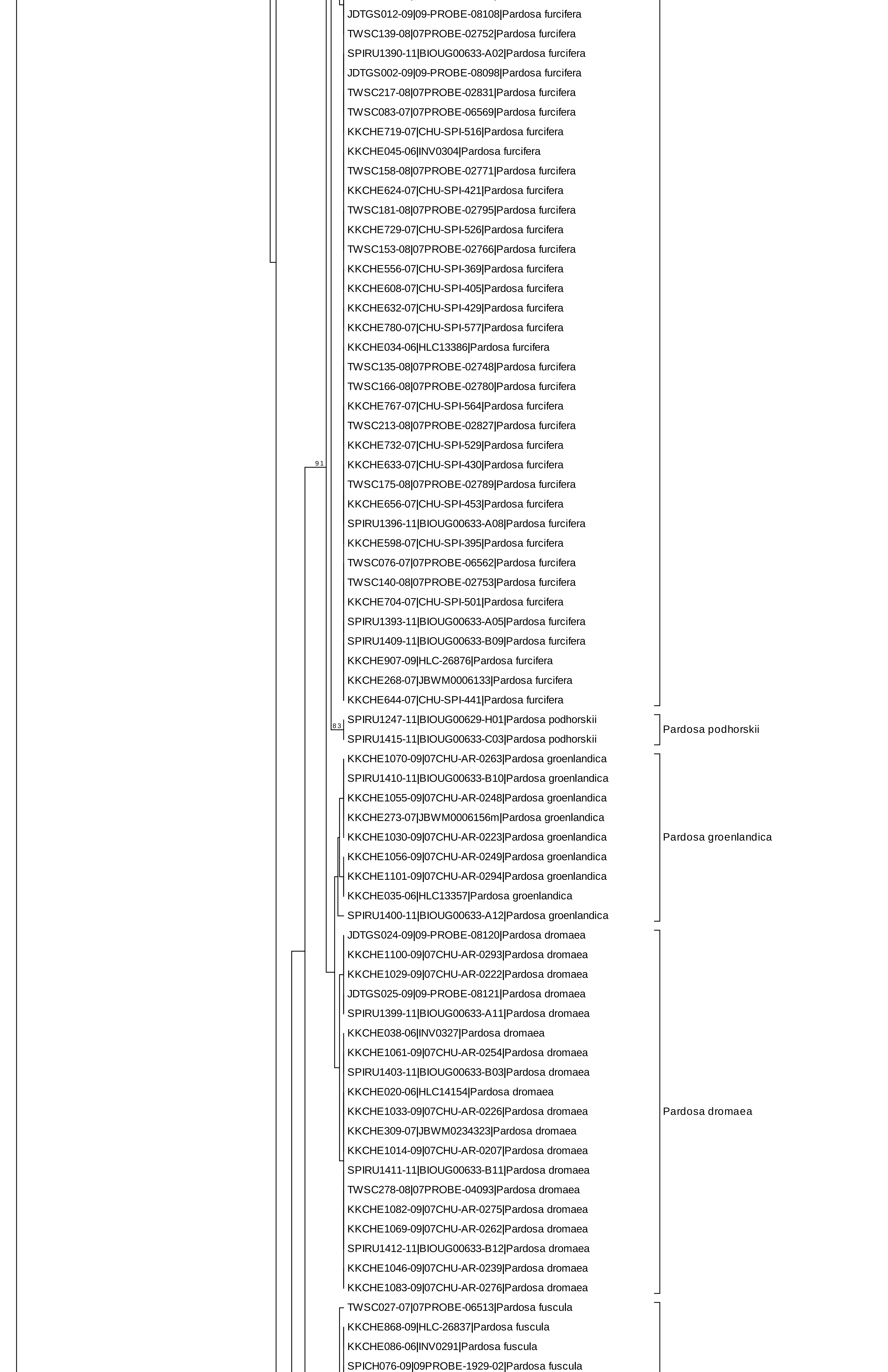
JDTGS012-09|09-PROBE-08108|Pardosa furcifera
TWSC139-08|07PROBE-02752|Pardosa furcifera
SPIRU1390-11|BIOUG00633-A02|Pardosa furcifera
JDTGS002-09|09-PROBE-08098|Pardosa furcifera
TWSC217-08|07PROBE-02831|Pardosa furcifera
TWSC083-07|07PROBE-06569|Pardosa furcifera
KKCHE719-07|CHU-SPI-516|Pardosa furcifera
KKCHE045-06|INV0304|Pardosa furcifera
TWSC158-08|07PROBE-02771|Pardosa furcifera
KKCHE624-07|CHU-SPI-421|Pardosa furcifera
TWSC181-08|07PROBE-02795|Pardosa furcifera
KKCHE729-07|CHU-SPI-526|Pardosa furcifera
TWSC153-08|07PROBE-02766|Pardosa furcifera
KKCHE556-07|CHU-SPI-369|Pardosa furcifera
KKCHE608-07|CHU-SPI-405|Pardosa furcifera
KKCHE632-07|CHU-SPI-429|Pardosa furcifera
KKCHE780-07|CHU-SPI-577|Pardosa furcifera
KKCHE034-06|HLC13386|Pardosa furcifera
TWSC135-08|07PROBE-02748|Pardosa furcifera
TWSC166-08|07PROBE-02780|Pardosa furcifera
KKCHE767-07|CHU-SPI-564|Pardosa furcifera
TWSC213-08|07PROBE-02827|Pardosa furcifera
KKCHE732-07|CHU-SPI-529|Pardosa furcifera
91
KKCHE633-07|CHU-SPI-430|Pardosa furcifera
TWSC175-08|07PROBE-02789|Pardosa furcifera
KKCHE656-07|CHU-SPI-453|Pardosa furcifera
SPIRU1396-11|BIOUG00633-A08|Pardosa furcifera
KKCHE598-07|CHU-SPI-395|Pardosa furcifera
TWSC076-07|07PROBE-06562|Pardosa furcifera
TWSC140-08|07PROBE-02753|Pardosa furcifera
KKCHE704-07|CHU-SPI-501|Pardosa furcifera
SPIRU1393-11|BIOUG00633-A05|Pardosa furcifera
SPIRU1409-11|BIOUG00633-B09|Pardosa furcifera
KKCHE907-09|HLC-26876|Pardosa furcifera
KKCHE268-07|JBWM0006133|Pardosa furcifera
KKCHE644-07|CHU-SPI-441|Pardosa furcifera
SPIRU1247-11|BIOUG00629-H01|Pardosa podhorskii
83
SPIRU1415-11|BIOUG00633-C03|Pardosa podhorskii
Pardosa podhorskii
KKCHE1070-09|07CHU-AR-0263|Pardosa groenlandica
SPIRU1410-11|BIOUG00633-B10|Pardosa groenlandica
KKCHE1055-09|07CHU-AR-0248|Pardosa groenlandica
KKCHE273-07|JBWM0006156m|Pardosa groenlandica
KKCHE1030-09|07CHU-AR-0223|Pardosa groenlandica
KKCHE1056-09|07CHU-AR-0249|Pardosa groenlandica
KKCHE1101-09|07CHU-AR-0294|Pardosa groenlandica
KKCHE035-06|HLC13357|Pardosa groenlandica
SPIRU1400-11|BIOUG00633-A12|Pardosa groenlandica
Pardosa groenlandica
JDTGS024-09|09-PROBE-08120|Pardosa dromaea
KKCHE1100-09|07CHU-AR-0293|Pardosa dromaea
KKCHE1029-09|07CHU-AR-0222|Pardosa dromaea
JDTGS025-09|09-PROBE-08121|Pardosa dromaea
SPIRU1399-11|BIOUG00633-A11|Pardosa dromaea
KKCHE038-06|INV0327|Pardosa dromaea
KKCHE1061-09|07CHU-AR-0254|Pardosa dromaea
SPIRU1403-11|BIOUG00633-B03|Pardosa dromaea
KKCHE020-06|HLC14154|Pardosa dromaea
KKCHE1033-09|07CHU-AR-0226|Pardosa dromaea
KKCHE309-07|JBWM0234323|Pardosa dromaea
KKCHE1014-09|07CHU-AR-0207|Pardosa dromaea
SPIRU1411-11|BIOUG00633-B11|Pardosa dromaea
TWSC278-08|07PROBE-04093|Pardosa dromaea
KKCHE1082-09|07CHU-AR-0275|Pardosa dromaea
KKCHE1069-09|07CHU-AR-0262|Pardosa dromaea
SPIRU1412-11|BIOUG00633-B12|Pardosa dromaea
KKCHE1046-09|07CHU-AR-0239|Pardosa dromaea
KKCHE1083-09|07CHU-AR-0276|Pardosa dromaea
Pardosa dromaea
TWSC027-07|07PROBE-06513|Pardosa fuscula
KKCHE868-09|HLC-26837|Pardosa fuscula
KKCHE086-06|INV0291|Pardosa fuscula
SPICH076-09|09PROBE-1929-02|Pardosa fuscula

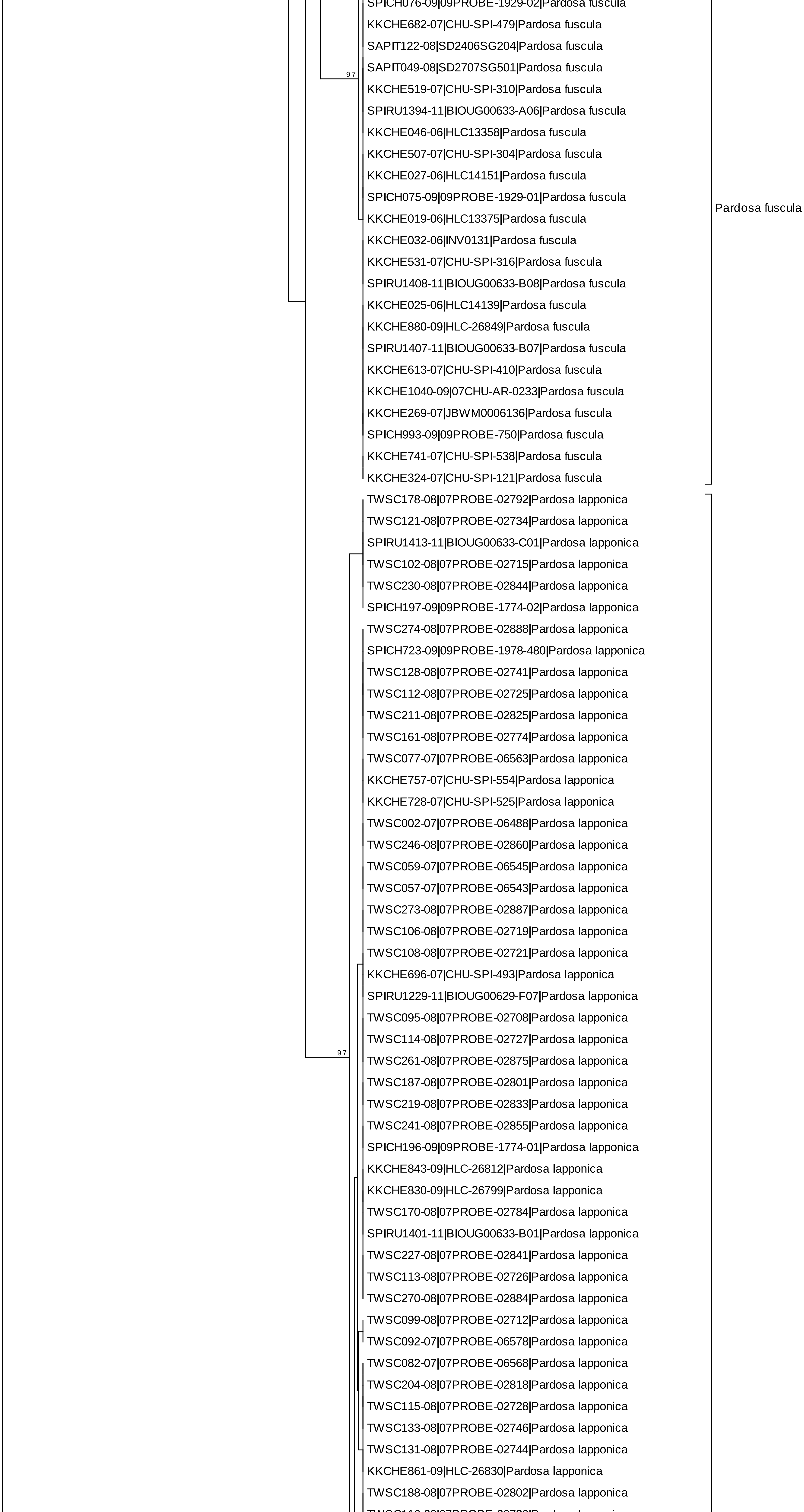
SPICH076-09|09PROBE-1929-02|Pardosa fuscula
KKCHE682-07|CHU-SPI-479|Pardosa fuscula
SAPIT122-08|SD2406SG204|Pardosa fuscula
SAPIT049-08|SD2707SG501|Pardosa fuscula
97
KKCHE519-07|CHU-SPI-310|Pardosa fuscula
SPIRU1394-11|BIOUG00633-A06|Pardosa fuscula
KKCHE046-06|HLC13358|Pardosa fuscula
KKCHE507-07|CHU-SPI-304|Pardosa fuscula
KKCHE027-06|HLC14151|Pardosa fuscula
SPICH075-09|09PROBE-1929-01|Pardosa fuscula
KKCHE019-06|HLC13375|Pardosa fuscula
Pardosa fuscula
KKCHE032-06|INV0131|Pardosa fuscula
KKCHE531-07|CHU-SPI-316|Pardosa fuscula
SPIRU1408-11|BIOUG00633-B08|Pardosa fuscula
KKCHE025-06|HLC14139|Pardosa fuscula
KKCHE880-09|HLC-26849|Pardosa fuscula
SPIRU1407-11|BIOUG00633-B07|Pardosa fuscula
KKCHE613-07|CHU-SPI-410|Pardosa fuscula
KKCHE1040-09|07CHU-AR-0233|Pardosa fuscula
KKCHE269-07|JBWM0006136|Pardosa fuscula
SPICH993-09|09PROBE-750|Pardosa fuscula
KKCHE741-07|CHU-SPI-538|Pardosa fuscula
KKCHE324-07|CHU-SPI-121|Pardosa fuscula
TWSC178-08|07PROBE-02792|Pardosa lapponica
TWSC121-08|07PROBE-02734|Pardosa lapponica
SPIRU1413-11|BIOUG00633-C01|Pardosa lapponica
TWSC102-08|07PROBE-02715|Pardosa lapponica
TWSC230-08|07PROBE-02844|Pardosa lapponica
SPICH197-09|09PROBE-1774-02|Pardosa lapponica
TWSC274-08|07PROBE-02888|Pardosa lapponica
SPICH723-09|09PROBE-1978-480|Pardosa lapponica
TWSC128-08|07PROBE-02741|Pardosa lapponica
TWSC112-08|07PROBE-02725|Pardosa lapponica
TWSC211-08|07PROBE-02825|Pardosa lapponica
TWSC161-08|07PROBE-02774|Pardosa lapponica
TWSC077-07|07PROBE-06563|Pardosa lapponica
KKCHE757-07|CHU-SPI-554|Pardosa lapponica
KKCHE728-07|CHU-SPI-525|Pardosa lapponica
TWSC002-07|07PROBE-06488|Pardosa lapponica
TWSC246-08|07PROBE-02860|Pardosa lapponica
TWSC059-07|07PROBE-06545|Pardosa lapponica
TWSC057-07|07PROBE-06543|Pardosa lapponica
TWSC273-08|07PROBE-02887|Pardosa lapponica
TWSC106-08|07PROBE-02719|Pardosa lapponica
TWSC108-08|07PROBE-02721|Pardosa lapponica
KKCHE696-07|CHU-SPI-493|Pardosa lapponica
SPIRU1229-11|BIOUG00629-F07|Pardosa lapponica
TWSC095-08|07PROBE-02708|Pardosa lapponica
TWSC114-08|07PROBE-02727|Pardosa lapponica
97
TWSC261-08|07PROBE-02875|Pardosa lapponica
TWSC187-08|07PROBE-02801|Pardosa lapponica
TWSC219-08|07PROBE-02833|Pardosa lapponica
TWSC241-08|07PROBE-02855|Pardosa lapponica
SPICH196-09|09PROBE-1774-01|Pardosa lapponica
KKCHE843-09|HLC-26812|Pardosa lapponica
KKCHE830-09|HLC-26799|Pardosa lapponica
TWSC170-08|07PROBE-02784|Pardosa lapponica
SPIRU1401-11|BIOUG00633-B01|Pardosa lapponica
TWSC227-08|07PROBE-02841|Pardosa lapponica
TWSC113-08|07PROBE-02726|Pardosa lapponica
TWSC270-08|07PROBE-02884|Pardosa lapponica
TWSC099-08|07PROBE-02712|Pardosa lapponica
TWSC092-07|07PROBE-06578|Pardosa lapponica
TWSC082-07|07PROBE-06568|Pardosa lapponica
TWSC204-08|07PROBE-02818|Pardosa lapponica
TWSC115-08|07PROBE-02728|Pardosa lapponica
TWSC133-08|07PROBE-02746|Pardosa lapponica
TWSC131-08|07PROBE-02744|Pardosa lapponica
KKCHE861-09|HLC-26830|Pardosa lapponica
TWSC188-08|07PROBE-02802|Pardosa lapponica

TWSC116-08|07PROBE-02729|Pardosa lapponica
TWSC101-08|07PROBE-02714|Pardosa lapponica
KKCHE906-09|HLC-26875|Pardosa lapponica
KKCHE648-07|CHU-SPI-445|Pardosa lapponica
TWSC014-07|07PROBE-06500|Pardosa lapponica
SPICH950-09|09PROBE-707|Pardosa lapponica
TWSC235-08|07PROBE-02849|Pardosa lapponica
KKCHE871-09|HLC-26840|Pardosa lapponica
SPIRU1402-11|BIOUG00633-B02|Pardosa lapponica
TWSC262-08|07PROBE-02876|Pardosa lapponica
KKCHE721-07|CHU-SPI-518|Pardosa lapponica
KKCHE601-07|CHU-SPI-398|Pardosa lapponica
TWSC155-08|07PROBE-02768|Pardosa lapponica
TWSC267-08|07PROBE-02881|Pardosa lapponica
TWSC097-08|07PROBE-02710|Pardosa lapponica
TWSC079-07|07PROBE-06565|Pardosa lapponica
SAPIT058-08|SD2707HE501|Pardosa lapponica
TWSC088-07|07PROBE-06574|Pardosa lapponica
KKCHE777-07|CHU-SPI-574|Pardosa lapponica
TWSC255-08|07PROBE-02869|Pardosa lapponica
KKCHE720-07|CHU-SPI-517|Pardosa lapponica
KKCHE847-09|HLC-26816|Pardosa lapponica
TWSC164-08|07PROBE-02778|Pardosa lapponica
TWSC110-08|07PROBE-02723|Pardosa lapponica
TWSC256-08|07PROBE-02870|Pardosa lapponica
SPICH952-09|09PROBE-709|Pardosa lapponica
TWSC263-08|07PROBE-02877|Pardosa lapponica
KKCHE858-09|HLC-26827|Pardosa lapponica
TWSC248-08|07PROBE-02862|Pardosa lapponica
TWSC118-08|07PROBE-02731|Pardosa lapponica
TWSC096-08|07PROBE-02709|Pardosa lapponica
TWSC072-07|07PROBE-06558|Pardosa lapponica
KKCHE756-07|CHU-SPI-553|Pardosa lapponica
KKCHE718-07|CHU-SPI-515|Pardosa lapponica
TWSC105-08|07PROBE-02718|Pardosa lapponica
KKCHE664-07|CHU-SPI-461|Pardosa lapponica
TWSC120-08|07PROBE-02733|Pardosa lapponica
KKCHE733-07|CHU-SPI-530|Pardosa lapponica
SAPIT025-08|SD2707HE601|Pardosa lapponica
SAPIT059-08|SD2707HE502|Pardosa lapponica
TWSC124-08|07PROBE-02737|Pardosa lapponica
KKCHE1026-09|07CHU-AR-0219|Pardosa lapponica
KKCHE661-07|CHU-SPI-458|Pardosa lapponica
TWSC264-08|07PROBE-02878|Pardosa lapponica
SPICH943-09|09PROBE-700|Pardosa lapponica
TWSC122-08|07PROBE-02735|Pardosa lapponica
TWSC084-07|07PROBE-06570|Pardosa lapponica
TWSC260-08|07PROBE-02874|Pardosa lapponica
TWSC277-08|07PROBE-02891|Pardosa lapponica
KKCHE041-06|HLC13418|Pardosa lapponica
SPIRU1280-11|BIOUG00630-B11|Pardosa lapponica
TWSC011-07|07PROBE-06497|Pardosa lapponica
KKCHE684-07|CHU-SPI-481|Pardosa lapponica
TWSC232-08|07PROBE-02846|Pardosa lapponica
TWSC143-08|07PROBE-02756|Pardosa lapponica
SPICH881-09|09PROBE-638|Pardosa lapponica
TWSC212-08|07PROBE-02826|Pardosa lapponica
SPIRU1210-11|BIOUG00629-D12|Pardosa lapponica
KKCHE637-07|CHU-SPI-434|Pardosa lapponica
TWSC111-08|07PROBE-02724|Pardosa lapponica
TWSC237-08|07PROBE-02851|Pardosa lapponica
SPICH951-09|09PROBE-708|Pardosa lapponica
KKCHE837-09|HLC-26806|Pardosa lapponica
SPIRU1215-11|BIOUG00629-E05|Pardosa lapponica
TWSC033-07|07PROBE-06519|Pardosa lapponica
TWSC119-08|07PROBE-02732|Pardosa lapponica
SPIRU1077-11|BIOUG00628-A10|Pardosa lapponica
KKCHE300-07|JBWM0234317f|Pardosa lapponica
TWSC104-08|07PROBE-02717|Pardosa lapponica
TWSC125-08|07PROBE-02738|Pardosa lapponica

Pardosa lapponica

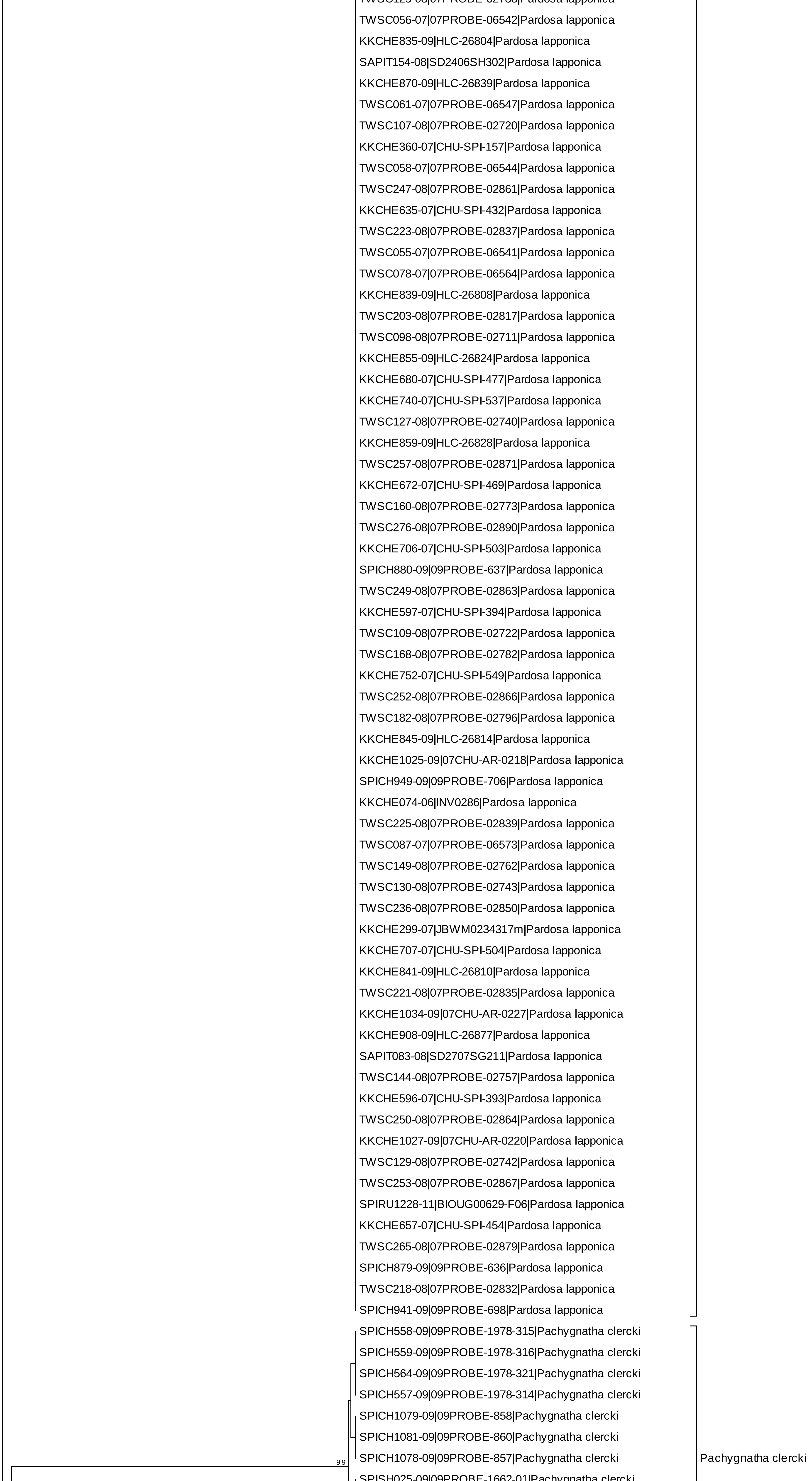
TWSC056-07|07PROBE-06542|Pardosa lapponica
KKCHE835-09|HLC-26804|Pardosa lapponica
SAPIT154-08|SD2406SH302|Pardosa lapponica
KKCHE870-09|HLC-26839|Pardosa lapponica
TWSC061-07|07PROBE-06547|Pardosa lapponica
TWSC107-08|07PROBE-02720|Pardosa lapponica
KKCHE360-07|CHU-SPI-157|Pardosa lapponica
TWSC058-07|07PROBE-06544|Pardosa lapponica
TWSC247-08|07PROBE-02861|Pardosa lapponica
KKCHE635-07|CHU-SPI-432|Pardosa lapponica
TWSC223-08|07PROBE-02837|Pardosa lapponica
TWSC055-07|07PROBE-06541|Pardosa lapponica
TWSC078-07|07PROBE-06564|Pardosa lapponica
KKCHE839-09|HLC-26808|Pardosa lapponica
TWSC203-08|07PROBE-02817|Pardosa lapponica
TWSC098-08|07PROBE-02711|Pardosa lapponica
KKCHE855-09|HLC-26824|Pardosa lapponica
KKCHE680-07|CHU-SPI-477|Pardosa lapponica
KKCHE740-07|CHU-SPI-537|Pardosa lapponica
TWSC127-08|07PROBE-02740|Pardosa lapponica
KKCHE859-09|HLC-26828|Pardosa lapponica
TWSC257-08|07PROBE-02871|Pardosa lapponica
KKCHE672-07|CHU-SPI-469|Pardosa lapponica
TWSC160-08|07PROBE-02773|Pardosa lapponica
TWSC276-08|07PROBE-02890|Pardosa lapponica
KKCHE706-07|CHU-SPI-503|Pardosa lapponica
SPICH880-09|09PROBE-637|Pardosa lapponica
TWSC249-08|07PROBE-02863|Pardosa lapponica
KKCHE597-07|CHU-SPI-394|Pardosa lapponica
TWSC109-08|07PROBE-02722|Pardosa lapponica
TWSC168-08|07PROBE-02782|Pardosa lapponica
KKCHE752-07|CHU-SPI-549|Pardosa lapponica
TWSC252-08|07PROBE-02866|Pardosa lapponica
TWSC182-08|07PROBE-02796|Pardosa lapponica
KKCHE845-09|HLC-26814|Pardosa lapponica
KKCHE1025-09|07CHU-AR-0218|Pardosa lapponica
SPICH949-09|09PROBE-706|Pardosa lapponica
KKCHE074-06|INV0286|Pardosa lapponica
TWSC225-08|07PROBE-02839|Pardosa lapponica
TWSC087-07|07PROBE-06573|Pardosa lapponica
TWSC149-08|07PROBE-02762|Pardosa lapponica
TWSC130-08|07PROBE-02743|Pardosa lapponica
TWSC236-08|07PROBE-02850|Pardosa lapponica
KKCHE299-07|JBWM0234317m|Pardosa lapponica
KKCHE707-07|CHU-SPI-504|Pardosa lapponica
KKCHE841-09|HLC-26810|Pardosa lapponica
TWSC221-08|07PROBE-02835|Pardosa lapponica
KKCHE1034-09|07CHU-AR-0227|Pardosa lapponica
KKCHE908-09|HLC-26877|Pardosa lapponica
SAPIT083-08|SD2707SG211|Pardosa lapponica
TWSC144-08|07PROBE-02757|Pardosa lapponica
KKCHE596-07|CHU-SPI-393|Pardosa lapponica
TWSC250-08|07PROBE-02864|Pardosa lapponica
KKCHE1027-09|07CHU-AR-0220|Pardosa lapponica
TWSC129-08|07PROBE-02742|Pardosa lapponica
TWSC253-08|07PROBE-02867|Pardosa lapponica
SPIRU1228-11|BIOUG00629-F06|Pardosa lapponica
KKCHE657-07|CHU-SPI-454|Pardosa lapponica
TWSC265-08|07PROBE-02879|Pardosa lapponica
SPICH879-09|09PROBE-636|Pardosa lapponica
TWSC218-08|07PROBE-02832|Pardosa lapponica
SPICH941-09|09PROBE-698|Pardosa lapponica
SPICH558-09|09PROBE-1978-315|Pachygnatha clercki
SPICH559-09|09PROBE-1978-316|Pachygnatha clercki
SPICH564-09|09PROBE-1978-321|Pachygnatha clercki
SPICH557-09|09PROBE-1978-314|Pachygnatha clercki
SPICH1079-09|09PROBE-858|Pachygnatha clercki
SPICH1081-09|09PROBE-860|Pachygnatha clercki
SPICH1078-09|09PROBE-857|Pachygnatha clercki
99
Pachygnatha clercki

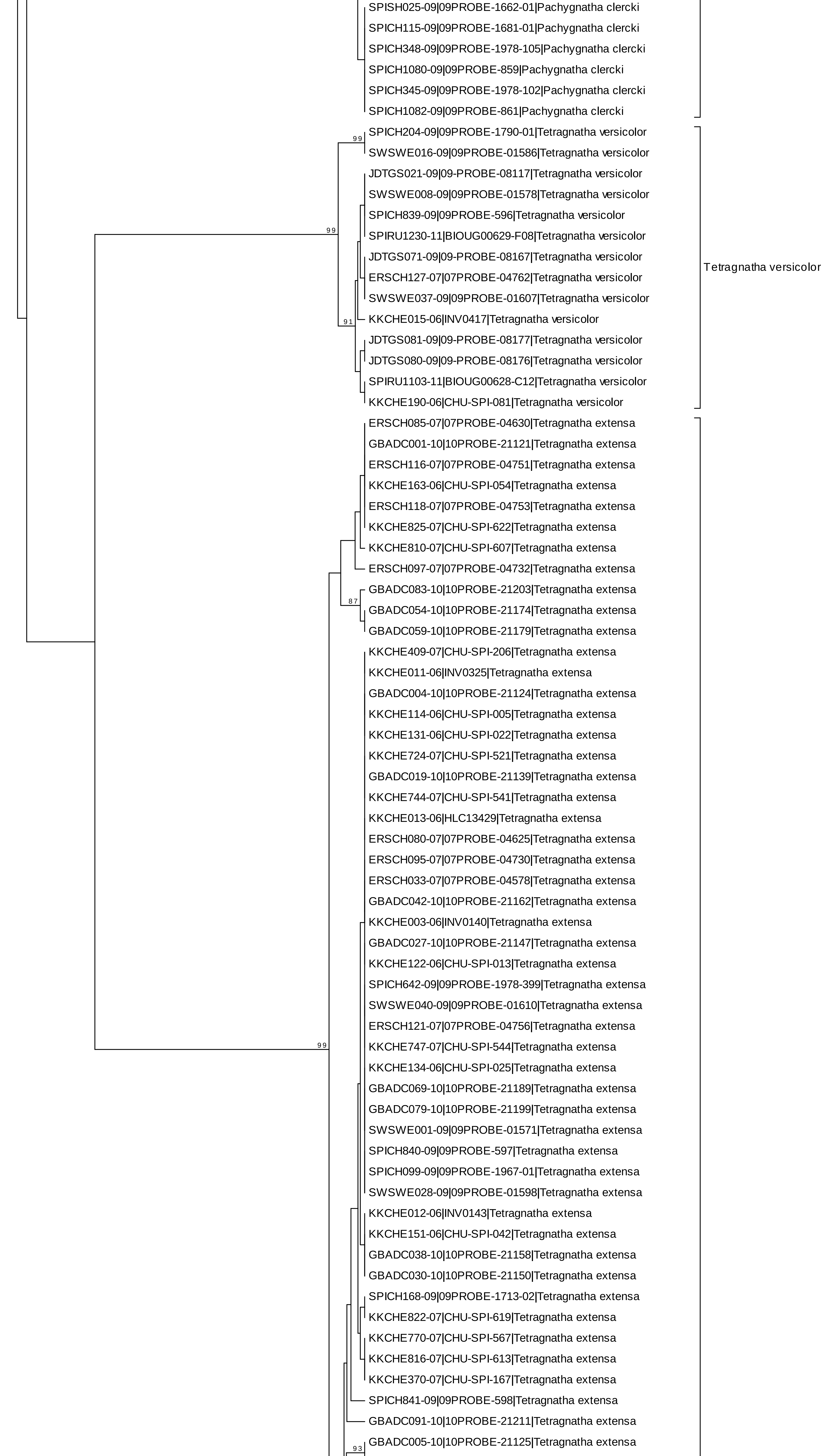
SPISH025-09|09PROBE-1662-01|Pachygnatha clercki
SPICH115-09|09PROBE-1681-01|Pachygnatha clercki
SPICH348-09|09PROBE-1978-105|Pachygnatha clercki
SPICH1080-09|09PROBE-859|Pachygnatha clercki
SPICH345-09|09PROBE-1978-102|Pachygnatha clercki
SPICH1082-09|09PROBE-861|Pachygnatha clercki
99
SPICH204-09|09PROBE-1790-01|Tetragnatha versicolor
SWSWE016-09|09PROBE-01586|Tetragnatha versicolor
JDTGS021-09|09-PROBE-08117|Tetragnatha versicolor
SWSWE008-09|09PROBE-01578|Tetragnatha versicolor
SPICH839-09|09PROBE-596|Tetragnatha versicolor
99
SPIRU1230-11|BIOUG00629-F08|Tetragnatha versicolor
JDTGS071-09|09-PROBE-08167|Tetragnatha versicolor
ERSCH127-07|07PROBE-04762|Tetragnatha versicolor
SWSWE037-09|09PROBE-01607|Tetragnatha versicolor
91
KKCHE015-06|INV0417|Tetragnatha versicolor
JDTGS081-09|09-PROBE-08177|Tetragnatha versicolor
JDTGS080-09|09-PROBE-08176|Tetragnatha versicolor
SPIRU1103-11|BIOUG00628-C12|Tetragnatha versicolor
KKCHE190-06|CHU-SPI-081|Tetragnatha versicolor
Tetragnatha versicolor
ERSCH085-07|07PROBE-04630|Tetragnatha extensa
GBADC001-10|10PROBE-21121|Tetragnatha extensa
ERSCH116-07|07PROBE-04751|Tetragnatha extensa
KKCHE163-06|CHU-SPI-054|Tetragnatha extensa
ERSCH118-07|07PROBE-04753|Tetragnatha extensa
KKCHE825-07|CHU-SPI-622|Tetragnatha extensa
KKCHE810-07|CHU-SPI-607|Tetragnatha extensa
ERSCH097-07|07PROBE-04732|Tetragnatha extensa
GBADC083-10|10PROBE-21203|Tetragnatha extensa
87
GBADC054-10|10PROBE-21174|Tetragnatha extensa
GBADC059-10|10PROBE-21179|Tetragnatha extensa
KKCHE409-07|CHU-SPI-206|Tetragnatha extensa
KKCHE011-06|INV0325|Tetragnatha extensa
GBADC004-10|10PROBE-21124|Tetragnatha extensa
KKCHE114-06|CHU-SPI-005|Tetragnatha extensa
KKCHE131-06|CHU-SPI-022|Tetragnatha extensa
KKCHE724-07|CHU-SPI-521|Tetragnatha extensa
GBADC019-10|10PROBE-21139|Tetragnatha extensa
KKCHE744-07|CHU-SPI-541|Tetragnatha extensa
KKCHE013-06|HLC13429|Tetragnatha extensa
ERSCH080-07|07PROBE-04625|Tetragnatha extensa
ERSCH095-07|07PROBE-04730|Tetragnatha extensa
ERSCH033-07|07PROBE-04578|Tetragnatha extensa
GBADC042-10|10PROBE-21162|Tetragnatha extensa
KKCHE003-06|INV0140|Tetragnatha extensa
GBADC027-10|10PROBE-21147|Tetragnatha extensa
KKCHE122-06|CHU-SPI-013|Tetragnatha extensa
SPICH642-09|09PROBE-1978-399|Tetragnatha extensa
SWSWE040-09|09PROBE-01610|Tetragnatha extensa
ERSCH121-07|07PROBE-04756|Tetragnatha extensa
99
KKCHE747-07|CHU-SPI-544|Tetragnatha extensa
KKCHE134-06|CHU-SPI-025|Tetragnatha extensa
GBADC069-10|10PROBE-21189|Tetragnatha extensa
GBADC079-10|10PROBE-21199|Tetragnatha extensa
SWSWE001-09|09PROBE-01571|Tetragnatha extensa
SPICH840-09|09PROBE-597|Tetragnatha extensa
SPICH099-09|09PROBE-1967-01|Tetragnatha extensa
SWSWE028-09|09PROBE-01598|Tetragnatha extensa
KKCHE012-06|INV0143|Tetragnatha extensa
KKCHE151-06|CHU-SPI-042|Tetragnatha extensa
GBADC038-10|10PROBE-21158|Tetragnatha extensa
GBADC030-10|10PROBE-21150|Tetragnatha extensa
SPICH168-09|09PROBE-1713-02|Tetragnatha extensa
KKCHE822-07|CHU-SPI-619|Tetragnatha extensa
KKCHE770-07|CHU-SPI-567|Tetragnatha extensa
KKCHE816-07|CHU-SPI-613|Tetragnatha extensa
KKCHE370-07|CHU-SPI-167|Tetragnatha extensa
SPICH841-09|09PROBE-598|Tetragnatha extensa
GBADC091-10|10PROBE-21211|Tetragnatha extensa
93
GBADC005-10|10PROBE-21125|Tetragnatha extensa

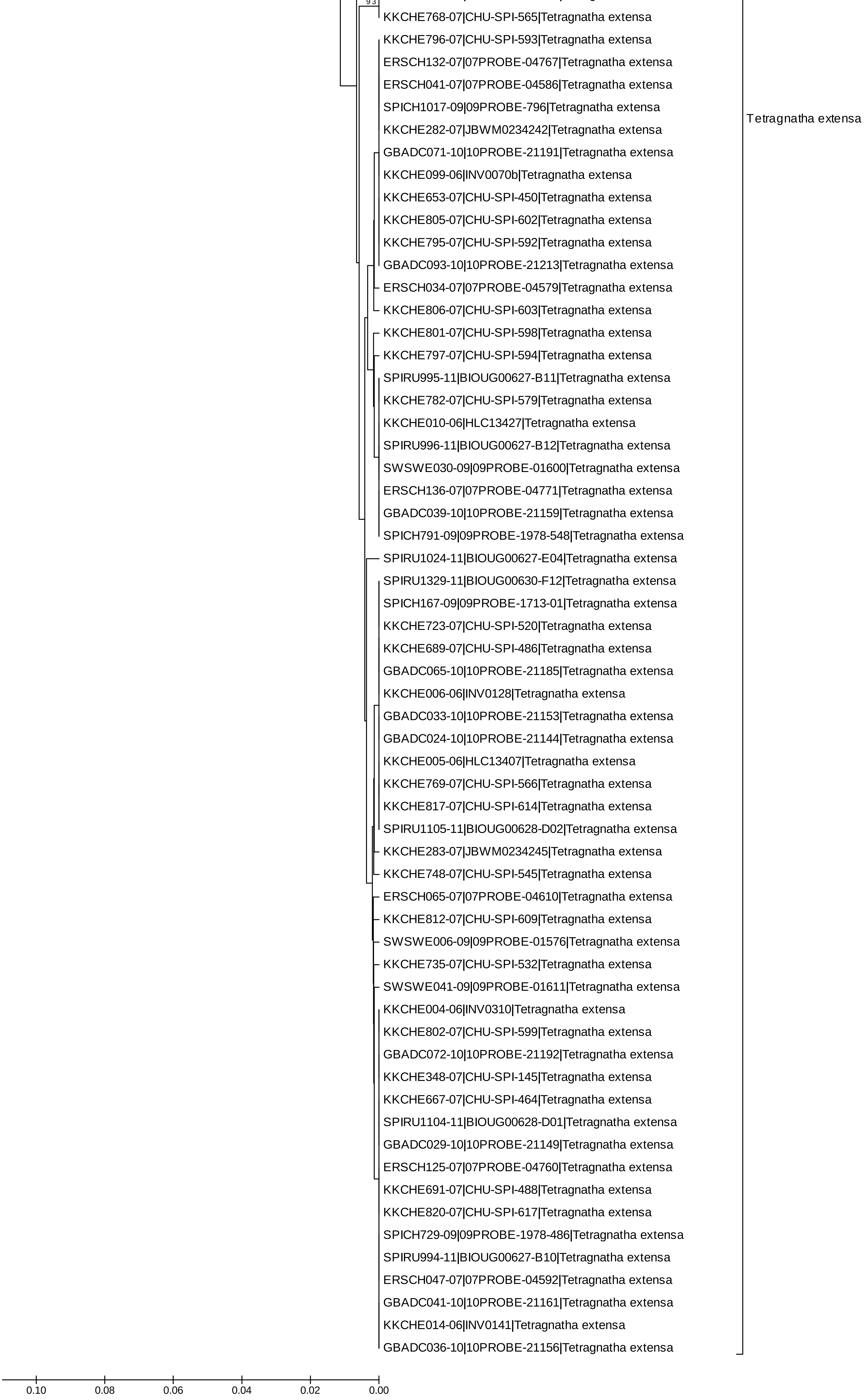
93
KKCHE768-07|CHU-SPI-565|Tetragnatha extensa
KKCHE796-07|CHU-SPI-593|Tetragnatha extensa
ERSCH132-07|07PROBE-04767|Tetragnatha extensa
ERSCH041-07|07PROBE-04586|Tetragnatha extensa
SPICH1017-09|09PROBE-796|Tetragnatha extensa
KKCHE282-07|JBWM0234242|Tetragnatha extensa
GBADC071-10|10PROBE-21191|Tetragnatha extensa
KKCHE099-06|INV0070b|Tetragnatha extensa
KKCHE653-07|CHU-SPI-450|Tetragnatha extensa
KKCHE805-07|CHU-SPI-602|Tetragnatha extensa
KKCHE795-07|CHU-SPI-592|Tetragnatha extensa
GBADC093-10|10PROBE-21213|Tetragnatha extensa
ERSCH034-07|07PROBE-04579|Tetragnatha extensa
KKCHE806-07|CHU-SPI-603|Tetragnatha extensa
KKCHE801-07|CHU-SPI-598|Tetragnatha extensa
KKCHE797-07|CHU-SPI-594|Tetragnatha extensa
SPIRU995-11|BIOUG00627-B11|Tetragnatha extensa
KKCHE782-07|CHU-SPI-579|Tetragnatha extensa
KKCHE010-06|HLC13427|Tetragnatha extensa
SPIRU996-11|BIOUG00627-B12|Tetragnatha extensa
SWSWE030-09|09PROBE-01600|Tetragnatha extensa
ERSCH136-07|07PROBE-04771|Tetragnatha extensa
GBADC039-10|10PROBE-21159|Tetragnatha extensa
SPICH791-09|09PROBE-1978-548|Tetragnatha extensa
SPIRU1024-11|BIOUG00627-E04|Tetragnatha extensa
SPIRU1329-11|BIOUG00630-F12|Tetragnatha extensa
SPICH167-09|09PROBE-1713-01|Tetragnatha extensa
KKCHE723-07|CHU-SPI-520|Tetragnatha extensa
KKCHE689-07|CHU-SPI-486|Tetragnatha extensa
GBADC065-10|10PROBE-21185|Tetragnatha extensa
KKCHE006-06|INV0128|Tetragnatha extensa
GBADC033-10|10PROBE-21153|Tetragnatha extensa
GBADC024-10|10PROBE-21144|Tetragnatha extensa
KKCHE005-06|HLC13407|Tetragnatha extensa
KKCHE769-07|CHU-SPI-566|Tetragnatha extensa
KKCHE817-07|CHU-SPI-614|Tetragnatha extensa
SPIRU1105-11|BIOUG00628-D02|Tetragnatha extensa
KKCHE283-07|JBWM0234245|Tetragnatha extensa
KKCHE748-07|CHU-SPI-545|Tetragnatha extensa
ERSCH065-07|07PROBE-04610|Tetragnatha extensa
KKCHE812-07|CHU-SPI-609|Tetragnatha extensa
SWSWE006-09|09PROBE-01576|Tetragnatha extensa
KKCHE735-07|CHU-SPI-532|Tetragnatha extensa
SWSWE041-09|09PROBE-01611|Tetragnatha extensa
KKCHE004-06|INV0310|Tetragnatha extensa
KKCHE802-07|CHU-SPI-599|Tetragnatha extensa
GBADC072-10|10PROBE-21192|Tetragnatha extensa
KKCHE348-07|CHU-SPI-145|Tetragnatha extensa
KKCHE667-07|CHU-SPI-464|Tetragnatha extensa
SPIRU1104-11|BIOUG00628-D01|Tetragnatha extensa
GBADC029-10|10PROBE-21149|Tetragnatha extensa
ERSCH125-07|07PROBE-04760|Tetragnatha extensa
KKCHE691-07|CHU-SPI-488|Tetragnatha extensa
KKCHE820-07|CHU-SPI-617|Tetragnatha extensa
SPICH729-09|09PROBE-1978-486|Tetragnatha extensa
SPIRU994-11|BIOUG00627-B10|Tetragnatha extensa
ERSCH047-07|07PROBE-04592|Tetragnatha extensa
GBADC041-10|10PROBE-21161|Tetragnatha extensa
KKCHE014-06|INV0141|Tetragnatha extensa
GBADC036-10|10PROBE-21156|Tetragnatha extensa
Tetragnatha extensa
0.10
0.08
0.06
0.04
0.02
0.00
